# Highly Regio- and Stereoselective Diels-Alder Cycloadditions via Two-Step and Multicomponent Reactions Promoted by Infrared Irradiation under Solvent-Free Conditions

**DOI:** 10.3390/ijms13032590

**Published:** 2012-02-24

**Authors:** Maria Ines Flores-Conde, Leonor Reyes, Rafael Herrera, Hulme Rios, Miguel A. Vazquez, Rene Miranda, Joaquin Tamariz, Francisco Delgado

**Affiliations:** ro. de Mayo S/N, Cuautitlán Izcalli, Estado de México 54740, Mexico; E-Mails: hulmerg@yahoo.com (H.R.); mirruv@yahoo.com.mx (R.M.); 1 Department of Organic Chemistry, National School of Biological Sciences, National Polytechnic Institute, Prol. Carpio y Plan de Ayala, S/N, 11340 Mexico, D. F., Mexico; E-Mails: maria_ines8182@yahoo.com.mx (M.I.F.-C.); lrdcistrans@yahoo.com.mx (L.R.); jtamariz@woodward.encb.ipn.mx (J.T.); 2 Institute of Chemical-Biology Research, University of Michoacan of San Nicolás de Hidalgo, Edif. B-1, Ciudad Universitaria, Francisco J. Mujica S/N, 58066 Morelia, Mich, Mexico; E-Mail: rhbucio@umich.mx; 3 Department of Chemical Sciences, Graduate School of Cuautitlán-National University of Mexico, Campo 1, Avenida 1; 4 Department of Chemistry, University of Guanajuato, Noria Alta, S/N, Guanajuato Gto. 36050, Mexico

**Keywords:** Diels-Alder cycloadditions, regioselectivity, Knoevenagel, infrared irradiation

## Abstract

Infrared irradiation promoted the Diels-Alder cycloadditions of *exo*-2-oxazolidinone dienes **1**–**3** with the Knoevenagel adducts **4**–**6**, as dienophiles, leading to the synthesis of new 3,5-diphenyltetrahydrobenzo[*d*]oxazol-2-one derivatives (**7**, **9**, **11** and **13**–**17**), under solvent-free conditions. These cycloadditions were performed with good regio- and stereoselectivity, favoring the *para*-*endo* cycloadducts. We also evaluated the one-pot three-component reaction of active methylene compounds **20**, benzaldehydes **21** and *exo*-2-oxazolidinone diene **2** under the same reaction conditions. A cascade Knoevenagel condensation/Diels-Alder cycloaddition reaction was observed, resulting in the final adducts **13**–**16** in similar yields. These procedures are environmentally benign, because no solvent and no catalyst were employed in these processes. The regioselectivity of these reactions was rationalized by Frontier Molecular Orbital (FMO) calculations.

## 1. Introduction

The Diels-Alder cycloaddition is one of the most powerful synthetic methodologies for the construction of cyclic six-membered rings, and tremendous efforts have been focused on expanding the scope of this cycloaddition with various combinations of dienes, dienophiles, catalysts and reaction conditions [1–6]. In this sense, alkenes containing two electron-withdrawing groups have been the target of a large number of recent studies, because many of them can act as Michael acceptors [7–8], as well as hetero-dienes [9–11] or dienophiles [12,13] in Diels-Alder reactions. Similarly, exocyclic dienes have received significant attention in recent years due to their high reactivity in cycloaddition reactions and their synthetic potential [14,15]. We described an efficient cascade methodology, which combines α-diketones and isocyanates in the presence of a dehydrating agent, to afford functionalized *N*-substituted *exo*-2-oxazolidinone dienes **1**–**3** (Tables 1–3). The latter have proved to be stable, and they undergo Diels-Alder cycloadditions with high selectivity [16–20]. In addition, they have shown to be useful synthons in the preparation of carbazoles [21–24], and in the synthesis of new polycyclic compounds by a cascade [4 + 2] cycloaddition/cyclopentannulation/1,5-sigmatropic rearrangement process with Fischer (arylalkynyl)(alkoxy)carbenes [25]. Moreover, dienes **1**–**3** have been employed to synthesize new η^4^-diene-Fe(CO)_3_ complexes, which undergo the addition of alkyllithium reagents to produce stable and unprecedented conjugated enamine-enol ester- and enamido-enol-Fe(CO)_3_ complexes [26].

We have appropriately employed infrared irradiation as an alternative energy source, working under solvent-free conditions and with various types of reactions, including Knoevenagel condensation [27–29], the Fischer indole reaction [30], the Biginelli reaction [31] and, more recently, the molecular rearrangement of perezone into isoperezone [32].

In this context, and as part of our ongoing research into the use of infrared irradiation as the energy source to promote organic reactions, we herein describe a convenient and versatile synthesis of the new substituted tetrahydrobenzo[*d*]oxazol-2-one derivatives **7**, **9**, **11** and **13**–**17**, starting from the *exo*-2-oxazolidinone dienes **1**–**3** and the Knoevenagel adducts **4**–**6** (Tables 1 and 2), as the dienophiles, in the Diels-Alder cycloadditions promoted by infrared irradiation, under solvent-free conditions. Moreover, we also carried out an evaluation of how the reactivity and stereoselectivity of these cycloadditions are affected by the structural modifications in the diene, as well as in the Knoevenagel adducts, such as the replacement of the cyano group by the ethoxycarbonyl group (Tables 1 and 2). In addition, we studied the one-pot three-component reactions to obtain the same cycloadducts starting from methylene active compounds **20a**–**c**, benzaldehydes **21a**–**d** and *exo*-2-oxazolidinone diene **2** via a cascade Knoevenagel/Diels-Alder process under similar reaction conditions.

## 2. Results and Discussion

### 2.1. Diels-Alder Cycloaddition with Diene 1

As we have previously demonstrated, Knoevenagel adducts can be easily prepared using an infrared irradiation protocol that employs the condensation reaction of benzaldehydes and active methylene compounds, under solvent-free conditions [27–29]. Therefore, we have used this methodology to prepare compounds **4**–**6**. The required dienes **1**–**3** were synthesized according to the already published procedure [16–20].

We explored synthetic access to the tetrahydrobenzo[*d*]oxazol-2-one derivatives **7**–**11** and **13**–**18**, in search of infrared irradiation as a viable promoter of the Diels-Alder cycloadditions, in a two-step synthesis, starting from the *exo*-heterocyclic dienes **1**–**3** and the Knoevenagel adducts **4**–**6**.

Initially, the unsubstituted *exo*-heterocyclic diene **1** was evaluated in terms of reactivity and regioselectivity in the Diels-Alder additions toward derivatives **4a**–**e**, which bear activating substituents such as ethoxycarbonyl (R_1_) and cyano (R_2_) groups. Thus, a mixture of diene **1** and olefin **4a** (1:1.2 mol-equiv., respectively) was irradiated with an infrared lamp [33] at 50 °C for ca. 3.5 h, under solvent-free conditions, leading to the total conversion of **1** to afford **7a**, judging by the ^1^H NMR analysis of the crude reaction mixture, as a single regioisomeric product in 73% yield. This high regioselectivity contrasts with that observed for the thermal Diels-Alder reaction of **1** with monosubstituted dienophiles, such as methyl vinyl ketone and methyl propiolate, in which the *para*/*meta* regioisomeric ratios were lower (from 1:1 up to 8:2) [17].

**Figure 1:**
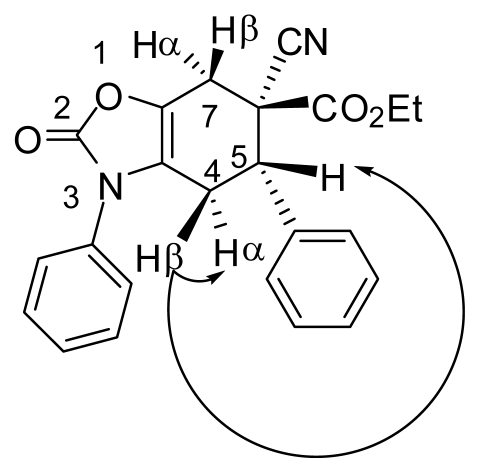
NOE effects observed upon irradiation of proton H-4β for the adduct **7a**.

The structure of compound **7a** was established by spectroscopic analysis. The spectrum of High Resolution Mass Spectrometry (HRMS) showed exactly the expected mass (*m/z* 388.1423); while the IR spectrum showed two carbonyl absorption bands (C=O) at 1757 and 1713 cm^−1^ and a cyano group absorption at 2362 cm^−1^. The ^1^H and ^13^C NMR spectral data are consistent with the tetrahydrobenzo[*d*]oxazol-2-one skeleton. It is interesting to note the large difference in the chemical shifts (*δ*) of the diastereotopic CH_2_ protons at the C-4 position of the cyclohexene ring, since H-4β appeared at 2.65 ppm as a ddd (*J* = 17.1, 4.8, 1.5 Hz) due to the geminal, vicinal and homoallylic couplings, respectively; while the signal due to H-4α appeared at 3.13 ppm as a dddd (*J* = 17.1, 11.4, 4.2, 2.1 Hz). The large difference in the *δ* value for these protons could be ascribed to the anisotropic effect of the phenyl groups at N-3 and C-5. Decoupling and Nuclear Overhauser Effect (NOE) experiments provided additional support for the structure: H-4α showed a three-bond coupling with H-5 (^3^*J*_4–5_ = 11.4 Hz); while the signal of protons H-5 (3.46 ppm) and H-4α (3.16 ppm) were enhanced when H-4β (2.65 ppm) was irradiated (Figure 1).

Interestingly, when the reaction was carried out under thermal (50 °C) and solvent-free conditions, the reaction time was longer and the yield lower (Table 1, entry 2). In an attempt to further improve the yield, under thermal conditions (50 °C), benzene and tetrahydrofuran were used as solvents, without yielding better results (Table 1, entries 3 and 4).

Comparing the reaction times (Table 1, entry 1 *vs.* entries 2–4), it appears that under infrared irradiation the reaction was substantially faster (~3.5 h) and the yield was higher (73%). As for the regioselectivity, it was comparable in both cases, only affording regioisomer **7**. Analysis of the crude reaction mixture by ^1^H NMR did not show evidence of regioisomer **8**.

To assess the effect of the substituent R_3_ in the aromatic ring of the dienophiles on the reactivity and the regioselectivity, several analogues using both electron-poor and electron-rich substituents in **4b**–**e**, were used. When **4b**, bearing an electron-releasing group, was irradiated in the presence of **1**, the conversion rate slightly decreased (Table 1, entry 5), giving **7b** in a lower yield (50%), together with recovered dienophile **4b** (50%), However, with the use of dienophile **4d**, containing an electron-withdrawing group, a higher yield of the corresponding adduct **7d** was obtained. The reactivity trend of the Diels-Alder cycloaddition of dienophiles **4a**–**e** with **1** (Table 1, entries 4–8) met the expectations of a normal electron-demand process [34].

**Figure 2:**
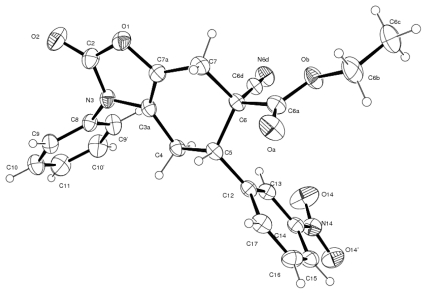
Molecular structure of **7e** with thermal ellipsoids at the 30% probability level.

Cycloadduct **7e** was isolated as yellow crystals (EtOAc/hexane, 8:2) and its *para* regiochemistry (as considered for the relative orientation in the cyclohexene ring between the nitrogen atom and the electron-withdrawing groups of the dienophile) was confirmed by X-ray crystallography (Figure 2). The X-ray structure shows that the aryl groups in N-3 and C-5 are almost perpendicular to the heterocycle and to the cyclohexene ring, respectively, presenting the following consistent torsion angles: −59.7(2)° for C(3a)-N(3)-C(8)-C(9) and −129.20(13)° for C(4)-C(5)-C(12)-C(13).

Complementarily, with the aim of exploring the scope and limitations of the process, as well as of detecting the effect on the cycloadducts induced by the change of the substituents R_1_ and R_2_ in the dienophiles, the ethoxycarbonyl group in **4** (R_1_ = CO_2_Et) was replaced by a CN group and the cyano group (R_2_ = CN) by an ethoxycarbonyl group, to produce a series of benzylidenemalononitriles **5a**–**d** (R_1_ = R_2_ = CN) and diethyl 2-benzylidenemalonates **6a**–**c** (R_1_ = R_2_ = CO_2_Et), respectively. The reactions were performed under identical conditions to those used for **1** and **4a**. The reaction of diene **1** with these two series of analogous dienophiles **5a**–**d** and **6a**–**c** yielded cycloadducts **9a**–**d** and **11a**–**c**, respectively. The fact that in both cases the product was a single *para* regioisomer indicates a similar behavior in the reactions. It is noteworthy that ^1^H NMR analysis (300 MHz) of the crude mixtures did not give evidence of the presence of the corresponding regioisomers **10** and **12**.

The best yields of the tetrahydrobenzo[*d*]oxazol-2-one derivatives **9** and **11** corresponded to the reactions between *exo*-heterocyclic diene **1** with benzylidenemalononitriles **5a**–**d** (Table 1, entries 9–12). In contrast, with the reactions between **1** and the ethyl (*E*)-2-cyano-3-phenylacrylates **4a**–**e**, the corresponding yields of derivatives **7a**–**e** were lower (Table 1, entries 1–8). When the sterically more demanding diethyl 2-benzylidenemalonates **6a**–**c** were used, the yields of adducts **11a**–**c** were the lowest of all (Table 1, entries 13–15). The reactivity trend found for the Knoevenagel dienophiles can also be explained by the higher electron-withdrawing effect of the cyano group in comparison with the ethoxycarbonyl group [35]. In accordance with previous reports, the regiochemistry of these cycloadditions mainly depends on the electron-donating effect of the nitrogen atom of the heterocycle ring of the diene [16]. However, the exclusive formation of the *para* regioisomer in our case contrasts with the tendency of the *exo*-heterocyclic diene **1** to produce a mixture of *para*/*meta* regioisomers [17]. This is probably due to the fact that the dienophiles used in the present work are geminally substituted by two electron-withdrawing groups, which enhance the reactivity and, consequently, the regioselectivity [36].

### 2.2. Diels-Alder Cycloaddition with Dienes 2 and 3

In order to evaluate the effect of the substituent in the *exo*-heterocyclic diene on the reactivity and selectivity in the course of the Diels-Alder reaction, dienes **2** and **3**, bearing a methyl group in the double bond, were added to dienophiles **4**–**6**.

The reactions of dienes **2** and **3** with acrylates **4a**–**e** (R_1_ = CO_2_Et, R_2_ = CN) gave, after IR irradiation and heating at 50 °C for 4–5 h, mixtures of *endo*/*exo* cycloadducts **13a**–**e**/**14a**–**e** in 55–88% yields (Table 2, entries 1–5). The Diels-Alder reactions were highly regio- and stereoselective, since the *para* (*N*-Ar/CO_2_Et and CN groups) derivatives **13** and **14** were the lone regioisomers, and the *para*-*endo* cycloadducts (*endo* = *syn* relative configuration between Me/CO_2_Et groups) **13a**–**e** were obtained in higher yields than the *para*-*exo* cycloadducts **14a**–**e**.

The *endo*/*exo* ratios of adducts **13a**–**e**/**14a**–**e** were determined by integration of the double signals of the methyl groups C-16 in the ^1^H NMR spectra of the crude mixtures (Table 2, entries 1–5). The separation of these mixtures was achieved by column chromatography on silica gel using hexane as eluent. The structural elucidation of the main products **13a**–**e** was made on the basis of their spectroscopic data (NMR, HRMS and IR). All the data are consistent with the substituted tetrahydrobenzo[*d*]oxazol-2-one skeleton of **13a**–**e**. The ^1^H NMR spectrum of **13a** shows the presence of ten aromatic protons at 7.26–7.51 ppm, a quartet integrating for two protons (OC*H*_2_CH_3_) at 4.09 ppm, and two overlapped signals attributed to H-7 and H-5 protons at 3.49–3.54 ppm. The proton H-4α appears as a doublet of doublets of doublets (*J* = 17.1, 11.1, 1.8 Hz) at 2.93 ppm; while the proton H-4β appears as a doublet of doublets (*J* = 17.1, 5.4 Hz) at 2.62 ppm. There is a signal at 1.35 ppm as a doublet integrating for three protons (H-16) and at 1.11 ppm (OCH_2_C*H*_3_) as a triplet.

The ^13^C NMR spectrum of **13a** displays signals for two carbonyl groups at 164.7 ppm (CO_2_Et) and 154.0 ppm (C-2), ten signals for vinyl and aromatic carbons at 138.0–120.0 ppm, one signal corresponding to the cyano group at 118.2 ppm, and seven signals at 62.9, 52.2, 40.4, 37.0, 27.0, 15.9 and 13.7 ppm for sp^3^ carbon atoms. The attributions of the signal were supported by 2D experiments such as Heteronuclear Multiple-Quantum Coherence (HMQC) and Heteronuclear Multiple-Bond Coherence (HMBC).

**Figure 3:**
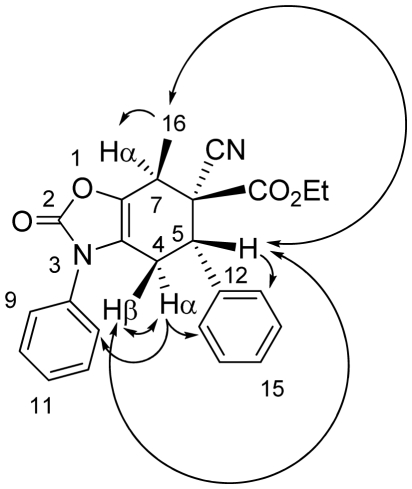
NOE observed upon irradiation of protons H-4β, H-5 and H-16 for the adduct **13a**.

The relative configuration at C-5, C-6 and C-7 of **13a** was determined by NOE experiments (Figure 3), where an enhancement of the signals of protons H-5 and H-4α was observed when the signal of H-4β was irradiated. Likewise, when H-5 was irradiated, an NOE effect was observed for H-4β and H-13. The irradiation of H-4α induced an NOE effect on the signals of H-4β, H-9 and H-13. An enhancement of the signals of protons H-5 and H-7 was observed when the signal of H-16 was irradiated. These data support a *syn* relationship between H-4β, H-5 and H-16 protons, and justify assigning the structure of the compound **13a** as the *endo* cycloadduct.

**Figure 4:**
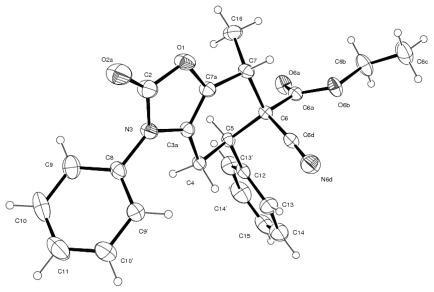
Molecular structure of **13a** with thermal ellipsoids at the 30% probability level.

The assignment of the stereochemistry of compound **13a** was confirmed by X-ray crystallography (Figure 4). The phenyl and ethoxycarbonyl groups at the stereogenic C-5 and C-6 centers have a *trans* diequatorial orientation. The torsion angle C(12)-C(5)-C(6)-C(6a) of 48.42(18)° supports the gauche conformation for the phenyl and ethoxycarbonyl groups. Meanwhile, the methyl group at the stereogenic C-7 center has a pseudoaxial orientation. Therefore, in the solid state, the carbocyclic six-membered ring adopts a half-chair conformation.

It is likely that the presence of an electron-donating methyl group in **2** greatly polarizes the π-system of the diene, giving rise to the major *para* regioisomers **13a**–**e**/**14a–e**. The *endo* preference might be due to both steric and electronic factors which favor the *endo* transition state (*vide infra*).

Similarly, benzylidenemalonitriles **5a**–**d** (R_1_ = R_2_ = CN) reacted with dienes **2** and **3** affording a mixture of *anti*/*syn* diastereoisomers **15**–**16**, respectively, in 55–90% yields, favoring the *anti* (relative configuration between the C-5 phenyl ring with respect to Me-16) cycloadducts **15a**–**e** as determined by ^1^H NMR (300 MHz) analysis of the crude reaction mixture (Table 2, entries 6–10).

In contrast, the reactions of diene **2** with dienophiles **6a**–**c** (R_1_ = R_1_ = CO_2_Et), under the same experimental condition, provided single diastereoisomers **17a**–**c** in low yields (Table 2, entries 11–13), due in part to the self-dimerization of diene **2** to adduct **19**, isolated as a by-product [17]. These results indicate that dienophiles **6a**–**c** are less reactive and more stereoselective that dienophiles **4** and **5**.

It appears that these reactions are sterically sensitive, since the use of the more hindered dienophiles **6a**–**c** afforded the corresponding products **17a**–**c** in the poorest yields, although with a better stereoselectivity.

As shown in Tables 1 and 2, the reaction times for diene **2** were similar to those employed for diene **1.** This is rather unexpected as previously mentioned [17], since the electron-releasing effect of the methyl substituent of diene **2** should increase the reactivity in Diels-Alder additions according to Alder’s rule. This behavior is also presumably due to the steric effect.

### 2.3. Multicomponent Reactions

In recent years, the development of multicomponent reactions in order to produce biologically active compounds has been accelerated and thus has become a very important area of research in organic and medicinal chemistry.

As an attempt to obtain compounds **13**–**18** more efficiently, we turned our attention to a one-pot procedure. Our synthetic strategy was based on the knowledge that the dienophiles **4** and **5** are accessible through a simple Knoevenagel condensation between compounds **20a**–**b** and benzaldehydes **21a**–**d** [27–29], followed by a subsequent Diels-Alder cycloaddition with diene **2**, to generate cycloadducts **13**–**16**.

Initially, in this multicomponent approach, a mixture of ethyl 2-cyanoacetate (**20a**), benzaldehyde (**21a**) and diene **2** was reacted in a 1:1:1 (mol-equiv.) ratio under infrared irradiation and solvent-free conditions. After 35 min, this reaction led to the desired mixture of tetrahydrobenzo[*d*]oxazol-2-ones **13a/14a** (65:35), albeit in moderate yield (55%), along with some amount of **4a** and **19** (40% and 5%, respectively). It is worth noting that the regio- and stereoselectivity was similar (Table 3, entry 1) to those found in the previous methodology (Table 2, entry 1). The Knoevenagel adduct **4a** was detected from the crude mixture by H^1^ NMR analysis, which supports the idea that its initial formation was accomplished before the intermolecular Diels-Alder reaction with diene **2** took place, to give the corresponding adducts.

A similar behavior was observed for the analogous substrates **21b**–**d** with **20a** and diene **2**, since the cycloadduct mixtures **13b/14b**, **13c/14c** and **13d/14d** were obtained in comparable yields (40–64%) to those obtained via the two-step procedure, confirming the efficiency of the multicomponent approach (Table 3, entries 2–4).

**Figure 5:**
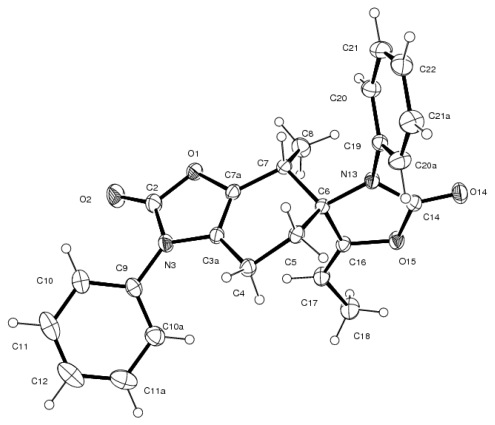
Molecular structure of **19** with thermal ellipsoids at the 30% probability level.

On the other hand, the multicomponent reaction between malononitrile **20b**, benzaldehydes **21a**–**d** and diene **2** produced fairly good yields of cycloadducts **15a**–**d/16a**–**d**. However, in the presence of diethyl malonate (**20c**), no domino Knoevenagel condensation/Diels-Alder cycloaddition was observed at all. When the reaction temperature was increased to 80 °C, compound **19** was obtained instead of the expected adducts **17**/**18**. These results revealed that the dimerization of **2** is also promoted by IR irradiation to yield **19**. The structure of the latter was established by spectroscopic data and corroborated by the study of X-ray diffraction (Figure 5). Previously, we observed the dimerization of diene **2** under thermal conditions (xylene, 120 °C, 10 h) [17]. Comparing the NMR data of these compounds, we found that there were notable differences in chemical shifts, as well as in the difference in their melting points (196–198 °C and 243–244 °C), which suggests that this dimer corresponds to different diastereoisomer. This result can be attributed to the probable influence of infrared radiation as a source of energy.

The higher reactivity of ethyl 2-cyanoacetate (**20a**) and malononitrile (**20b**) in comparison with diethyl malonate (**20c**), which successively leads to the Knoevenagel condensation and Diels-Alder reaction under infrared irradiation conditions, may be explained in terms of the difference of acidity constants of the activated methylene: **20c** (p*K*_a_ = 13) [37], **20b** (p*K*_a_ = 11) [38] and **20a** (p*K*_a_ = 9) [39]. This acidity can affect the formation of the Knoevenagel products and, consequently, the final adduct. In addition, these results also suggest that the steric hindrance generated by the ethoxycarbonyl group seems to play a role in controlling the domino reactions and therefore in providing acceptable yields.

### 2.4. Diels-Alder Regioselectivity and FMO Theory

The regioselectivity of the Diels-Alder additions of dienes **1**–**3** to dienophiles **4**–**6** was rationalized in terms of the FMO theory [34]. The geometries of dienes **1** and **2** were previously calculated [17], while the geometries of diene **3** and dienophiles **4**–**6** were calculated using the B3LYP/6-31G** method [40–42] without any symmetry constraints calculation, and employed as the starting point for the *ab initio* molecular orbital calculations, using the RHF/6-31G** basis set [43]. It is noteworthy that for derivatives **6**, the *cis* ethoxycarbonyl group to the aryl ring adopts a preferential non-coplanar conformation, leaving the *trans* acrylate moiety in conjugation with the aromatic substituent. This conjugation is also observed for derivatives **4**. This is probably due to the fact that in this conformation the aryl ring is maintained coplanar to the acrylate conjugated π-system, giving rise to a higher stability.

By using the same basis set, the energies of the FMO were calculated for both dienes and dienophiles (Table 4). Since, in the entire series lower, energy gaps were calculated for the interaction between HOMO_diene_-LUMO_dienophile_ (Normal Electronic Demand) than between the opposite interaction LUMO_diene_-HOMO_dienophile_ (Inverse Electronic Demand), as illustrated by some examples in Table 5, it is then expected that the reaction is conducted under the former interaction.

As expected, the methyl group attached to the diene moiety in dienes **2** and **3** induced an increase of the energy of the HOMO, with respect to the energy of the unsubstituted diene **1**. Hence, the reactivity of dienes **2** and **3** should be higher than that of diene **1**, as observed for the cycloadditions with mono substituted dienophiles [17]. Nevertheless, in the case of dienophiles **4**–**6**, the reaction times are very similar for all the dienes (Tables 1 and 2), which indicates a similar reactivity as well. It is likely that other factors are involved, such as the steric hindrance generated between the dienes and the substituents in dienophiles **4**–**6**. Although these factors are not sufficiently important to modify the regioselectivity, which is *para* in the whole series, the preference for the *anti* relative configuration between the methyl group and the aryl ring in adducts **13**–**17** seems to support their existence. Moreover, in spite of the presence of electron-withdrawing groups in the aryl ring of the dienophiles, such as the nitro group, which may induce a higher reactivity and higher selectivity [34,36], there is no correlation between the stereoselectivity and the structure of the dienophiles bearing other substituents. Once again, this suggests the significant effect of the steric repulsions at the transition state, and also seems to be the reason for the formation of the single *endo* stereoisomer (**17**) in the case of the more hindered dienophiles **6** (Table 2, entries 11–13). The stabilizing secondary orbital interactions eventually present at the *endo* transition state may reinforce this preference.

The exclusive *para* regioselectivity (*N*-Ar/CO_2_Me or CN groups) observed in all the cycloadditions can be explained on the basis of the coefficient differences for the HOMO_diene_-LUMO_dienophile_ interactions (Table 4). These latter should generate the greatest perturbation, since the energy gap is smaller than the inverse interactions (LUMO_diene_-HOMO_dienophile_). Indeed, if the largest FMO coefficients become bonded preferentially at the transition state [44–47], and considering that the relative magnitude of the coefficient of the terminus C-4 is bigger than that of C-1 in the HOMO of dienes **1**–**3**, and that the *beta* C-1 coefficient is bigger than that of the *alpha* C-2 in the LUMO of olefins **4**–**6**, a “*para*” orientation is expected, in agreement with the experimental results. This *para* regioselectivity supports the idea that the electronic effects also control the course of the reaction, despite the presence of steric interactions generated between the methyl group and the geminal disubstituted carbon at the vicinal carbons in the adducts **13**–**17**.

Therefore, for these cycloadditions, the regio and stereoselectivities can be ascribed to the electronic effects, which are due to the polarization of the π-systems, and to the steric interactions, the latter mainly caused by the polysubstitution of the dienophiles.

## 3. Experimental Section

### 3.1. General Procedures and Instrumentation

All reactions were carried out under nitrogen in anhydrous solvents. All glassware was dried in an oven prior to use. All commercially available compounds were used without further purification. Tetrahydrofuran and benzene were distilled from sodium benzophenone ketyl under an N_2_ atmosphere prior to use. *n*-Hexane and ethyl acetate were distilled before use. Melting points (uncorrected) were determined with a Fisher-Johns melting point apparatus. ^1^H NMR and ^13^C NMR spectra were recorded on a Varian Mercury (300 MHz) and Varian VNMR System (500 MHz) instruments, in CDCl_3_ as solvent and with TMS as internal reference. High-resolution mass spectra (HRMS) were obtained with a JSM-GCMate II mass spectrometer, and electron impact techniques (70 eV) were employed. X-ray data were collected on Siemens P4 and Oxford Diffraction Xcalibur S single-crystal X-ray difractometers. Thin-layer Chromatography (TLC) analyses were performed using silica plates and were visualized using UV (254 nm) or iodine. The Knoevenagel adducts **4a**–**e**, **5a**–**d and 6a**–**c** [27–29] and the *exo*-2-oxazolidinone dienes **1**–**3** [16–20] were prepared by the methods described in the literature.

### 3.2. General Procedures for the Synthesis of Adducts **7a–e**, **9a–d**, **11a–c**, **13a–e/14a–e**, **15a–e/16a–e** and **17a–c** via a Two-Step Reaction. Method A

A mixture of the Knoevenagel adducts **4a**–**e**, **5a**–**d**, or **6a**–**c** (1.2 equiv.) and the corresponding dienes, **1**, **2**, or **3** (1 mol-equiv.) was placed in a 25 mL two-necked, round-bottomed flask (equipped with a reflux condenser, a rubber septum and under nitrogen atmosphere), and the mixture was stirred and was irradiated with an infrared lamp [33] at 50 °C for ~30 min–6 h under solvent-free conditions until the consumption of the diene (*tlc*). The reaction mixture was allowed to cool to room temperature, and then purified by column chromatography over silica gel (230–400 mesh) using *n*-hexane/EtOAc (98:2) as eluent, to afford the corresponding cycloadducts **7a**–**e**, **9a**–**d**, **11a–c**, **13a**–**e**/**14a**–**e**, **15a**–**e/16a**–**e** and **17a**–**c**.

### 3.3. General Procedure for the Synthesis of Adducts **13a**–**d**/**14a**–**d** and **15a**–**d/16a**–**d** via a One-Step Reaction. Method B

A mixture of active methylene compounds **20a**–**c** (1 mol-equiv.), benzaldehydes **21a**–**d** (mol-equiv.) and the corresponding diene **2** (1 mol-equiv.), was placed in a 25 mL two-necked, round-bottomed flask (equipped with a reflux condenser, a rubber septum and under nitrogen atmosphere), and the mixture was stirred and was irradiated with an infrared lamp [33] at 50 °C for ~30 min–6 h, under solvent-free conditions, until the consumption of the diene (*tlc*). The reaction mixture was allowed to cool to room temperature, and then was purified by column chromatography on silica gel (230–400 mesh) using *n*-hexane/EtOAc (98:2) as eluent, to afford the corresponding cycloadducts **13a**–**d**/**14a**–**d** and **15a**–**d/16a**–**d**.

(5S*,6R*)-6-Ethoxycarbonyl-6-cyano-3,5-diphenyl-4,5,6,7-tetrahydrobenzo[*d*]oxazol-2-one (**7a**). According to Method A, the reaction between **4a** (0.330 g, 0.0016 mol) and diene **1** (0.250 g, 0.0013 mol), followed by flash column chromatography, afforded **7a** (0.380 g, 73%) as a white solid: mp 145–146 °C; FT-IR (KBr) *ν*_max_ 2928, 2362, 1757, 1713, 1598 cm^−1; 1^H NMR (500 MHz, CDCl_3_) *δ* 0.87 (t, *J* = 6.9 Hz, 3H, OCH_2_C*H*_3_), 2.65 (ddd, *J* = 17.1, 4.8, 1.5 Hz, 1H, H-4β), 3.13 (dddd, *J* = 17.1, 11.4, 4.2, 2.1 Hz, 1H, H-4α), 3.16 (dd, *J* = 16.8, 1.5 Hz, 1H, H-7α), 3.43–3.48 (m, 2H, H-5, H-7β), 3.92 (q, *J* = 6.9, 2H, OC*H*_2_CH_3_), 7.32–7.46 (m, 10H, H-Ar); ^13^C NMR (125 MHz, CDCl_3_) *δ* 13.3 (OCH_2_*C*H_3_), 25.6 (C-4), 31.4 (C-7), 46.5 (C-5), 49.6 (C-6), 63.2 (O*C*H_2_CH_3_), 117.2 (*C*N), 120.4 (C-3a), 125.0 (C-9), 125.1 (C-10), 128.0 (C-13), 128.1 (C-11), 128.8 (C-14), 129.5 (15), 130.1 (C-7a), 133.3 (C-8), 136.4 (C-12), 154.0 (C-2), 166.5 (*C*O_2_CH_2_CH_3_); HRMS (EI^+^) calcd for C_22_H_16_N_4_O_4_ 388.1423, found (M^+^) 388.1436.

(5S*,6R*)-6-Ethoxycarbonyl-6-cyano-5-(4-methoxyphenyl)-3-(phenyl)-4,5,6,7-tetrahydrobenzo[*d*] oxazol-2-one (**7b**). According to Method A, the reaction between **4b** (0.44 g, 0.0019 mol) and diene **1** (0.300 g, 0.0016 mol), followed by flash column chromatography, afforded **7b** (0.330 g, 50%) as a pale yellow solid: mp 168–169 °C; FT-IR (KBr) *ν*_max_ 2934, 2244, 1769, 1716, 1598 cm^−1; 1^H NMR (500 MHz, CDCl_3_) *δ* 0.94 (t, *J =* 7.2 Hz, 3H, OCH_2_C*H*_3_), 2.61 (ddd, *J* = 16.2, 4.8, 1.2 Hz, 1H, H-4β), 3.06 (dddd, *J* = 16.2, 11.4, 4.2, 1.2 Hz, 1H, H-4α), 3.14 (dd, *J* = 16.2, 2.1 Hz, 1H, 7α), 3.39–3.46 (m, 2H, H-5, H-7β), 3.78 (s, 3H, OCH_3_), 3.96 (q, *J* = 7.2 Hz, 2H, OC*H*_2_CH_3_), 6.85 (d, *J* = 8.7 Hz, 2H, H-14), 7.30 (d, *J* = 8.7 Hz, 2H, H-13), 7.36–7.48 (m, 5H, H-Ar); ^13^C NMR (125 MHz, CDCl_3_) *δ* 13.4 (OCH_2_*C*H_3_), 25.6 (C-4), 30.1 (C-7), 45.6 (C-5), 49.8 (C-6), 55.2 (O*C*H_3_), 63.1 (O*C*H_2_CH_3_), 114.3 (C-14), 117.5 (*C*N), 120.8 (C-3a), 125.4 (C-9), 128.3 (C-11), 128.6 (C-12), 129.6 (C-13), 129.9 (C-10), 130.4 (C-7a), 133.5 (C-8), 154.0 (C-2), 159.7 (C-15), 166.5 (*C*O_2_CH_2_CH_3_); HRMS (EI^+^) calcd for C_16_H_16_N_2_O_3_ 418.1529, found (M^+^) 418.1526.

(5S*,6R*)-6-Ethoxycarbonyl-5-(4-chlorophenyl)-6-cyano-3-(phenyl)-4,5,6,7-tetrahydrobenzo[*d*]oxazol-2-one (**7c**). According to Method A, the reaction between **4c** (0.452 g, 0.0019 mol) and diene **1** (0.300 g, 0.0016 mol), followed by flash column chromatography, afforded **7c** (0.400 g, 60%) as a pale yellow solid: mp 175–177 °C; FT-IR (KBr) *ν*_max_ 2910, 2256, 1762, 1710, 1566 cm^−1; 1^H NMR (300 MHz, CDCl_3_) *δ* 0.96 (t, *J =* 7.0 Hz, 3H, OCH_2_C*H*_3_), 2.63 (ddd, *J* = 16.8, 4.8, 1.8 Hz, 1H, H-4β), 3.05 (dddd, *J* = 16.8, 11.4, 4.8, 2.1 Hz, 1H, H-4α), 3.16 (dd, *J* = 16.5, 1.8 Hz, 1H, H-7α), 3.41 (ddd, *J* = 16.5, 4.2, 1.8 Hz, 1H, H-7β), 3.45 (dd, *J* = 11.4, 4.8 Hz, 1H, H-5), 3.98 (qd, *J* = 7.2, 2.0 Hz, 2H, OC*H*_2_CH_3_), 7.30–7.47 (m, 9H, H-Ar); ^13^C NMR (75.4 MHz, CDCl_3_) *δ* 13.5 (OCH_2_*C*H_3_), 25.5 (C-4), 31.3 (C-7), 45.7 (C-5), 49.4 (C-6), 63.3 (O*C*H_2_CH_3_), 116.9 (*C*N), 120.2 (C-3a), 125.1 (C-9), 128.2 (C-11), 129.0 (C-10), 129.6 (C-13), 129.7 (C-14), 130.0 (C-7a), 133.2 (C-8), 134.8 (C-15), 134.9 (C-12), 154.0 (C-2), 166.4 (*C*O_2_CH_2_CH_3_); HRMS (EI^+^) calcd for C_23_H_19_N_2_O_4_Cl 422.1033, found (M^+^) 422.1032.

(5S*,6R*)-6-Ethoxycarbonyl-6-cyano-5-(4-nitrophenyl)-3-(phenyl)-4,5,6,7-tetrahydrobenzo[*d*]oxazol-2-one (**7d**). According to Method A, the reaction between **4d** (0.47 g, 0.0019 mol) and diene **1** (0.300 g, 0.0016 mol), followed by flash column chromatography, afforded **7d** (0.550 g, 80%) as a pale yellow solid: mp 161–163 °C; FT-IR (KBr) *ν*_max_ 2982, 2246, 1770, 1743, 1600 cm^−1; 1^H NMR (300 MHz, CDCl_3_) *δ* 0.97 (t, *J =* 6.5 Hz, 3H, OCH_2_C*H*_3_), 2.68 (ddd, *J* = 18.5, 4.5, 1.5 Hz, 1H, H-4β), 3.09 (dddd, *J* = 18.5, 11.5, 4.5, 2.0 Hz, 1H, H-4α), 3.22 (dd, *J* = 16.5 Hz, 0.5, 1H, H-7α), 3.43 (ddd, *J* = 16.5, 4.5, 2.0 Hz, 1H, H-7β), 3.61 (dd, *J* = 12.0, 4.5 Hz, 1H, H-5), 4.01 (qd, *J* = 6.5, 1.6 Hz, 2H, OC*H*_2_CH_3_), 7.36–7.48 (m, 5H, H-Ar), 7.27 (d, *J* = 9.0 Hz, 2H, H-13), 8.21 (d, *J* = 9.0 Hz, 2H, H-14); ^13^C NMR (75.4 MHz, CDCl_3_) *δ* 13.5 (OCH_2_*C*H_3_), 25.4 (C-4), 30.5 (C-7), 45.7 (C-5), 49.0 (C-6), 63.6 (O*C*H_2_CH_3_), 116.5 (*C*N), 119.9 (C-3a), 123.9 (C-9), 125.0 (C-10), 128.3 (C-11), 129.4 (C-13), 129.7 (C-14), 129.7 (C-8), 133.0 (C-7a), 143.6 (C-12), 148.0 (C-15), 153.8 (C-2), 166.0 (*C*O_2_CH_2_CH_3_); HRMS (EI_+_) calcd for C_23_H_19_N_3_O_6_ 433.1274, found (M^+^) 433.1272.

(5S*,6R*)-6-Ethoxycarbonyl-6-cyano-5-(3-nitrophenyl)-3-(phenyl)-4,5,6,7-tetrahydrobenzo[*d*]oxazol-2-one (**7e**). According to Method A, the reaction between **4e** (0.413 g, 0.0019 mol) and diene **1** (0.300 g, 0.0016 mol), followed by flash column chromatography, afforded **7e** (0.380 g, 55%) as a pale yellow solid: mp 180–181 °C; FT-IR (KBr) *ν*_max_ 2925, 2244, 1771, 1741, 1531 cm^−1; 1^H NMR (500 MHz, CDCl_3_) *δ* 0.96 (t, *J =* 7.2 Hz, 3H, OCH_2_C*H*_3_), 2.69 (ddd, *J* = 16.8, 4.8, 1.5 Hz, 1H, H-4β), 3.12 (dddd, *J* = 16.8, 11.4, 5.7, 2.1 Hz, 1H, H-4α), 3.22 (dd, *J* = 16.8, 1.5 Hz, 1H, H-7α), 3.45 (ddd, *J* = 16.8, 3.9, 2.1 Hz, 1H, H-7β), 3.65 (dd, *J* = 11.4, 4.8 Hz, 1H, H-5), 4.01 (qd, *J* = 7.2, 1.8 Hz, 2H, OC*H*_2_CH_3_), 7.35–7.50 (m, 5H, H-Ar), 7.57 (t, *J* = 7.5 Hz, 1H, H-16), 7.83 (d, *J* = 7.5 Hz, 1H, H-17), 8.21 (dd, *J* = 7.5, 1.8 Hz, 1H, H-15), 8.26 (dd, *J* = 7.5, 1.8 Hz, 1H, H-13); ^13^C NMR (125 MHz, CDCl_3_) *δ* 13.9 (OCH_2_*C*H_3_), 24.7 (C-4), 31.9 (C-7), 45.8 (C-5), 49.5 (C-6), 64.0 (O*C*H_2_CH_3_), 116.3 (*C*N), 120.0 (C-3a), 123.7(C-13), 123.8 (C-15), 125.0 (C-9), 128.2 (C-11), 129.4 (C-10), 130.2 (C-7a), 130.3 (C-17), 133.3 (C-8), 134.2 (C-16), 139.0 (C-12), 148.2 (C-14), 153.8 (C-2), 166.2 (*C*O_2_CH_2_CH_3_). HRMS (EI^+^) calcd for C_23_H_19_N_3_O_6_ 433.1273, found (M^+^) 433.1273.

6,6-Dicyano-3,5-diphenyl-4,5,6,7-tetrahydrobenzo[*d*]oxazol-2-one (**9a**). According to Method A, the reaction between **5a** (0.345 g, 0.0022 mol) and diene **1** (0.350 g, 0.0018 mol), followed by flash column chromatography, afforded **9a** (0.510 g, 80%) as a pale yellow solid: mp 150–151 °C; FT-IR (KBr) *ν*_max_ 2933, 2251, 1769, 1720, 1501 cm^−1; 1^H NMR (300 MHz, CDCl_3_) *δ* 2.76 (dd, *J* = 17.1, 5.4 Hz, 1H, H-4β), 3.02–3.13 (m, 2H, H-4α, H-7α), 3.41–3.49 (m, 1H, H-7β), 3.46 (dd, *J* = 10.8, 5.4 Hz, 1H, H-5), 7.35–7.50 (m, 10H, H-Ar); ^13^C NMR (75.4 MHz, CDCl_3_) *δ* 24.5 (C-4), 32.5 (C-6), 37.3 (C-7), 46.6 (C-5), 113.3 (*C*N), 113.6 (*C*N), 121.1 (C-3a), 125.3 (C-9), 127.9 (C-15), 128.0 (C-14), 128.5 (C-11), 129.7 (C-13), 129.8 (C-10), 129.8 (C-7a), 132.8 (C-8), 134.9 (C-12), 153.6 (C-2); HRMS (EI^+^) calcd for C_21_H_15_N_3_O_2_ 341.1164, found (M^+^) 341.1165.

6,6-Dicyano-5-(4-methoxyphenyl)-3-phenyl-4,5,6,7-tetrahydrobenzo[*d*]oxazol-2-one (**9b**). According to Method A, the reaction between **5b** (0.436 g, 0.0023 mol), and diene **1** (0.370 g, 0.0019 mol) followed by flash column chromatography, afforded **9b** (0.400 g, 55%) as a pale yellow solid: mp 157–159 °C; FT-IR (KBr) *ν*_max_ 2935, 2252, 2217, 1768, 1598 cm^−1; 1^H NMR (300 MHz, CDCl_3_) *δ* 2.73 (dd, *J* = 16.8, 4.8 Hz, 1H, H-4β), 2.97–3.03 (m, 1H, H-4α), 3.39–4.01 (m, 2H, H-7α, H-7β), 3.44 (dd, *J* = 10.5, 5.1 Hz, 1H, H-5), 3.78 (OC*H*_3_), 6.92 (d, *J* = 8.7 Hz, 2H, H-14), 7.33–7.39 (m, 5H, H-Ar), 7.45 (d, *J* = 8.5 Hz, 2H, H-13); ^13^C NMR (75.4 MHz, CDCl_3_) *δ* 24.4 (C-4), 32.2 (C-7), 37.6 (C-6), 45.7 (C-5), 55.2 (O*C*H_3_), 113.4 (*C*N), 113.7 (*C*N), 114.5 (C-14), 121.0 (C-3a), 125.1 (C-9), 126.8 (C-11), 127.9 (C-13), 129.3 (C-10), 129.4 (C-12), 130.8 (C-7a), 132.8 (C-8), 153.6 (C-2), 160.3 (C-15); HRMS (EI^+^) calcd for C_22_H_17_N_3_O_3_ 371.1270, found (M^+^) 371.1270.

5-(4-Chlorophenyl)-6,6-dicyano-3-phenyl-4,5,6,7-tetrahydrobenzo[*d*]oxazol-2-one (**9c**). According to Method A, the reaction between **5c** (0.482 g, 0.0025 mol) and diene **1** (0.400 g, 0.0021 mol), followed by flash column chromatography, afforded **9c** (0.60 g, 75%) as a pale yellow solid: mp 179–181 °C; FT-IR (KBr) *ν*_max_ 2923, 2215, 1778, 1549 cm^−1; 1^H NMR (300 MHz, CDCl_3_) *δ* 2.75 (dd, *J* = 17.5, 5.0 Hz, 1H, H-4β), 3.01–3.07 (m, 1H, H-4α), 3.42–3.47 (m, 3H, H-5, H-7α, H-7β), 7.35–7.50 (m, 9H, H-Ar); ^13^C NMR (75.4 MHz, CDCl_3_) *δ* 24.4 (C-4), 29.6 (C-7), 37.2 (C-6), 46.1 (C-5), 113.1 (*C*N), 113.4 (*C*N), 120.8 (C-3a), 125.3 (C-9), 127.9 (C-7a), 128.6 (C-11), 129.7, 129.7, 129.8, 129.9 (C-10, C-13, C-14), 132.8 (C-8), 133.3 (C-15), 136.0 (C-12), 153.6 (C-2); HRMS (EI^+^) calcd for C_21_H_14_ClN_3_O_2_ 375.0774, found (M^+^) 375.0774.

6,6-Dicyano-5-(4-nitrophenyl)-3-phenyl-4,5,6,7-tetrahydrobenzo[*d*]oxazol-2-one (**9d**). According to Method A, the reaction between **5d** (0.383 g, 0.0019 mol) and diene **1** (0.300 g, 0.0016 mol) followed by flash column chromatography, afforded **9d** (0.525 g, 85%) as a pale yellow solid: mp 166–167 °C; FT-IR (KBr) *ν*_max_ 2918, 2220, 1772, 1599 cm^−1; 1^H NMR (300 MHz, CDCl_3_) *δ* 2.80 (dd, *J* = 17.7, 5.1 Hz, 1H, H-4β), 3.01–3.16 (m, 1H, H-4α), 3.45–3.50 (m, 2H, H-7α, H-7β), 3.60 (dd, *J* = 10.5, 5.1 Hz, 1H, H-5), 7.30–7.55 (m, 5H, H-Ar), 7.62 (d, *J* = 8.5 Hz, 2H, H-13), 8.30 (d, *J* = 8.5 Hz, 2H, H-14); ^13^C NMR (75.4 MHz, CDCl_3_) *δ* 24.3 (C-4), 32.6 (C-7), 36.8 (C-6), 46.3 (C-5), 113.0 (*C*N), 113.3 (*C*N), 120.5 (C-3a), 124.5 (C-14), 125.3 (C-9), 127.8 (C-7a), 128.8 (C-11), 129.4 (C-13), 129.9 (C-10), 133.0 (C-8), 141.5 (C-12), 149.0 (C-15), 153.0 (C-2); HRMS (EI^+^) calcd for C_21_H_14_N_4_O_4_ 386.1015, found (M^+^) 386.1015.

6,6-Diethoxycarbonyl-3,5-diphenyl-4,5,6,7-tetrahydrobenzo[*d*]oxazol-2-one (**11a**). According to Method A, the reaction between **6a** (0.445 g, 0.0018 mol) and diene **1** (0.280 g, 0.0015 mol), followed by flash column chromatography, afforded **11a** (0.243 g, 35%) as a white solid: mp 124–125 °C; FT-IR (KBr) *ν*_max_ 2926, 1770, 1732, 1502 cm^−1; 1^H NMR (300 MHz, CDCl_3_) *δ* 1.16 (t, *J* = 7.2 Hz, 3H, OCH_2_C*H*_3_), 1.20 (t, *J* = 6.9 Hz, 3H, OCH_2_C*H*_3_), 2.58 (d, *J* = 16.8 Hz, 1H, H-4α), 3.11 (ddd, *J* = 16.8, 3.9, 2.4 Hz, 1H, H-7β), 3.22–3.32 (m, 2H, H-4α, H-7α), 3.94 (dd, *J* = 7.2, 2.4 Hz, 1H, H-5), 4.09 (qd, *J* = 7.2, 4.8 Hz, 2H, OC*H*_2_CH_3_), 4.19 (qd, *J* = 6.9, 4.8 Hz, 2H, OC*H*_2_CH_3_), 7.13–7.45 (m, 10H, H-Ar); ^13^C NMR (75.4 MHz, CDCl_3_) *δ* 13.8 (OCH_2_*C*H_3_), 13.8 (OCH_2_*C*H_3_), 24.5 (C-4), 25.4 (C-7), 42.5 (C-5), 58.3 (C-6), 61.8 (O*C*H_2_CH_3_), 62.1 (O*C*H_2_CH_3_), 120.2 (C-3a), 125.1 (C-13), 127.8 (C-11), 127.8 (C-15), 128.0 (C-10), 128.7 (C-9), 129.4 (C-14), 132.1 (C-7a), 133.6 (C-8), 139.8 (C-12), 154.5 (C-2), 168.3 (*C*O_2_CH_2_CH_3_), 169.5 (*C*O_2_CH_2_CH_3_); HRMS (EI^+^) calcd for C_25_H_25_NO_6_ 435.1681, found (M^+^) 435.1681.

6,6-Diethoxycarbonyl-5-(4-methoxyphenyl)-3-phenyl-4,5,6,7-tetrahydrobenzo[*d*]oxazol-2-one (**11b**). According to Method A, the reaction between **6b** (0.624 g, 0.0022 mol) and diene **1** (0.350 g, 0.0018 mol), followed by flash column chromatography, afforded **11b** (0.215 g, 25%) as a white solid: mp 140–141 °C; FT-IR (KBr) *ν*_max_ 2929 1769, 1729, 1504 cm^−1; 1^H NMR (300 MHz, CDCl_3_) *δ* 1.20–1.23 (m, 6H, OCH_2_C*H*_3_), 2.85 (d, *J* = 16.8, 1H, H-4α), 3.11 (ddd, *J* = 16.8, 3.6, 2.2 Hz, 1H, H-7β), 3.23–3.32 (m, 2H, H-4β, H-7α), 3.48–3.56 (m, 1H, H-5), 3.76 (s, 3H, OC*H*_3_), 4.08–4.18 (m, 4H, OC*H*_2_CH_3_), 6.78 (d, *J* = 9.0 Hz, 2H, H-13), 7.07 (d, *J* = 9.0 Hz, 2H, H-14), 7.31–7.42 (m, 5H, H-Ar); ^13^C NMR (75.4 MHz, CDCl_3_) *δ* 13.8 (OCH_2_*C*H_3_), 13.9 (OCH_2_*C*H_3_), 24.8 (C-7), 25.4 (C-4), 42.5 (C-5), 55.1 (C-6), 61.8 (O*C*H_2_CH_3_), 62.1 (O*C*H_2_CH_3_), 113.8 (C-14), 120.2 (C-3a), 125.1 (C-9), 127.8 (C-11), 129.0 (C-13), 129.4 (C-12), 131.8 (C-10), 132.1 (C-7a), 154.4 (C-2), 159.0 (C-15), 168.5 (*C*O_2_CH_2_CH_3_), 169.5 (*C*O_2_CH_2_CH_3_); HRMS (EI^+^) calcd for C_26_H_27_NO_7_ 465.1787, found (M^+^) 465.1787.

5-(4-Chlorophenyl)-6,6-diethoxycarbonyl-3-phenyl-4,5,6,7-tetrahydrobenzo[*d*]oxazol-2-one (**11c**). According to Method A, the reaction between **6c** (0.542 g, 0.0019 mol) and diene **1** (0.300 g, 0.0016 mol), followed by flash column chromatography, afforded **11c** (0.225 g, 30%) as a white solid: mp 167–169 °C; FT-IR (KBr) *ν*_max_ 2981, 1770, 1732, 1503 cm^−1; 1^H NMR (300 MHz, CDCl_3_) *δ* 1.18 (t, *J* = 7.5 Hz, 3H, OCH_2_C*H*_3_), 1.19 (t, *J* = 7.5 Hz, 3H, OCH_2_C*H*_3_), 2.57 (dd, *J =* 17.1, 2.4 Hz, 1H, H-4α), 3.08 (ddd, *J =* 17.1, 3.3, 2.4 Hz, 1H, H-7β), 3.18–3.30 (m, 2H, H-4β, H-7α), 3.90 (dd, *J* = 6.9, 2.4 Hz, 1H, H-5), 4.05–4.21 (m, 2H, OC*H*_2_CH_3_), 7.10 (d, *J* = 8.7 Hz, 2H, H-13), 7.24 (d, *J* = 8.7 Hz, 2H, H-14), 7.30–7.46 (m, 5H, H-Ar); ^13^C NMR (75.4 MHz, CDCl_3_) *δ* 13.8 (OCH_2_*C*H_3_), 13.9 (OCH_2_*C*H_3_), 24.8 (C-7), 25.4 (C-4), 42.2 (C-5), 58.2 (C-6), 61.9 (O*C*H_2_CH_3_), 62.2 (O*C*H_2_CH_3_), 120.0 (C-3a), 125.1 (C-9), 127.9 (C-11), 128.8 (C-14), 129.4 (C-13), 129.5 (C-10), 132.0 (C-15), 133.7 (C-7a), 138.3 (C-12), 154.4 (C-2), 168.2 (*C*O_2_CH_2_CH_3_), 169.3 (*C*O_2_CH_2_CH_3_); HRMS (EI^+^) calcd for C_25_H_24_NO_6_Cl 469.1292, found (M^+^) 469.1292.

(5R*,6S*,7R*)-6-Cyano-6-ethoxycarbonyl-7-methyl-3,5-diphenyl-4,5,6,7-tetrahydrobenzo[*d*]oxazol-2-one (**13a**). (5R*,6S*,7S*)-6-Cyano-6-ethoxycarbonyl-7-methyl-3,5-diphenyl-4,5,6,7-tetrahydrobenzo [*d*]oxazol-2-one (**14a**). According to Method A, the reaction between **4a** (0.480 g, 0.0023 mol) and diene **2** (0.400 g, 0.0020 mol) gave a mixture of isomers **13a**/**14a** (80:20) as a white solid. The isomers were separated by flash column chromatography, giving 0.450 g (56%) of **13a** as white solid, mp 165–166 °C and 0.250 g (32%) of **14a** as a pale yellow solid, mp 165–167 °C. Data of **13a**: FT-IR (KBr) *ν*_max_ 2984, 2362, 1769, 1750, 1500 cm^−1; 1^H NMR (500 MHz, CDCl_3_) *δ* 1.11 (t, *J =* 7.2 Hz, 3H, OCH_2_C*H*_3_), 1.35 (d, *J* = 6.9 Hz, 3H, H-16), 2.62 (dd, *J* = 17.1, 5.4 Hz, 1H, H-4β), 2.93 (ddd, *J* = 17.1, 11.1, 1.8 Hz, 1H, H-4α), 3.49–3.54 (m, 2H, H-5, H-7), 4.09 (q, *J* = 7.2 Hz, 2H, OC*H*_2_CH_3_), 7.26–7.51 (m, 10H, H-Ar); ^13^C NMR (125 MHz, CDCl_3_) *δ* 13.7 (OCH_2_*C*H_3_), 15.9 (C-16), 27.0 (C-4), 37.0 (C-7), 40.4 (C-5), 52.2 (C-6), 62.9 (O*C*H_2_CH_3_), 118.2 (*C*N), 120.0 (C-3a), 125.2 (C-9), 128.1 (C-11), 128.2 (C-15), 128.4 (C-14), 128.7 (C-13), 129.6 (C-10), 133.3 (C-7a), 134.6 (C-8), 138.0 (C-12), 154.0 (C-2), 164.7 (*C*O_2_CH_2_CH_3_); Data of **14a**. FT-IR (KBr) *ν*_max_ 2984, 2262, 1769, 1750, cm^−1; 1^H NMR (500 MHz, CDCl_3_) *δ* 0.86 (t, *J =* 7.2 Hz, 3H, OCH_2_C*H*_3_), 1.42 (d, *J* = 6.9 Hz, 3H, H-16), 3.13 (ddd, *J* = 17.1, 11.7, 4.5 Hz, 1H, H-4α), 3.48–3.53 (m, 2H, H-5, H-7), 3.95 (q, *J* = 7.2 Hz, 2H, OC*H*_2_CH_3_), 7.24–7.58 (m, 10H, H-Ar); ^13^C NMR (125 MHz, CDCl_3_) *δ* 13.4 (OCH_2_*C*H_3_), 15.7 (C-16), 26.8 (C-4), 36.4 (C-7), 154.1 (C-2); HRMS (EI^+^) calcd for C_24_H_22_N_2_O_4_ 402.1579, found (M^+^) 402.1580.

Method B. Reaction of ethyl 2-cyanoacetate **20a** (0.200 g, 0.0017 mol), benzaldehyde **21a** (0.184 g, 0.0017 mol) with *exo*-2-oxazolidinone diene **2** (0.350 g, 0.0017 mol) gave a mixture of isomers **13a**/**14a** (65:35). The isomers were separated by flash column chromatography, giving 0.340 g (43%) of **13a** and 0.095 g (12%) of **14a**.

(5R*,6S*,7R*)-6-Cyano-6-ethoxycarbonyl-5-(4-methoxyphenyl)-7-methyl-3-phenyl-4,5,6,7-tetrahydrobenzo[*d*]oxazol-2-one (**13b**). (5R*,6S*,7S*)-6-Cyano-6-ethoxycarbonyl-5-(4-methoxyphenyl)-7-methyl-3-phenyl-4,5,6,7-tetrahydrobenzo[*d*]oxazol-2-one (**14b**). According to Method A, the reaction between **4b** (0.480 g, 0.0020 mol) and diene **2** (0.350 g, 0.0017 mol) gave a mixture of isomers **13b**/**14b** (75:25) as a pale yellow solid, which was purified by flash column chromatography, to yield 0.520 g (65%) of major isomer **13b** as pale yellow solid: mp 172–174 °C. Data of **13b**: FT-IR (KBr) *ν*_max_ 2933, 2200, 1767, 1754, 1501 cm^−1; 1^H NMR (300 MHz, CDCl_3_) *δ* 1.14 (t, *J =* 7.2 Hz, 3H, OCH_2_C*H*_3_), 1.37 (d, *J* = 6.9 Hz, 3H, H-16), 2.59 (dd, *J* = 17.1, 4.8 Hz, 1H, H-4β), 2.86–2.95 (m, 1H, H-4α), 3.46–3.51 (m, 2H, H-5, H-7), 3.83 (s, 3H, OC*H*_3_), 4.09 (q, *J* = 7.2 Hz, 2H, OC*H*_2_CH_3_), 6.84 (d, *J =* 8.7 Hz, 2H, H-14), 7.27–7.47 (m, 7H, H-Ar). Signals attributed to minor isomer **14b**: 0.92 (t, *J =* 7.2 Hz, 3H, OCH_2_C*H*_3_), 1.42 (d, *J* = 6.9 Hz, 3H, H-16), 2.38 (dd, *J* = 17.1, 4.8 Hz, 1H, H-4β), 3.08 (dd, *J* = 16.8, 10.9 Hz, 1H, H-4α), 3.43–3.60 (m, 2H, H-5, H-7), 3.96 (q, *J* = 7.2 Hz, 2H, OC*H*_2_CH_3_); ^13^C NMR (75.4 MHz, CDCl_3_) *δ* 14.0 (OCH_2_*C*H_3_), 16.2 (C-16), 27.2 (C-7), 31.2 (C-4), 37.1 (C-5), 52.8 (C-6), 55.4 (O*C*H_3_), 63.1 (O*C*H_2_CH_3_), 114.2 (C-14), 118.0 (CN), 120.3 (C-3a), 125.4 (C-9), 127.5 (C-11), 129.5 (C-13), 129.8 (C-10), 130.0 (C-8), 133.4 (C-7a), 134.8 (C-12), 152 (C-2), 159.5 (*C*O_2_CH_2_CH_3_), 165.0 (C-15); HRMS (EI^+^) calcd for C_25_H_24_N_2_O_5_ 432.1685, found (M^+^). 432.1681.

Method B. Reaction of ethyl 2-cyanoacetate **20a** (0.190 g, 0.0017 mol) and benzaldehyde **21b** (0.230 g, 0.0017 mol) with *exo*-2-oxazolidinone diene **2** (0.350 g, 0.0017 mol) gave a mixture of isomers **13b**/**14b** (75:25). The isomers were separated by flash column chromatography, giving 0.300 g (40%) of major isomer **13b**.

(5R*,6S*,7R*)-5-(4-Chlorophenyl)-6-cyano-6-ethoxycarbonyl-7-methyl-3-phenyl-4,5,6,7-tetrahydrobenzo[*d*]oxazol-2-one (**13c**). (5R*,6S*,7S*)-5-(4-Chlorophenyl)-6-cyano-6-ethoxycarbonyl-7-methyl-3-phenyl-4,5,6,7-tetrahydrobenzo[*d*]oxazol-2-one (14c). According to Method A, the reaction between **4c** (0.500 g, 0.0020 mol) and diene **2** (0.350 g, 0.0017 mol) gave a mixture of isomers **13c**/**14c** (90:10) as a pale yellow solid, which was purified by flash column chromatography, to yield 0.570 g (75%) of major isomer **13c** as pale yellow solid: mp 182–184 °C. Data of **13c**: FT-IR (KBr) *ν*_max_ 2931, 2230, 1773, 1719, 1544 cm^−1; 1^H NMR (300 MHz, CDCl_3_) *δ* 1.16 (t, *J* = 6.9 Hz, 3H, OCH_2_C*H*_3_), 1.33 (d, *J* = 6.9 Hz, 3H, H-16), 2.58 (dd, *J* = 16.8, 5.1 Hz, 1H, H-4β), 2.89 (ddd, *J* = 16.8, 11.1, 1.6 Hz, 1H, H-4α), 3.46–3.54 (m, 2H, H-5, H-7), 4.11 (qd, *J* = 6.9, 1.5 Hz, 2H, OC*H*_2_CH_3_), 7.28–7.47 (m, 9H, H-Ar); ^13^C NMR (75.4 MHz, CDCl_3_) *δ* 13.7 (OCH_2_*C*H_3_), 15.8 (C-16), 27.0 (C-4), 37.0 (C-7), 40.1 (C-5), 52.2 (C-6), 62.9 (O*C*H_2_CH_3_), 118.2 (*C*N), 120.0 (C-3a), 125.2 (C-9), 128.1 (C-10), 128.4 (C-11), 129.4 (C-13), 129.6 (C-14), 133.2 (C-8), 134.5 (C-7a), 146.0 (C-12), 148.0 (C-15), 154.0 (C-2), 164.2 (*C*O_2_CH_2_CH_3_); HRMS (EI^+^) calcd for C_24_H_21_N_2_O_4_Cl 436.1190, found (M^+^) 436.1186.

Method B. Reaction of ethyl 2-cyanoacetate **20a** (0.17 g, 0.0015 mol) and benzaldehyde **21c** (0.200 g, 0.0015 mol) with *exo*-2-oxazolidinone diene **2** (0.300 g, 0.0015 mol) gave a mixture of isomers **13c**/**14c** (68:32). The isomers were separated by flash column chromatography, giving 0.456 g (64%) of major isomer **13c**.

(5R*,6S*,7R*)-6-Cyano-6-ethoxycarbonyl-7-methyl-5-(4-nitrophenyl)-3-phenyl-4,5,6,7-tetrahydrobenzo[*d*]oxazol-2-one (**13d**). (5R*,6S*,7S*)-6-Cyano-6-ethoxycarbonyl-7-methyl-5-(4-nitrophenyl)-3-phenyl-4,5,6,7-tetrahydrobenzo[*d*]oxazol-2-one (14d). According to Method A, the reaction between **4d** (0.513 g, 0.0020 mol) and diene **2** (0.350 g, 0.0017 mol) gave a mixture of isomers **13d**/**14d** (85:15) as a pale yellow solid, which was purified by flash column chromatography, to yield 0.660 g (80%) of major isomer **13d** as white solid: mp 178–180 °C. Data of **13d**: FT-IR (KBr) *ν*_max_ 3078, 2983, 2240, 1764, 1710, 1520 cm^−1; 1^H NMR (300 MHz, CDCl_3_) *δ* 1.19 (t, *J =* 6.9 Hz, 3H, OCH_2_C*H*_3_), 1.34 (d, *J* = 7.0 Hz, 3H, H-16), 2.64 (dd, *J* = 16.5, 4.8 Hz, 1H, H-4β), 2.94 (ddd, *J* = 16.5, 11.4, 1.5 Hz, 1H, H-4α), 3.56–3.67 (m, 2H, H-5, H-7), 4.14 (qdd, *J* = 6.9, 4.5, 2.7 Hz, 2H, OC*H*_2_CH_3_), 7.37–7.45 (m, 5H, H-Ar), 7.71 (d, *J* = 8.7 Hz, 2H, H-13), 8.19 (d, *J* = 8.7 Hz, 2H, H-14). Signals attributed to minor isomer **14d**: 0.95 (t, *J =* 6.9 Hz, 3H, OCH_2_C*H*_3_), 1.47 (d, *J* = 7.0 Hz, 3H, H-16), 3.12 (ddd, *J* = 16.5, 11.4, 1.5 Hz, 1H, H-4α), 3.99 (qdd, *J* = 6.9, 4.5, 2.7 Hz, 2H, OC*H*_2_CH_3_); ^13^C NMR (75.4 MHz, CDCl_3_) *δ* 13.8 (OCH_2_*C*H_3_), 15.7 (C-16), 26.7 (C-4), 37.0 (C-7), 40.0 (C-5), 51.7 (C-6), 63.4 (O*C*H_2_CH_3_), 117.6 (CN), 119.4 (C-3a), 123.7 (C-9), 125.2 (C-10), 128.3 (C-11), 129.4 (C-13), 129.6 (C-14), 133.0 (C-8), 133.1 (C-7a), 134.3 (C-12), 147.6 (C-15), 153.8 (C-2), 164.4 (*C*O_2_CH_2_CH_3_); HRMS (EI^+^) calcd for C_24_H_21_N_3_O_6_ 447.1430, found (M^+^). 447.1450.

Method B. Reaction of ethyl 2-cyanoacetate **20a** (0.17 g, 0.0015 mol) and benzaldehyde **21d** (0.220 g, 0.0015 mol) with *exo*-2-oxazolidinone diene **2** (0.300 g, 0.0015 mol) gave a mixture of isomers **13d**/**14d** (75:25). The isomers were separated by flash column chromatography, giving 0.419 g (64%) of major isomer **13d**.

(5R*,6S*,7R*)-3-(4-Chlorophenyl)-6-cyano-6-ethoxycarbonyl-7-methyl-5-(3-nitrophenyl)-4,5,6,7-tetrahydrobenzo[*d*]oxazol-2-one (**13e**). (5R*,6S*,7S*)-3-(4-Chlorophenyl)-6-cyano-6-ethoxycarbonyl-7-methyl-5-(3-nitrophenyl)-4,5,6,7-tetrahydrobenzo[*d*]oxazol-2-one (**14e**). According to Method A, the reaction between **4e** (0.280 g, 0.0015 mol) and diene **3** (0.300 g, 0.0012 mol) gave a mixture of isomers **13e**/**14e** (75:25) as a pale yellow solid, which was purified by flash column chromatography, to yield 0.430 g (70%) **13e** as a pale yellow solid: mp 180–182 °C; FT-IR (KBr) *ν*_max_ 2926, 2230, 1772, 1749, 1529 cm^−1; 1^H NMR (300 MHz, CDCl_3_) *δ* 1.19 (t, *J =* 7.2 Hz, 3H, OCH_2_C*H*_3_), 1.34 (d, *J* = 6.9 Hz, 3H, H-16), 2.67 (dd, *J* = 17.2, 5.4 Hz, 1H, H-4β), 2.95 (ddd, *J* = 17.2, 11.1, 1.8 Hz, 1H, H-4α), 3.57 (q, *J* = 6.9 Hz, 1H, H-7), 3.65 (dd, *J* = 11.1, 5.4 Hz, 1H, H-5), 4.13 (q, *J* = 7.2 Hz, 2H, OC*H*_2_CH_3_), 7.33 (d, *J* = 8.7 Hz, 2H, H-9), 7.43 (d, *J* = 8.7 Hz, 2H, H-10), 7.55 (t, *J* = 8.1 Hz, 1H, H-17), 7.91 (d, *J* = 8.1 Hz, 1H, H-18), 8.18 (d, *J* = 8.4, 3 Hz, 1H, H-15), 8.36 (dd, *J* = 1.8, 1.2 Hz, 1H, H-13). Signals attributed to minor isomer **14e**: 0.92 (t, *J =* 7.2 Hz, 3H, OCH_2_C*H*_3_), 1.42 (d, *J* = 6.9 Hz, 3H, H-16), 3.17 (ddd, *J* = 17.1, 11.1, 1.8 Hz, 1H, H-4α), 4.02 (qd, *J* = 7.2, 2.1 Hz, 2H, OC*H*_2_CH_3_), 7.82 (d, *J* = 8.1 Hz, 1H, H-17), 8.21 (d, *J* = 8.4 Hz, 1H, H-15); ^13^C NMR (75.4 MHz, CDCl_3_) *δ* 13.8 (OCH_2_*C*H_3_), 15.8 (C-16), 26.7 (C-4), 36.9 (C-7), 39.9 (C-5), 51.8 (C-6), 63.4 (O*C*H_2_CH_3_), 117.5 (*C*N), 119.2 (C-3a), 123.3 (C-11), 124.3 (C-17), 126.4 (C-13), 129.9 (C-10), 134.0 (C-8), 134.1 (C-9), 134.7 (C-15), 139.9 (C-18), 144.0 (C-14), 148.0 (C-2), 164.3 (*C*O_2_CH_2_CH_3_); HRMS (EI^+^) calcd for C_24_H_20_ClN_3_O_6_ 481.1040, found (M^+^) 481.1039.

(5R*,7R*)-6,6-Dicyano-7-methyl-3,5-diphenyl-4,5,6,7-tetrahydrobenzo[*d*]oxazol-2-one (**15a**). (5R*,7S*)-6,6-Dicyano-7-methyl-3,5-diphenyl-4,5,6,7-tetrahydrobenzo[*d*]oxazol-2-one (**16a**). According to Method A, the reaction between **5a** (0.386 g, 0.0025 mol) and diene **2** (0.420 g, 0.0020 mol) gave a mixture of isomers **15a**/**16a** (80:20) as a pale yellow solid, which was purified by flash column chromatography, to yield 0.520 g (70%) of major isomer **15a** as pale yellow solid: mp 145–147 °C. Data of **15a**: FT-IR (KBr) *ν*_max_ 2979, 2210, 2215, 1777, 1523 cm^−1; 1^H NMR (300 MHz, CDCl_3_) *δ* 1.71 (d, *J =* 7.2 Hz, 3H, H-16), 2.70 (ddd, *J* = 17.1, 4.8, 2.1 Hz, 1H, H-4β), 3.14 (ddd, *J* = 17.1, 11.4, 4.0 Hz, 1H, H-4α), 3.48 (dd, *J* = 11.4, 4.8 Hz, 1H, H-5), 3.54–3.58 (m, 1H, H-7), 7.35–7.50 (m, 10H, H-Ar). Signals attributed to minor isomer **16a**: 1.67 (d, *J =* 7.2 Hz, 3H, H-16); ^13^C NMR (75.4 MHz, CDCl_3_) *δ* 13.8 (C-16), 24.8 (C-4), 38.6 (C-7), 45.7 (C-6), 47.6 (C-5), 112.0 (*C*N*)*, 113.5 (*C*N), 120.7 (C-3a), 125.2 (C-9),128.0 (C-11), 128.5 C (15), 129.4 (C-14), 129.7 (C-13), 129.8 (C-10), 131.9 (C-7a), 132.2 (C-12), 135.3 (C-8), 154.0 (C-2); HRMS (EI^+^) calcd for C_22_H_17_N_3_O_2_ 355.1320, found (M^+^) 355.1319.

Method B. Reaction of malononitrile **20b** (0.114 g, 0.0017 mol) and benzaldehyde **21a** (0.184 g, 0.0017 mol) with *exo*-2-oxazolidinone diene **2** (0.350 g, 0.0017 mol) gave a mixture of isomers **15a**/**16a** (70:30). The isomers were separated by flash column chromatography, giving 0.340 g (55%) of major isomer **15a**.

(5R*,7R*)-6,6-Dicyano-5-(4-methoxyphenyl)-7-methyl-3-phenyl-4,5,6,7-tetrahydrobenzo[*d*]oxazol-2-one (**15b**). (5R*,7R*)-6,6-Dicyano-5-(4-methoxyphenyl)-7-methyl-3-phenyl-4,5,6,7-tetrahydrobenzo [*d*]oxazol-2-one (**16b**). According to Method A, the reaction between **5b** (0.60 g, 0.0030 mol) and diene **2** (0.500 g, 0.0024 mol) gave a mixture of isomers **15b**/**16b** (82:18) as a pale yellow solid, which was purified by flash column chromatography, to yield 0.528 g (55%) of major isomer **15b** as pale yellow solid: mp 150–152 °C. Data of **15b**: FT-IR(KBr) *ν*_max_ 2977, 2936, 2235, 2230, 1776, 1597, cm^−1; 1^H NMR (300 MHz, CDCl_3_) *δ* 1.68 (d, *J =* 7.2 Hz, 3H, H-16), 2.66 (ddd, *J* = 17.1, 4.8, 2.1 Hz, 1H, H-4β), 3.08 (ddd, *J* = 17.1, 11.4, 3.9 Hz, 1H, H-4α), 3.47 (dd, *J* = 11.4, 4.8 Hz, 1H, H-5) 3.52 (qdd, *J* = 7.2, 3.9, 2.1 Hz, 1H, H-7), 3.80 (s, 3H, OCH_3_), 6.92 (d, *J* = 8.7 Hz, 2H, H-14), 7.34–7.48 (m, 7H, H-9, H-10, H-11, H-13). Signals attributed to minor isomer **16b**: 1.67 (d, *J =* 7.2 Hz, 3H, H-16); ^13^C NMR (75.4 MHz, CDCl_3_) *δ* 13.8 (C-16), 24.7 (C-4), 38.3 (C-7), 46.1 (C-6), 46.7 (C-5), 55.2 (O*C*H_3_), 111.8 (*C*N*)*, 113.4 (*C*N),114.5 (C-14), 120.4 (C-3a), 125.2 (C-9), 127.2 (C-12), 128.4 (C-11), 129.3 (C-13), 129.7 (C-10), 132.0 (C-7a), 132.9 (C-8), 153.7 (C-2), 160.3 (C-15); HRMS (EI^+^) calcd for C_23_H_19_N_3_O_3_ 385.1426, found (M^+^) 385.1411.

Method B. Reaction of malononitrile **20b** (0.114 g, 0.0017 mol) and benzaldehyde **21b** (0.236 g, 0.0017 mol) with *exo*-2-oxazolidinone diene **2** (0.350 g, 0.0017 mol) gave a mixture of isomers **15b**/**16b** (85:15). The isomers were separated by flash column chromatography, giving 0.478 g (50%) of major isomer **15b**.

(5R*,7R*)-5-(4-Chlorophenyl)-6,6-dicyano-7-methyl-3-phenyl-4,5,6,7-tetrahydrobenzo[*d*]oxazol-2-one (**15c**). (5R*,7S*)-5-(4-Chlorophenyl)-6,6-dicyano-7-methyl-3-phenyl-4,5,6,7-tetrahydrobenzo[*d*] oxazol-2-one (**16c**). According to Method A, the reaction between **5c** (0.336 g, 0.0017 mol) and diene **2** (0.300g, 0.0015 mol) gave a mixture of isomers **15c**/**16c** (90:10) as a pale yellow solid, which was purified by flash column chromatography, to yield 0.435 g (75%) of major isomer **15c** as pale yellow solid: mp 172–173 °C. Data of **15c**: FT-IR (KBr) ν_max_ 2926, 2230, 1754, 1520 cm^−1; 1^H NMR (500 MHz, CDCl_3_) *δ* 1.62 (d, *J =* 6.9 Hz, 3H, H-16), 2.94 (dd, *J* = 8.5, 3.0 Hz, 1H, H-4β), 3.31 (ddd, *J* = 17.1, 11.4, 3.9, Hz, 1H, H-4α), 3.91–3.99 (m, 1H, H-7), 4.03 (dd, *J* = 11.4, 5.1 Hz, 1H, H-5), 7.40–7.66 (m, 9H, H-Ar). Signals attributed to minor isomer **16c**; ^13^C NMR (125 MHz, CDCl_3_) *δ* 14.5 (C-16), 24.3 (C-4), 38.6 (C-7), 43.2 (C-5), 47.3 (C-6), 112.1 (*C*N*)*, 113.0 (*C*N), 125.7 (C-9), 128.2 (C-14), 128.4 (C-11), 129.0 (C-13), 130.3, (C-10), 131.0 (C-15), 132.0 (C-7a), 133.4 (C-8), 134.0 (C-12), 154.0 (C-2); HRMS (EI^+^) calcd for C_22_H_16_N_3_O_2_Cl 389.0931, found (M^+^) 389.0901.

Method B. Reaction of malononitrile **20b** (0.131 g, 0.0019 mol) and benzaldehyde **21c** (0.027 g, 0.0019 mol) with *exo*-2-oxazolidinone diene **2** (0.400 g, 0.0019 mol) gave a mixture of isomers **15c**/**16c** (70:30). The isomers were separated by flash column chromatography, giving 0.425 g (55%) of **15c** and 0.11 g (15%) of **16c**.

(5R*,7R*)-6,6-Dicyano-7-methyl-5-(4-nitrophenyl)-3-phenyl-4,5,6,7-tetrahydrobenzo[*d*]oxazol-2-one (**15d**). (5R*,7S*)-6,6-Dicyano-7-methyl-5-(4-nitrophenyl)-3-phenyl-4,5,6,7-tetrahydrobenzo[*d*]oxazol-2-one (**16d**). According to Method A, the reaction between **5d** (0.53 g, 0.0026 mol) and diene **2** (0.450 g, 0.0022 mol) gave a mixture of isomers **15d**/**16d** (80:20) as a pale yellow solid. The isomers were separated by flash column chromatography, giving 0.670 g (75%) of **15d** as pale yellow solid, mp 169–171 °C and 0.134 g (15%) of **16d** as a pale yellow solid, mp 170–171 °C. Data of **16d**: FT-IR (KBr) *ν*_max_ 2925, 2215, 1772, 1599, cm^−1; 1^H NMR (300 MHz, CDCl_3_) *δ* 1.66 (d, *J =* 6.9 Hz, 3H, H-16), 3.03 (ddd, *J* = 16.8, 4.8, 2.1 Hz, 1H, H-4β), 3.41 (ddd, *J* = 16.8, 11.4, 3.9 Hz, 1H, H-4α), 3.92–4.01 (m, 1H, H-7), 4.27 (dd, *J* = 11.4, 4.8 Hz, 1H, H-5), 7.48–7.57 (m, 5H, H-9, H-10, H-11), 7.95 (d, *J* = 8.9 Hz, 2H, H-13), 8.35 (d, *J* = 8.9 Hz, 2H, H-14). Signals attributed to minor isomer **16d**: 1.61 (d, *J =* 6.9 Hz, 3H, H-16), 3.09 (ddd, *J* = 16.8, 4.8, 2.1 Hz, 1H, H-4β), 3.29 (ddd, *J* = 16.8, 11.4, 3.9 Hz, 1H, H-4α), 3.81–3.84 (m, 1H, H-7), 4.24 (dd, *J* = 8.4, 4.8 Hz, 1H, H-5); ^13^C NMR (75.4 MHz, DMSO-*d*_6_) *δ* 13.9 (C-16), 24.6 (C-4), 38.2 (C-7), 46.0 (C-6), 46.5 (C-5), 112.8 (*C*N), 114.2 (*C*N), 121.2 (C-3a), 124.7 (C-14), 126.1 (C-9), 128.7 (C-11), 128.8 (C-10), 130.1 (C-13), 132.5 (C-7a), 131.1 (C-8), 134.4(C-12), 144.5 (C-8), 149.2 (C-15), 154.0 (C-2); HRMS (EI^+^) calcd for C_22_H_16_N_4_O_4_ 400.1171, found (M^+^) 400.1165.

Method B. Reaction of malononitrile **20b** (0.170 g, 0.0015 mol) and benzaldehyde **21d** (0.300 g, 0.0019 mol) with *exo*-2-oxazolidinone diene **2** (0.300 g, 0.0019 mol) gave a mixture of isomers **15d**/**16d** (70:30). The isomers were separated by flash column chromatography, giving 0.431 g (55%) of **15d** and 0.165 g (15%) of **16d**.

(5R*,7R*)-3-(4-Chlorophenyl)-6,6-dicyano-5-(4-methoxyphenyl)-7-methyl-4,5,6,7-tetrahydrobenzo [*d*]oxazol-2-one (**15e**). (5R*,7S*)-3-(4-Chlorophenyl)-6,6-dicyano-5-(4-methoxyphenyl)-7-methyl-4,5,6,7-tetrahydrobenzo[*d*]oxazol-2-one (**16e**). According to Method A, the reaction between **5b** (0.280 g, 0.0015 mol) and diene **3** (0.300 g, 0.0012 mol) gave a mixture of isomers **15e**/**16e** (75:25) as a pale yellow solid. The isomers were separated by flash column chromatography, giving 0.387 g (70%) of **15e** as pale yellow solid, mp 162–163 °C and 0.083 g (15%) of **16e** as a pale yellow solid, mp 162–163 °C. Data of **15e**: FT-IR (KBr) *ν*_max_ 2935, 2250, 1760, 1496 cm^−1; 1^H NMR (300 MHz, CDCl_3_) *δ* 1.67 (d, *J =* 6.6 Hz, 3H, H-16), 2.69 (ddd, *J* = 17.4, 5.1, 2.1 Hz, 1H, H-4β), 3.09 (ddd, *J* = 17.4, 11.7, 3.9 Hz, 1H, H-4α), 3.45 (dd, *J* = 10.8, 5.1 Hz,1H, H-5), 3.47–3.82 (m, 1H, H-7), 3.82 (s, 3H, OC*H*_3_), 6.96 (d, *J =* 9.0 Hz, 2H, H-14), 7.33 (d, *J* = 9.0 Hz, 4H, H-13, H-9), 7.40 (d, *J* = 9.0 Hz, 2H, H-9), 7.46 (d, *J* = 9.0 Hz, 2H, H-10); ^13^C NMR (75.4 MHz, CDCl_3_) *δ* 14.1 (C-16), 25.1 (C-4), 38.7 (C-7), 46.3 (C-6), 47.1 (C-5), 55.6 (O*C*H_3_), 112.1 (*C*N), 113.6 (*C*N), 114.9 (C-14), 120.4 (C-3a), 126.7 (C-9), 127.3 (C-12), 129.5 (C-13), 130.2 (C-10), 131.7 (C-8), 132.5 (C-7a), 134.6 (C-11), 153.7 (C-2), 160.5 (C-15). Data of **16e**. Yield: 57% (pale yellow solid, mp 163–165 °C); FT-IR (KBr) *ν*_max_ 2935, 2235, 1760, 1609 cm^−1; 1^H NMR (300 MHz, CDCl_3_) *δ* 1.63 (d, *J =* 6.9 Hz, 3H, H-16), 2.83–2.98 (m, 2H, H-4), 3.37–3.39 (m, 1H, H-7), 3.54–3.57 (m, 1H, H-5), 3.82 (s, 3H, OC*H*_3_), 6.94 (d, *J* = 8.7 Hz, 2H, H-14), 7.31 (d, *J* = 8.7 Hz, 4H, H-13, H-9), 7.45 (d, *J* = 9.0 Hz, 2H, H-10); ^13^C NMR (75.4 MHz, CDCl_3_) *δ* 15.1 (C-16), 24.5 (C-4), 35.1 (C-7), 42.3 (C-6), 43.3 (C-5), 55.3 (O*C*H_3_), 112.8 (*C*N), 113.6 (*C*N), 114.6 (C-14), 119.6 (C-3a), 126.4 (C-12), 126.5 (C-9), 129.4 (C-13), 129.9 (C-10), 131.3 (C-8), 133.0 (C-7a), 134.3 (C-11), 153.4 (C-2), 160.4 (C-15); HRMS (EI^+^) calcd for C_23_H_18_N_3_O_3_Cl 419. 1036, found (M^+^) 419. 1036.

(5R*,7R*)-6,6-Diethoxycarbonyl-7-methyl-3,5-diphenyl-4,5,6,7-tetrahydrobenzo[*d*]oxazol-2-one (**17a**). According to Method A, the reaction between **6a** (0.44 g, 0.0017 mol) and diene **2** (0.300 g, 0.0015 mol) produced only the isomer **17a** (0.154 g, 23%) as pale yellow solid: mp 132–133 °C. FT-IR: *ν*_max_ 2980, 1770, 1727, 1503 cm^−1; 1^H NMR (300 MHz, CDCl_3_) *δ* 1.18 (t, *J* = 7.2 Hz, 3H, OCH_2_C*H*_3_), 1.26 (t, *J* = 7.2 Hz, 3H, OCH_2_C*H*_3_), 1.38 (d, *J* = 6.9 Hz, 1H, C-16), 2.42 (ddd, *J* = 17.4, 4.8, 2.1 Hz, H-4α), 3.49–3.59 (m, 2H, H-7, H-4β), 3.82 (dd, *J* = 6.6, 2.1 Hz, 1H, H-5), 4.04–4.27 (m, 4H, OC*H*_2_CH_3_), 7.23–7.45 (m, 10H, H-Ar); ^13^C NMR (75.4 MHz, CDCl_3_) *δ* 13.6 (C-16), 13.8 (OCH_2_*C*H_3_), 14.0 (OCH_2_*C*H_3_), 26.3 (C-7), 29.5 (C-4), 43.3 (C-5), 61.1 (C-6), 61.3 (O*C*H_2_CH_3_), 61.4 (O*C*H_2_CH_3_*)*, 119.5 (C-3a), 125.2 (C-9), 127.7 (C-11), 128.0 (C-12), 128.4 (C-14), 128.6 (C-13), 129.3 (C-10), 133.6 (C-8), 135.1 (C-7a), 154.7 (C-2), 168.5 (*C*O_2_CH_2_CH_3_) 169.3 (*C*O_2_CH_2_CH_3_). HRMS (EI_+_) calcd for C_26_H_27_NO_6_ 449.1838, found (M^+^) 449.1838.

(5R*,7R*)-6,6-Diethoxycarbonyl-5-(4-methoxyphenyl)-7-methyl-3-phenyl-4,5,6,7-tetrahydrobenzo [*d*]oxazol-2-one (**17b**). According to Method A, the reaction between **6b** (0.58 g, 0.0028mol) and diene **2** (0.350 g, 0.0017 mol) produced only the isomer **17b** (0.269 g, 32%) as a pale yellow solid: mp 143–145 °C. FT-IR: *ν*_max_ 2980, 1768, 1727, 1504 cm^−1; 1^H NMR (300 MHz, CDCl_3_) *δ* 1.20 (t, *J* = 7.2 Hz, 3H, OCH_2_C*H*_3_), 1.26 (t, *J* = 7.2 Hz, 3H, OCH_2_C*H*_3_), 1.37 (d, *J* = 6.6 Hz, 3H, H-16), 2.38 (ddd, *J* = 17.1, 4.8, 2.4 Hz, 1H, H-4α), 3.47–3.56 (m, 2H, H-4β, H-7), 3.75 (s, 3H, OC*H*_3_), 3.78 (dd, *J* = 6.9, 2.4 Hz, 1H, H-5), 4.10–4.26 (m, 4H, OC*H*_2_CH_3_), 6.77 (d, *J* = 8.7 Hz, 2H, H-14), 7.04 (d, *J* = 8.7 Hz, 2H, H-15), 7.28–7.45 (m, 5H, H-Ar); ^13^C NMR (75.4 MHz, CDCl_3_) *δ* 13.6 (C-16), 13.8 (OCH_2_*C*H_3_), 13.9 (OCH_2_*C*H_3_), 26.5 (C-4), 29.5 (C-7), 42.5 (C-5), 55.1 (O*C*H_3_), 61.2 (C-6), 61.2 (O*C*H_2_CH_3_), 61.3 (O*C*H_2_CH_3_), 113.8 (C-14), 119.5 (C-3a), 125.2 (C-9), 127.7 (C-11), 129.1 (C-10), 129.3 (C-13), 132.5 (C-12), 133.7 (C-7a), 135.2 (C-8), 154.7 (C-2), 158.9 (C-15), 168.6 (*C*O_2_CH_2_CH_3_), 169.3 (*C*O_2_CH_2_CH_3_). HRMS (EI^+^) calcd for C_26_H_27_NO_6_ 479.1944, found (M^+^) 479.1898.

(5R*,7R*)-5-(4-Chlorophenyl)-6,6-diethoxycarbonyl-7-methyl-3-phenyl-4,5,6,7-tetrahydrobenzo[*d*] oxazol-2-one (**17c**). According to Method A, the reaction between **4a** (144 mg, 2.0 mmol) and diene **2** (162 mg, 1.0 mmol) produced only the isomer **17c** (0.121 g, 25%) as a pale yellow solid: mp 166–168 °C. FT-IR: *ν*_max_ 2980, 1770, 1729, 1597 cm^−1; 1^H NMR (300 MHz, CDCl_3_) *δ* 1.20 (t, *J* = 7.2 Hz, 3H, OCH_2_C*H*_3_), 1.25 (t, *J* = 7.2 Hz, 3H, OCH_2_C*H*_3_), 1.38 (d, *J* = 6.6 Hz, 1H, H-16), 2.40 (dd, *J* = 17.4, 2.4 Hz, 1H, H-4α), 3.44–3.52 (m, 2H, H-4β, H-7), 3.80 (dd, *J* = 6.9, 2.4 Hz, 1H, H-5), 4.08–4.26 (m, 4H, OC*H*_2_CH_3_), 7.09 (d, *J* = 8.4 Hz, 2H, H-14), 7.22 (d, *J* = 8.4 Hz, 1H, H-13), 7.31–7.45 (m, 5H, H-Ar); ^13^C NMR (75.4 MHz, CDCl_3_) *δ* 13.7 (C-16), 13.8 (OCH_2_*C*H_3_), 13.8 (OCH_2_*C*H_3_), 29.6 (C-7), 31.4 (C-4), 42.5 (C-5), 60.8 (C-6), 61.4 (O*C*H_2_CH_3_), 61.5 (O*C*H_2_CH_3_), 119.2 (C-3a), 125.0 (C-9), 127.7 (C-11), 128.6 (C-10), 129.3 (C-13), 129.5 (C-14), 130.4 (C-7a), 133.5 (C-15), 135.0 (C-8), 138.8 (C-12), 154.5 (C-2), 168.3 (*C*O_2_CH_2_CH_3_), 168.9 (*C*O_2_CH_2_CH_3_). HRMS (EI^+^) calcd for C_26_H_26_NO_6_Cl 483.1448, found (M^+^) 483.1445.

Dimerization of Diene **2**. According to Method B, the reaction between **20a** (0.250 g, 0.0023 mol), **21c** (0.375 g, 0.0023 mol) and diene **2** (0.474 g, 0.0023 mol), produced two products: **4a** (0.375 g, 64%) and dimer **19** (0.0.340 g, 36%) as a pale yellow crystal (acetone/hexane). **19**: mp 196–198 °C; IR (KBr) *ν*_max_ 2924, 17850, 1762, 1706, 1501, 1245 cm^−1; 1^H NMR (500 MHz, CDCl_3_) *δ* 1.38 (d, *J* = 6.9 Hz, 3H, H-8), 1.81 (d, 1H, *J* = 6.9 Hz, 3H, H-14), 2.00–2.10 (m, 1H, H-4β), 2.14–2.20 (m, 1H, H-5β), 2.34–2.41 (m, 1H, H-4α), 2.62–2.75 (m, 1H, H-5α), 2.95–3.08 (m, 1H, H-7α), 4.70 (q, *J* = 6.9 Hz, 1H, H-13), 7.22–7.48 (m, 10H, H-Ar); ^13^C NMR (125 MHz, CDCl_3_) *δ* 10.2 (C-14), 10.3 (C-8), 17.3 (C-4), 32.2 (C-5), 34.0 (C-7), 67.5 (C-6), 99.5 (C-13), 118.9 (C-3a), 124.9 (C-16), 127.9 (C-20), 128.0 (C-22), 129.5 (C-18), 129.8 (C-21), 130.0 (C-17), 133.0 (C-15), 133.4 (C-19), 134.9 (C-7a), 145.6 (C-12), 154.2 (C-2), 154.3 (C-10); HRMS (EI^+^) calcd for C_24_H_22_N_2_O_4_ 402.1579, found (M^+^) 402.1578.

### 3.4. X-ray Structure Study of **7e**, **13a** and **19**

Single crystals were obtained by slow evaporation of concentrated solutions of **7e** (*n*-hexane/AcOEt, pale yellow solid), **13a** (*n*-hexane/CH_2_Cl_2_, white solid), and **19** (*n*-hexane/AcOEt, pale yellow). These were mounted on glass fibers. Crystallographic measurements were performed on a Siemens P-4 difractometer using Mo KR radiation (graphite crystal monochromator, λ = 71073 Ǻ) at room temperature. Three standard reflections, which were monitored periodically, showed no change during data collection. Unit cell parameters were obtained from least-squares refinement of 26 reflections in the range 2° < 2θ < 20°. Intensities were corrected for Lorentz and polarization effects. No absorption correction was applied. Anisotropic temperature factors were introduced for all non-hydrogen atoms. Hydrogen atoms were placed in idealized positions and their atomic coordinates refined. Structures were solved using the SHELXTL [48], SHELX97 [49], or SIR92 [50] programs as implemented in the WinGX suite [51] and refined using SHELXTL or SHELX97 within WinGX, on a personal computer. In all cases ORTEP and packing diagrams were made with PLATON and ORTEP-3 [52–53].

### 3.5. Theoretical Calculations

The *ab initio* HF/6-31G(d,p) and DFT B3LYP/6-31G(d,p) calculations were carried out using Gaussian 03 [43] (PC-Linux). Geometries were calculated at the B3LYP/6-31G(d,p) level, and these were employed as the starting point for optimizations at the same level. The energies and coefficients of the frontier molecular orbitals were obtained at single point from the HF/6-31G(d,p) level.

## 4. Conclusions

In summary, we have successfully developed a new, efficient, regio- and stereoselective Diels-Alder reaction between a series of Knoevenagel adducts as dienophiles and *exo*-2-oxazolidinone dienes. This process was also satisfactorily carried out via the one-pot, three-component reaction between the corresponding benzaldehydes, the active methylene compounds and the *exo*-2-oxazolidinone dienes. Both methodologies were promoted by infrared irradiation, as an eco-friendly energy source for the first time, under solvent-free conditions. In all the cases, the *para*-*endo* cycloadducts were favored, with respect to the *meta* or *para*-*exo* adducts. An additional advantage of these methods is the fact that the use of a solvent and the activation of the reactions by an acid catalyst were unnecessary, finding environmentally friendly protocols.

## Supporting Information



## Figures and Tables

**Table 1:** Diels-Alder reactions of diene **1** with dienophiles **4**–**6** a.

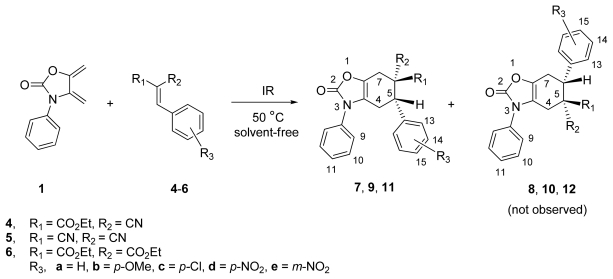						
Entry	Dienophile	R_1_	R_2_	R_3_	Reaction Time (h)	Product e (%)
1	**4a**	CO_2_Et	CN	H	3.5	**7a** (73)
2 b	**4a**	CO_2_Et	CN	H	20	**7a** (30)
3 c	**4a**	CO_2_Et	CN	H	24	**7a** (20)
4 d	**4a**	CO_2_Et	CN	H	24	**7a** (20)
5	**4b**	CO_2_Et	CN	*p*-OMe	4.0	**7b** (50)
6	**4c**	CO_2_Et	CN	*p*-Cl	3.5	**7c** (60)
7	**4d**	CO_2_Et	CN	*p*-NO_2_	3.0	**7d** (80)
8	**4e**	CO_2_Et	CN	*m*-NO_2_	3.5	**7e** (55)
9	**5a**	CN	CN	H	4.0	**9a** (80)
10	**5b**	CN	CN	*p*-OMe	4.5	**9b** (55)
11	**5c**	CN	CN	*p*-Cl	3.0	**9c** (75)
12	**5d**	CN	CN	*p*-NO_2_	3.0	**9d** (85)
13	**6a**	CO_2_Et	CO_2_Et	H	5.0	**11a** (35)
14	**6b**	CO_2_Et	CO_2_Et	*p*-OMe	6.0	**11b** (25)
15	**6c**	CO_2_Et	CO_2_Et	*p*-Cl	5.0	**11c** (30)

a All entries were carried out under IR irradiation at 50 °C and solvent-free conditions, except entries 2–4;

b Under thermal (50 °C) and solvent-free conditions;

c Under thermal conditions (50 °C) in benzene as the solvent;

d Under thermal conditions (50 °C) in THF as the solvent;

e After column chromatography.

**Table 2:** Diels-Alder reactions of dienes **2** and **3** with dienophiles **4**–**6** a.

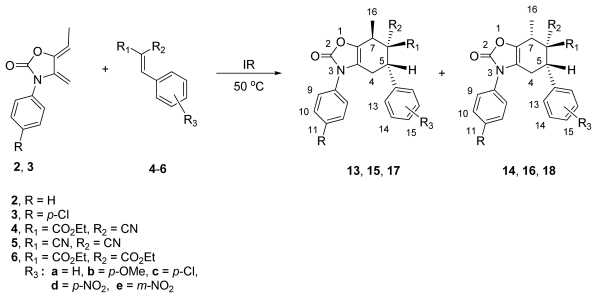									
Entry	Diene	Dienophile	R	R_1_	R_2_	R_3_	Reaction Time (h)	Products (*endo*/*exo*) b	Yield c (%)
1	**2**	**4a**	H	CO_2_Et	CN	H	4.0	**13a/14a** (80:20)	56/32
2	**2**	**4b**	H	CO_2_Et	CN	*p*-OMe	5.0	**13b/14b** (75:25)	65 d
3	**2**	**4c**	H	CO_2_Et	CN	*p*-Cl	4.5	**13c/14c** (68:32)	60 d
4	**2**	**4d**	H	CO_2_Et	CN	*p*-NO_2_	4.0	**13d/14d** (75:25)	64 d
5	**3**	**4e**	*p*-Cl	CO_2_Et	CN	*m*-NO_2_	4.5	**13e/14e** (75:25)	70 d
6	**2**	**5a**	H	CN	CN	H	3.0	**15a/16a** (80:20)	70 d
7	**2**	**5b**	H	CN	CN	*p*-OMe	4.0	**15b/16b** (82:18)	55 d
8	**2**	**5c**	H	CN	CN	*p*-Cl	5.0	**15c/16c** (90:10)	75 d
9	**2**	**5d**	H	CN	CN	*p*-NO_2_	2.0	**15d/16d** (80:20)	75/15
10	**3**	**5b**	*p*-Cl	CN	CN	*p*-OMe	3.0	**15e/16e** (75:25)	70/15
11	**2**	**6a**	H	CO_2_Et	CO_2_Et	H	6.0	**17a/18a** (100:0)	23 d
12	**2**	**6b**	H	CO_2_Et	CO_2_Et	*p*-OMe	5.0	**17b/18b** (100:0)	32 d
13	**2**	**6c**	H	CO_2_Et	CO_2_Et	*p*-Cl	4.0	**17c/18c** (100:0)	25 d

a All entries under IR irradiation at 50 °C and solvent-free conditions;

b Determined by ^1^H NMR of the crude reaction mixtures, corresponding to the mixture of stereoisomers;

c Yields of the products after column chromatography;

d Yield of the major product.

**Table 3:** Domino Knoevenagel condensation/Diels-Alder cycloaddition between diene **2**, methylene active compounds **20a**–**c** and benzaldehydes **21a**–**d** a.

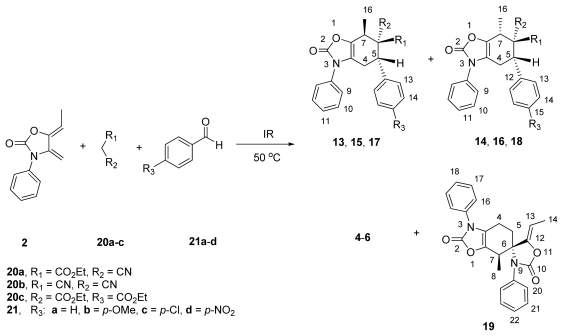						
Entry	Methylene Active	Benzaldehyde	Reaction Time (min)	By-Products (%) b	Adducts (*endo*/*exo*) c	Yield d (%)
1	**20a**	**21a**	35	**4a**/**19** (40:5)	**13a/14a** (65:35)	43/12
2	**20a**	**21b**	50	**4b**/**19** (52:8)	**13b/14b** (75:25)	40 e
3	**20a**	**21c**	30	**4c**/**19** (35:5)	**13c/14c** (68:32)	60 e
4	**20a**	**21d**	40	**4d**/**19** (32:4)	**13d/14d** (75:25)	64 e
5	**20b**	**21a**	30	**5a**/**19** (40:5)	**15a/16a** (70:30)	55 e
6	**20b**	**21b**	40	**5b**/**19** (40:10)	**15b/16b** (85:15)	50 e
7	**20b**	**21c**	30	**5c**/**19** (20:5)	**15c/16c** (80:20)	65/10
8	**20b**	**21d**	35	**5d**/**19** (25:5)	**15d/16d** (70:30)	55/15
9	**20c**	**21a**	150	**6a**/**19** (64:36)	--	--
10	**20c**	**21b**	210	**6b**/**19** (70:30)	--	--
11	**20c**	**21c**	240	**6c**/**19** (60:40)	--	--
12	**20c**	**21d**	240	**6d**/**19** (65:35)	--	--

a An equimolar mixture of **2**, **20** and **21** was irradiated with IR at 50 °C, under solvent-free conditions;

b After column chromatography;

c Determined by ^1^H NMR of the crude reaction;

d Yields of the adducts after column chromatography;

e Yield of the major adduct.

**Table 4:** *Ab initio* 6-31G** calculations of energies (eV) and coefficients (C_i_) of the frontier molecular orbitals for dienes **1**–**3** and dienophiles **4**–**6** a.

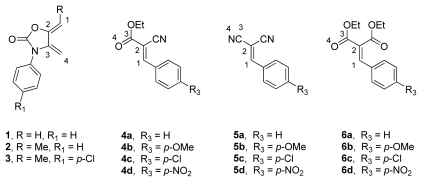												
HOMO							LUMO					

Compd b	*E* (eV)	*C*_1_	*C*_2_	*C*_3_	*C*_4_	Δ*C*_i_^c^	*E* (eV)	*C*_1_	*C*_2_	*C*_3_	*C*_4_	Δ*C*_i_^c^)
**1**^d^	−8.8051	0.246	0.164	−0.209	−0.326	0.080	2.9065	0.263	−0.245	−0.245	0.258	−0.005
**2**^d^	−8.5610	−0.257	−0.199	0.198	0.320	0.063	3.1035	0.274	−0.222	−0.245	0.248	−0.026
**3**	−8.6408	−0.277	−0.220	0.199	0.324	0.047	2.7244	0.288	−0.232	−0.247	0.258	−0.030
**4a**	−8.9382	0.122	0.276	0.015	−0.114	−0.154	1.0104	0.296	−0.210	−0.131	0.113	0.086
**4b**	−8.4299	−0.084	−0.260	−0.020	0.105	−0.176	1.2204	0.306	−0.203	−0.134	0.112	0.103
**4c**	−9.0541	0.116	0.265	0.014	−0.109	−0.149	0.7532	0.288	−0.211	−0.126	0.110	0.077
**4d**	−9.7679	0.160	0.284	0.008	−0.121	−0.124	−0.1056	0.219	−0.196	−0.096	0.092	0.023
**5a**	−9.2234	0.126	0.275	−0.058		−0.149	0.5331	0.305	−0.227	−0.071		0.078
**5b**	−8.6859	−0.087	−0.261	0.044		−0.174	0.7611	0.315	−0.219	−0.073		0.096
**5c**	−9.3227	0.118	0.263	−0.055		−0.145	0.2759	0.298	−0.227	−0.068		0.071
**5d**	−10.0495	0.163	0.280	−0.073		−0.117	−0.5323	0.238	−0.214	−0.050		0.024
**5c**	−9.3227	0.118	0.263	−0.055		−0.145	0.2759	0.298	−0.227	−0.068		0.071
**6a**	−8.6751	0.112	0.257	0.013	−0.100	−0.145	1.7804	0.256	−0.215	−0.153	0.126	0.041
**6b**	−8.1608	0.043	0.242	0.017	−0.091	−0.199	1.9179	0.272	−0.210	−0.151	0.122	0.062
**6c**	−8.8008	0.106	0.246	0.012	−0.094	−0.140	1.5032	0.248	−0.215	−0.142	0.119	0.033
**6d**	−9.5564	0.148	0.271	0.005	−0.108	−0.123	0.5236	−0.164	0.183	0.095	−0.088	−0.019

a These are the values of the *p_z_* coefficients, the relative *p_z_'* contributions and their **Δ***C_i_* are analogous;

b The most stable planar (aryl ring-double bond-numbered *trans* carbonyl group) *s*-*cis* (acrylate moiety) conformation for olefins **4** and **6**, and planar (aryl ring-double bond-cyano group) conformation for olefins **5**, as shown in the structures at the head of the table;

c Carbon 4-carbon 1 for the dienes; carbon 1-carbon 2 for the dienophile;

d Reference 17.

**Table 5:** Energy gaps (eV) of the frontier molecular orbitals for dienes **1**–**3** and dienophiles **4a**–**6a**.

	4a a			5a a			6a a		
						
Diene	HOMO-LUMO	LUMO-HOMO	Diff.	HOMO-LUMO	LUMO-HOMO	Diff.	HOMO-LUMO	LUMO-HOMO	Diff.
**1**	9.8155	11.8447	2.0292	9.3382	12.1299	2.7917	10.5855	11.5816	0.9961
**2**	9.5714	12.0417	2.4703	9.0941	12.3269	3.2328	10.3414	11.7786	1.4372
**3**	9.6512	11.6626	2.0114	9.1739	11.9478	2.7739	10.4212	11.3995	0.9783

a HOMO_diene_-LUMO_dienophile_ and LUMO_diene_-HOMO_dienophile_.
