# Supplementary material for: Highly Regio- and Stereoselective Diels-Alder Cycloadditions via Two-Step and Multicomponent Reactions Promoted by Infrared Irradiation under Solvent-Free Conditions
Source: Int J Mol Sci. 2012 Feb 24;13(3):2590–617. doi: 10.3390/ijms13032590 (PMC3317676; doi:10.3390/ijms13032590)
Supplement: Supplementary file 1 [file ijms-13-02590-s001.pdf]

Supporting Information

Highly Regio-and Stereoselective Diels-Alder Cycloadditions via Two-step and Multicomponent Reactions Promoted by Infrared Irradiation Under Solvent-free Conditions.

María Inés Flores-Conde,[1] Leonor Reyes,[1] Rafael Herrera,[2] Hulme Rios,[3] Miguel A. Vázquez,[4] * René Miranda,[3] Joaquín Tamariz,[1] Francisco Delgado.[1]*

[1] Departamento de Química Orgánica, Escuela Nacional de Ciencias Biologicas-IPN, Prol. Carpio y Plan de Ayala, 11340 Mexico, D. F., Mexico. [2] Instituto de Investigaciones Quimicobiologicas, Universidad Michoacana de San Nicolas de Hidalgo, Edif. B-1, Ciudad Universitaria, Francisco J. Mújica S/N, 58066 Morelia, Mich., Mexico. [3] Departamento de Ciencias Quımicas, Facultad de Estudios Superiores Cuautitlan-UNAM, Campo 1, Avenida 1ro. de Mayo s/n, Cuautitlan Izcalli, Estado de Mexico 54740, Mexico. [4] Facultad de Quimica, Universidad de Guanajuato, Noria Alta, s/n, Guanajuato Gto. 36050, Mexico.

fdelgado@woodward.encb.ipn.mx

Contents **page**

IR, HRMS, ^{1}H, ^{13}C NMR and other NMR data	S1-S25, S29-S67, S71-S172.
Figures (X-ray)	S26-S28, S68-S70, S173-S175.
Theoretical Calculations	S176-S209.

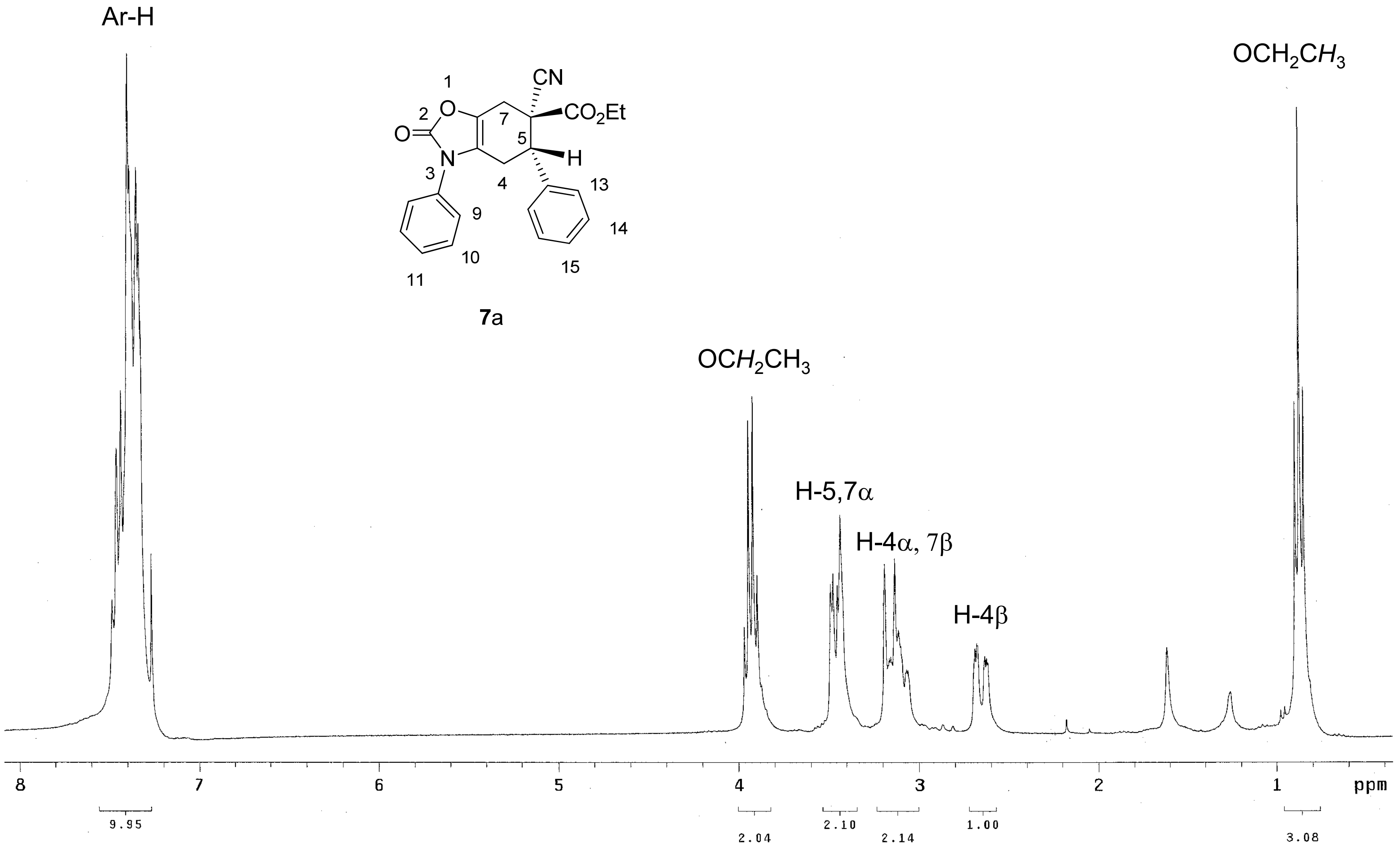

^{1}H NMR ($CDCl_3$) **7a**

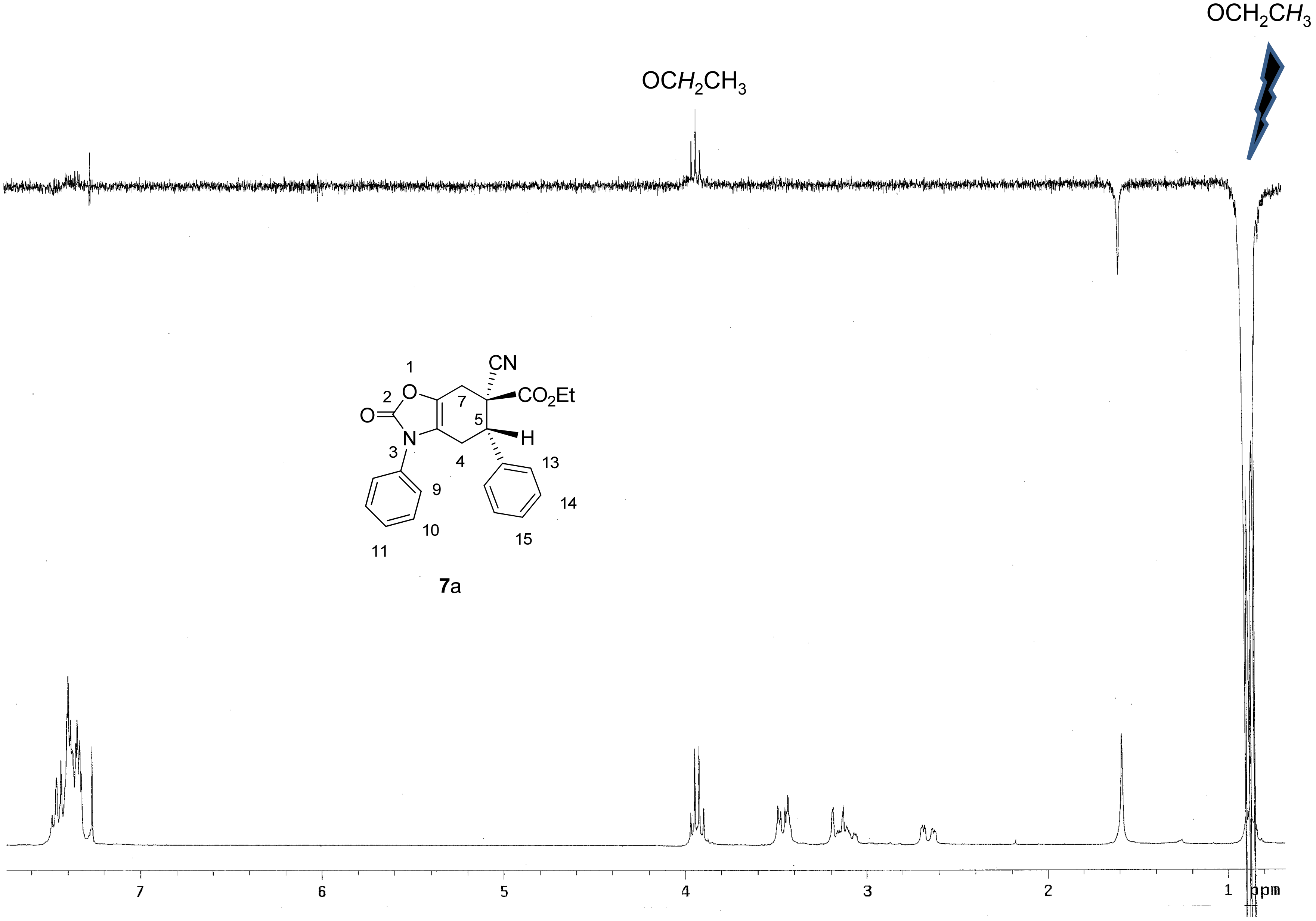

NOE experiment ($CDCl_3$) **7a**

S3

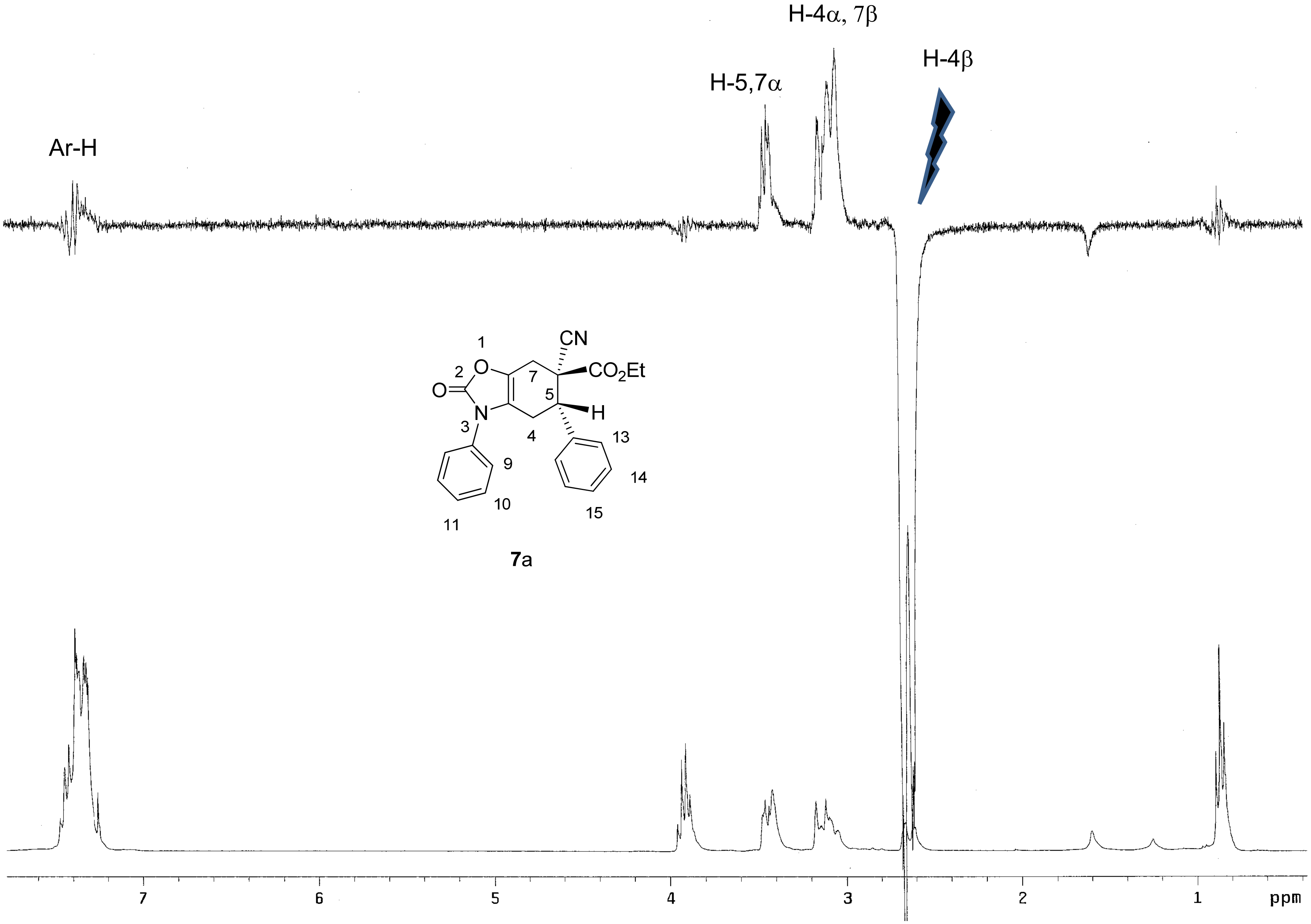

NOE experiment ($CDCl_3$) **7a**

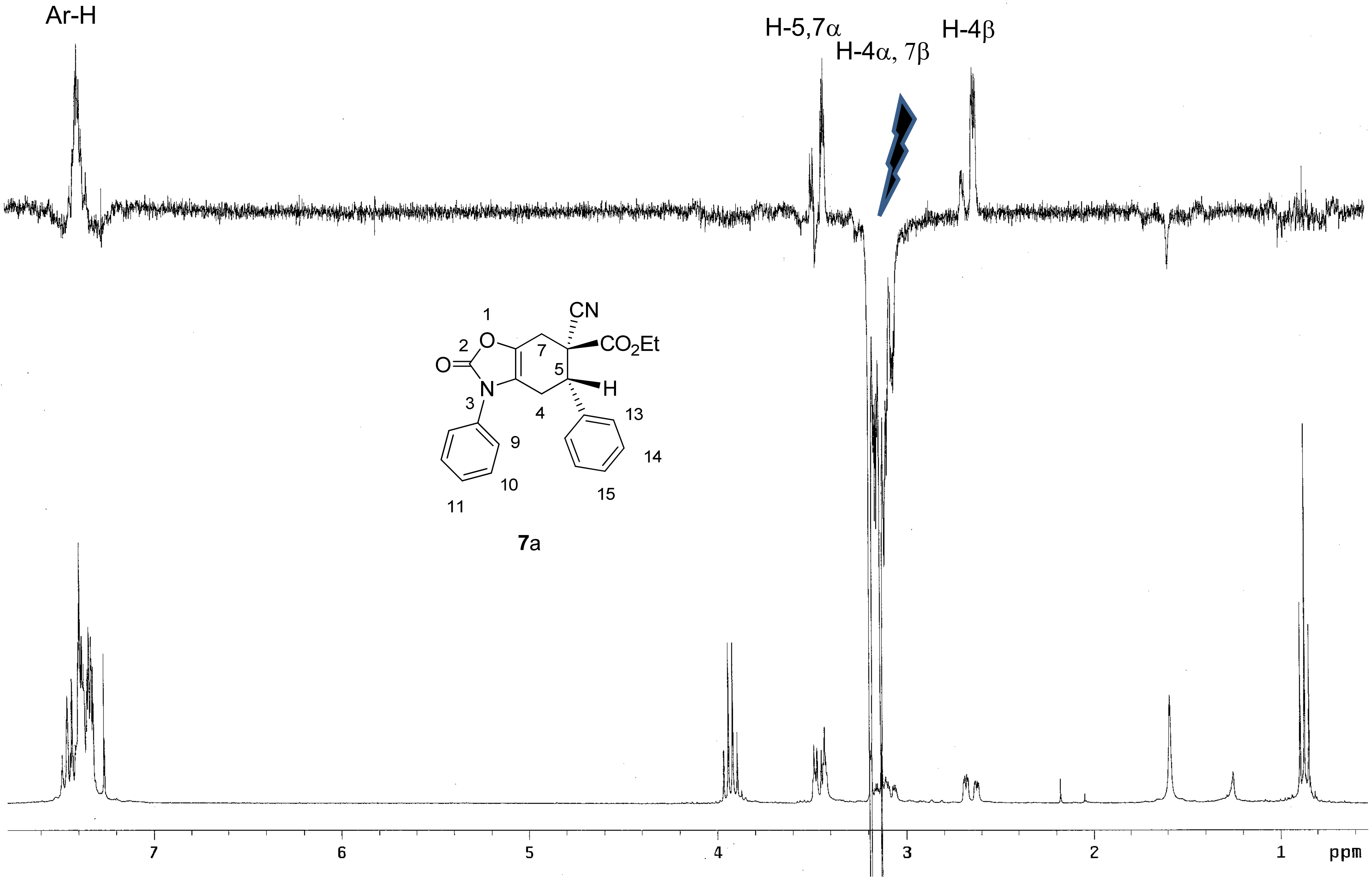

NOE experiment ($CDCl_3$) **7a**

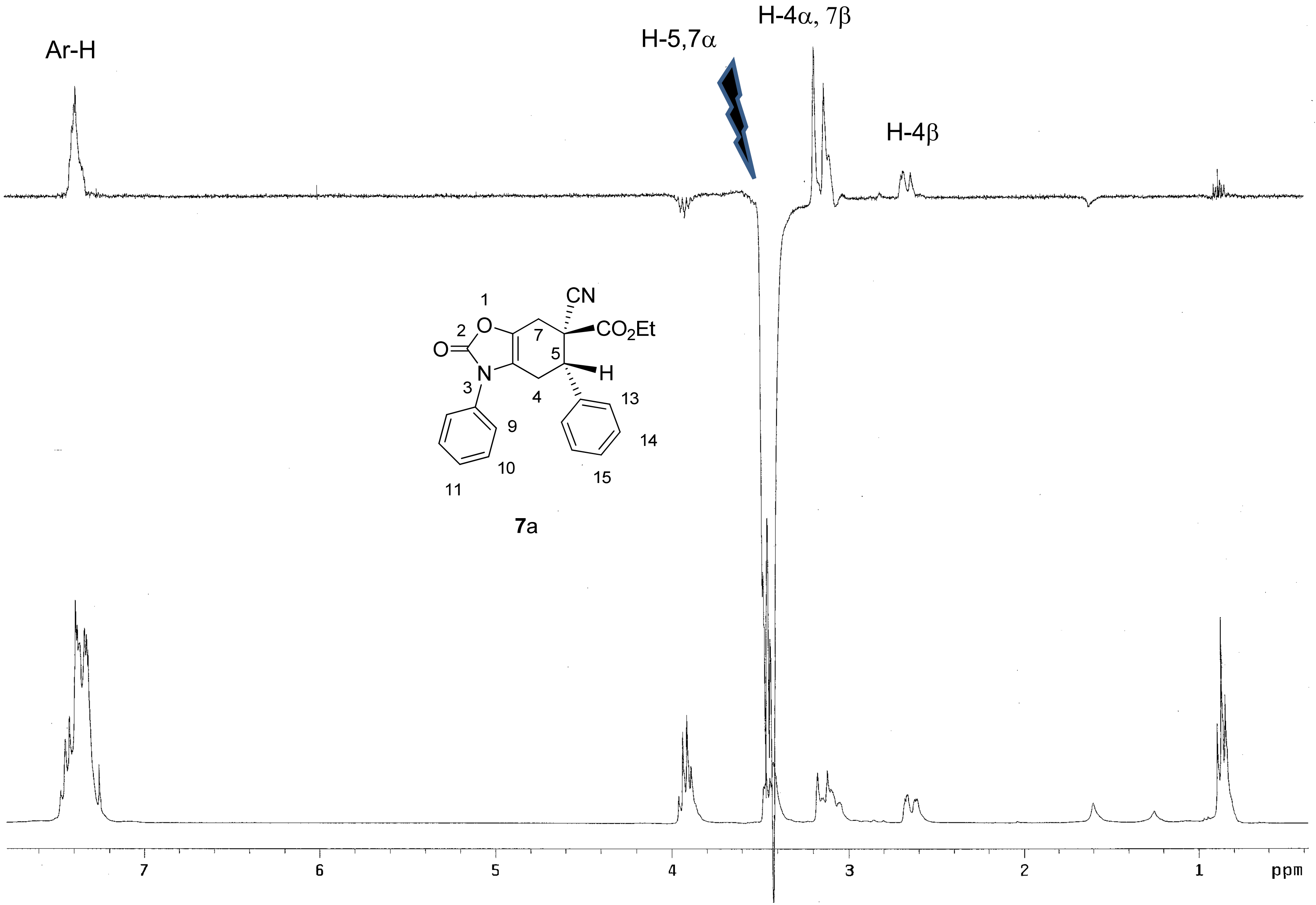

NOE experiment ($CDCl_3$) **7a**

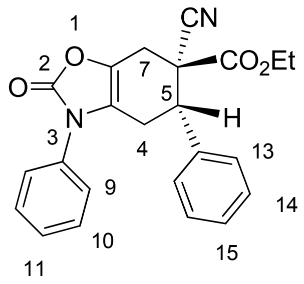

7a

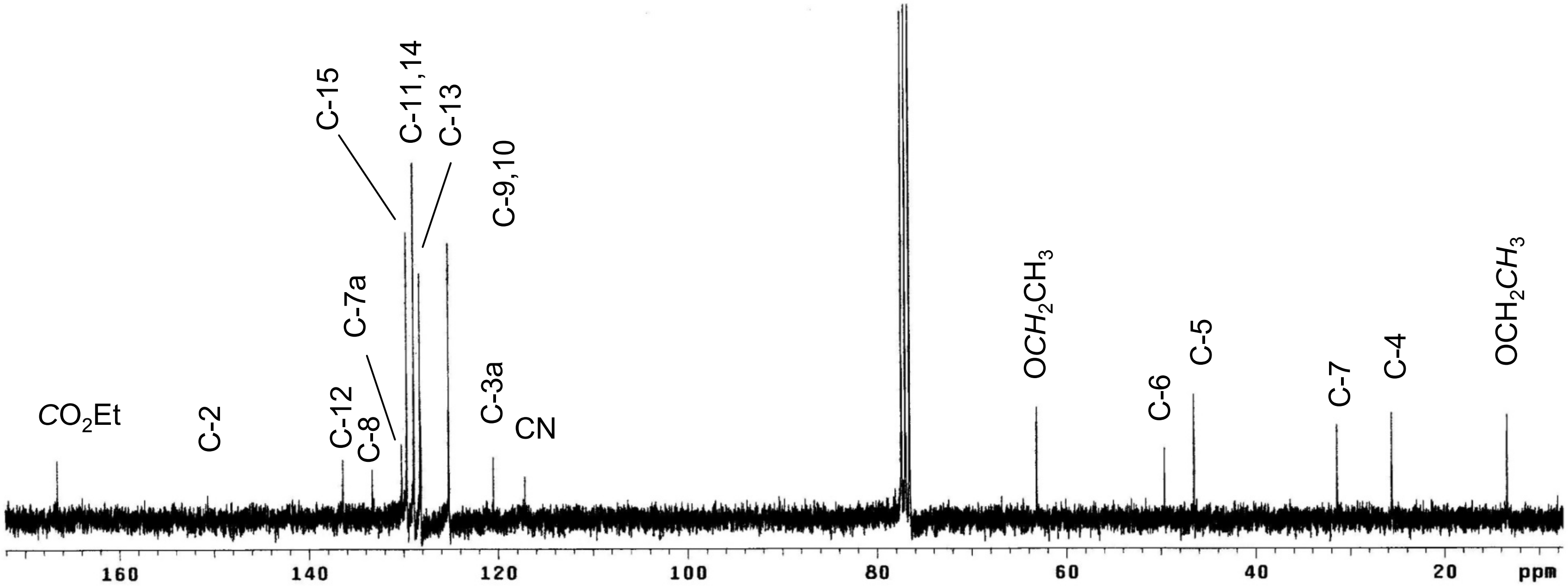

^{13}C NMR (CDCl$_3$) **7a**

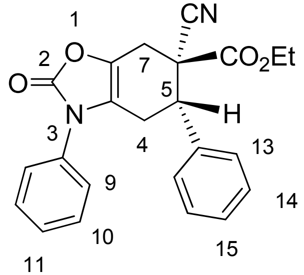

7a

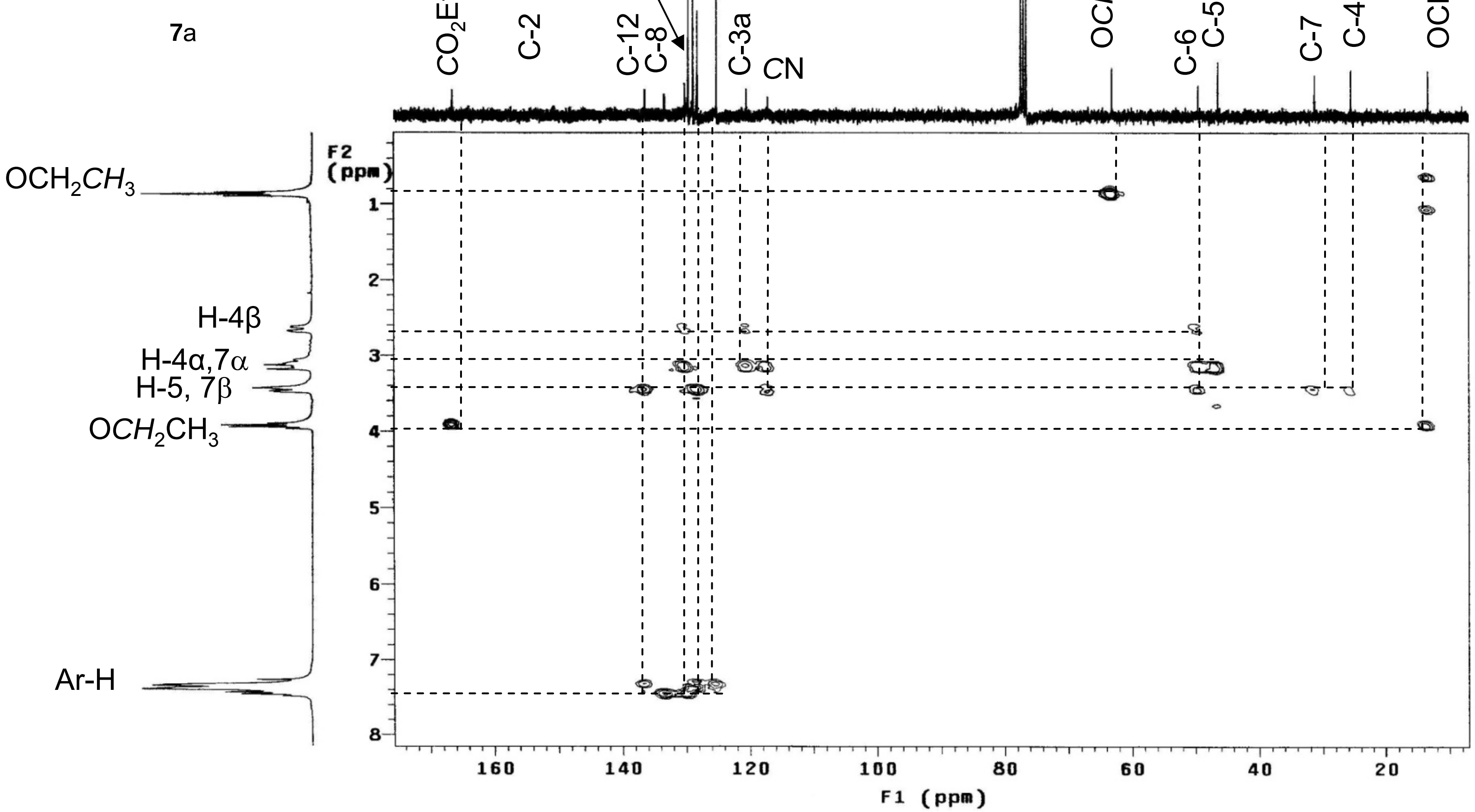

HMBC experiment ($CDCl_3$) **7a**

File: Ines 388 ID IE MI1Date Run: 02-01-2008 (Time Run: 13:05:29)
Sample: MI1
Instrument: JEOL GCmate
Inlet: Direct Probe **Ionization mode: EI+**

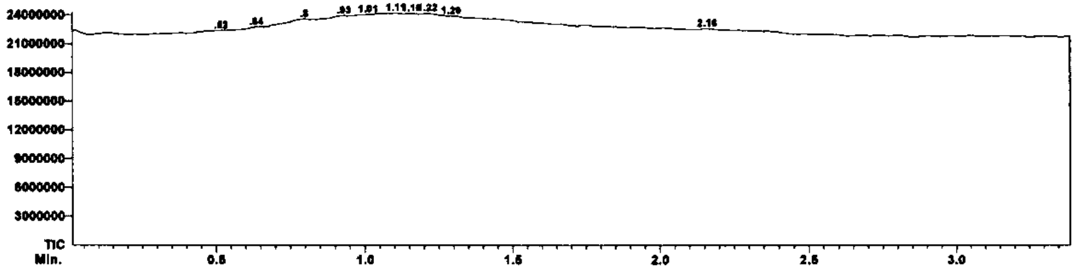

Scan: 80-147 **R.T.: 1.52**
Base: m/z 388; 1.4%FS TIC: 942019 **#Ions: 2850**

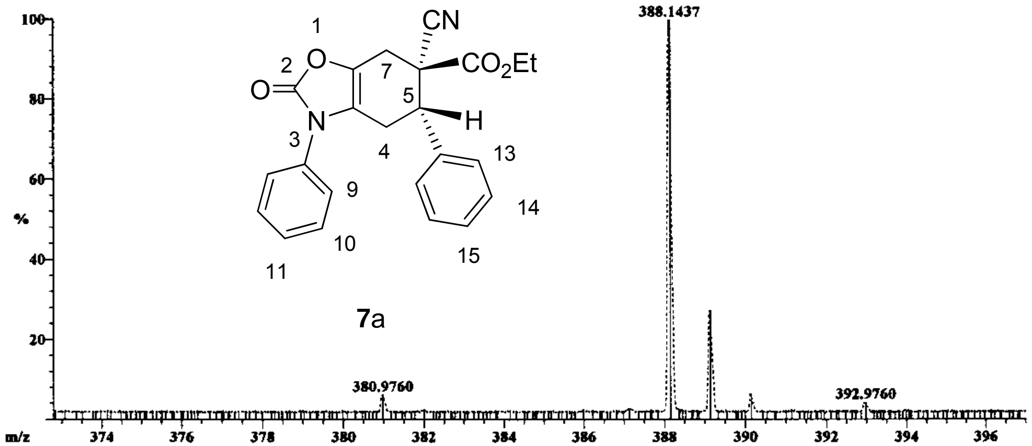

Selected Isotopes : $N_{0-3}O_{0-5}H_{0-25}C_{0-25}$ **Error Limit : 10 mmu**

Measured Mass	***% Base***	***Formula***	***Calculated Mass***	***Error***
388.14369	100.0%	$C_{23}H_{20}N_2O_4$	388.14231	1.4

HRMS **7a**

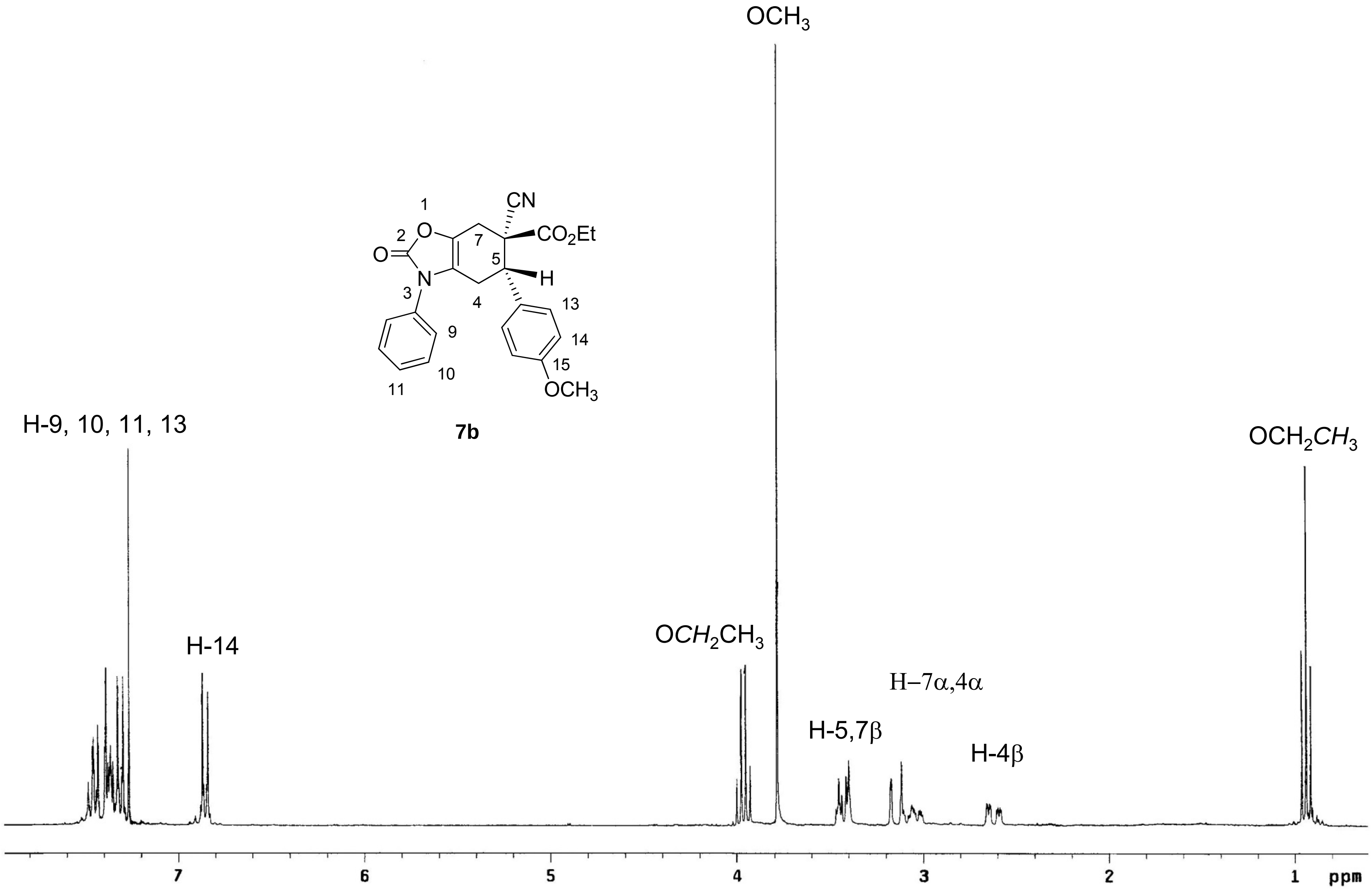

^{1}H NMR (CDCl$_3$) **7b**

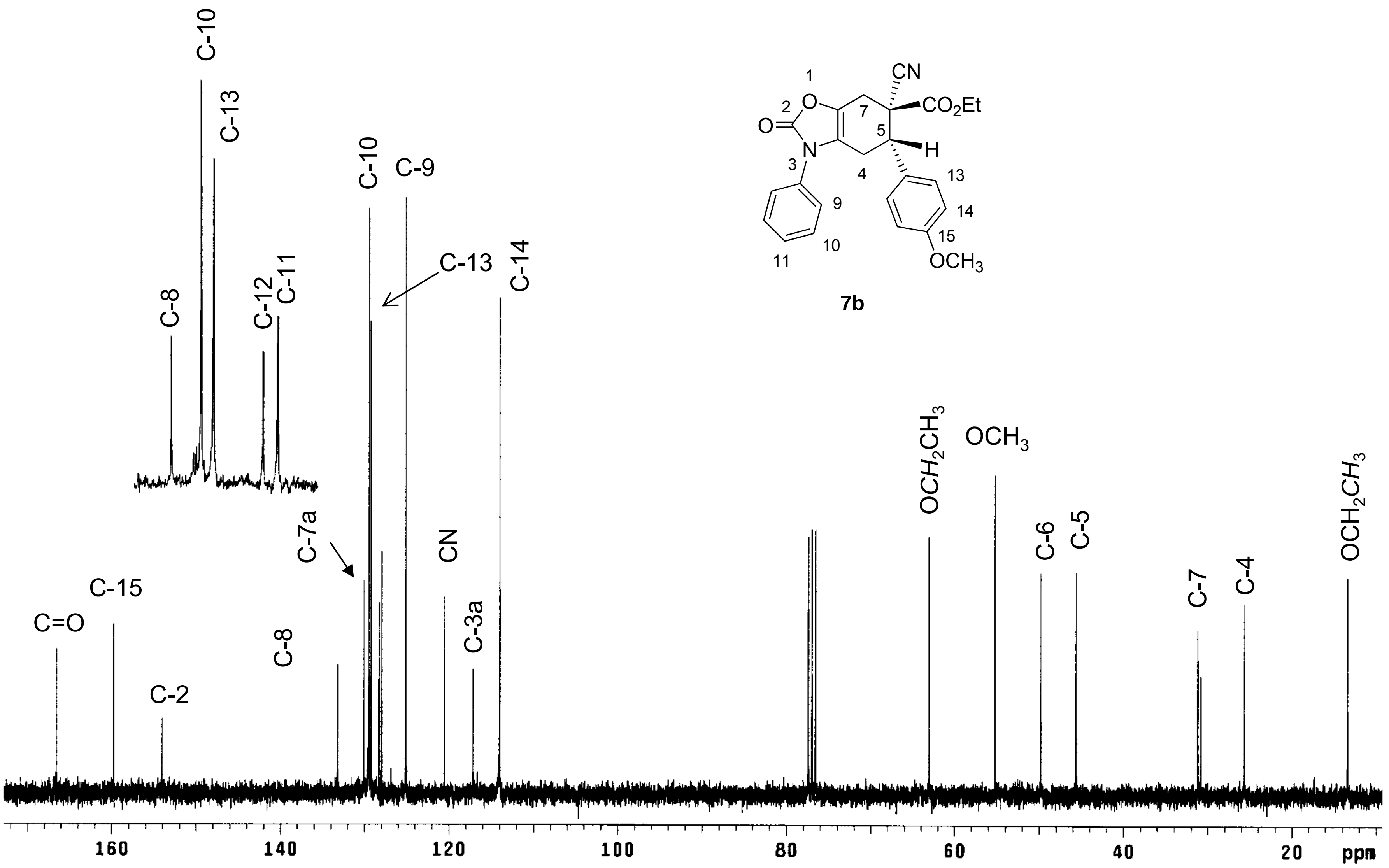

^{13}C NMR ($CDCl_3$) **7b**

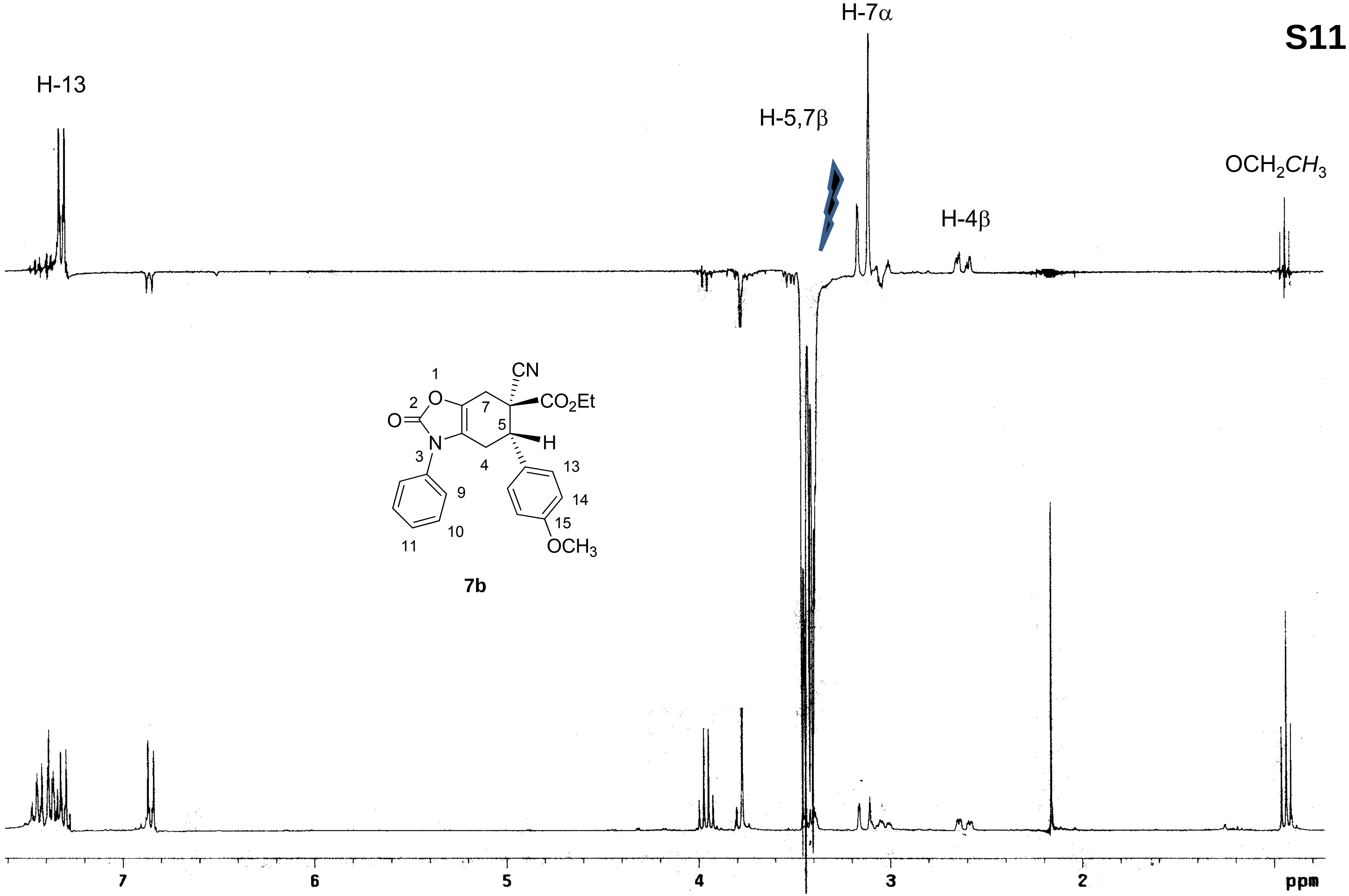

NOE experiment ($CDCl_3$) **7b**

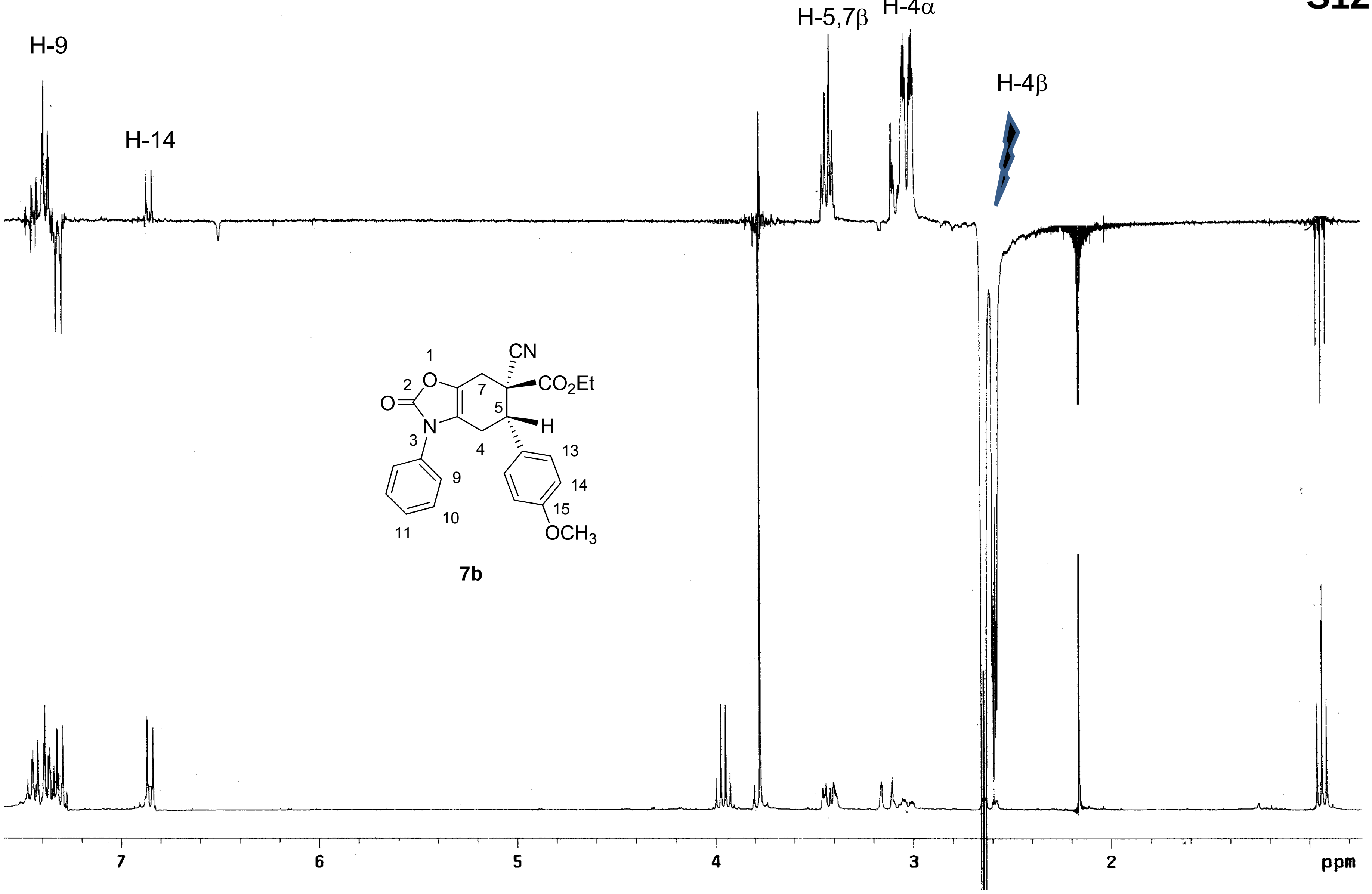

NOE experiment ($CDCl_3$) **7b**

File: Ines MI3 IDIE 418Date Run: 05-29-2008 (Time Run: 23:05:56)
Sample: Ines MI3 IDIE 418
Instrument: JEOL GCmate
Inlet: Direct Probe **Ionization mode: EI+**

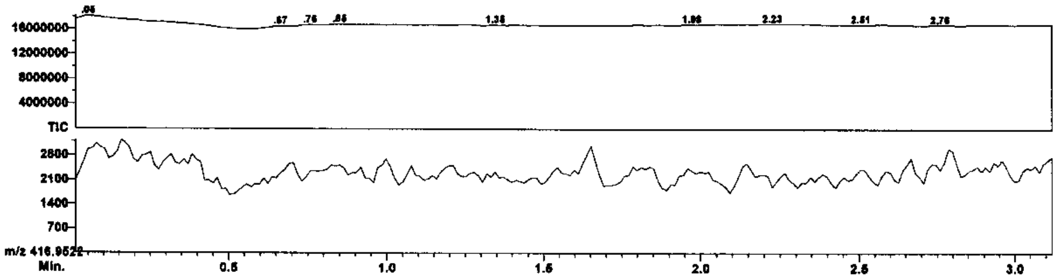

Scan: 124-145 **R.T.: 1.8**
Base: m/z 281; 4.2%FS TIC: 882674 **#Ions: 1773**

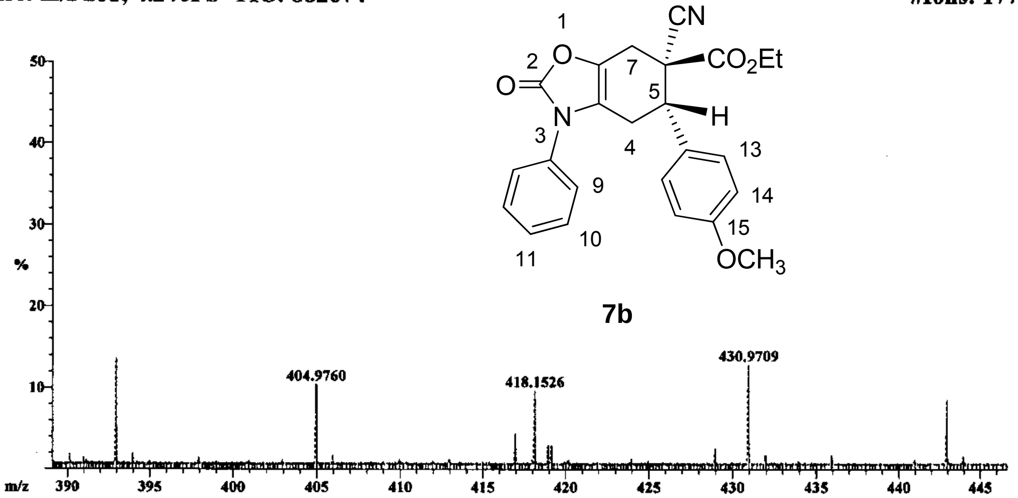

Selected Isotopes : $H_{0\text{-}22}C_{0\text{-}24}N_{0\text{-}2}O_{0\text{-}5}$ **Error Limit : 20 mmu**

Measured Mass	***% Base***	***Formula***	***Calculated Mass***	***Error***
418.1526	9.8%	$C_{24}H_{22}N_2O_5$	418.1529	-0.3

HRMS **7b**

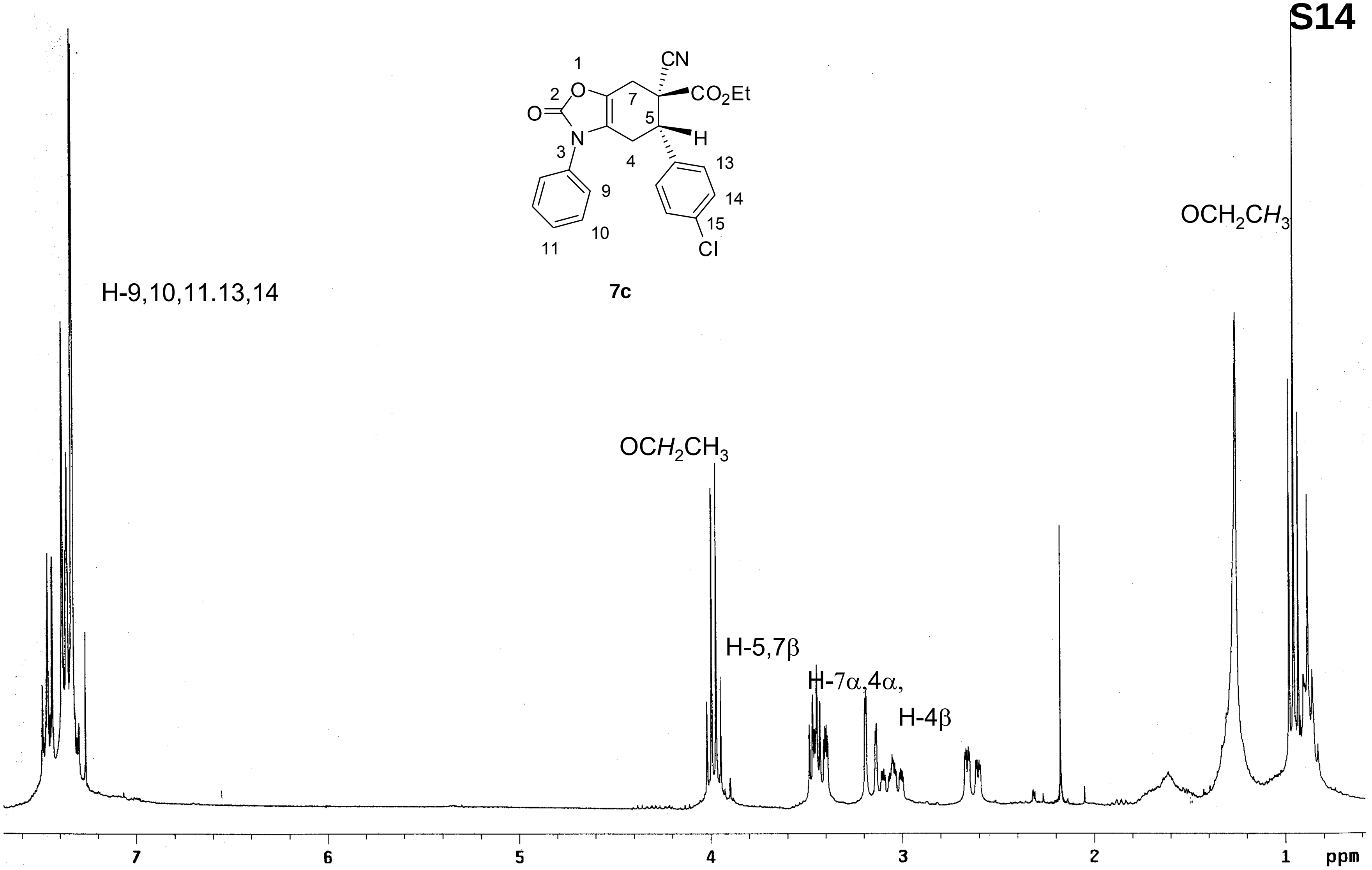

^{1}H NMR ($CDCl_3$) **7c**

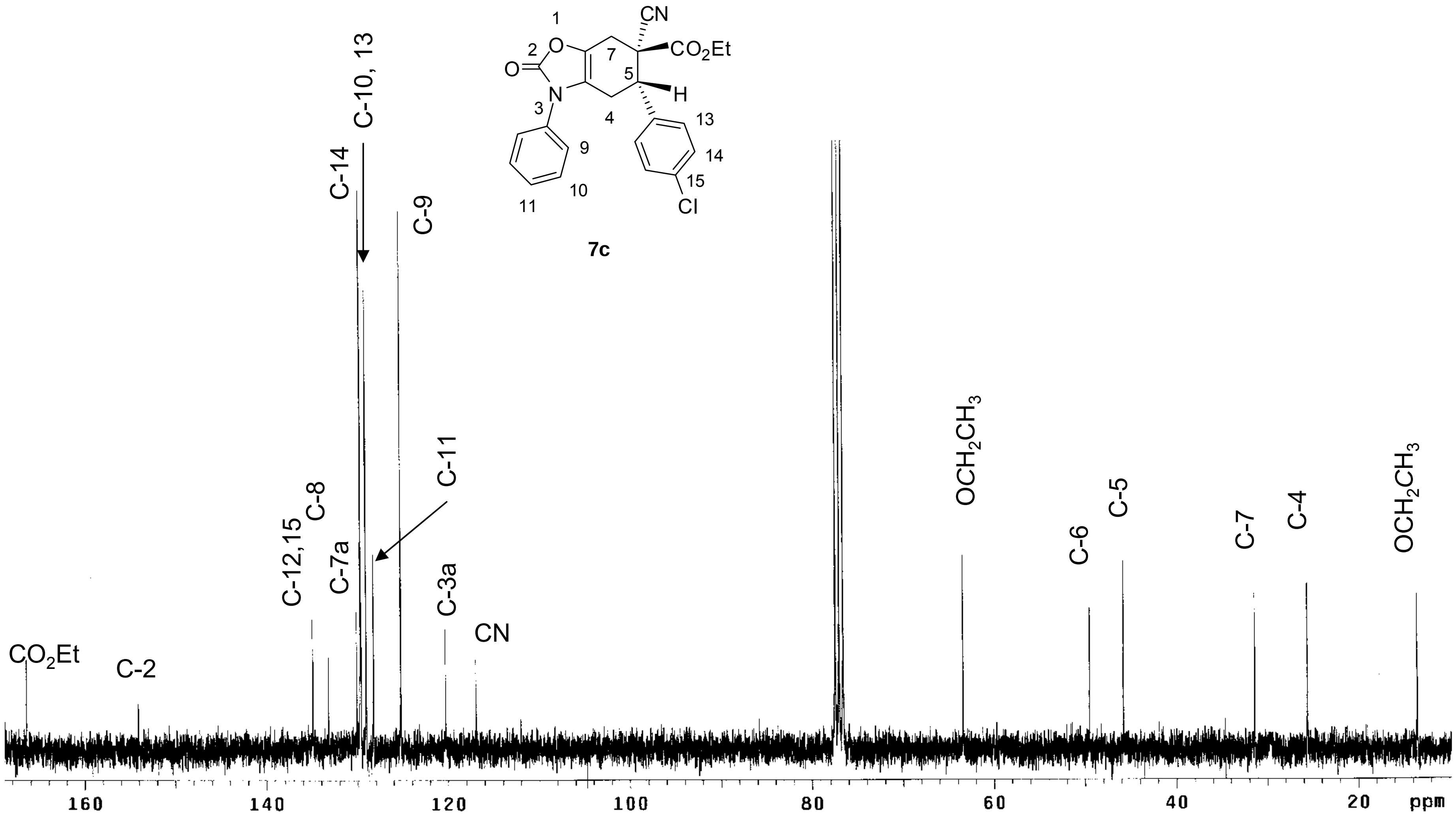

^{13}C NMR (CDCl$_3$) **7c**

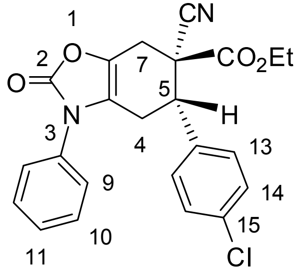

7c

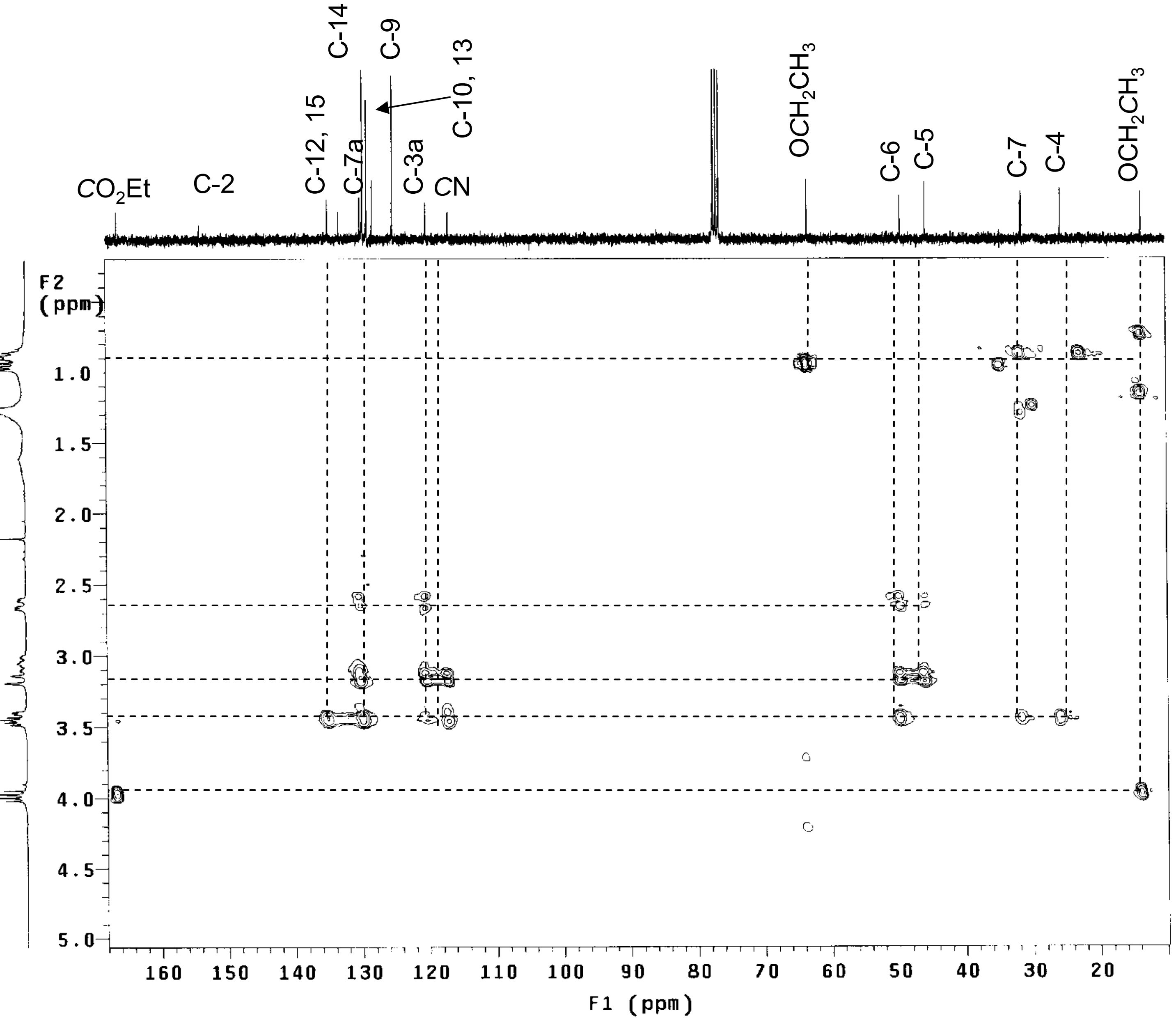

HMBC experiment ($CDCl_3$) **7c**

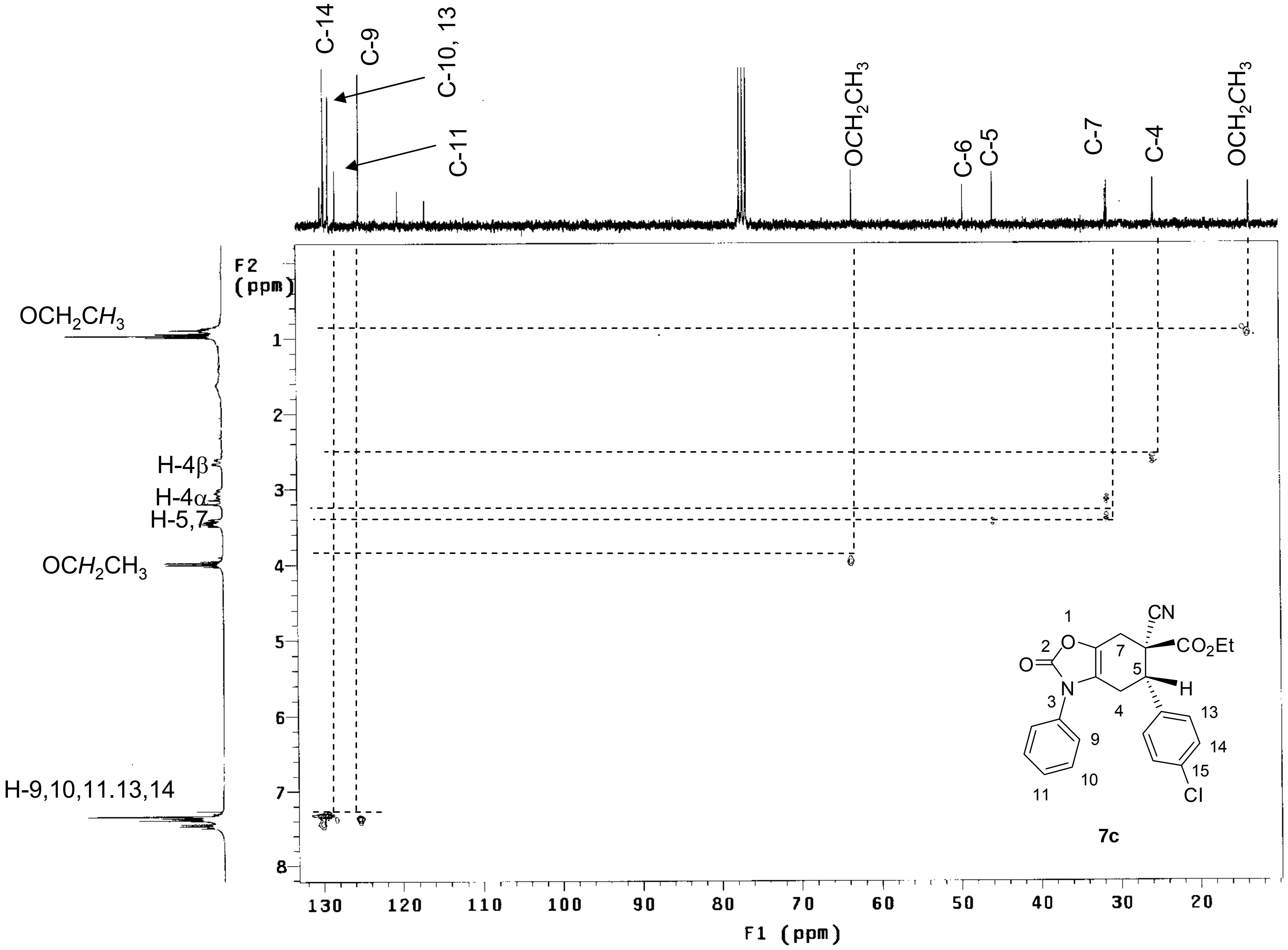

HSQC experiment ($CDCl_3$) **7c**

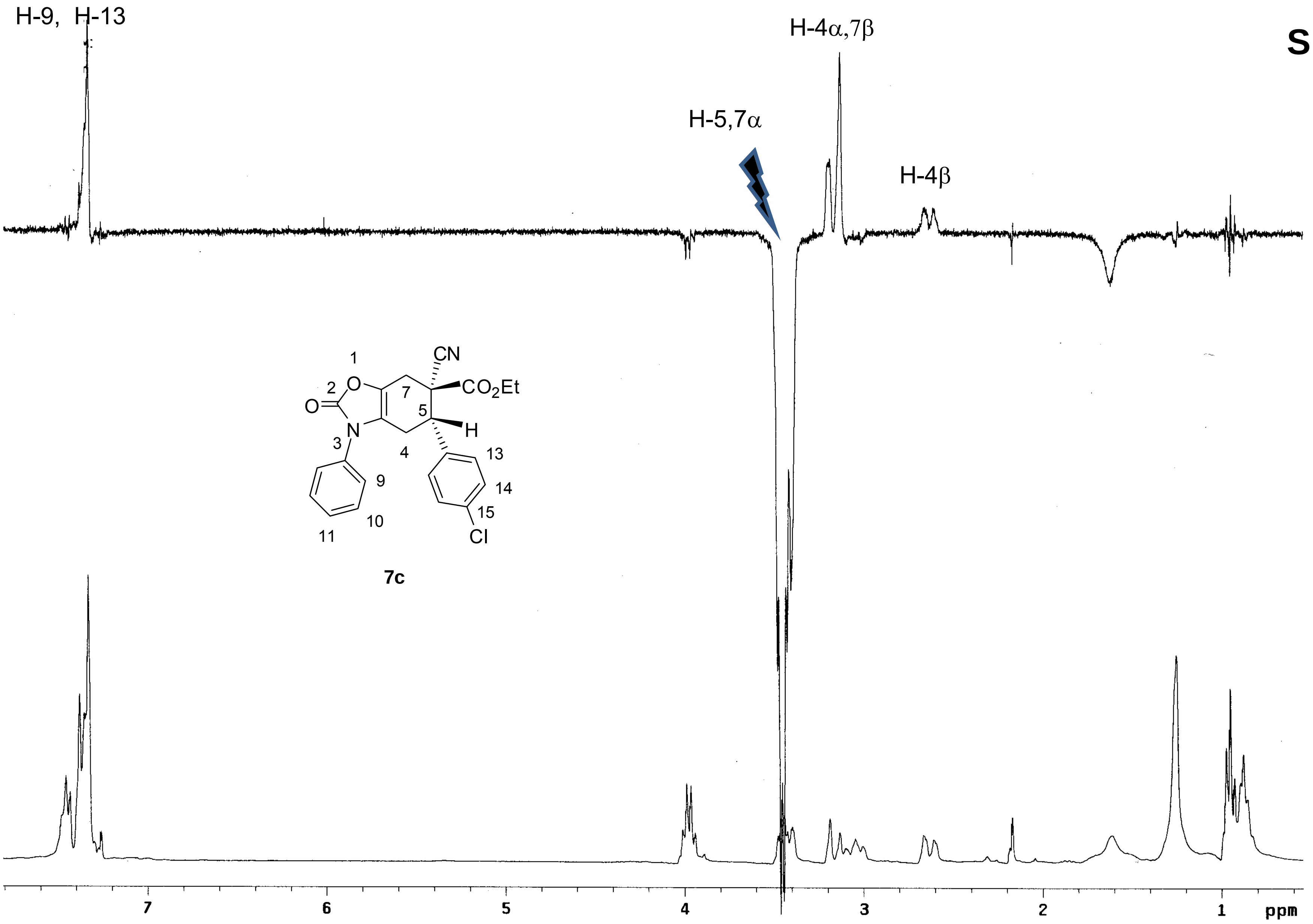

NOE experiment ($CDCl_3$) **7c**

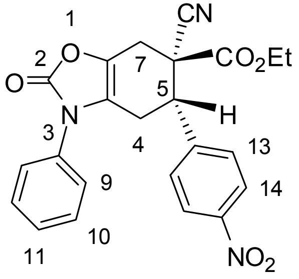

7d

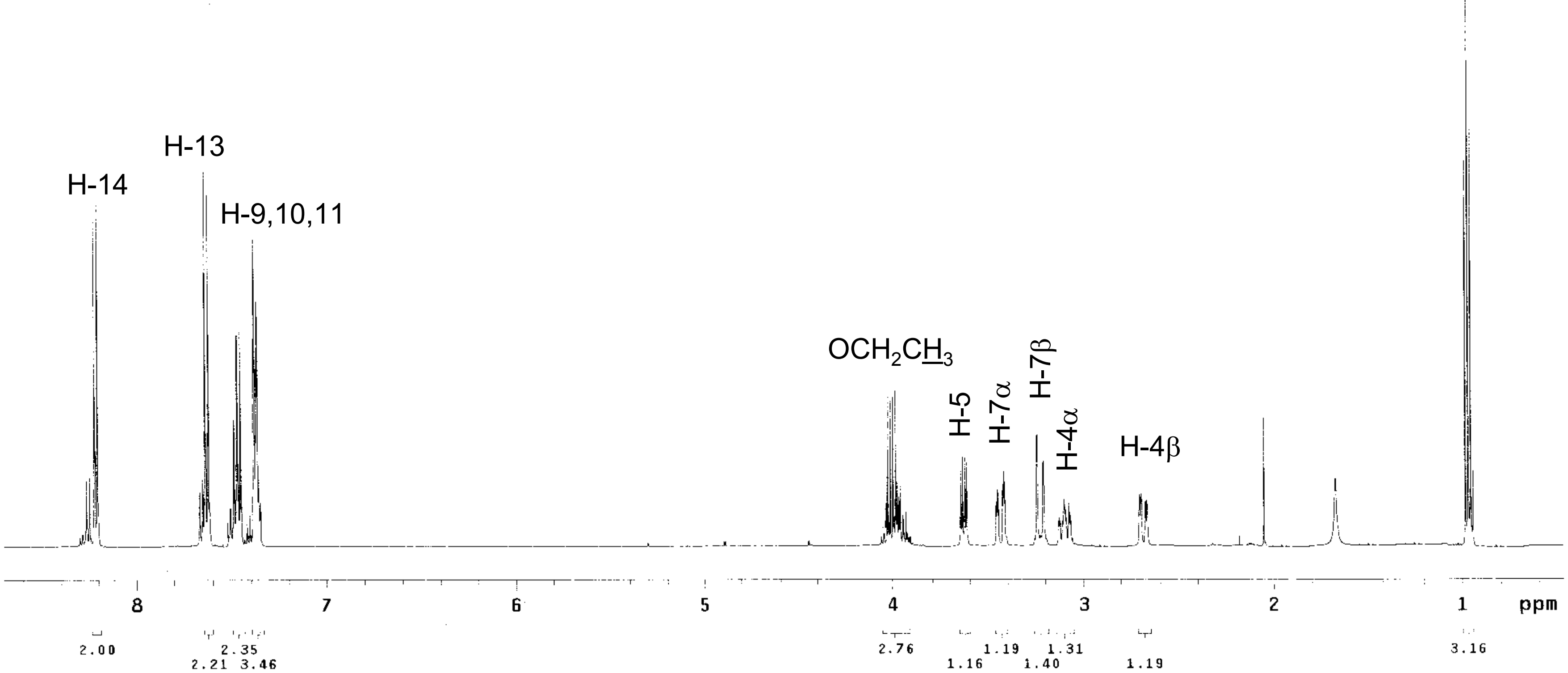

^{1}H NMR ($CDCl_3$) **7d**

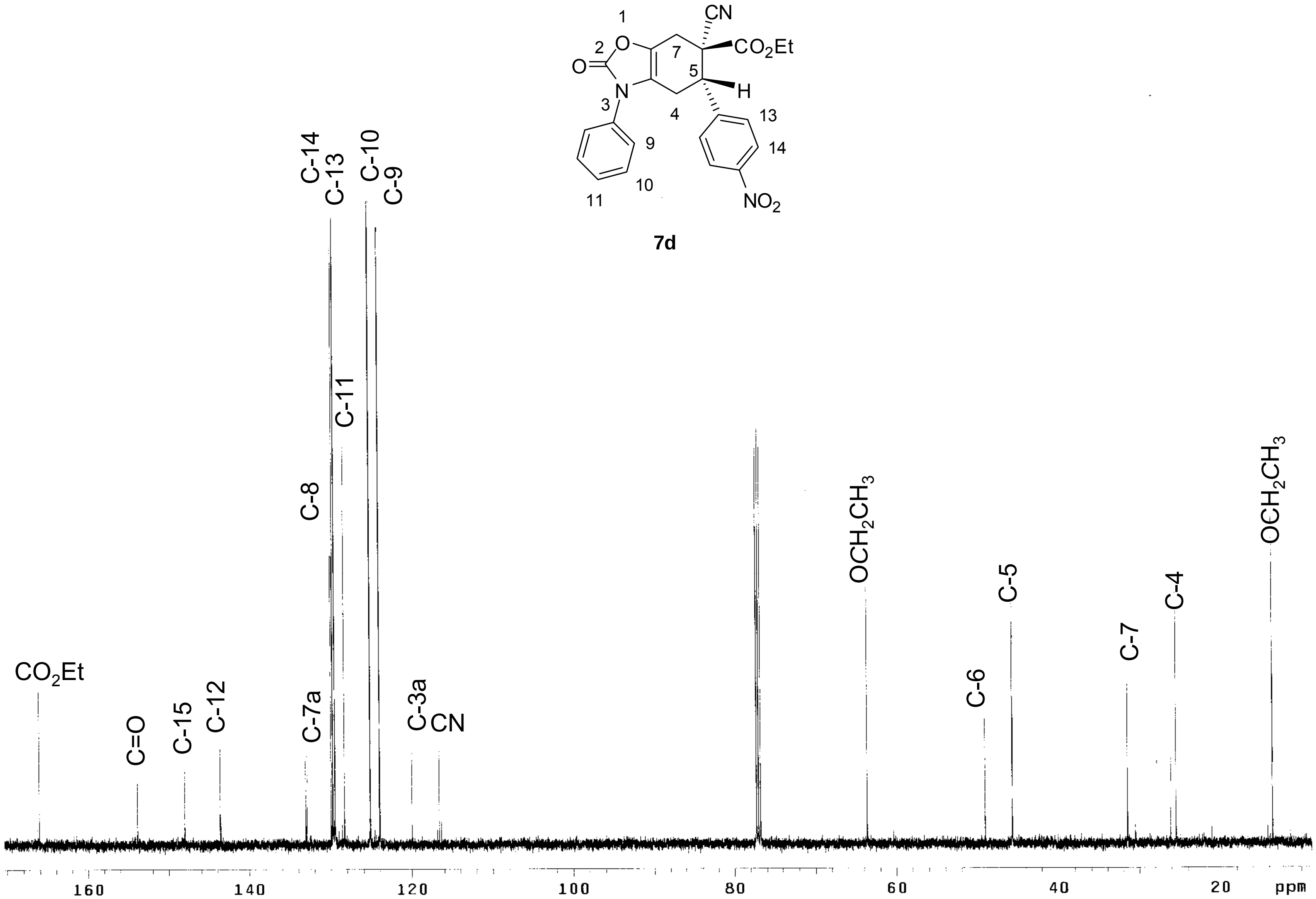

^{13}C NMR ($CDCl_3$) **7d**

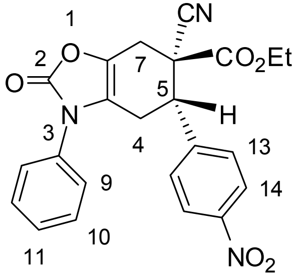

7d

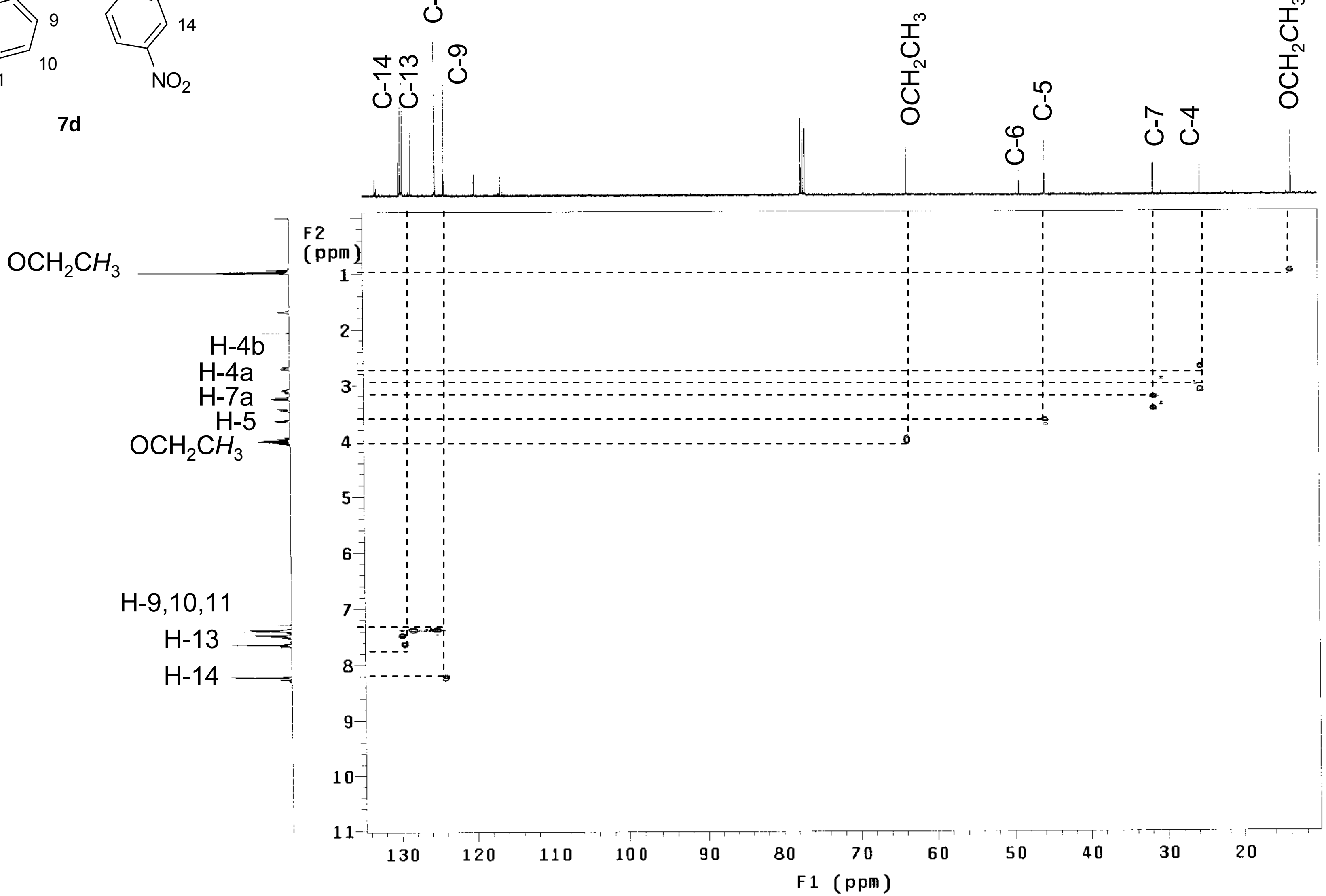

HSQC experiment ($CDCl_3$) **7d**

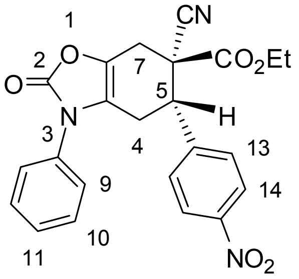

7d

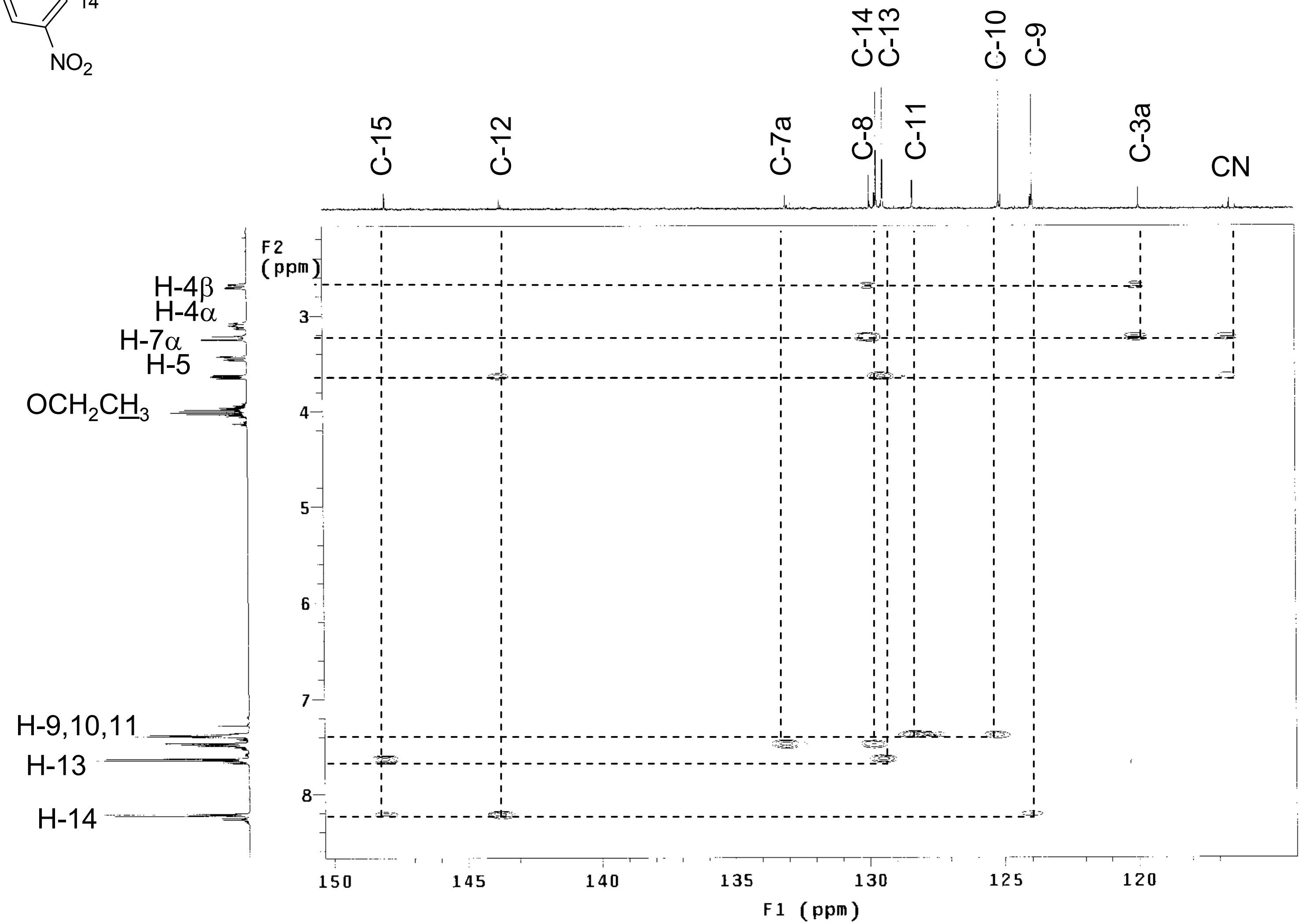

HMBC experiment ($CDCl_3$) **7d**

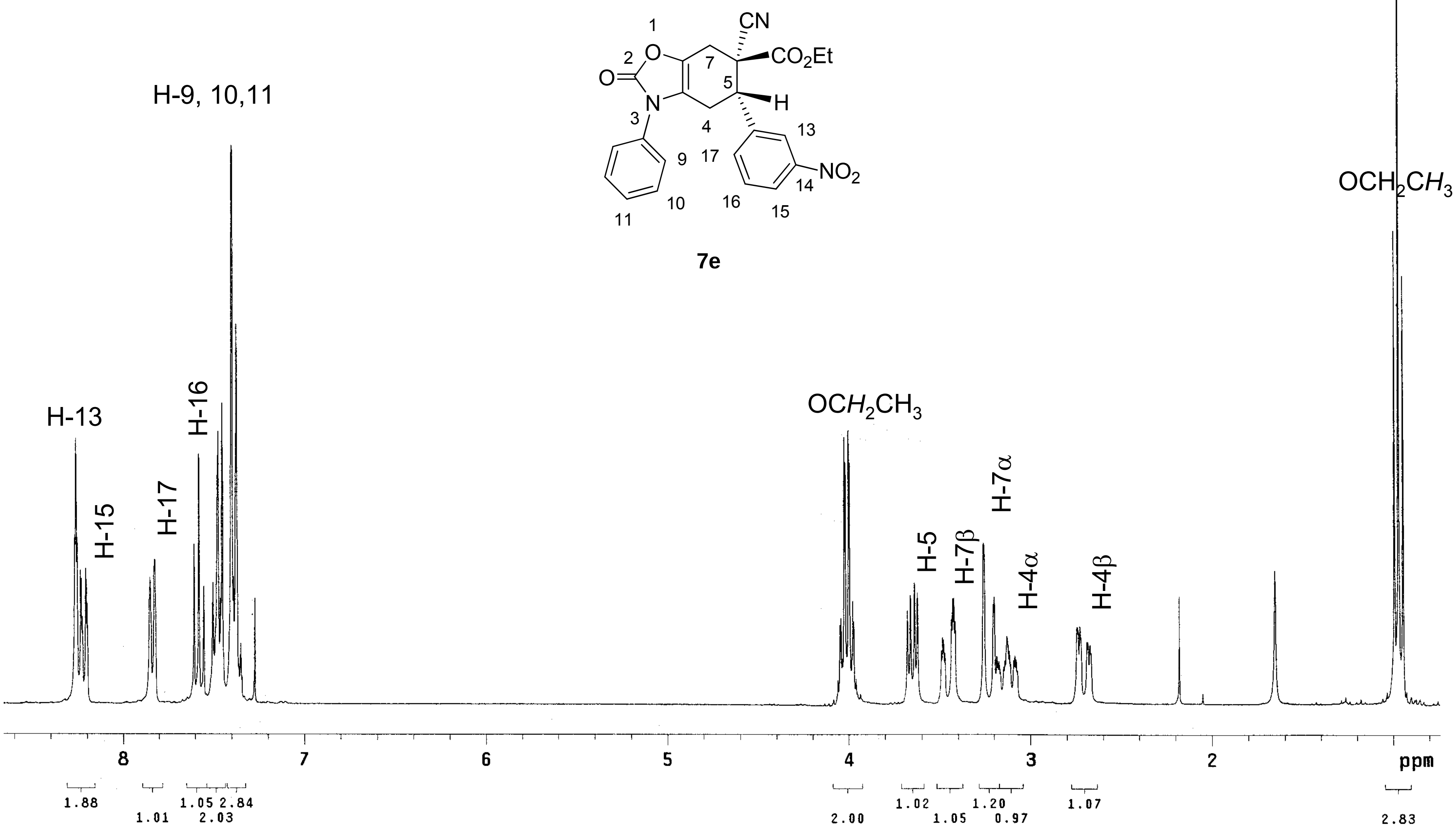

^1H NMR ($CDCl_3$) **7e**

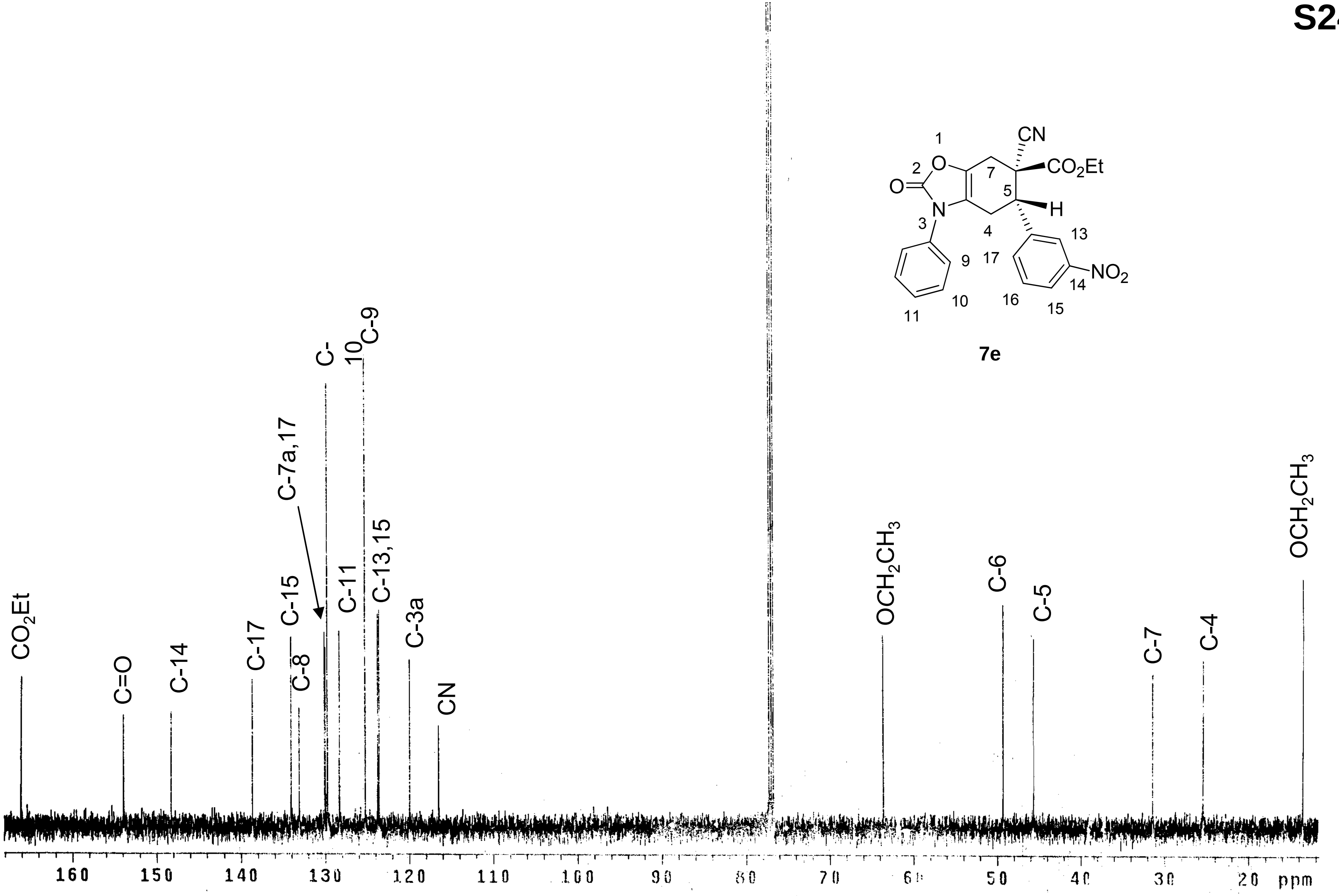

^{13}C NMR ($CDCl_3$) **7e**

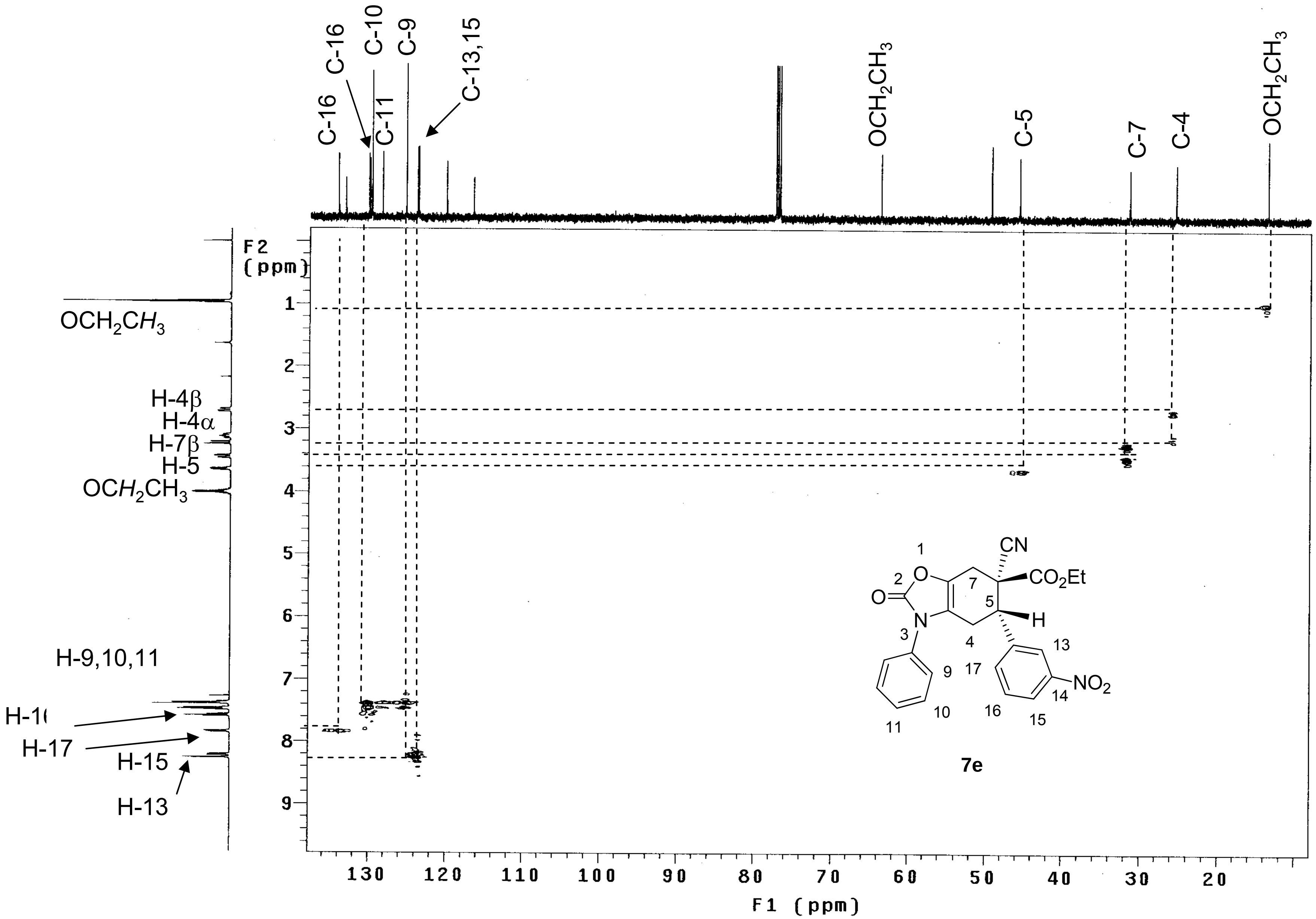

HSQC experiment ($CDCl_3$) **7e**

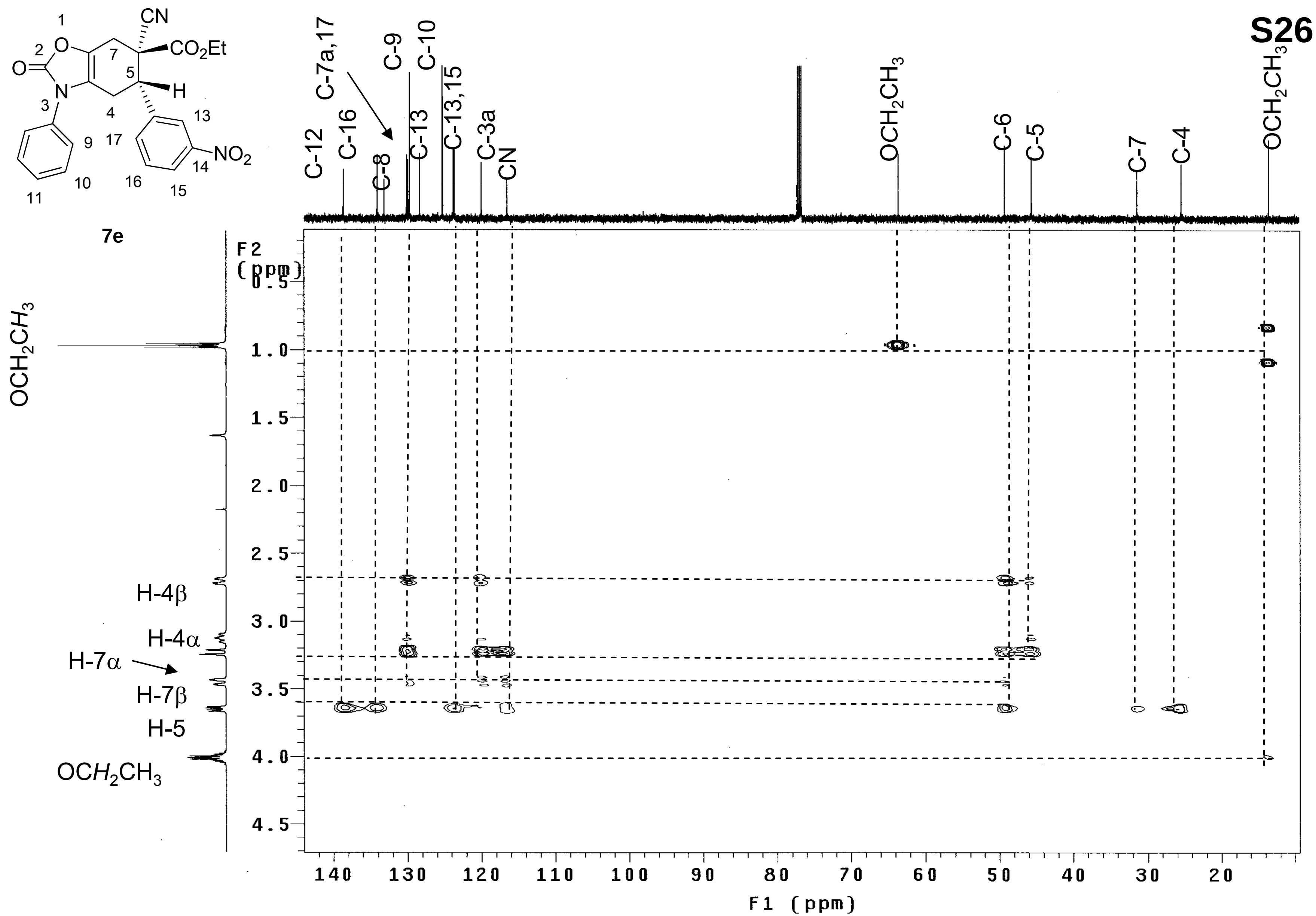

HMBC experiment ($CDCl_3$) **7e**

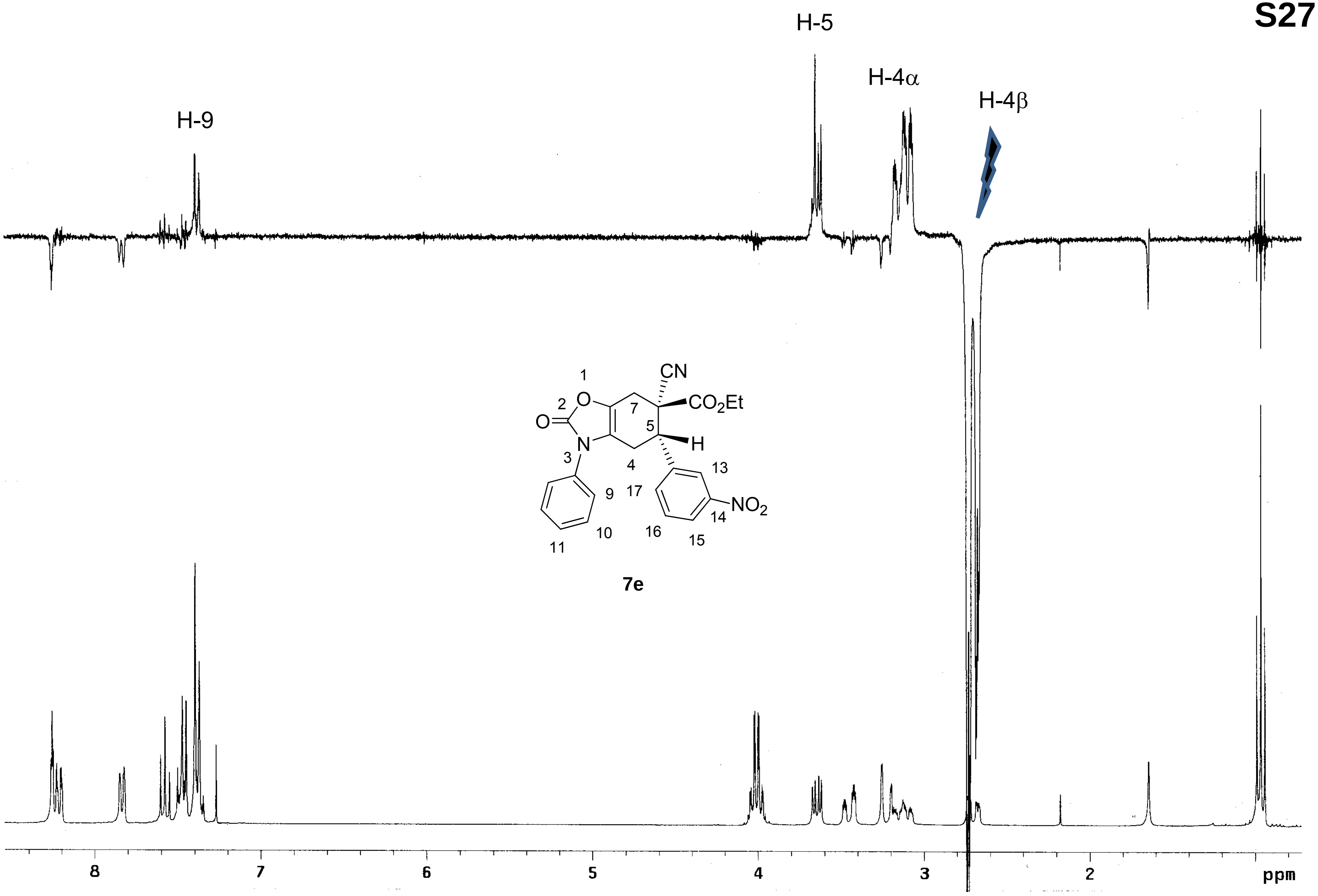

NOE experiment ($CDCl_3$) **7e**

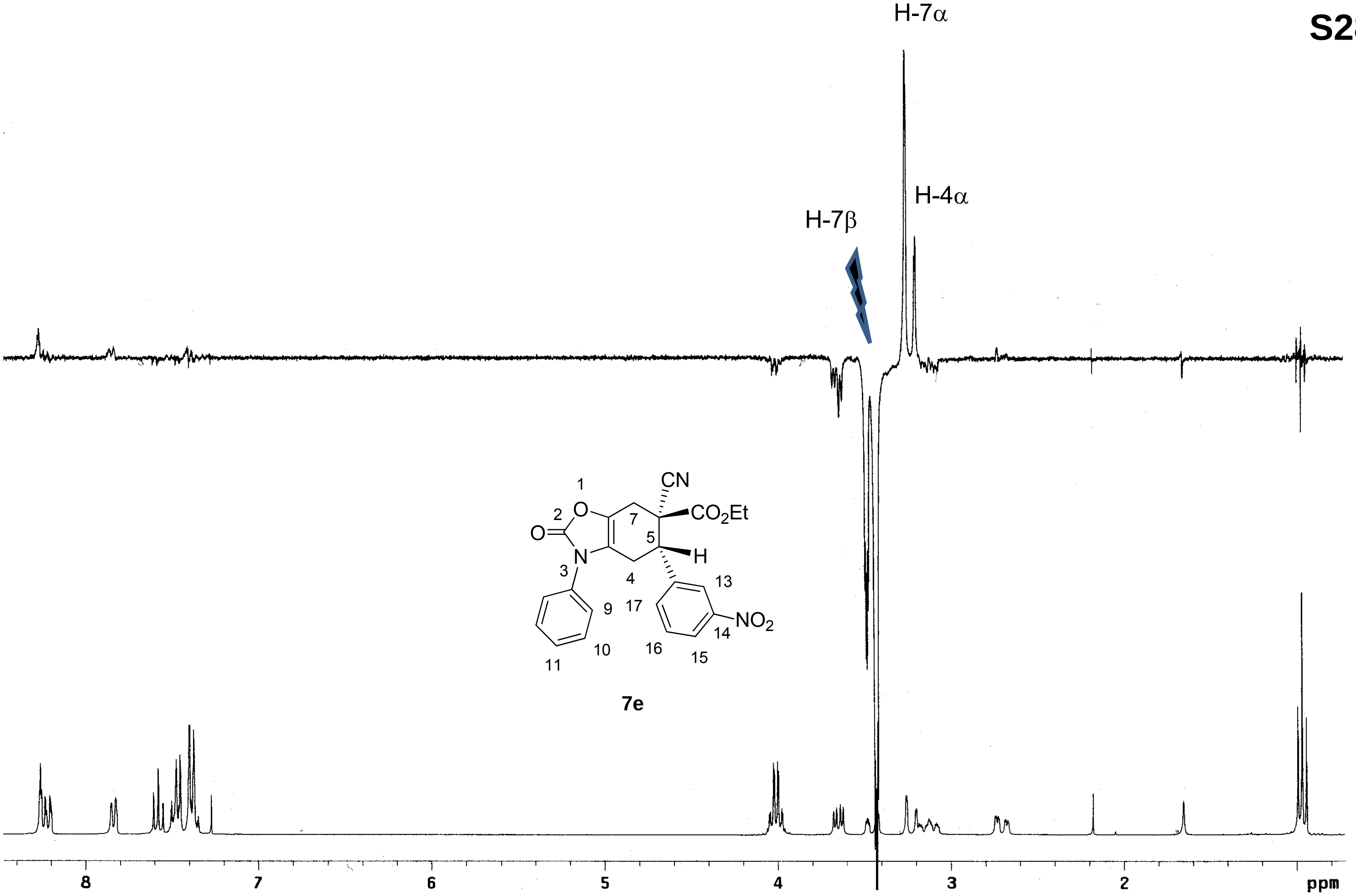

NOE experiment ($CDCl_3$) **7e**

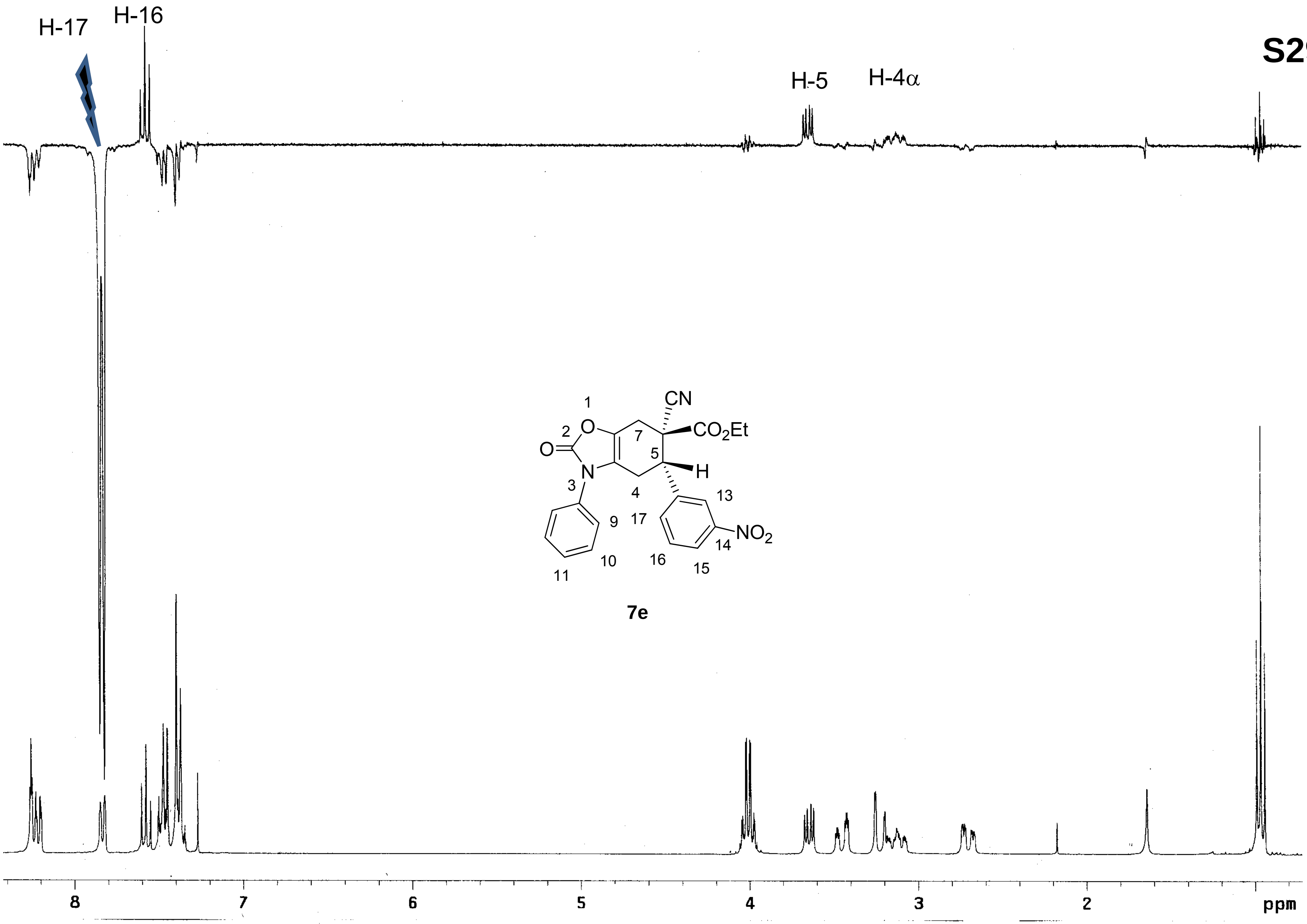

NOE experiment ($CDCl_3$) **7e**

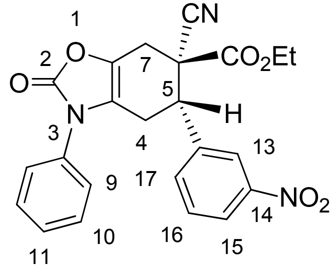

7e

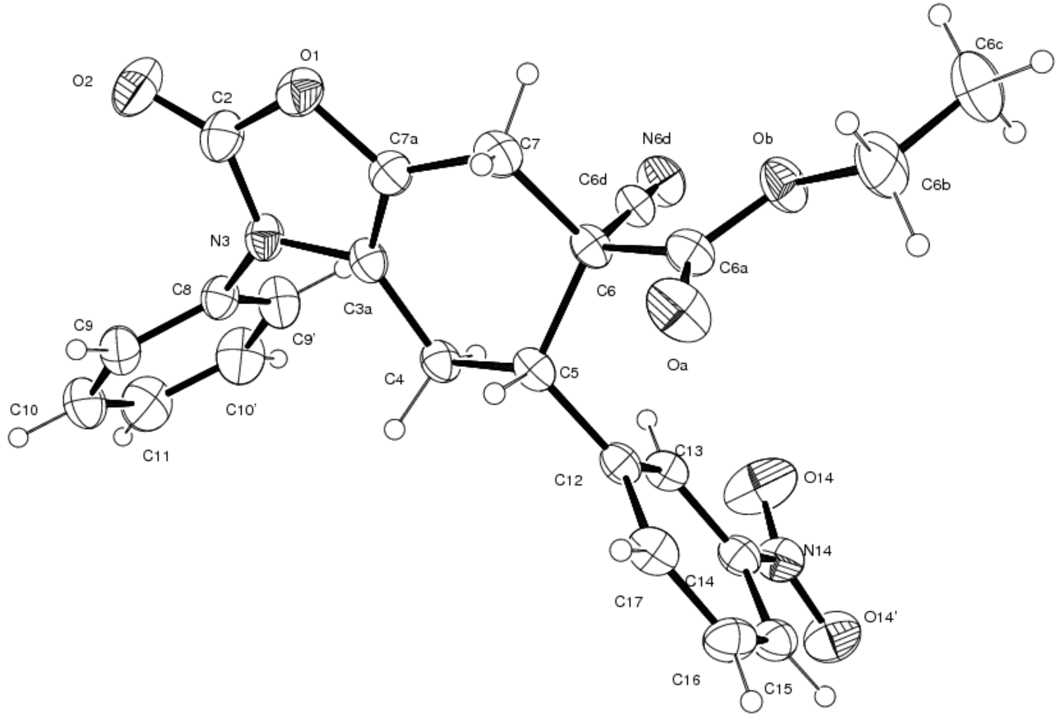

X-Ray structure for **7e**

Table 1. Crystal data and structure refinement for adduct **7e**.

Identification code		0010-fdr
Empirical formula		C23 H19 N3 O6
Formula weight		433.41
Temperature		292(2) K
Wavelength		0.71073 Å
Unit cell dimensions	a = 8.7852(4) Å	α= 91.266(3)°
	b = 10.6923(3) Å	β= 99.236(3)°.
	c = 12.0266(5) Å	γ = 109.440(3)°.
Volume		1048.03(7) Å3
Z		2
Density (calculated)		1.373 Mg/m3
Absorption coefficient		0.101 mm-1
F(000)		452
Crystal size		0.22 x 0.15 x 0.13 mm3
Theta range for data collection		2.50 to 32.54°.
Index ranges		-13<=h<=13, -15<=k<=16, -18<=l<=17
Reflections collected		14935
Independent reflections		6788 [R(int) = 0.0283]
Completeness to theta = 27.50°		99.1 %
Max. and min. transmission		0.9870 and 0.9781

Refinement method	Full-matrix least-squares on F^2
Data / restraints / parameters	6788 / 0 / 290
Goodness-of-fit on F^2	1.003
Final R indices [I>2sigma(I)]	R1 = 0.0522, wR2 = 0.1262
R indices (all data)	R1 = 0.1046, wR2 = 0.1460
Largest diff. peak and hole	0.270 and -0.254 e.Å-3

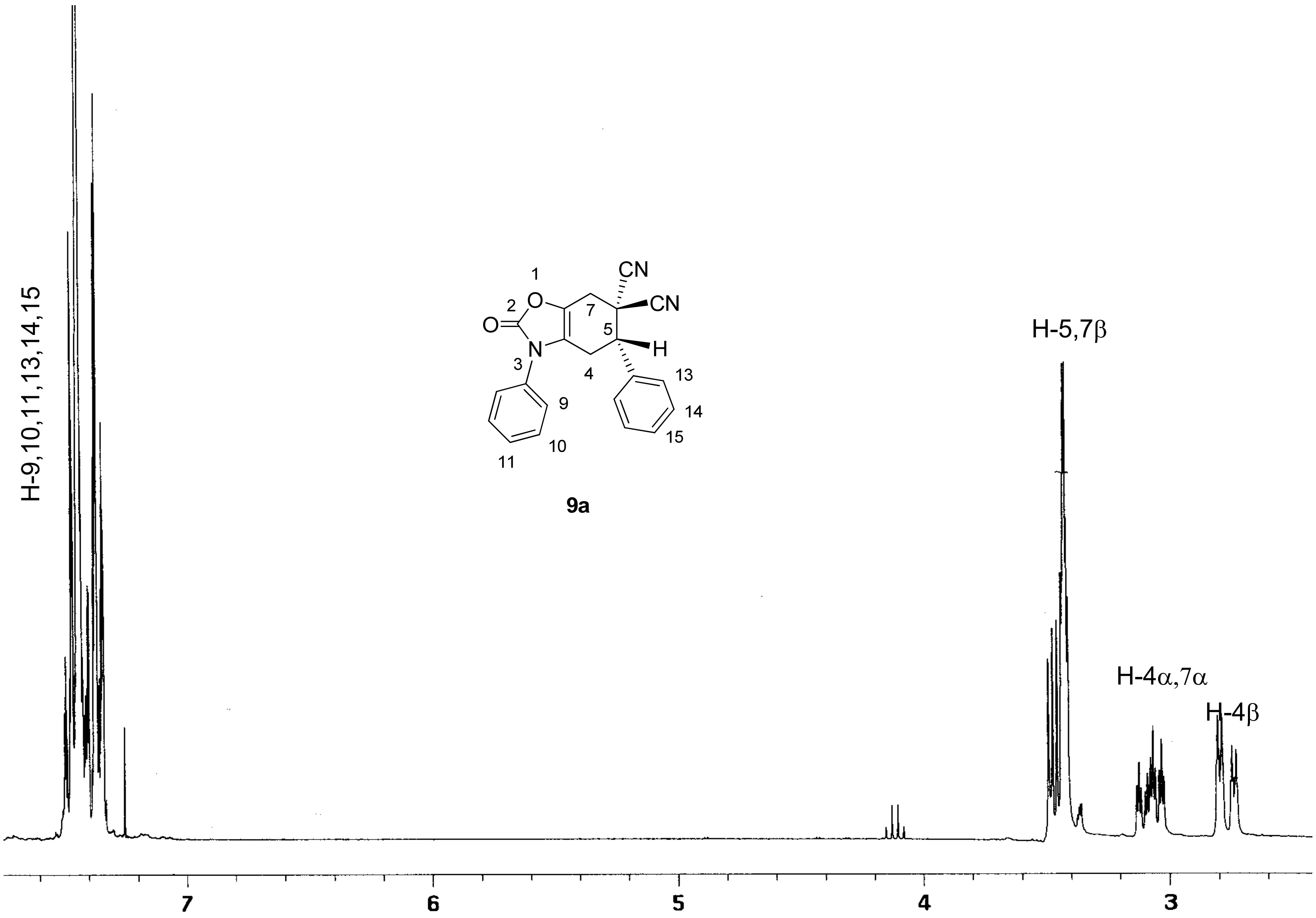

^{1}H NMR ($CDCl_3$) **9a**

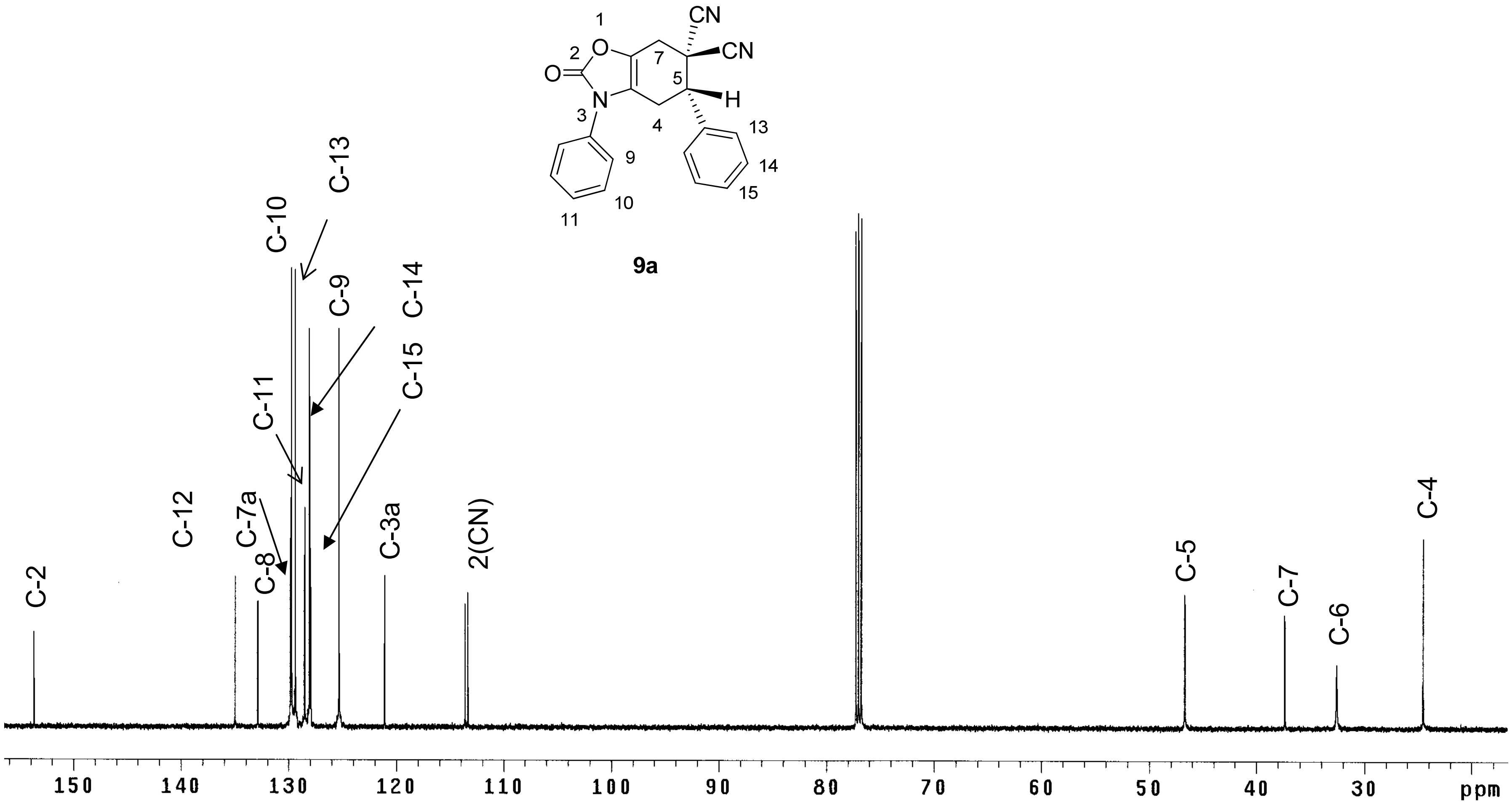

^{13}C NMR ($CDCl_3$) **9a**

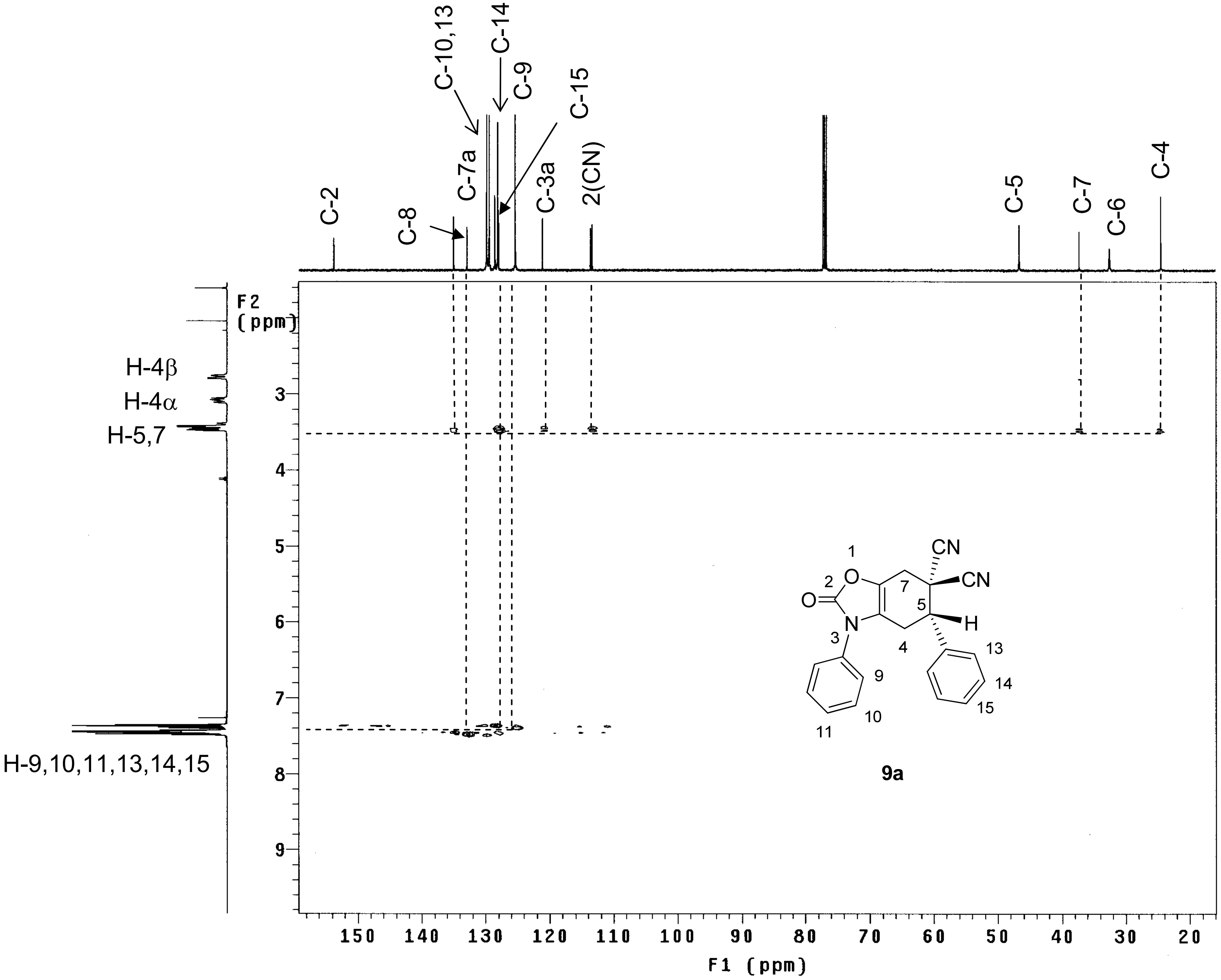

HSQC experiment ($CDCl_3$) **9a**

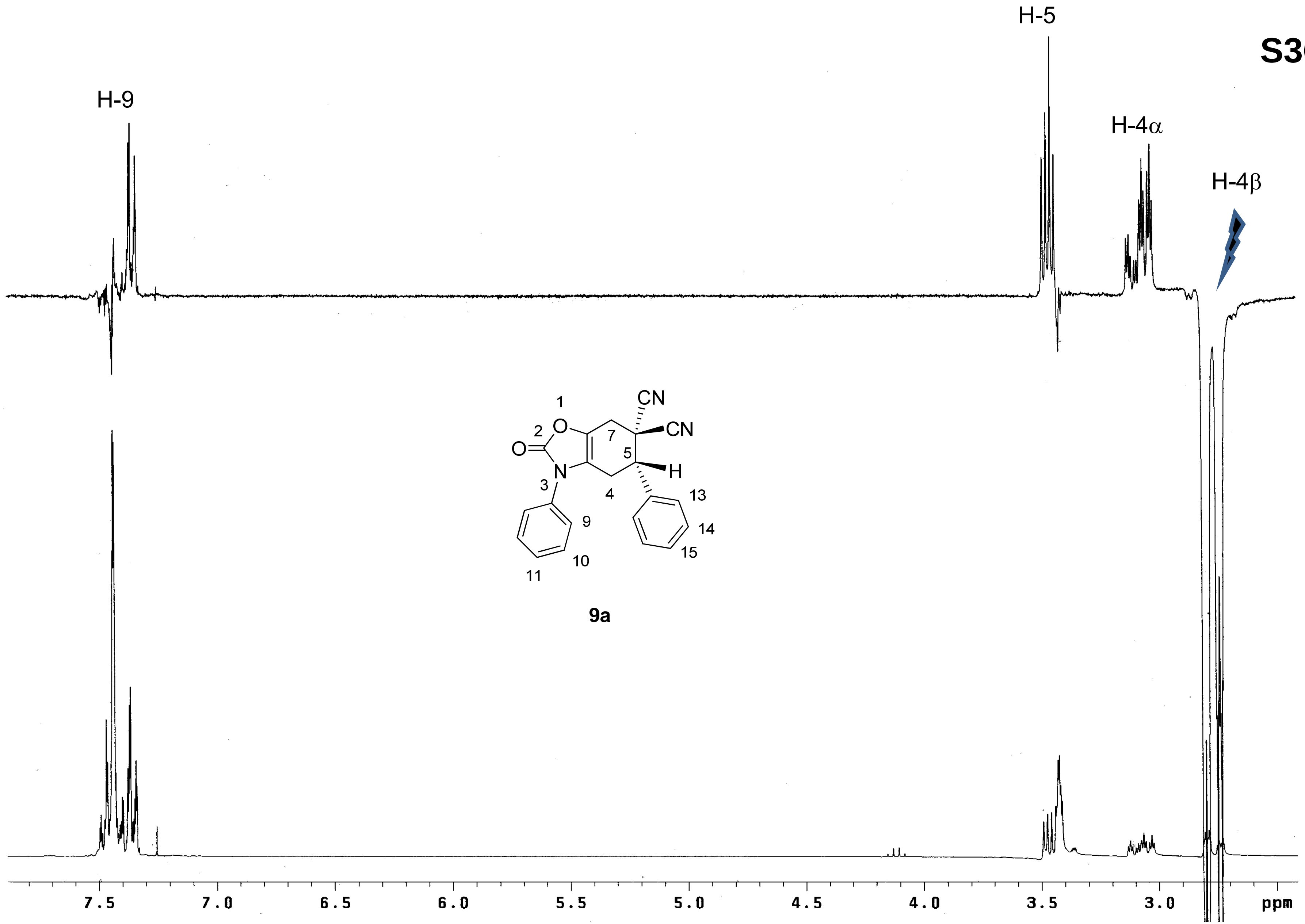

NOE experiment ($CDCl_3$) **9a**

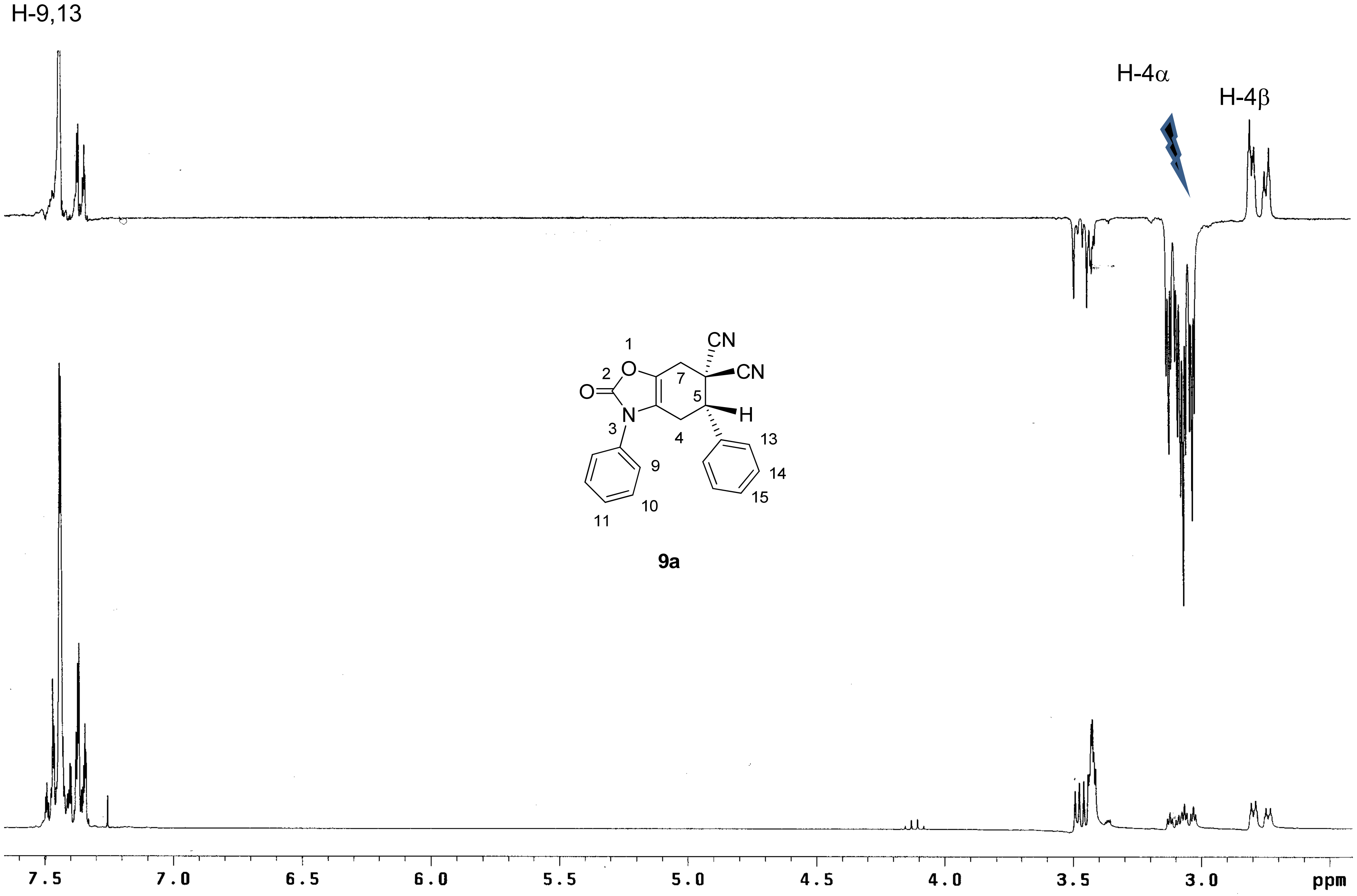

NOE experiment ($CDCl_3$) **9a**

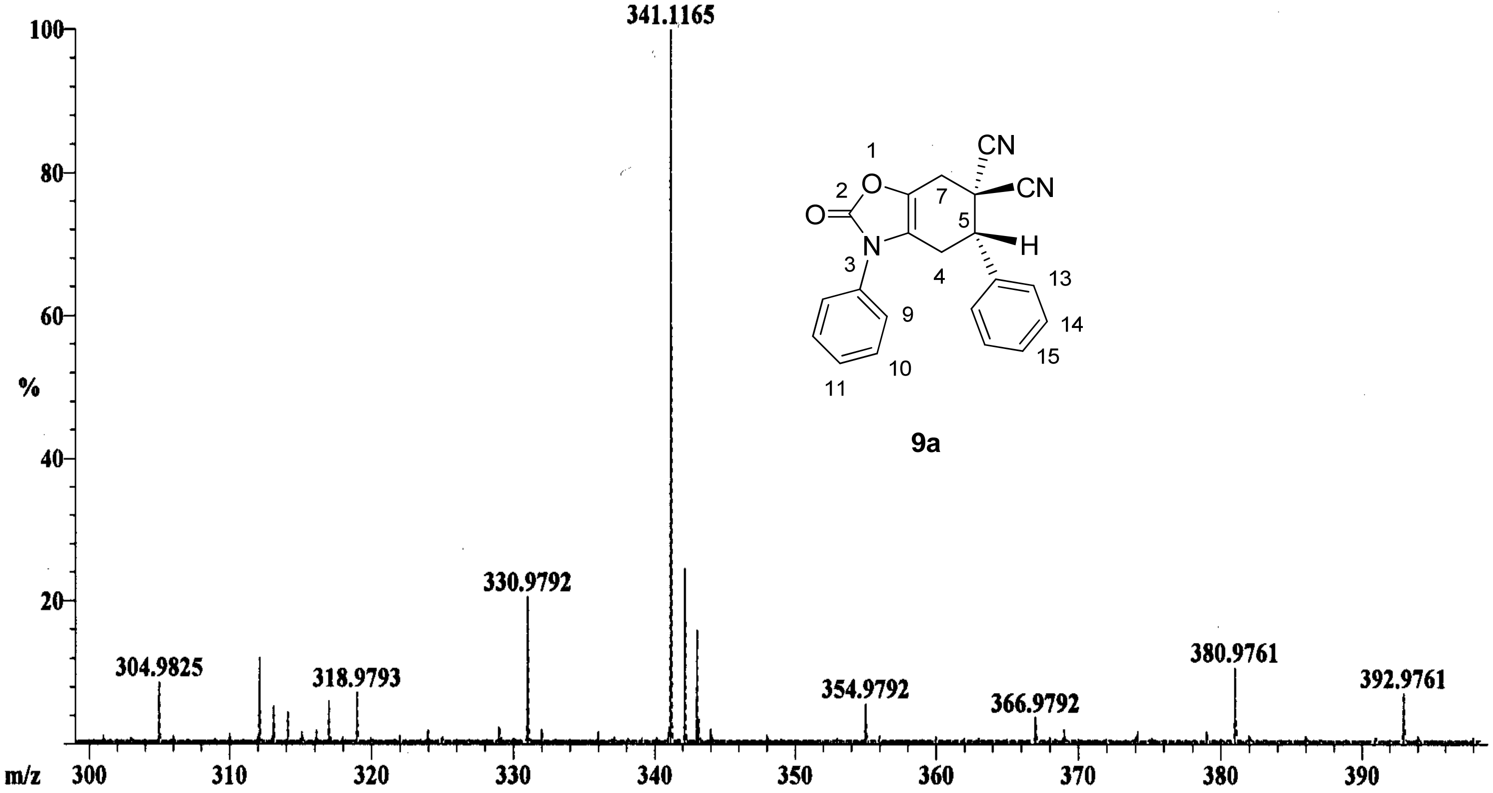

9a

Selected Isotopes : $N_{0-3}C_{0-21}H_{0-15}O_{0-2}$

Error Limit : 20 mmu

Measured Mass	% Base	Formula	Calculated Mass	Error
341.1165	100.0%	$C_{21}H_{15}N_3O_2$	341.1164	0.1

HRMS **9a**

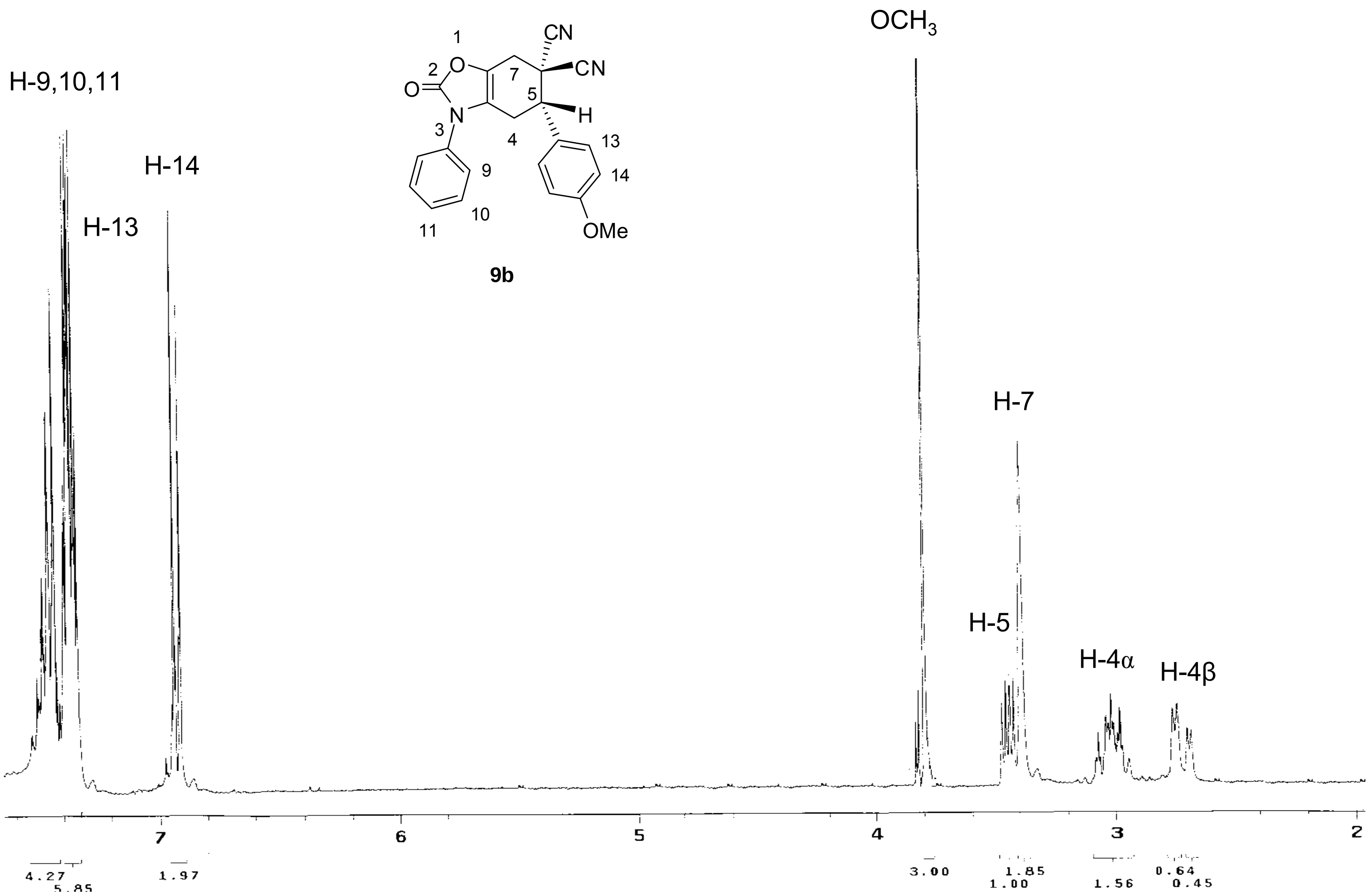

^{1}H NMR ($CDCl_3$) **9b**

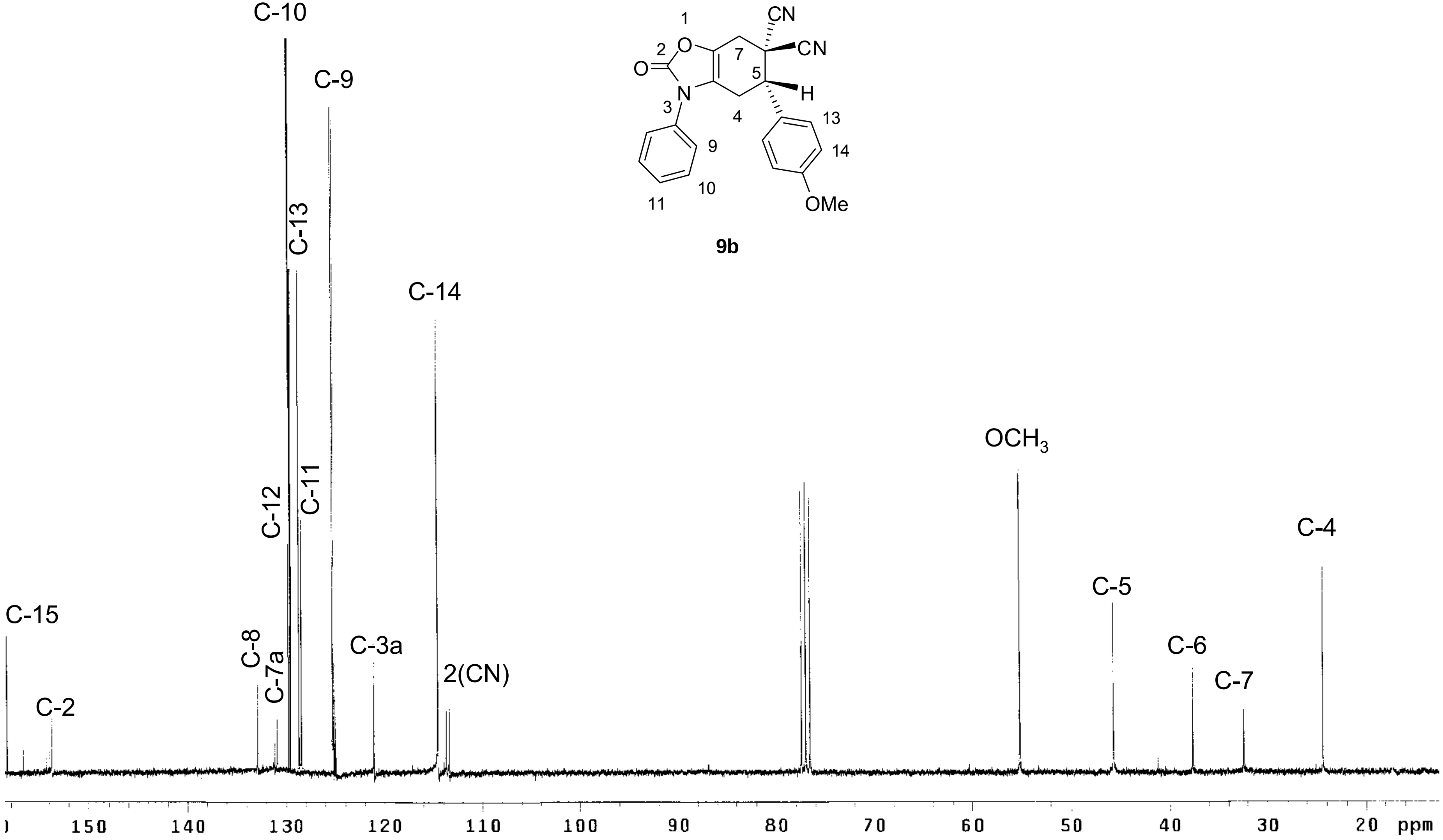
C-10
C-9
C-13
C-14
C-12
C-11
C-15
C-2
C-8
C-7a
C-3a
2(CN)
OCH3
C-5
C-6
C-7
C-4
1
2
3
4
5
7
9
10
11
13
14
O
N
CN
CN
H
OMe
9b
150
140
130
120
110
100
90
80
70
60
50
40
30
20
ppm

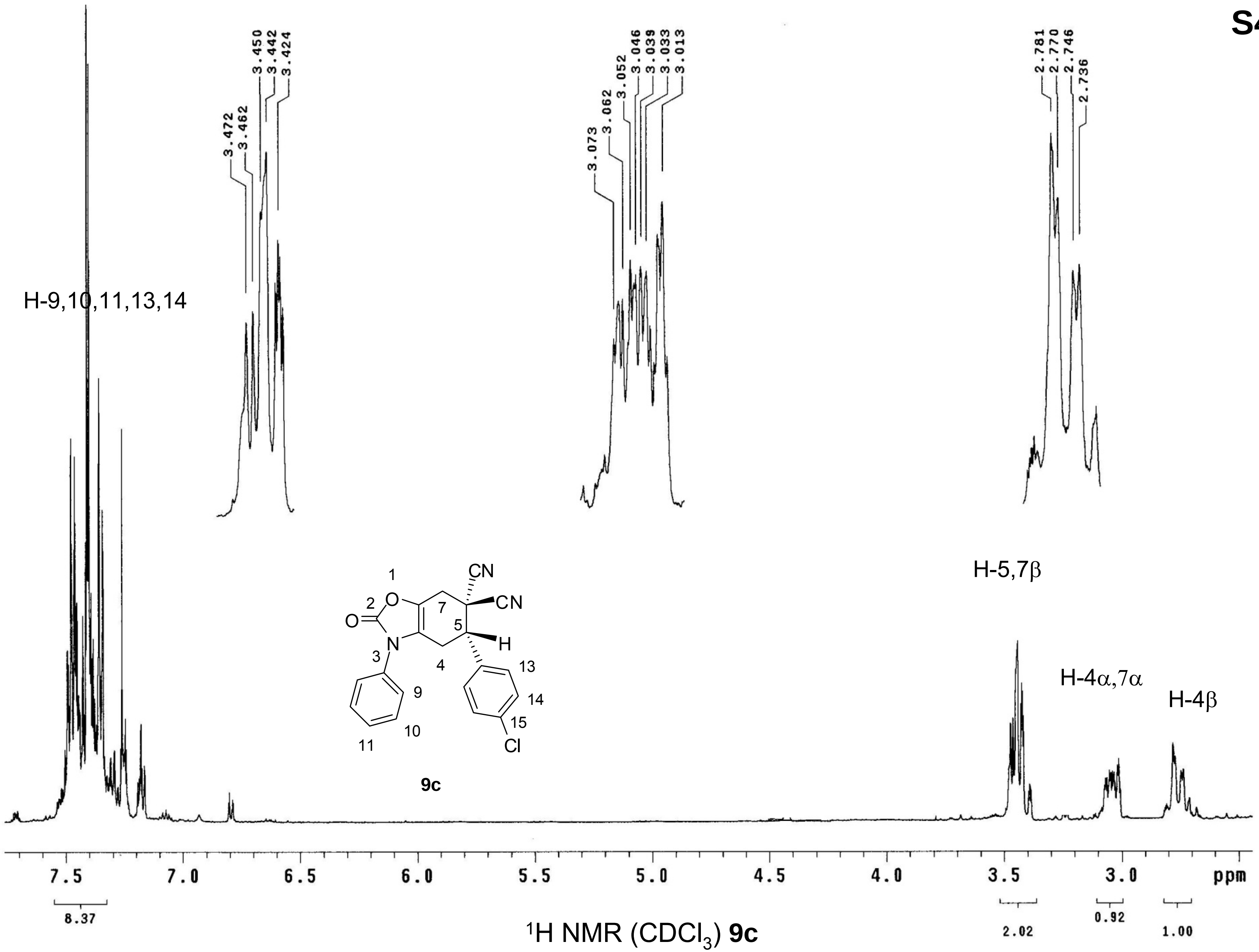

^{1}H NMR ($CDCl_3$) **9c**

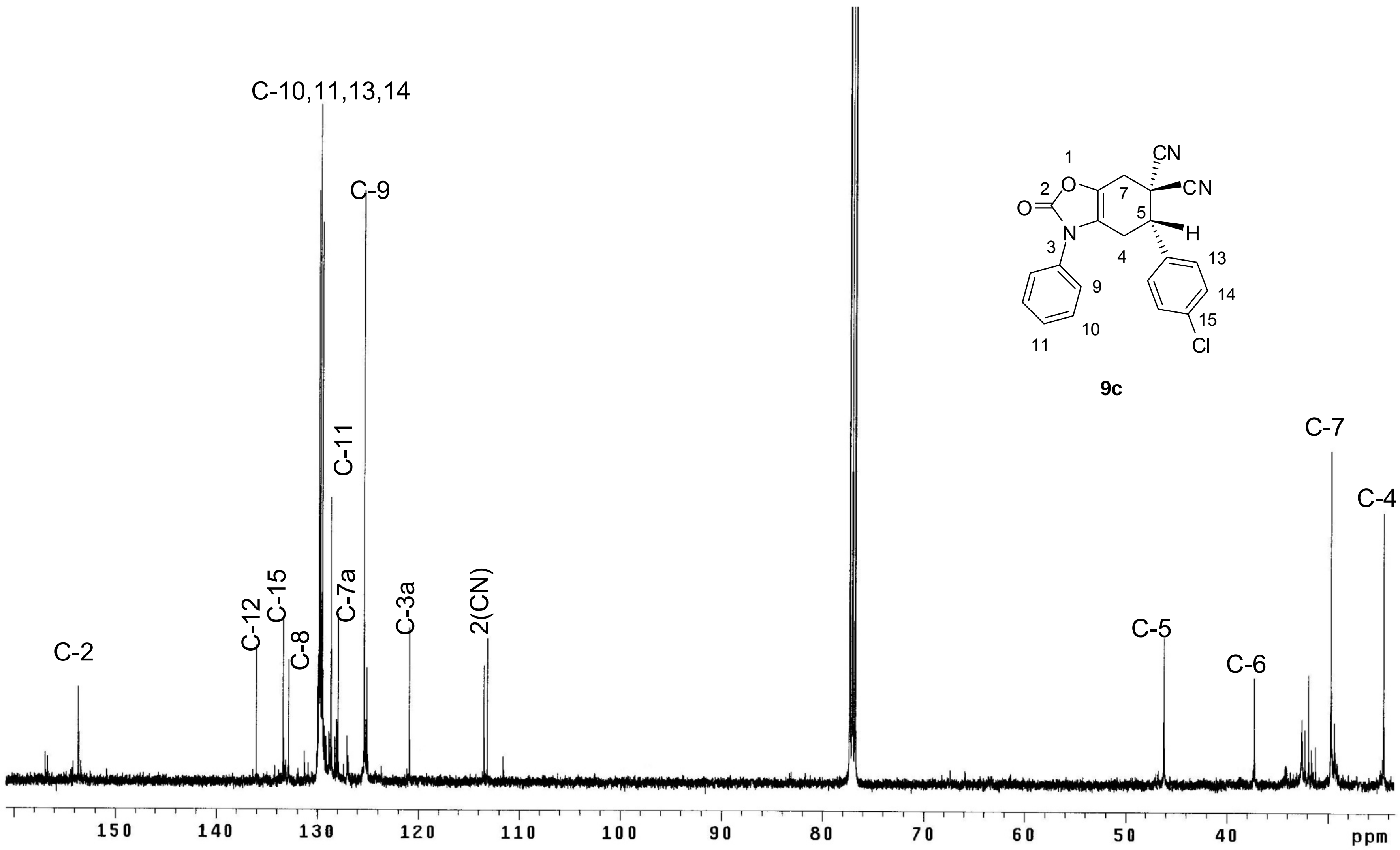

^{13}C NMR ($CDCl_3$) **9c**

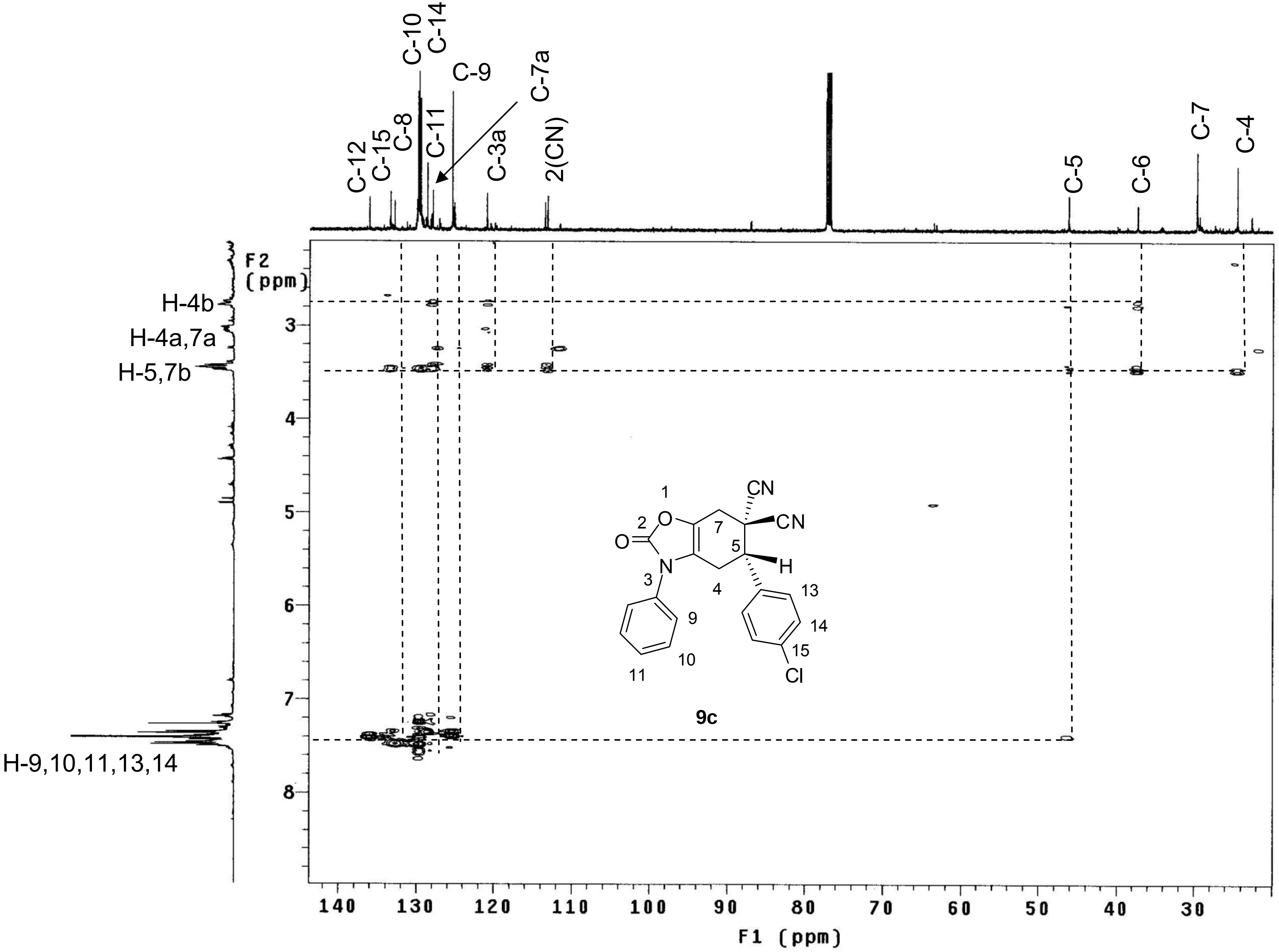

HMBC experiment ($CDCl_3$) **9c**

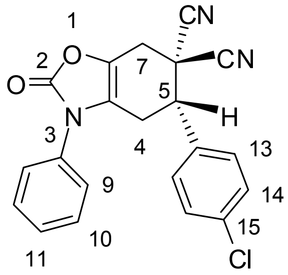

9c

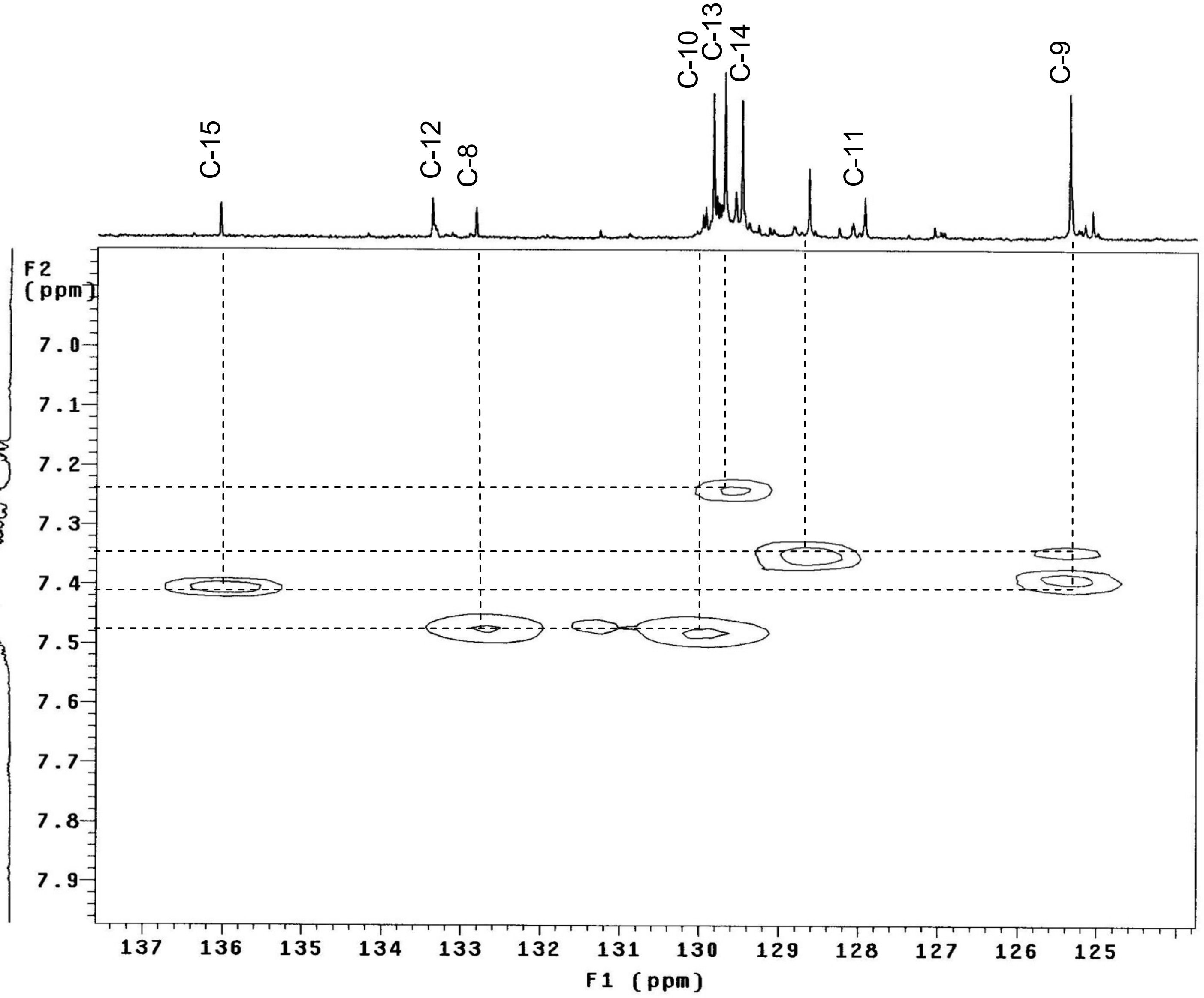

HSQC experiment ($CDCl_3$) **9c**

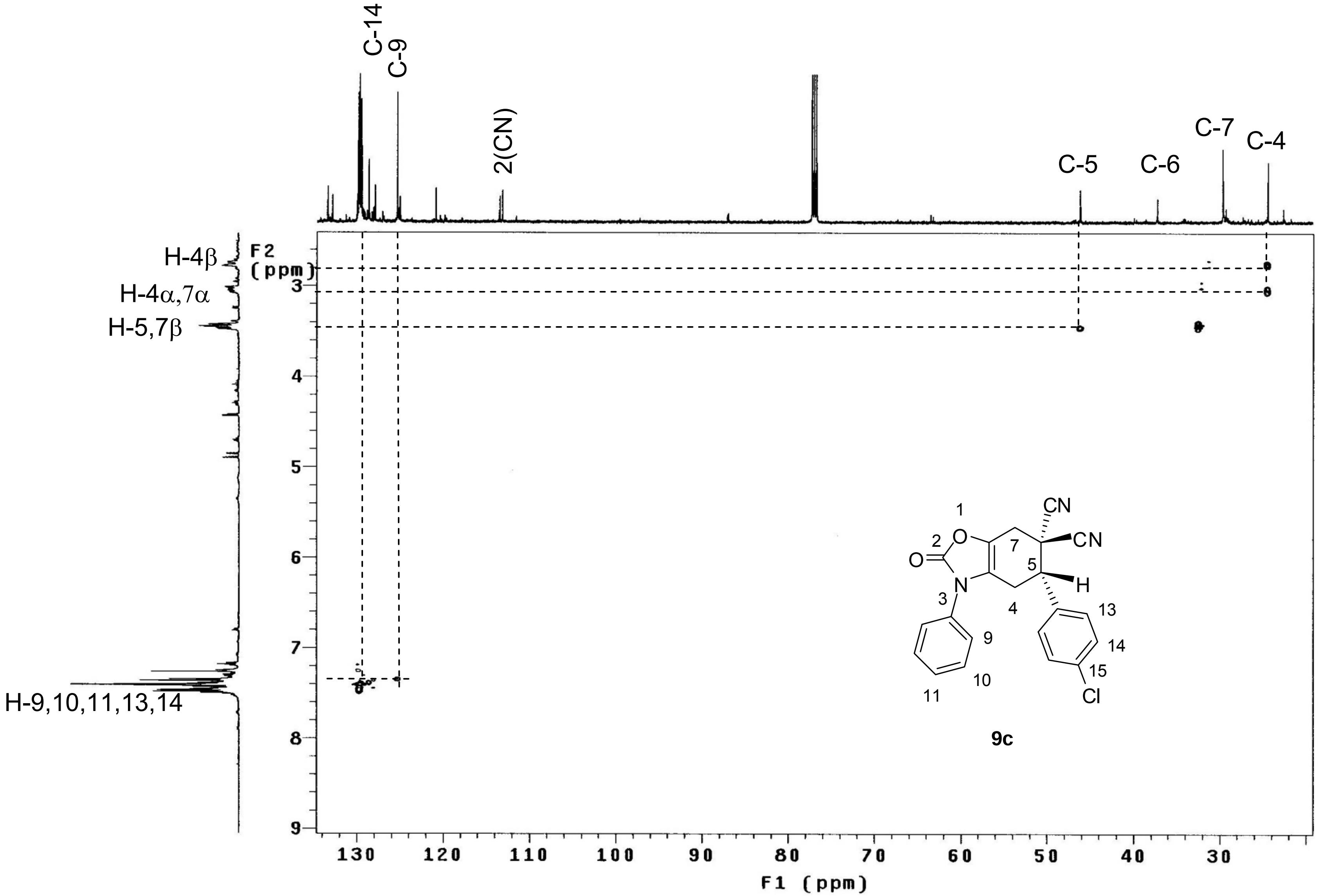

HSQC experiment ($CDCl_3$) **9c**

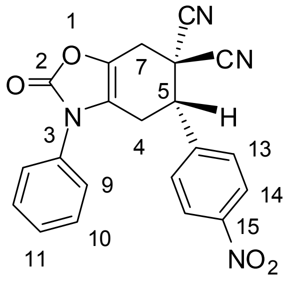

9d

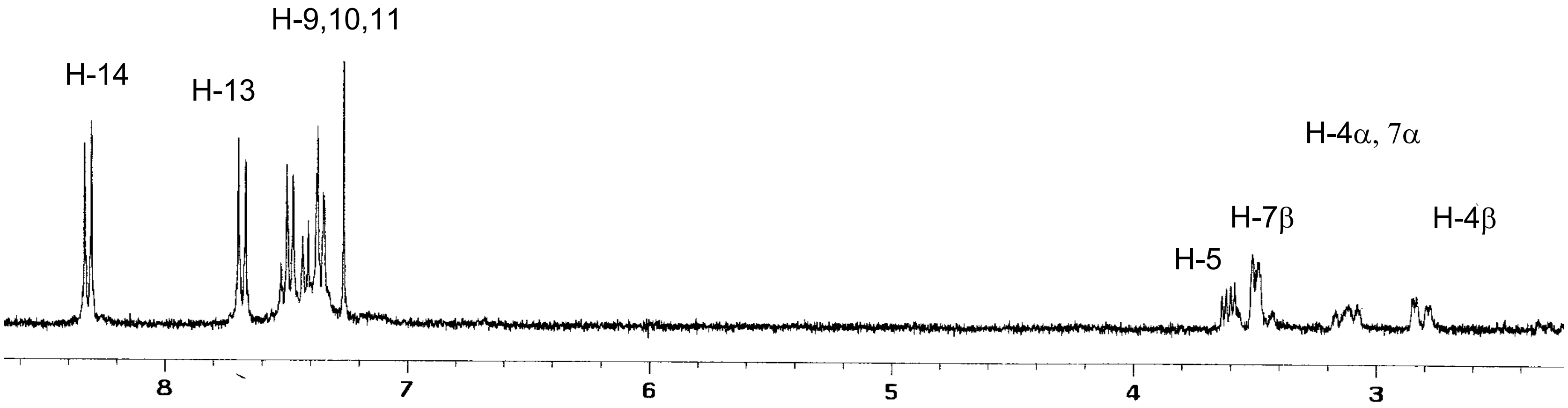

^{1}H NMR ($CDCl_3$) **9d**

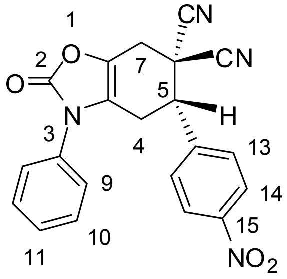

9d

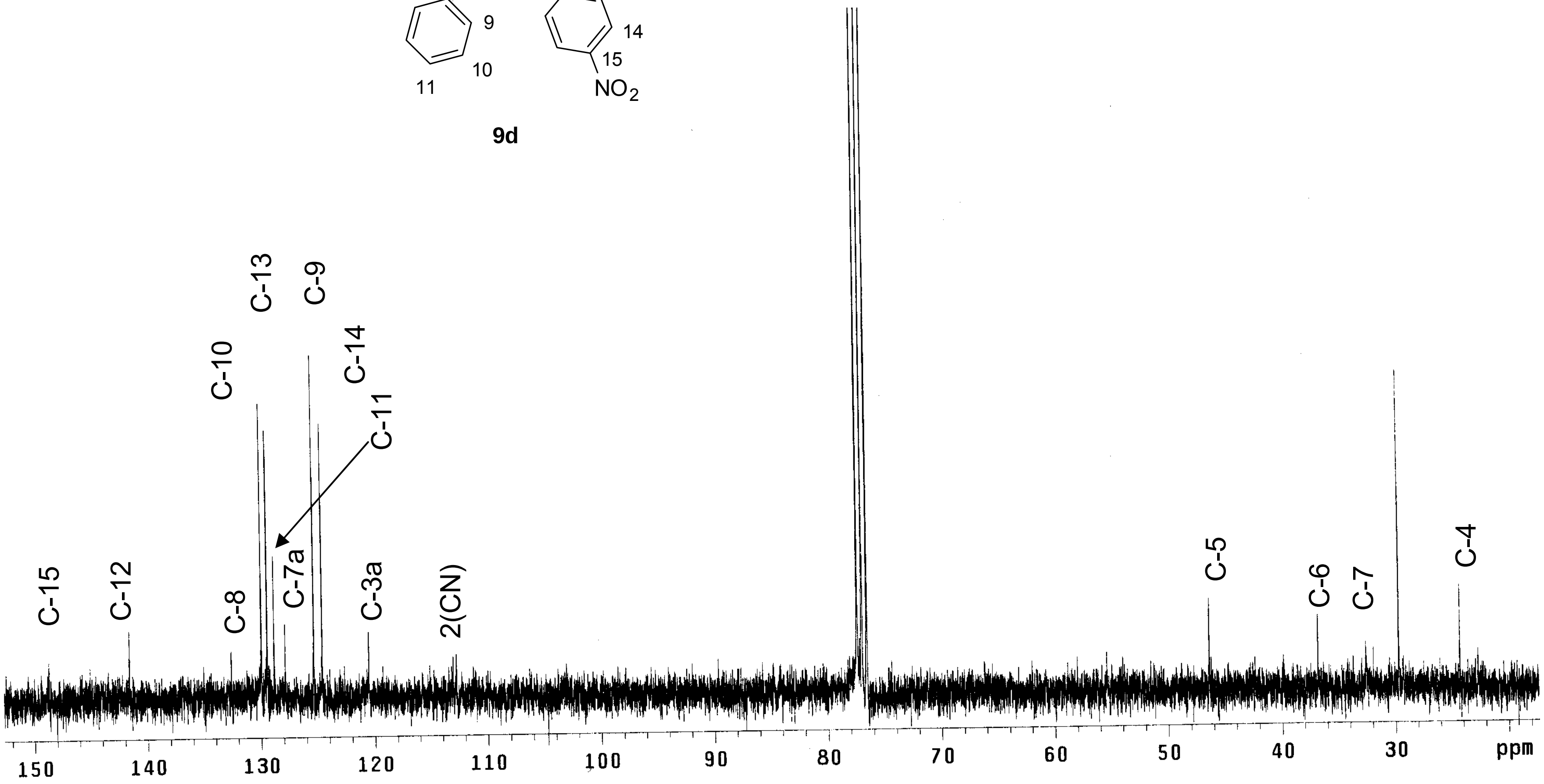

^{13}C NMR (CDCl$_3$) **9d**

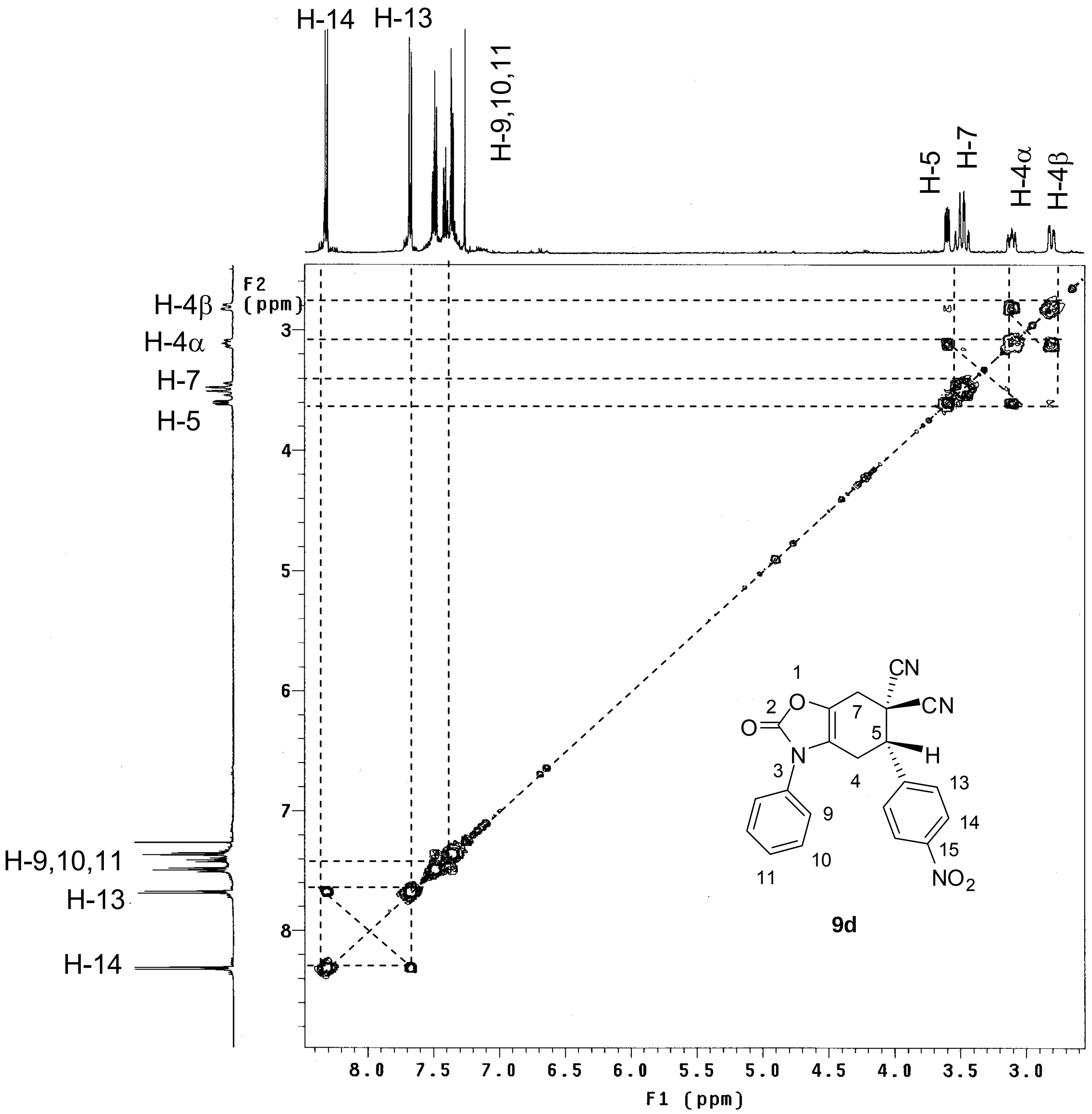

COSY experiment ($CDCl_3$) **9d**

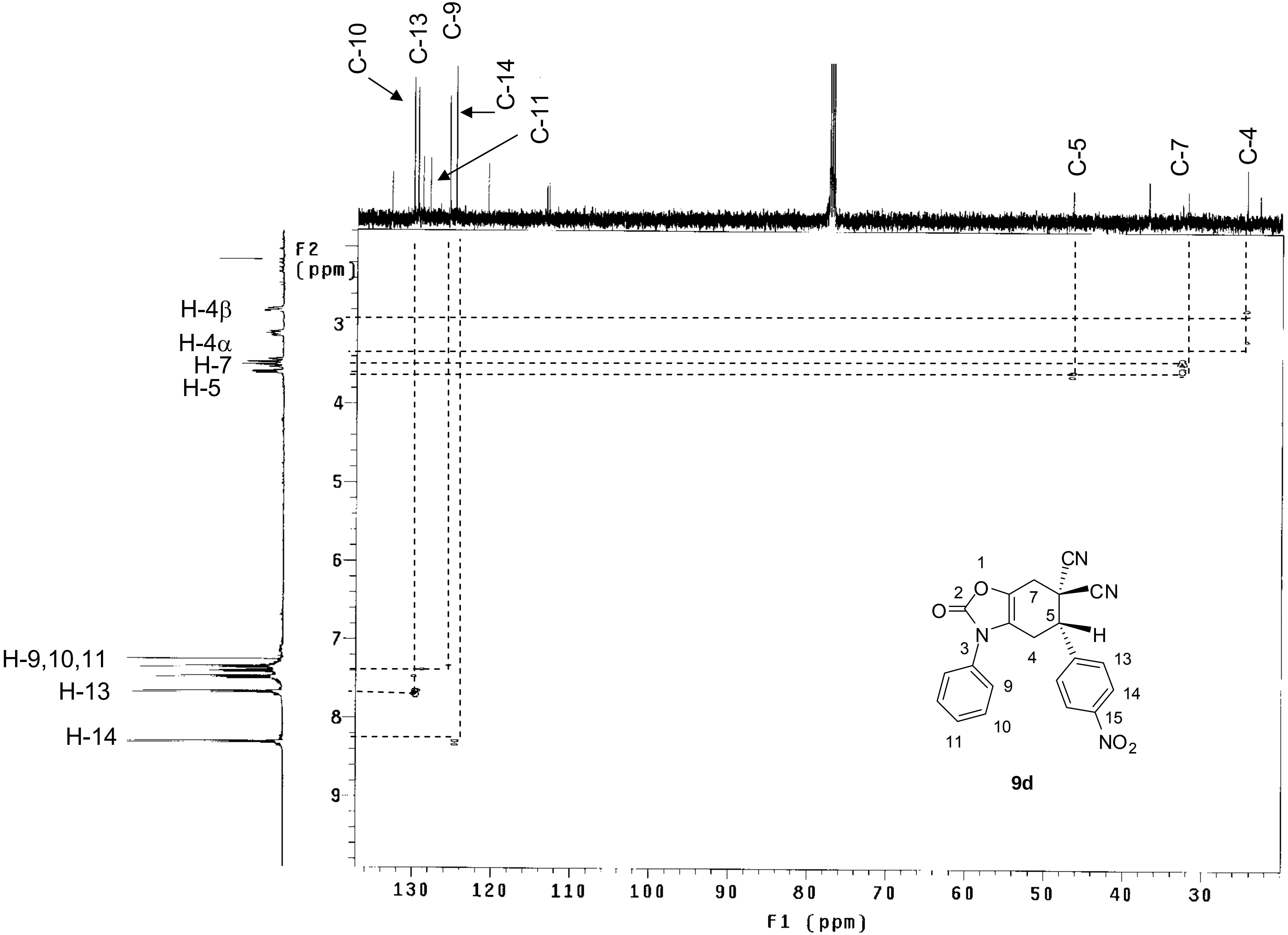

HSQC experiment ($CDCl_3$) **9d**

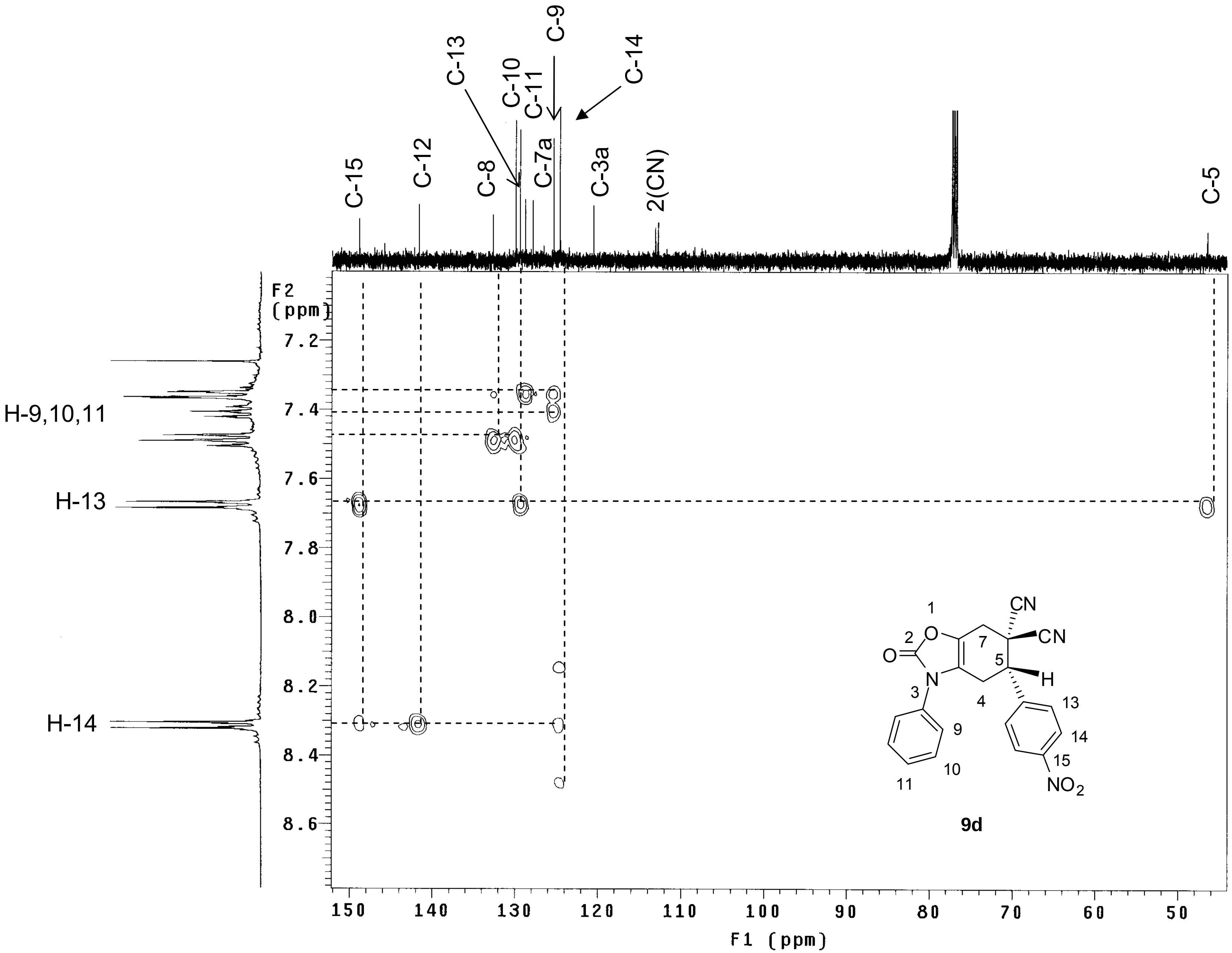

HMBC experiment ($CDCl_3$) **9d**

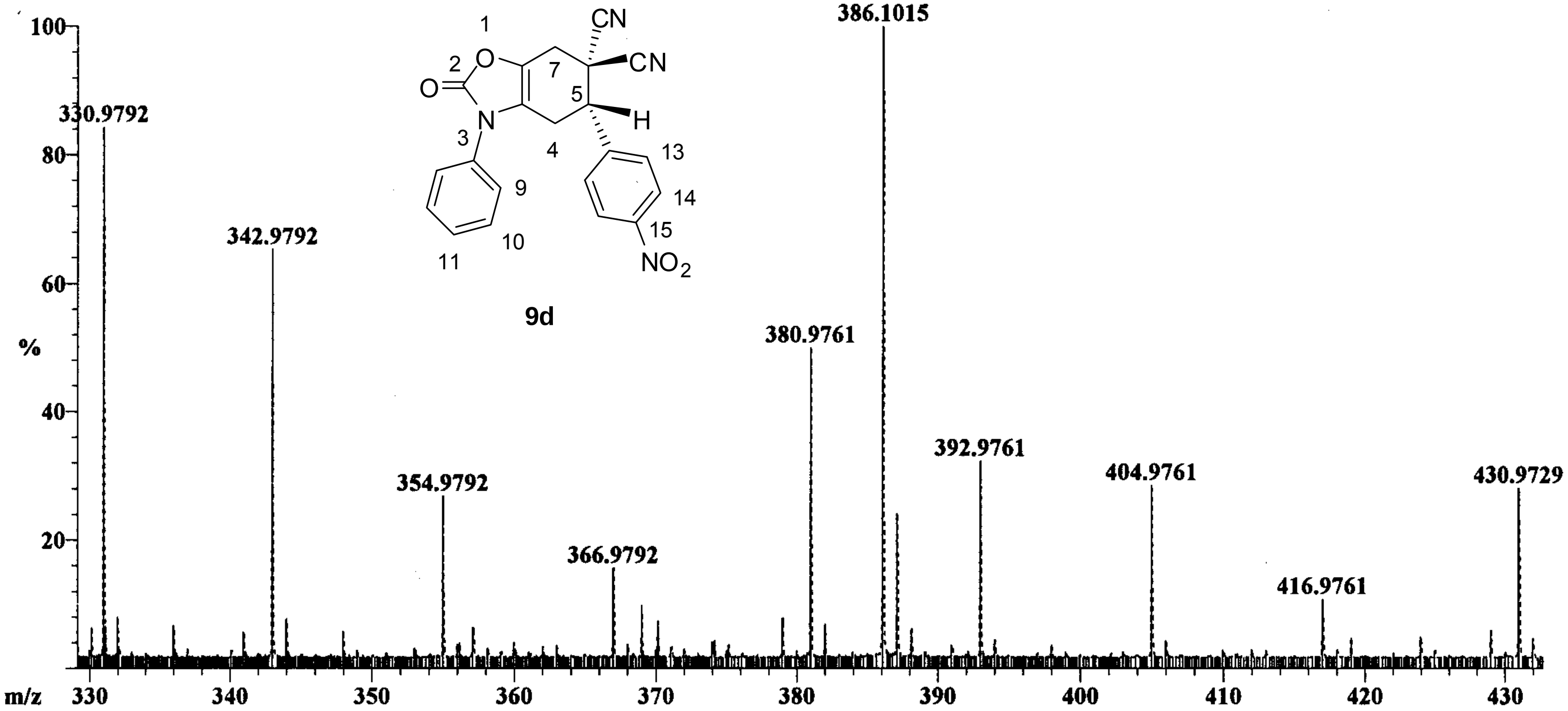

Selected Isotopes : $N_{0\text{-}4}O_{0\text{-}4}H_{0\text{-}14}C_{0\text{-}21}$

Error Limit : 10 mmu

Measured Mass	*% Base*	*Formula*	*Calculated Mass*	*Error*
386.1015	100.0%	$C_{21}H_{14}N_4O_4$	386.1015	0.0

HRMS **9d**

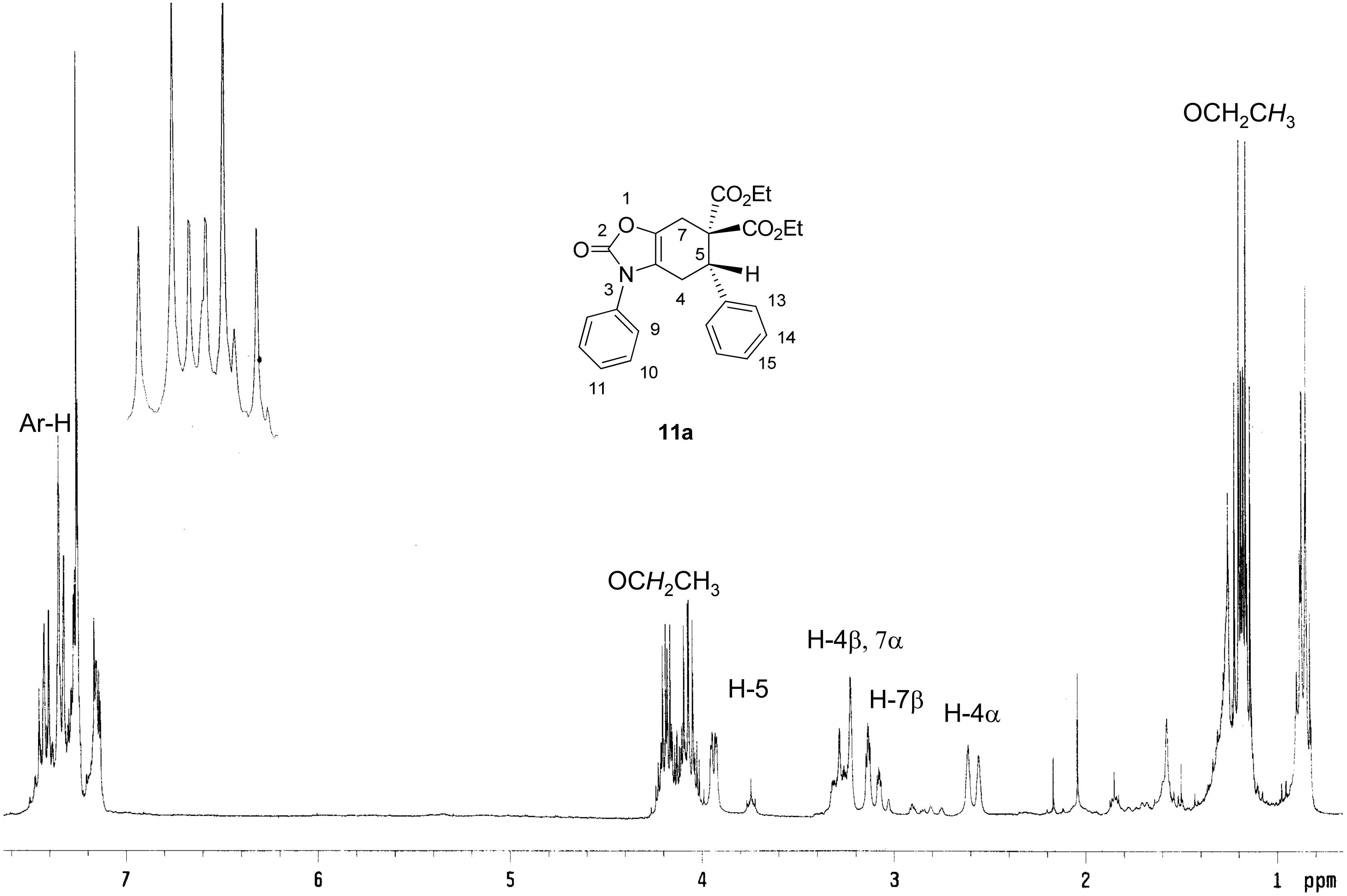

^{1}H NMR (CDCl$_3$) **11a**

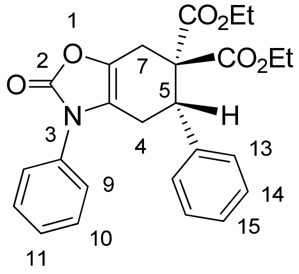

11a

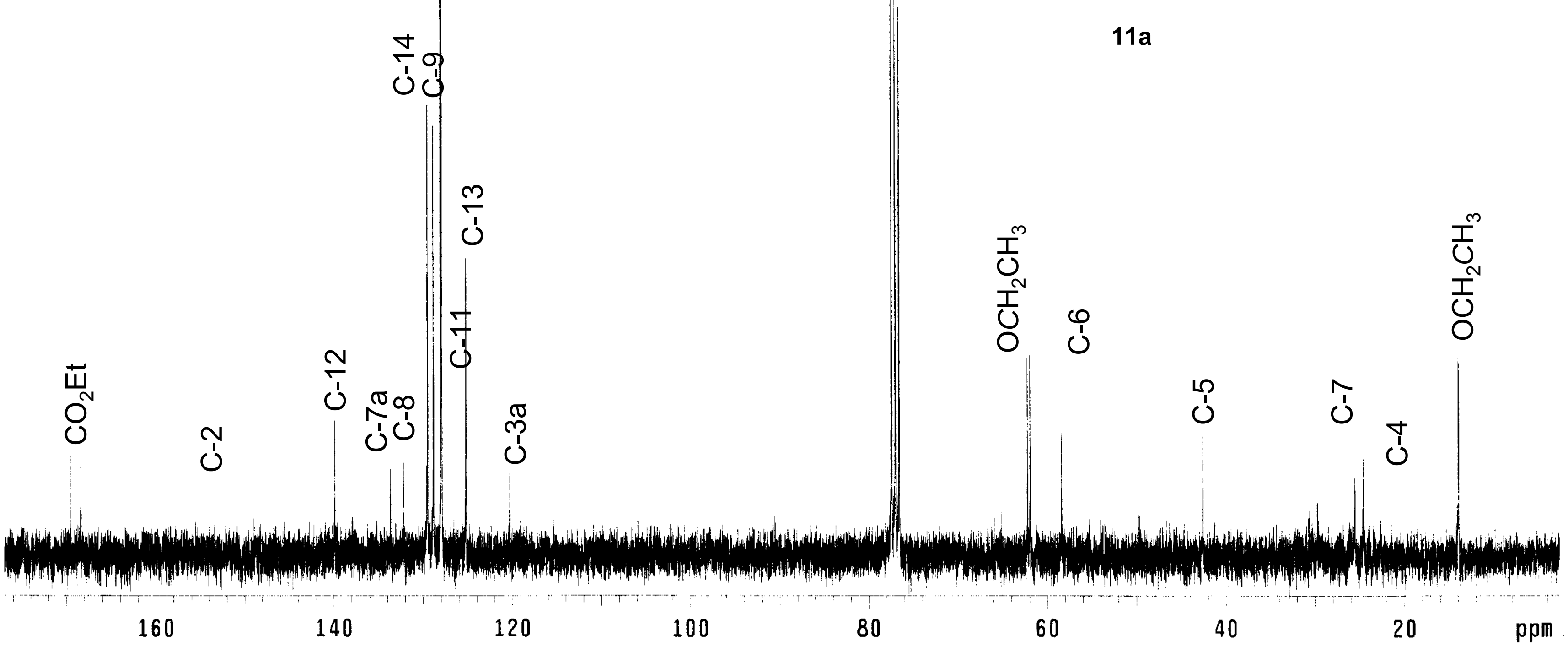

^{13}C NMR ($CDCl_3$) **11a**

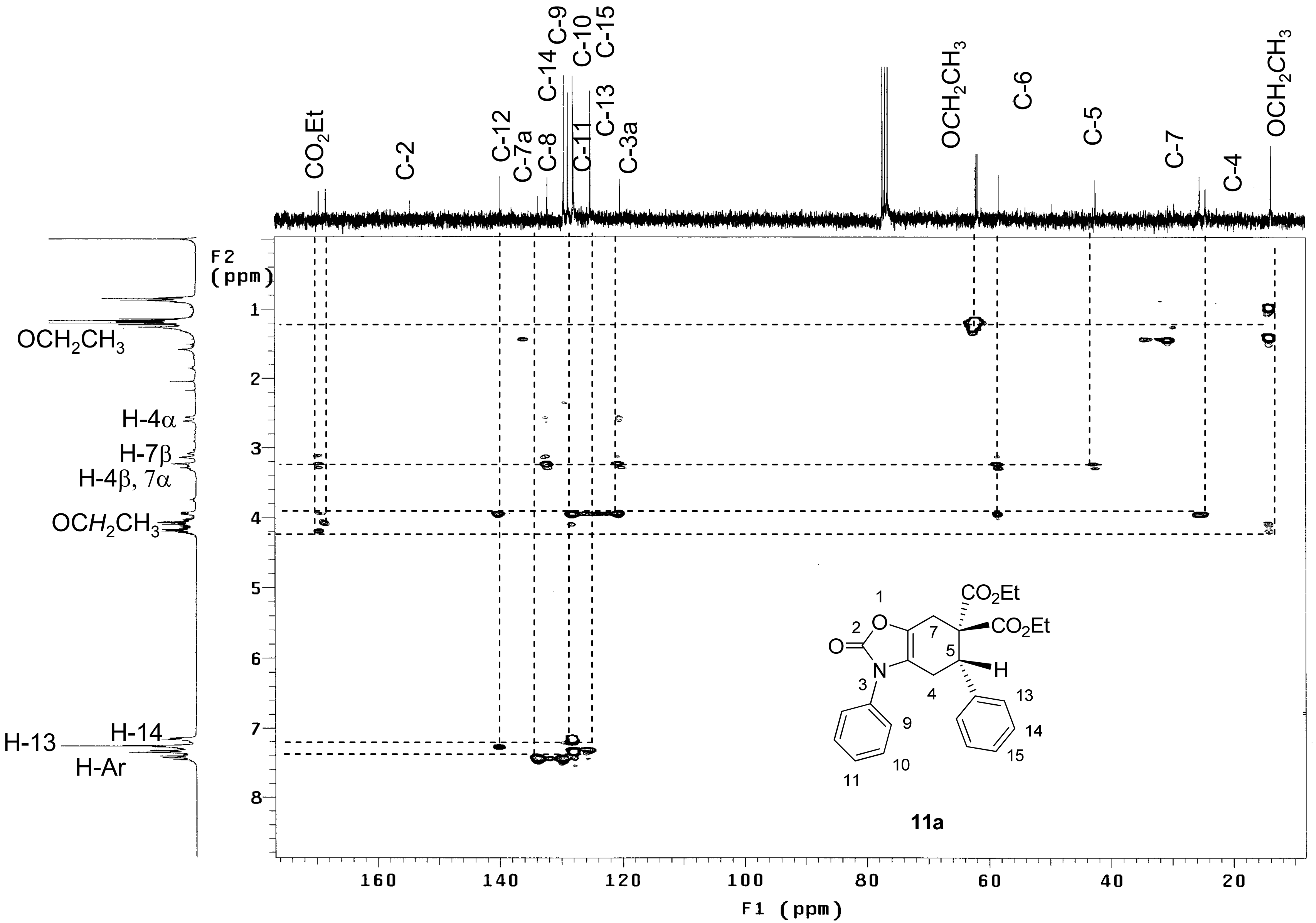

HMBC experiment ($CDCl_3$) **11a**

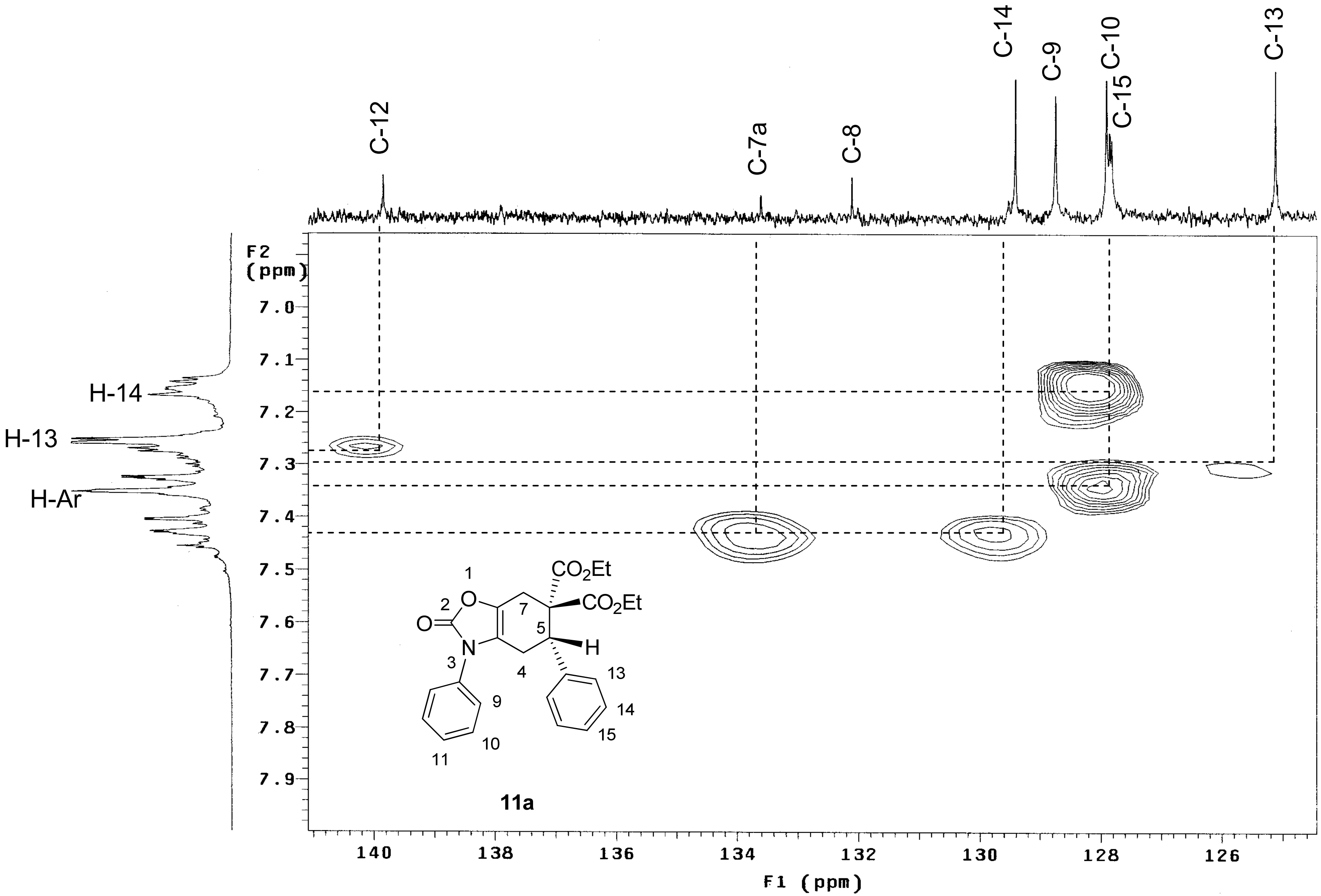

HSQC experiment ($CDCl_3$) **11a**

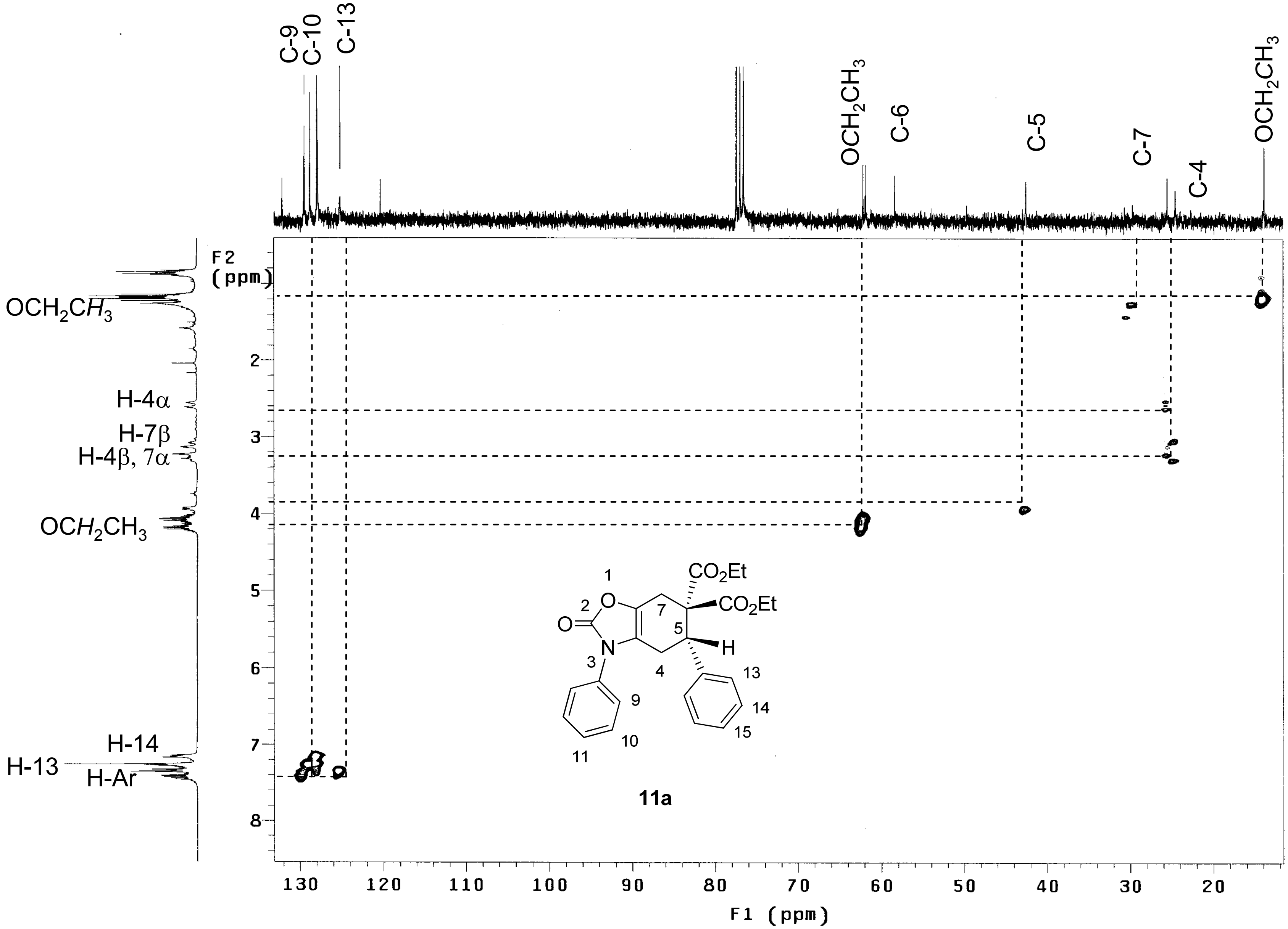

HSQC experiment ($CDCl_3$) **11a**

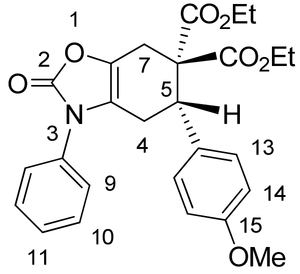

11b

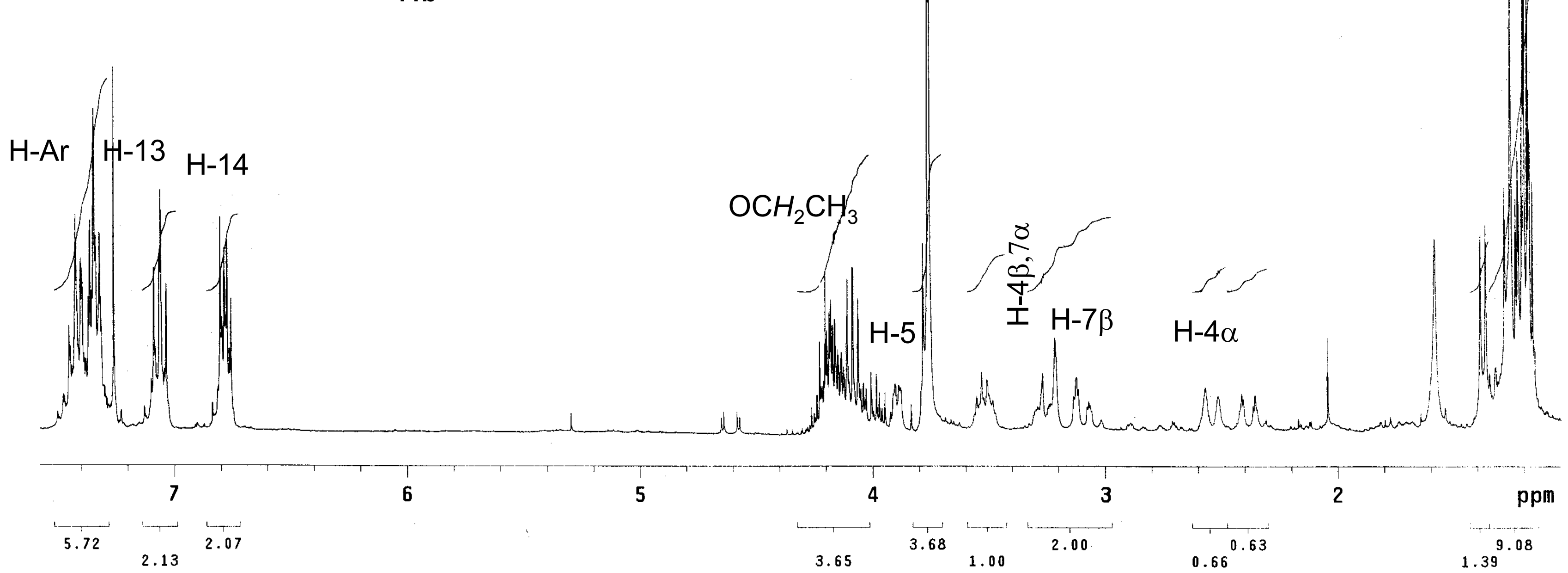

^{1}H NMR ($CDCl_3$) **11b**

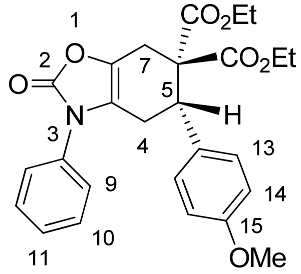

11b

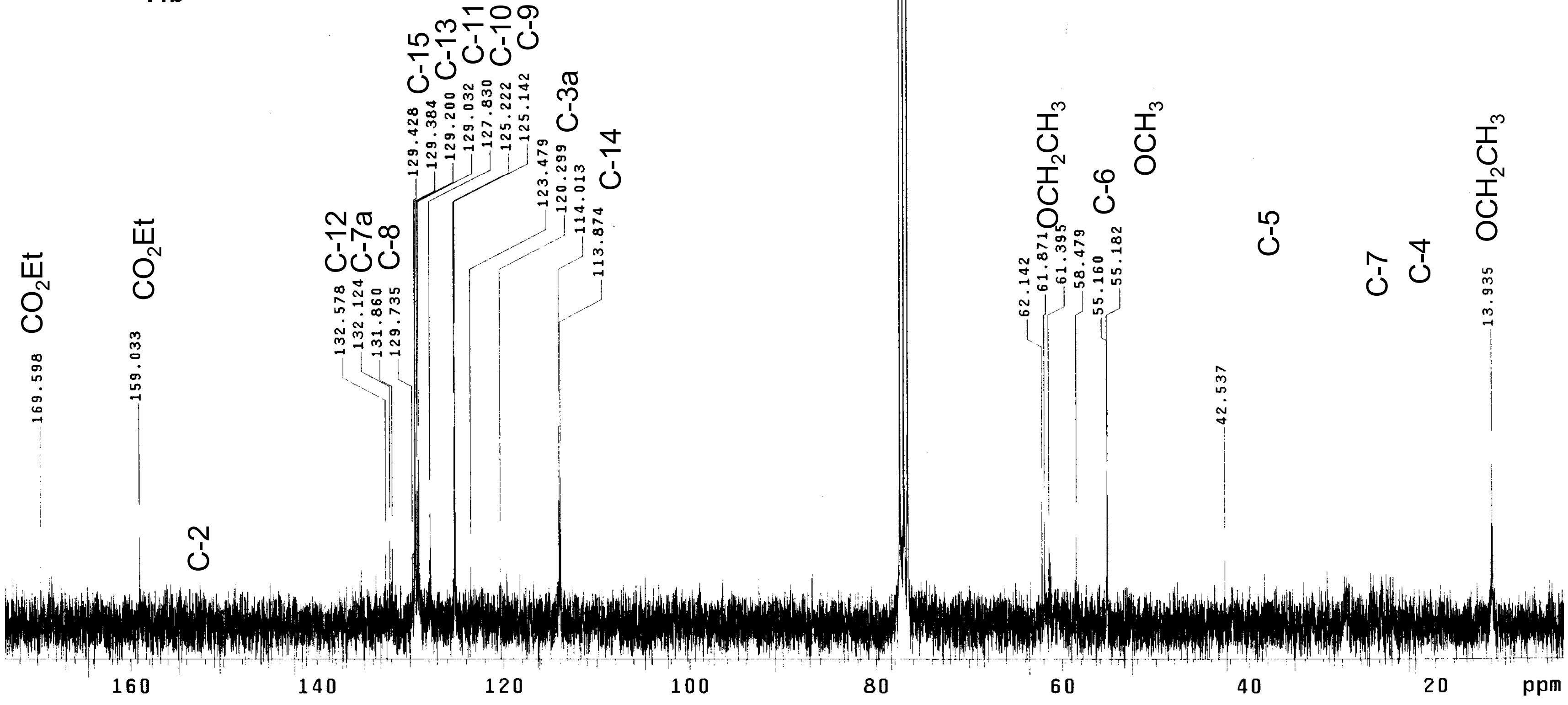

^{13}C NMR ($CDCl_3$) **11b**

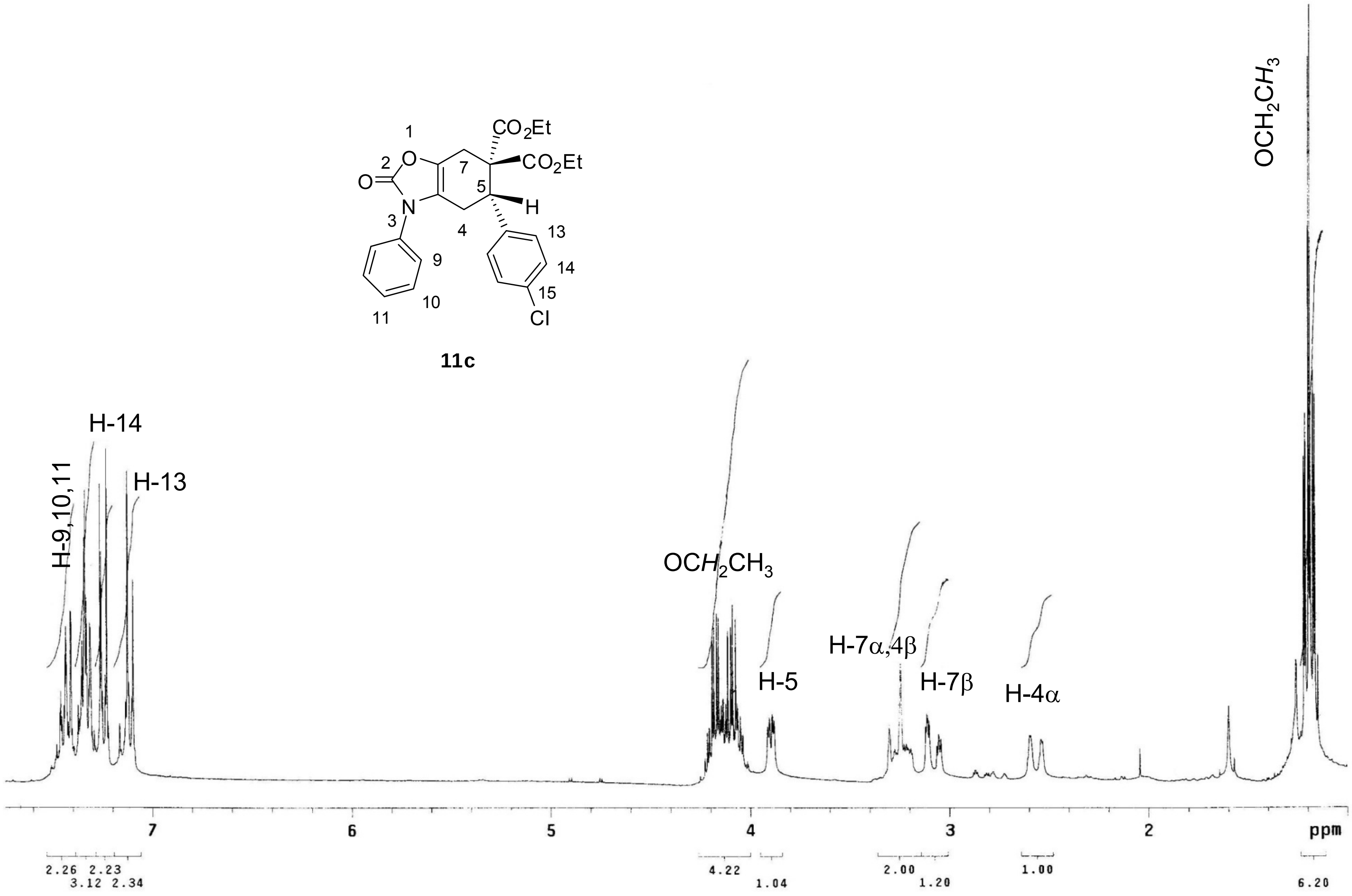

11c

^{1}H NMR ($CDCl_3$) **11c**

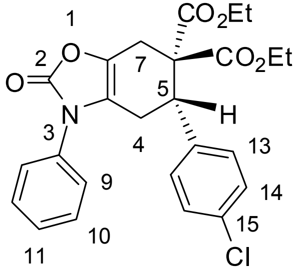

11c

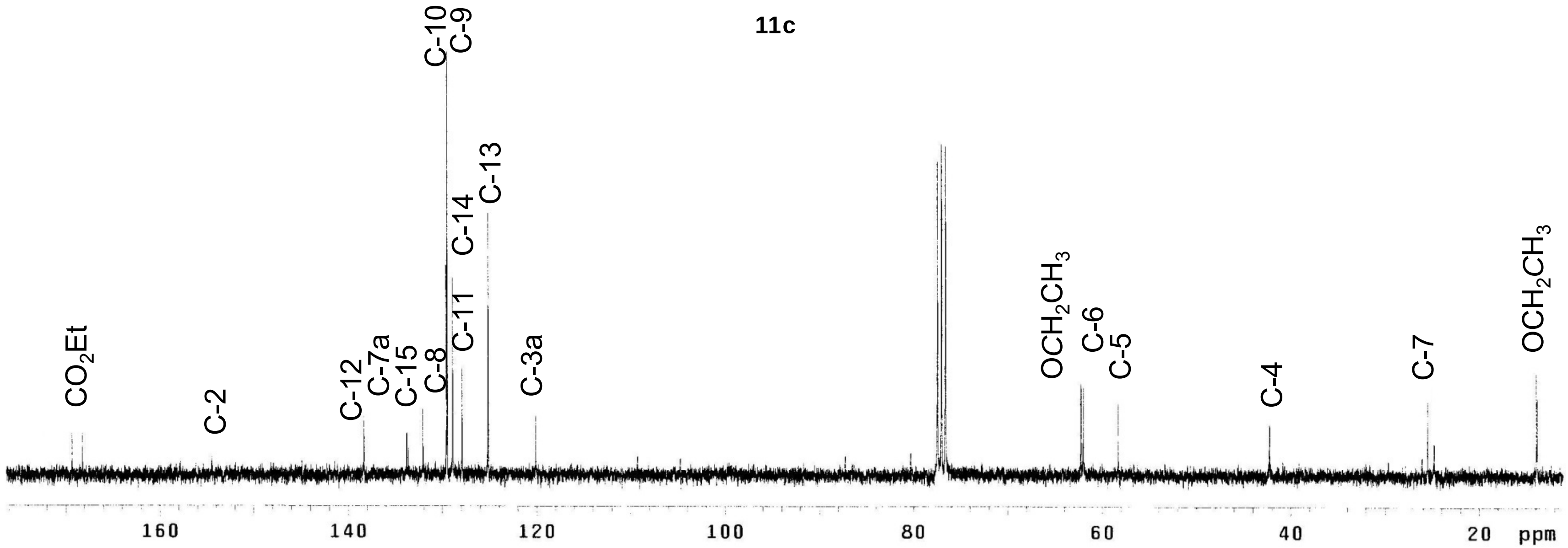

^{13}C NMR ($CDCl_3$) **11c**

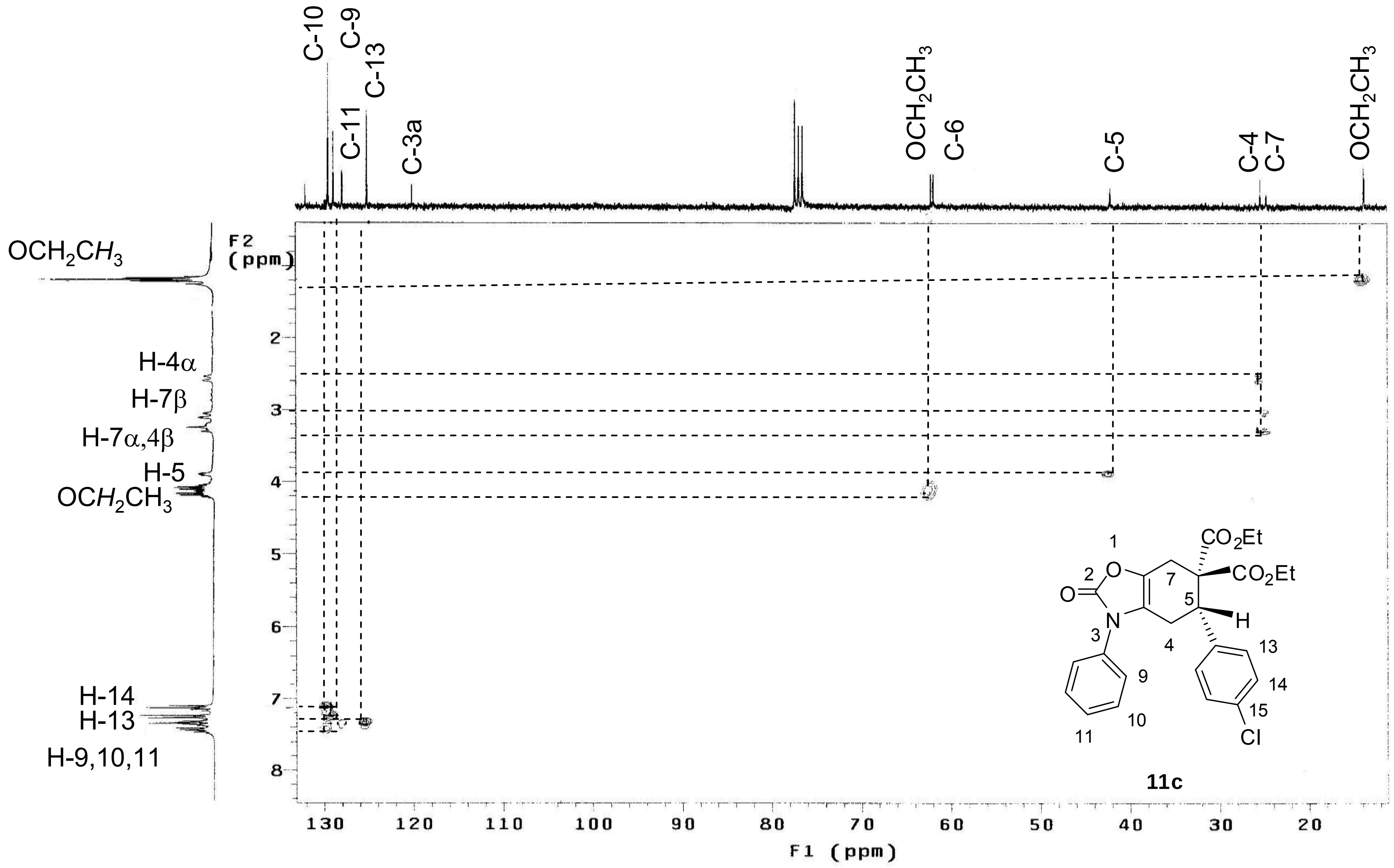

HSQC experiment ($CDCl_3$) **11c**

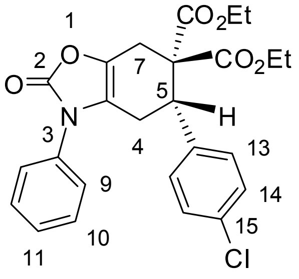

11c

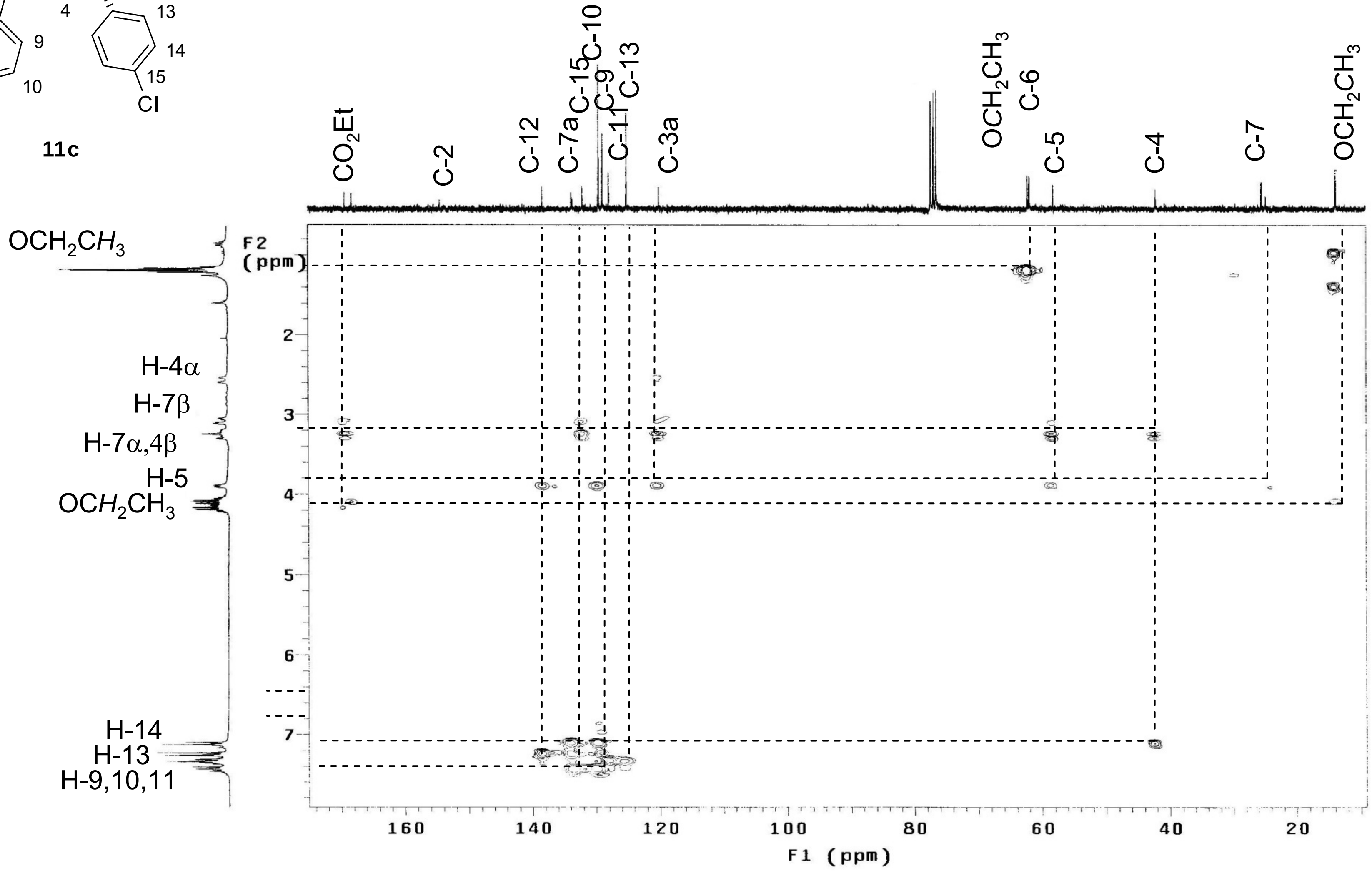

HMBC experiment ($CDCl_3$) **11c**

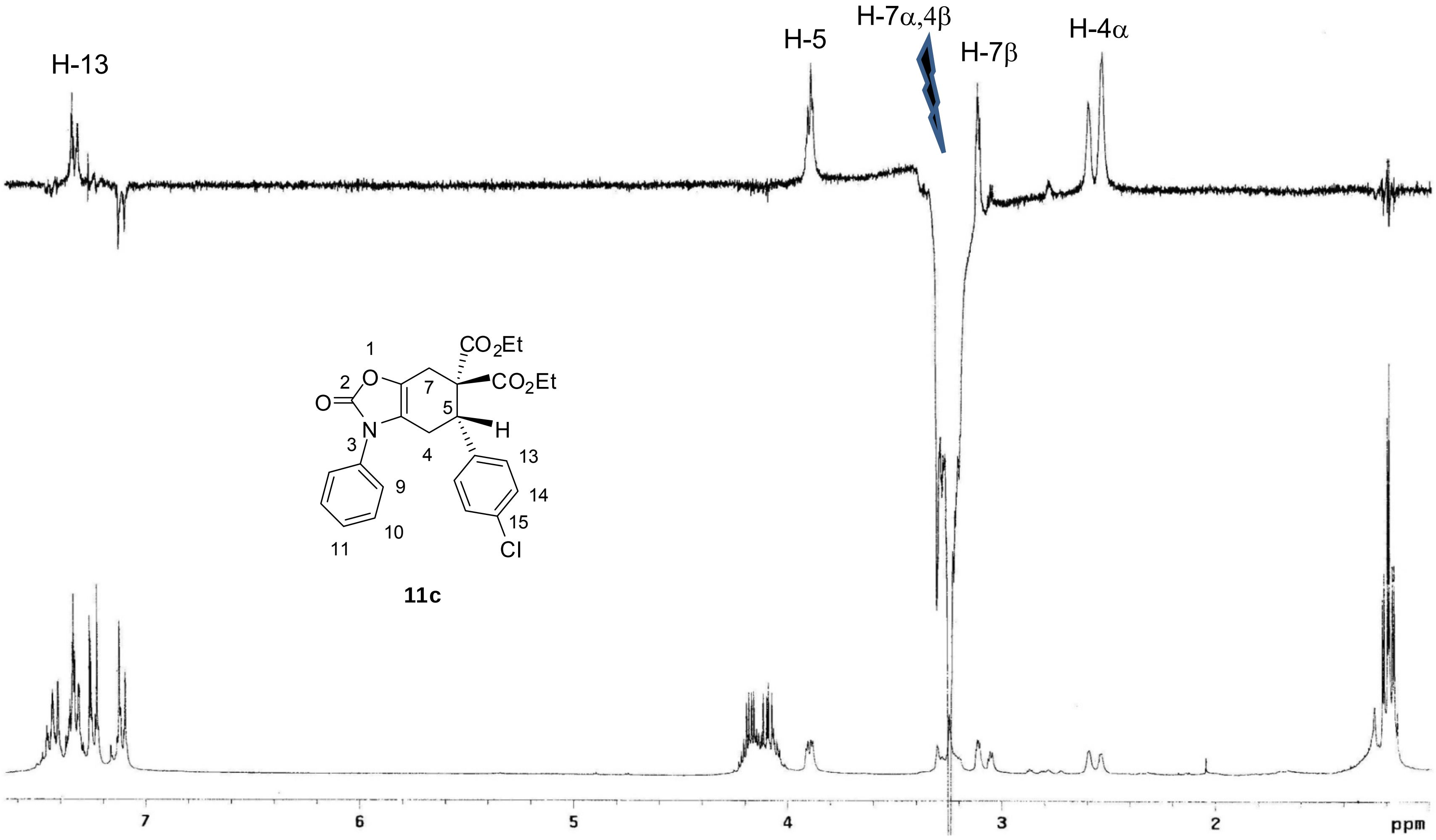

NOE experiment ($CDCl_3$) **11c**

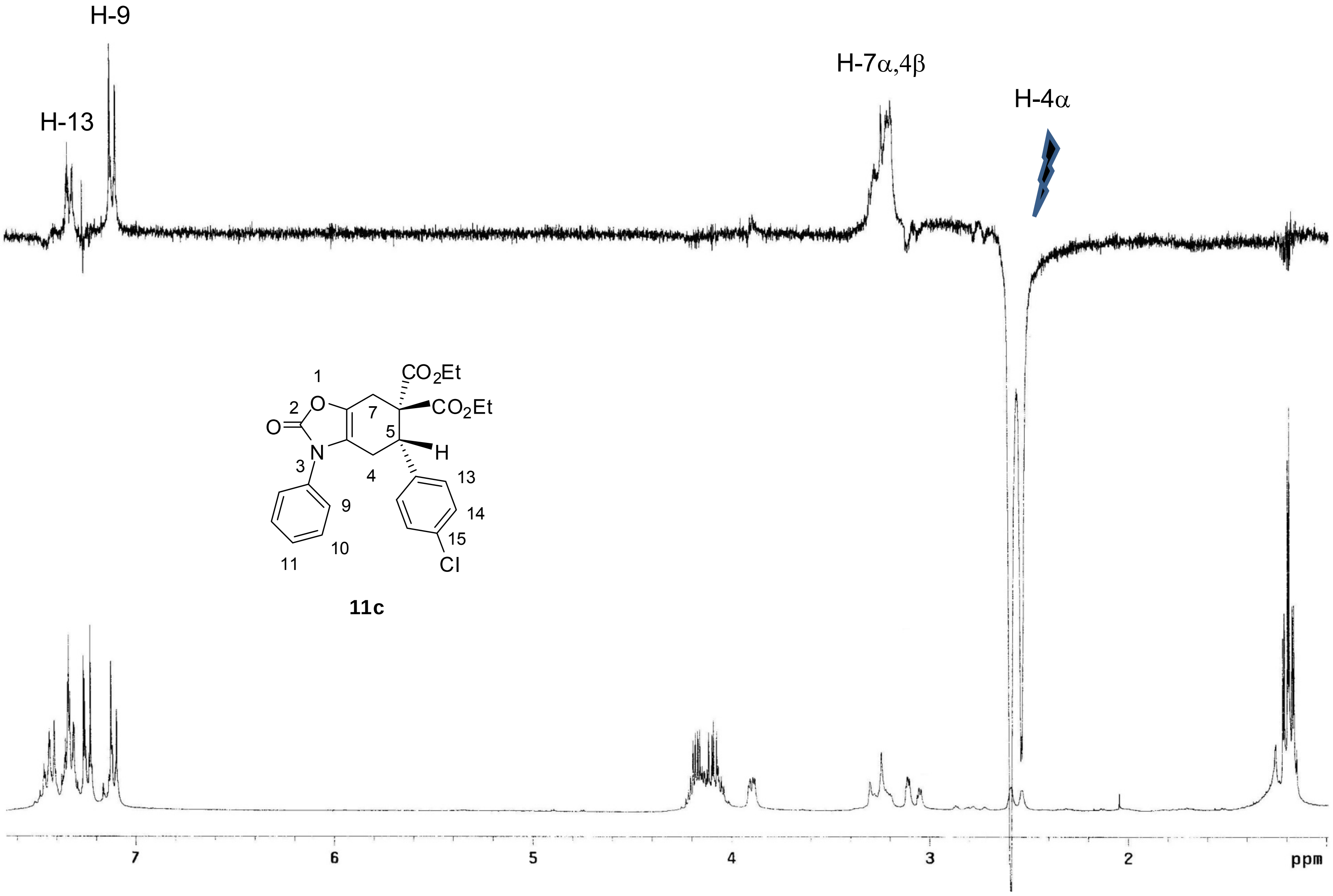

11c

NOE experiment ($CDCl_3$) **11c**

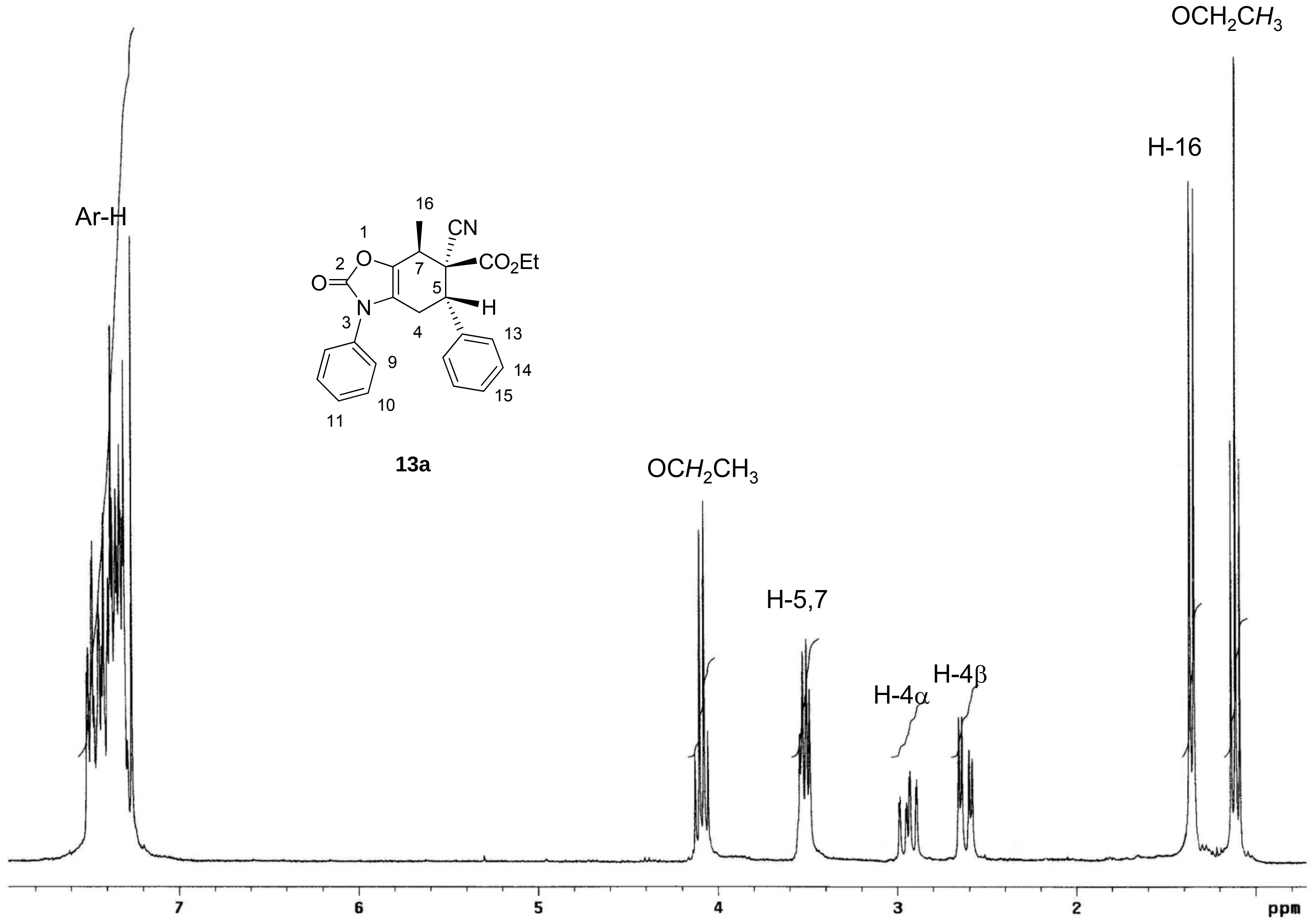

^{1}H NMR ($CDCl_3$) **13a**

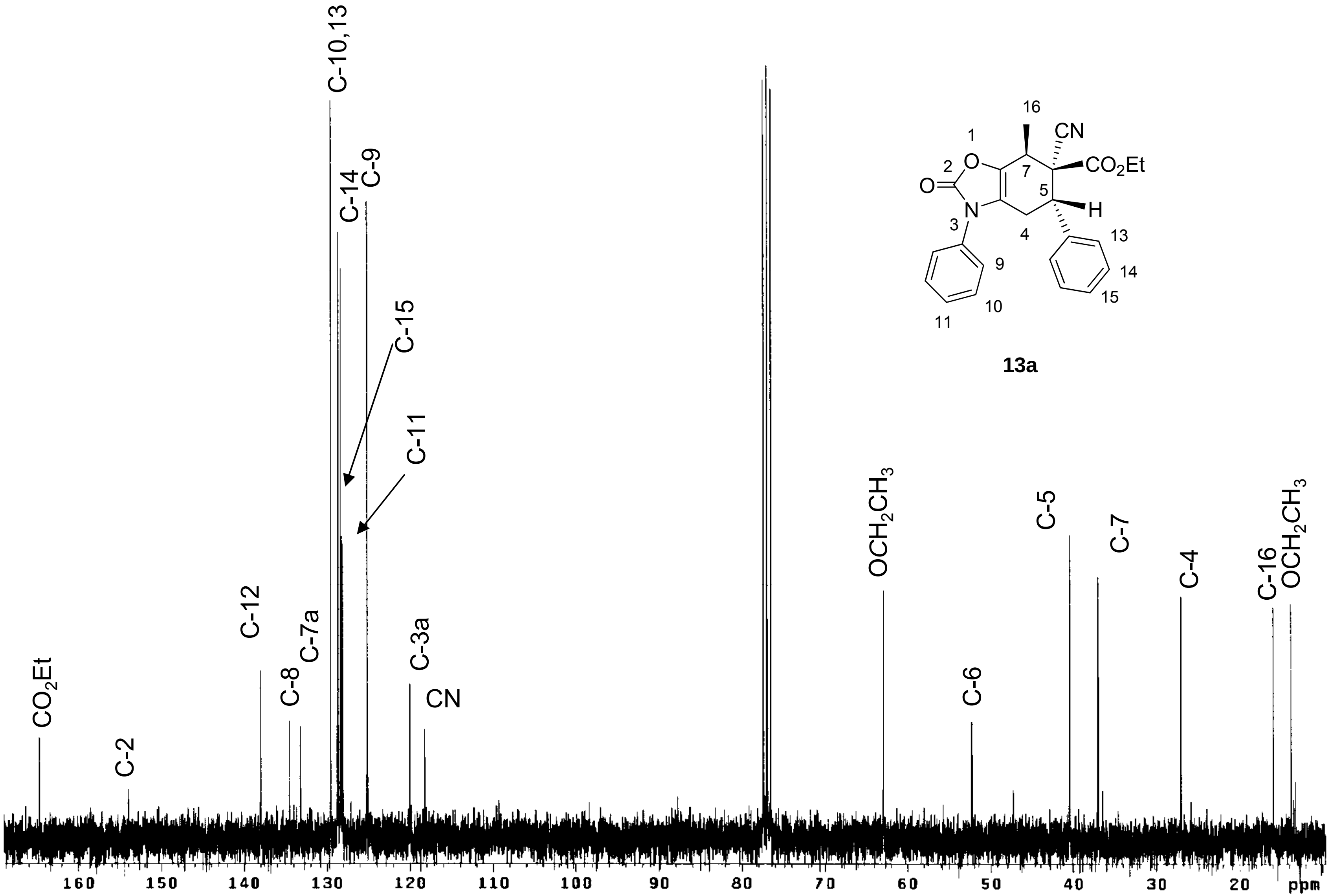

^{13}C NMR ($CDCl_3$) **13a**

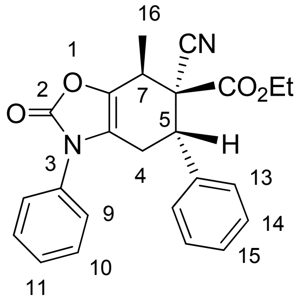

13a

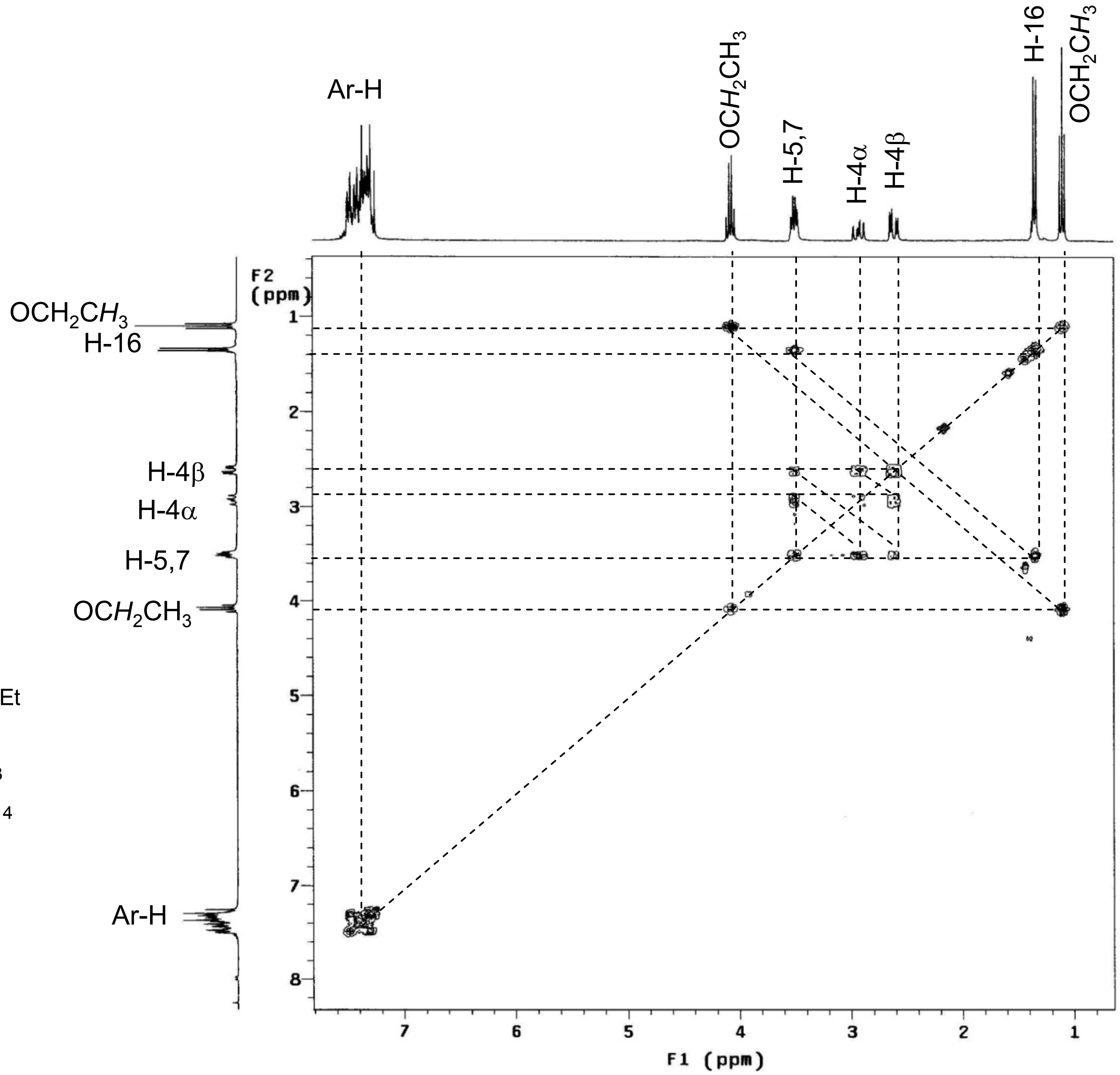

COSY experiment (CDCl$_3$) **13a**

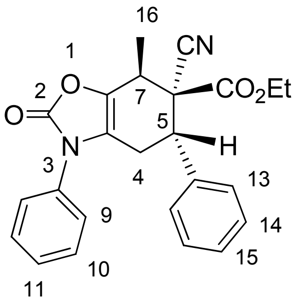

13a

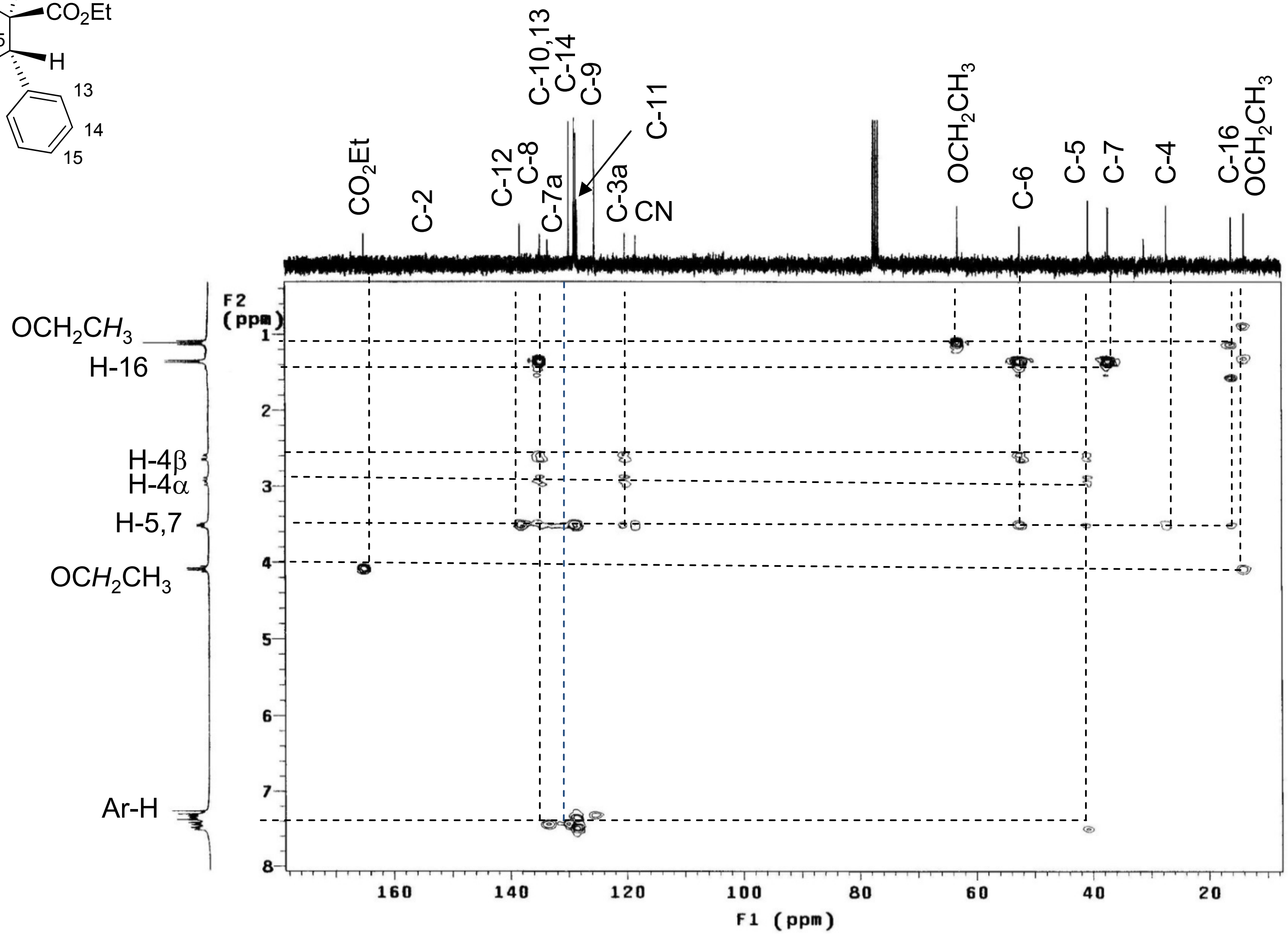

HMBC experiment ($CDCl_3$) **13a**

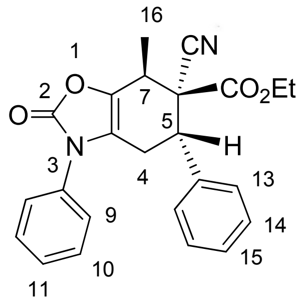

13a

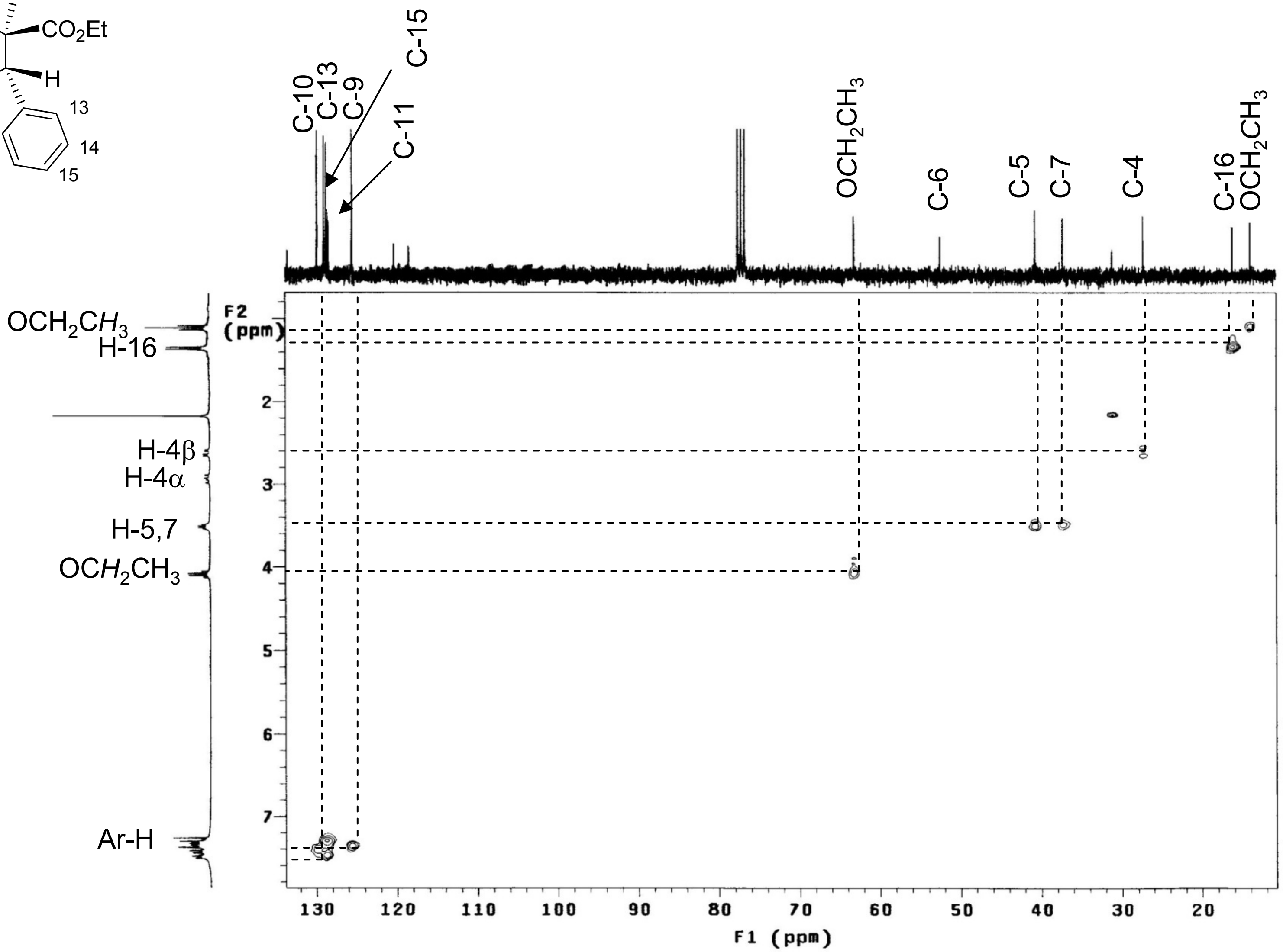

HSQC experiment ($CDCl_3$) **13a**

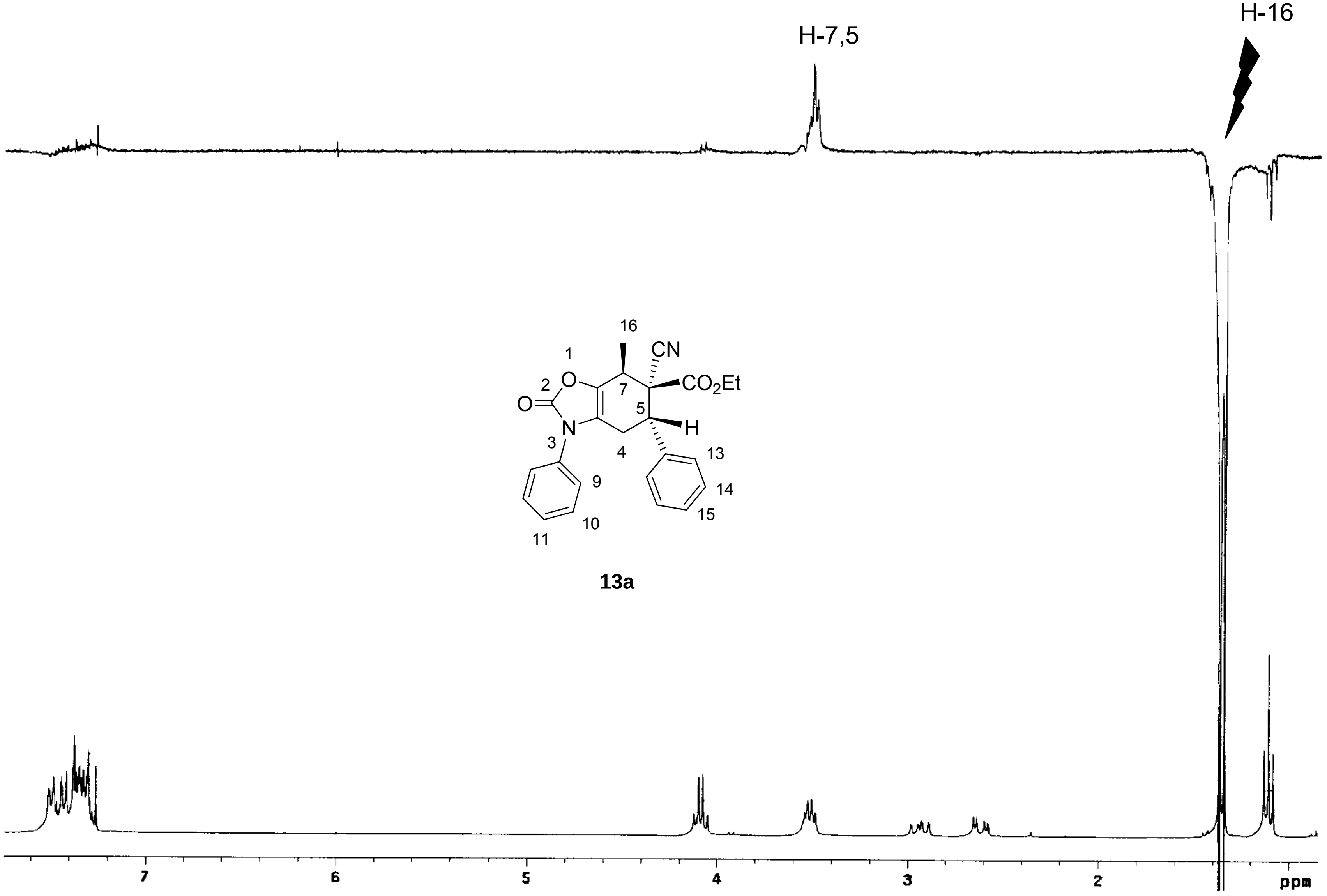

NOE experiment ($CDCl_3$) **13a**

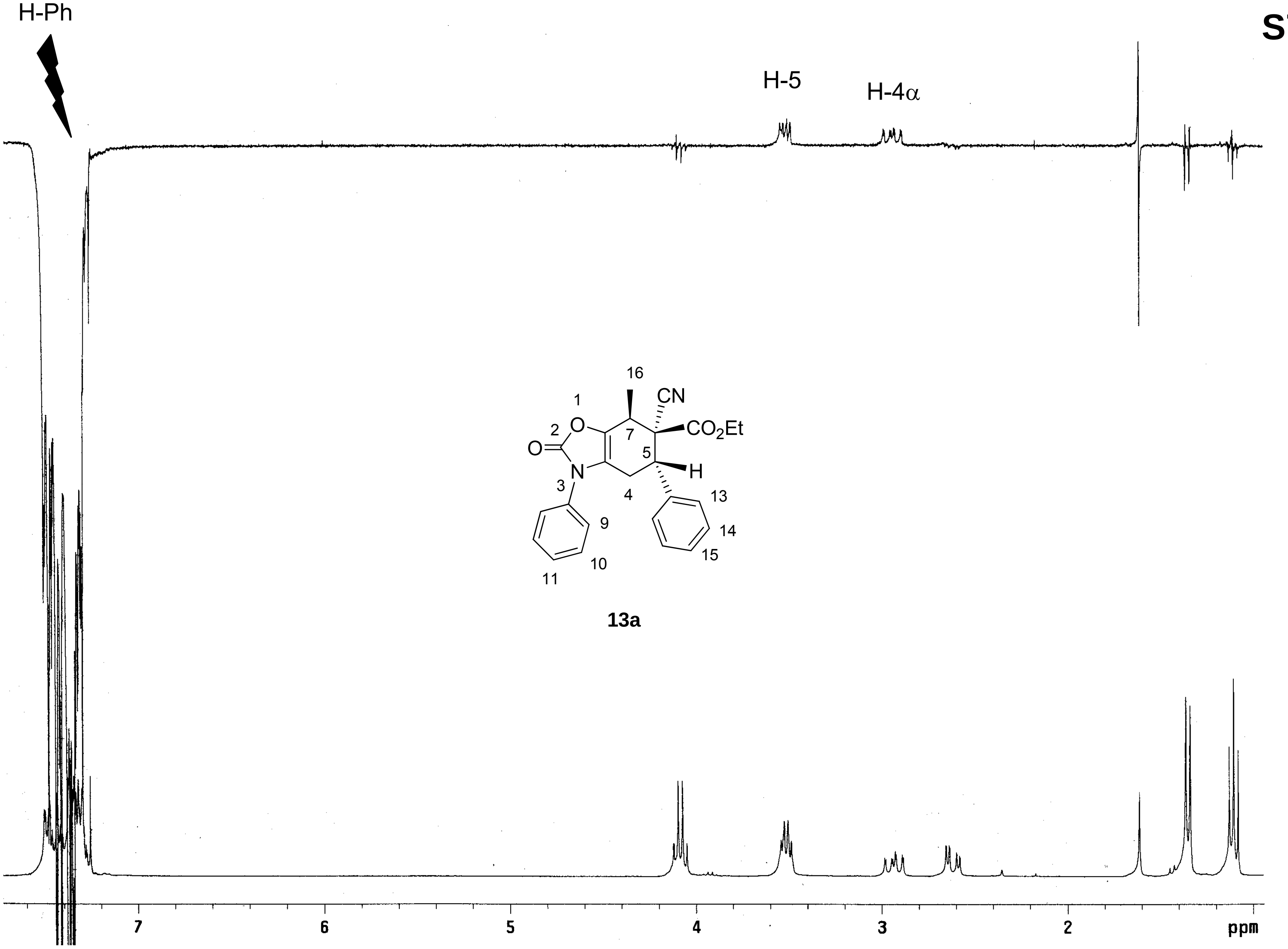

NOE experiment ($CDCl_3$) **13a**

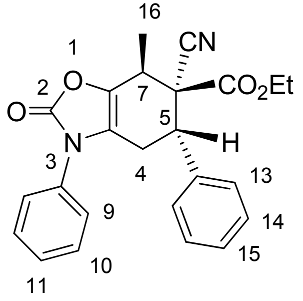

13a

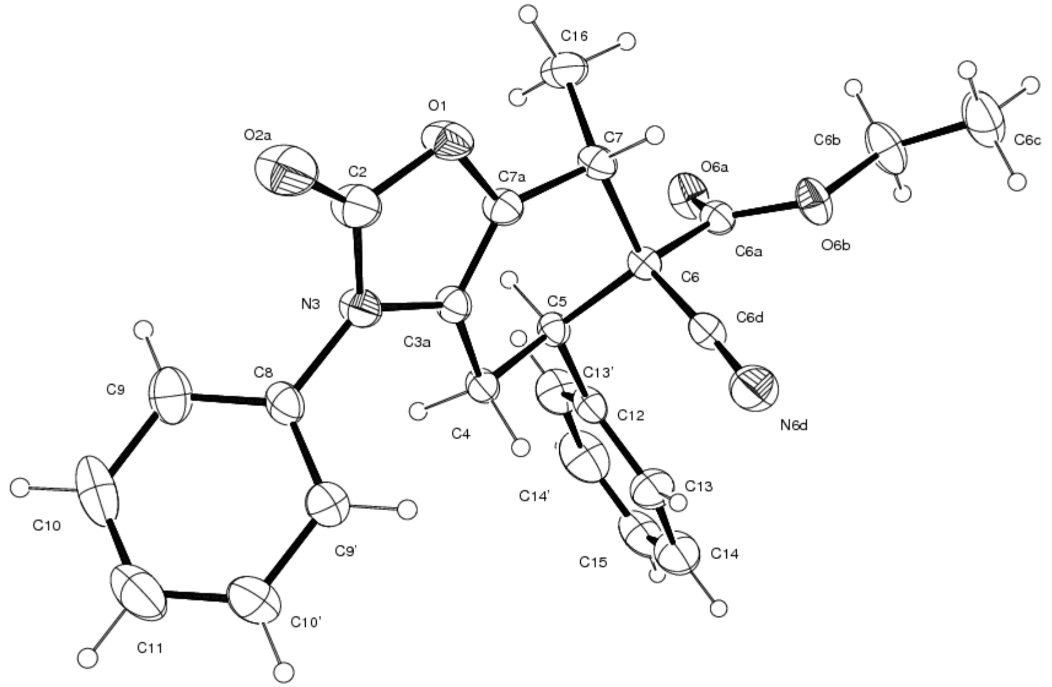

X-Ray structure for **13a**

Table 1. Crystal data and structure refinement for compound **13a**.

Identification code		0004-fdr
Empirical formula		C24 H22 N2 O4
Formula weight		402.44
Temperature		292(2) K
Wavelength		0.71073 Å
Crystal system		Monoclinic
Space group		P 1 21/c 1
Unit cell dimensions	a = 11.8041(2) Å	α= 90°.
	b = 11.5167(3) Å	β= 95.742(2)°.
	c = 16.0207(4) Å	γ = 90°.
Volume		2166.99(9) Å3
Z		4
Density (calculated)		1.234 Mg/m3
Absorption coefficient		0.085 mm-1
F(000)		848
Crystal size		0.24 x 0.18 x 0.14 mm3
Theta range for data collection		2.48 to 32.53°.
Index ranges		-17<=h<=17, -17<=k<=15, -23<=l<=19
Reflections collected		19540
Independent reflections		7117 [R(int) = 0.0417]
Completeness to theta = 27.50°		99.5 %

Max. and min. transmission	0.9882 and 0.9800
Refinement method	Full-matrix least-squares on F^2
Data / restraints / parameters	7117 / 0 / 273
Goodness-of-fit on F^2	0.936
Final R indices [I>2sigma(I)]	R1 = 0.0641, wR2 = 0.1335
R indices (all data)	R1 = 0.1531, wR2 = 0.1536
Largest diff. peak and hole	0.266 and -0.148 e.Å-3

File: Ines 402 ID IE MI2Date Run: 02-01-2008 (Time Run: 12:20:06)
Sample: Ines MI2
Instrument: JEOL GCmate
Inlet: Direct Probe **Ionization mode: EI+**

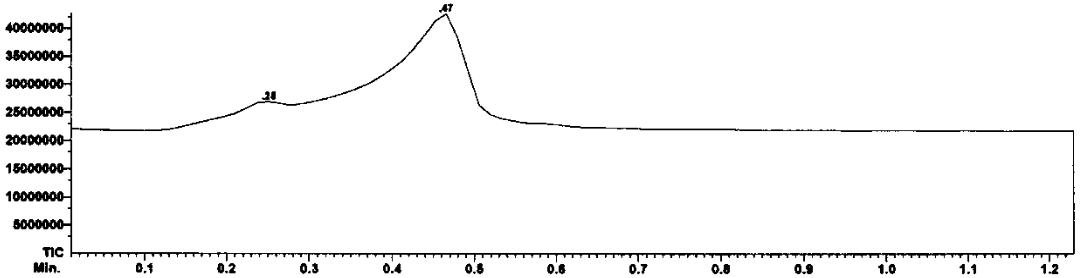

Scan: 33-36 **R.T.: .46**
Base: m/z 402; 44.6%FS TIC: 1521992 **#Ions: 630**

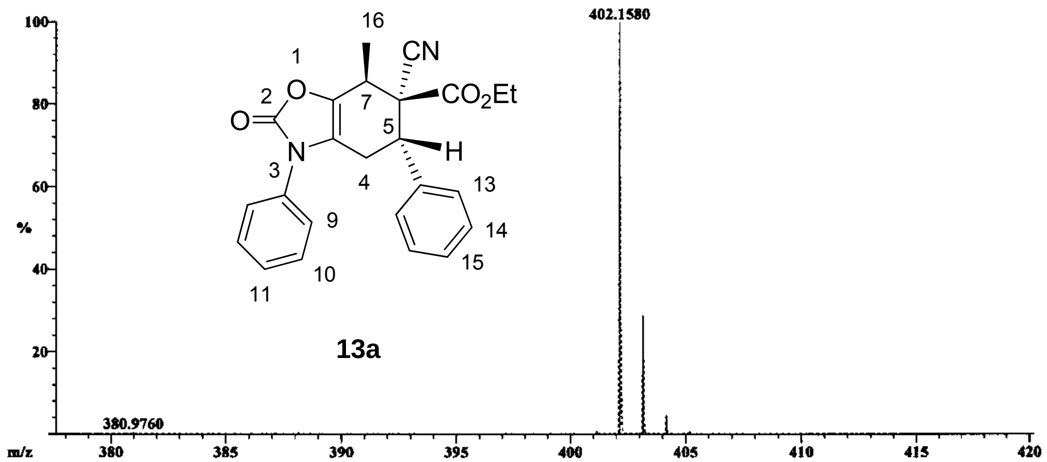

Selected Isotopes : $N_{0-3}O_{0-5}C_{0-26}H_{0-25}$ **Error Limit : 1 mmu**

Measured Mass	% Base	Formula	Calculated Mass	Error
402.15802	100.0%	$C_{24}H_{22}N_2O_4$	402.15796	0.1

HRMS **13a**

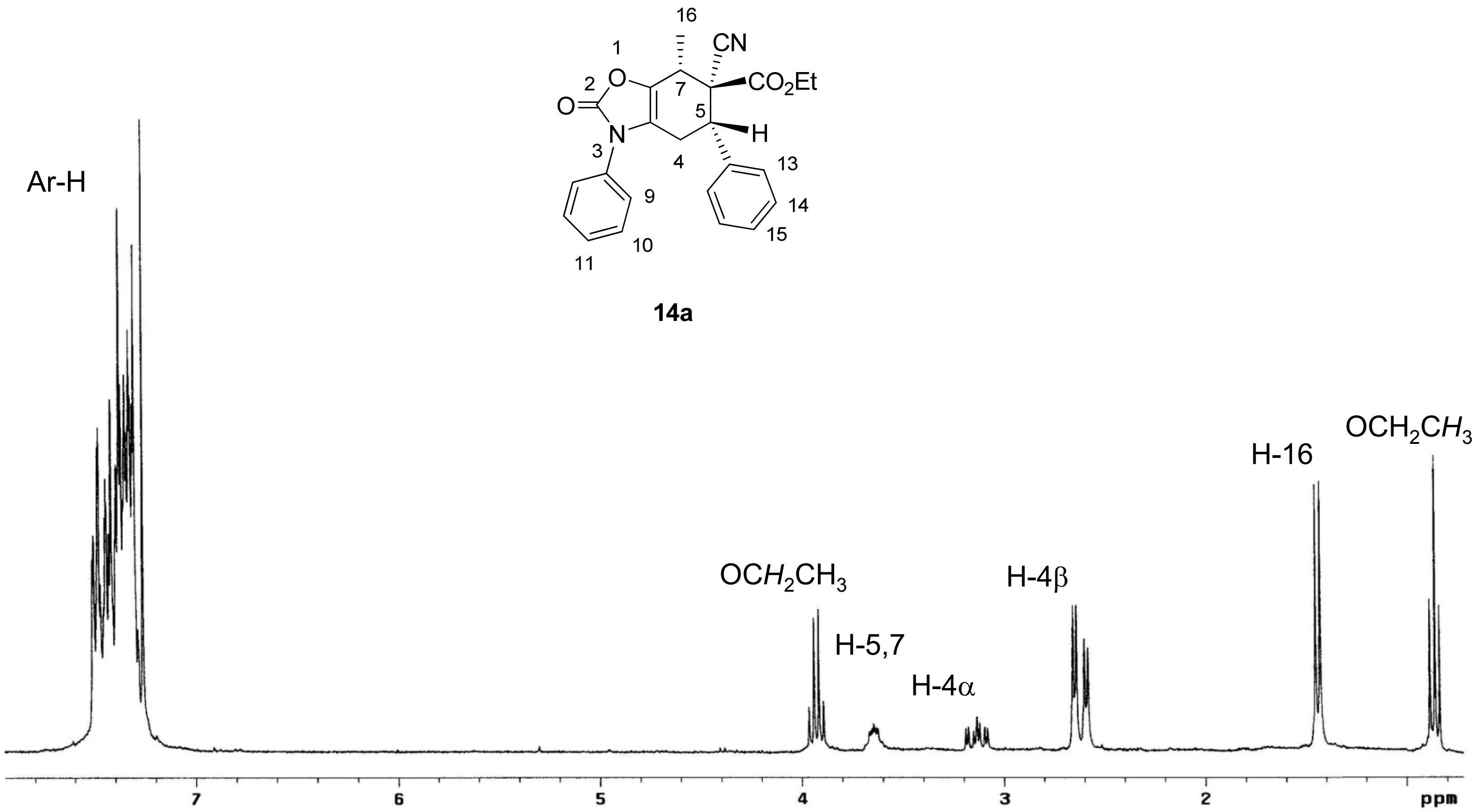

^{1}H NMR ($CDCl_3$) **14a**

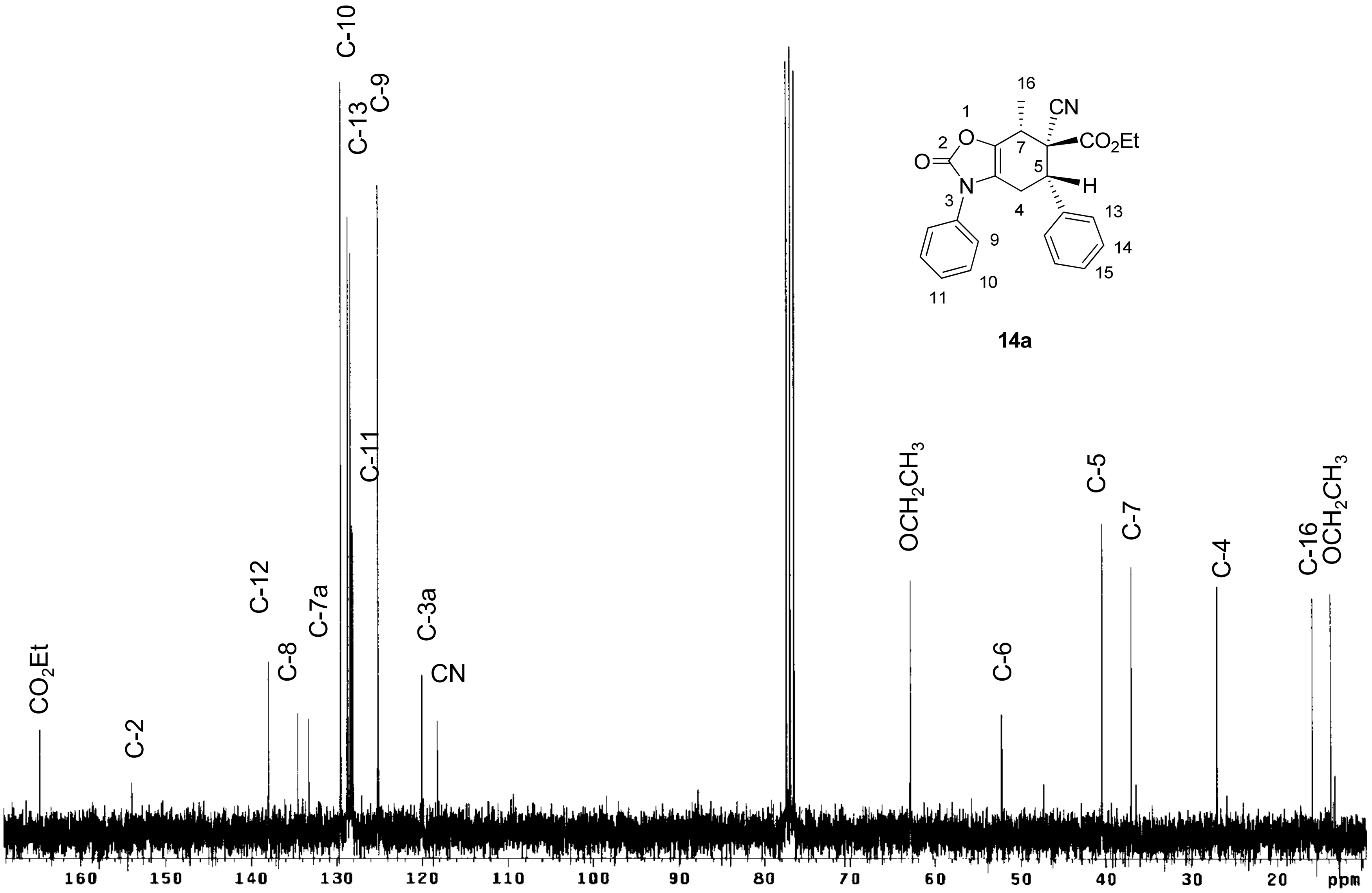

14a

^{13}C NMR ($CDCl_3$) **14a**

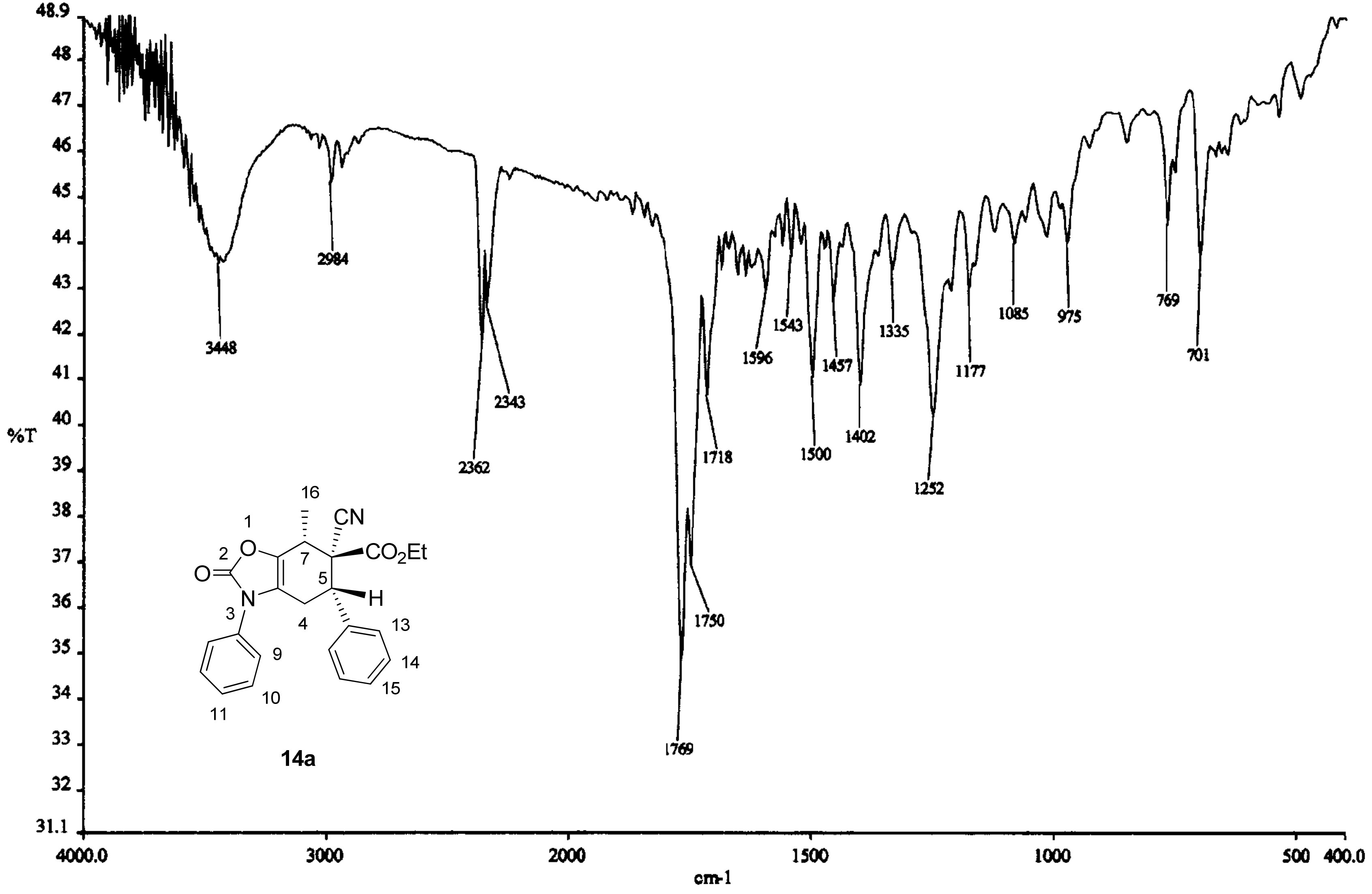
48.9
48
47
46
45
44
43
42
41
40
39
38
37
36
35
34
33
32
31.1
%T
4000.0
3000
2000
1500
1000
500
400.0
cm-1
3448
2984
2362
2343
1769
1750
1718
1596
1543
1500
1457
1402
1335
1252
1177
1085
975
769
701
CN
CO_2Et
H
O
N
14a

FT-IR (KBr) **14a**

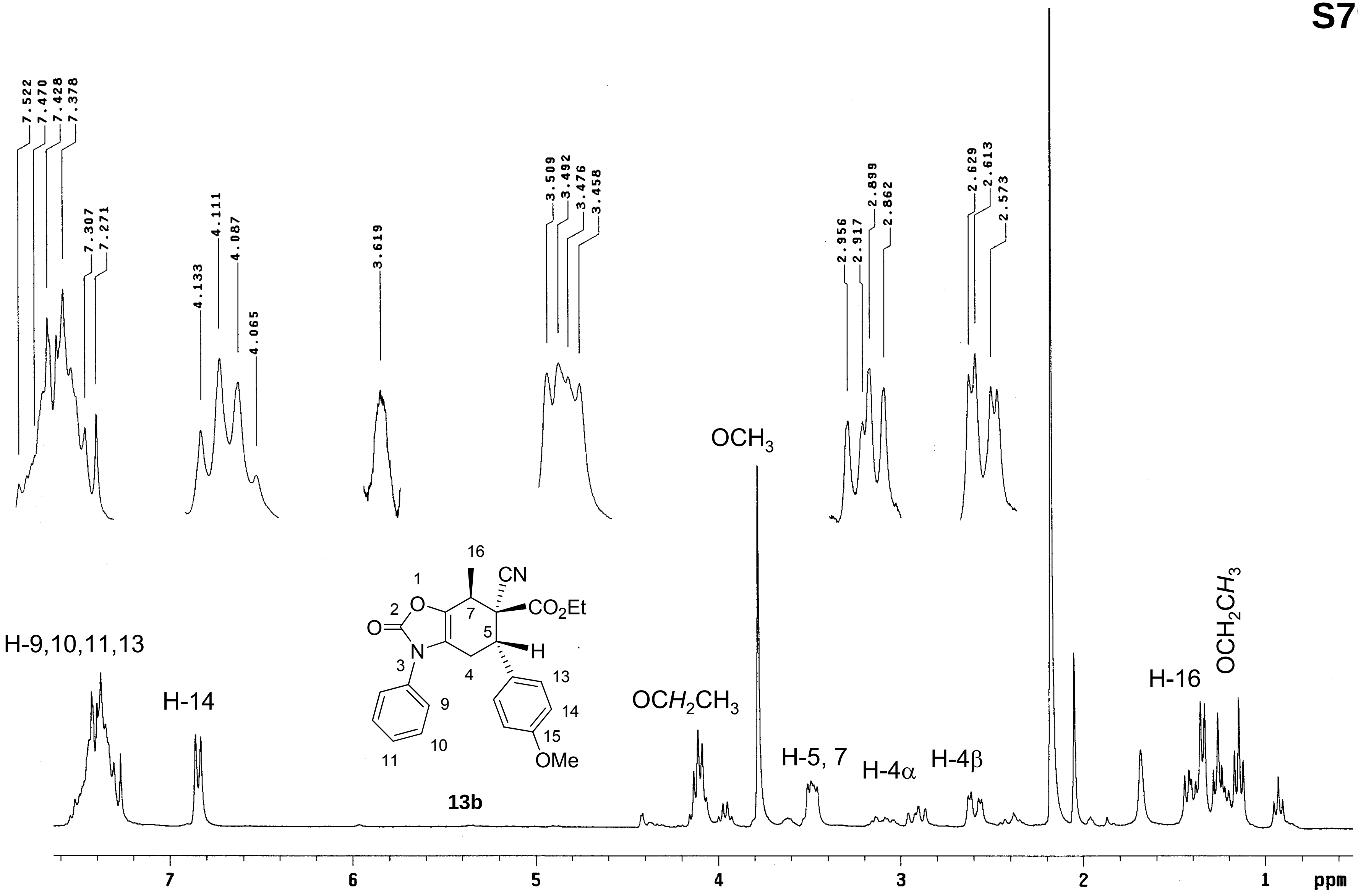

^{1}H NMR ($CDCl_3$) **13b**

S80

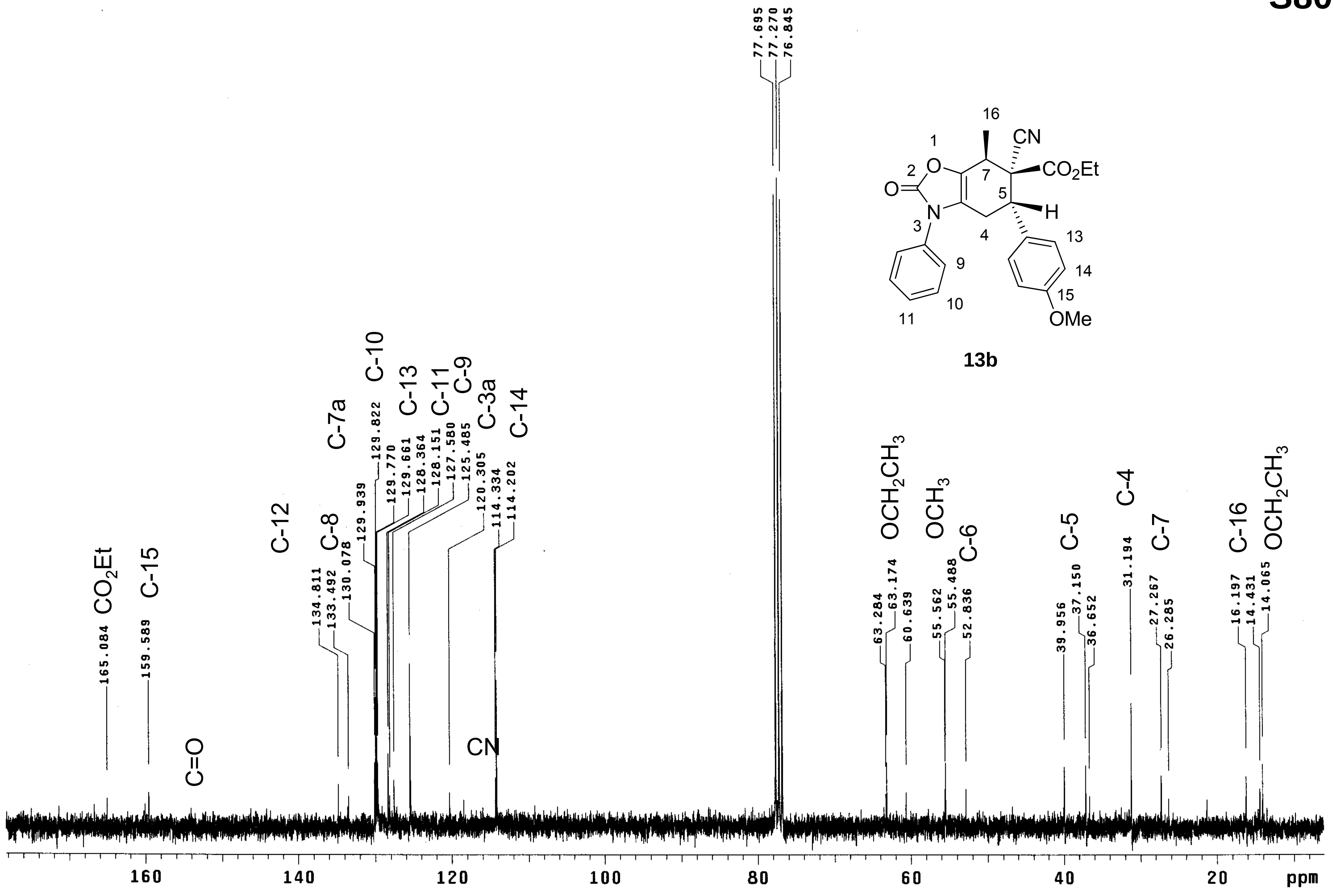

^{13}C NMR ($CDCl_3$) **13b**

File: Ines MI55 IDIE 432Date Run: 05-29-2008 (Time Run: 23:34:28)
Sample: Ines MI55 IDIE 432
Instrument: JEOL GCmate
Inlet: Direct Probe **Ionization mode: EI+**

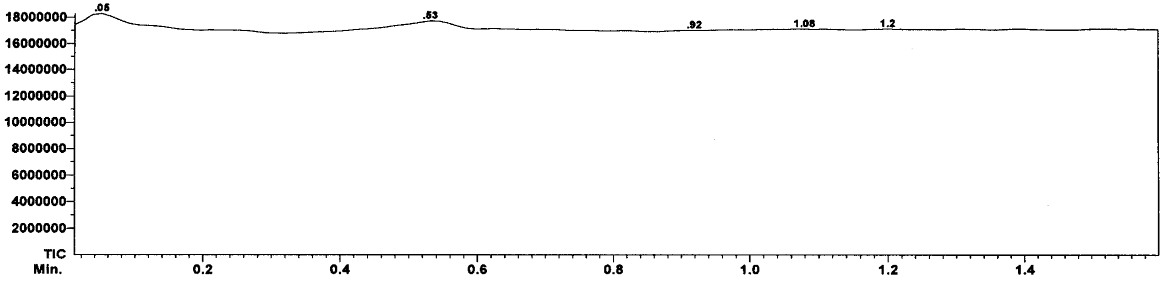

Scan: 33-43 **R.T.: .51**
Base: m/z 281; 1.5%FS TIC: 862901 **#Ions: 1760**

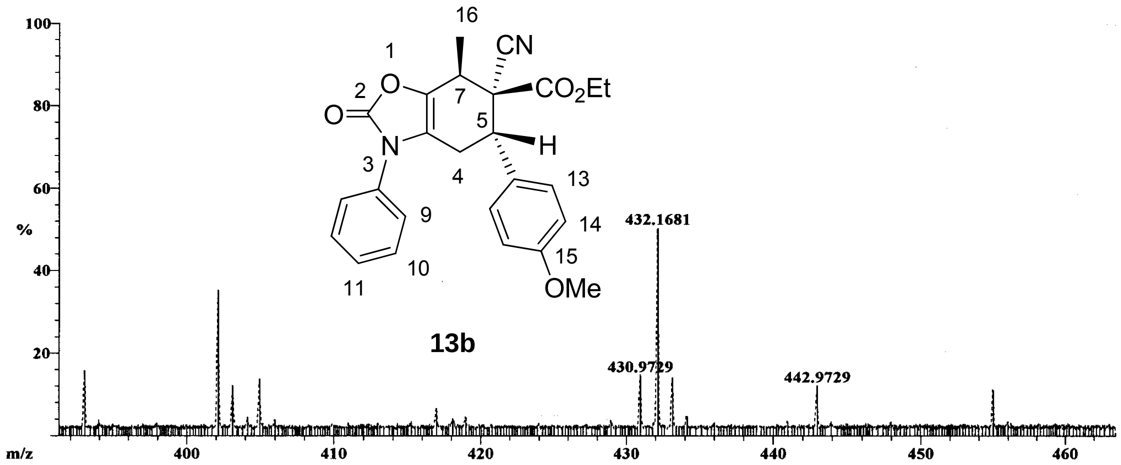

13b

Selected Isotopes : $H_{0-24}C_{0-25}N_{0-2}O_{0-5}$ **Error Limit : 20 mmu**

Measured Mass	*% Base*	*Formula*	*Calculated Mass*	*Error*
432.1681	50.4%	$C_{25}H_{24}N_2O_5$	432.1685	-0.4

HRMS **13b**

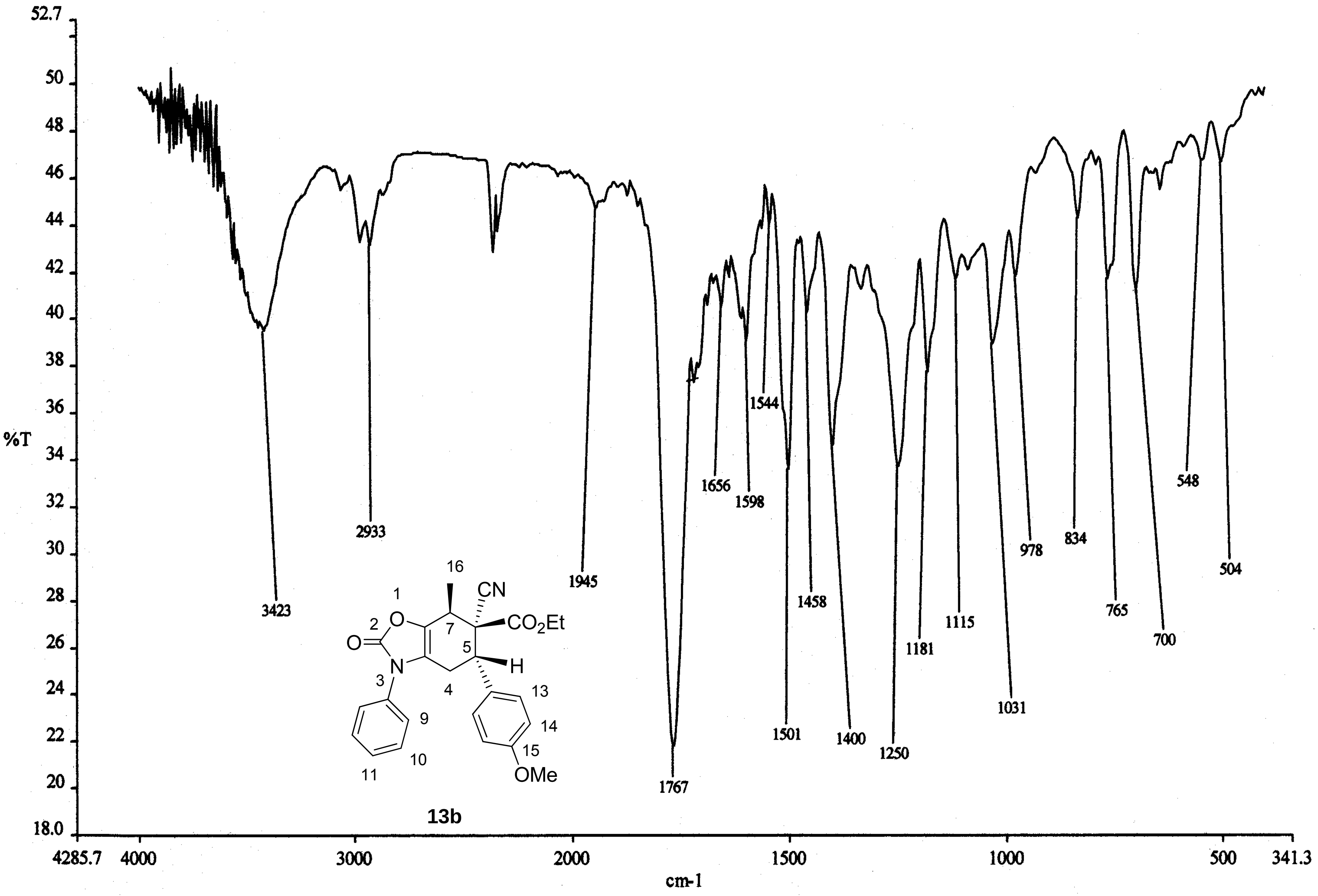
52.7
50
48
46
44
42
40
38
36
34
32
30
28
26
24
22
20
18.0
%T
4285.7
4000
3000
2000
1500
1000
500
341.3
cm-1
3423
2933
1945
1767
1656
1598
1544
1501
1458
1400
1250
1181
1115
1031
978
834
765
700
548
504
13b

FT-IR (KBr) **13b**

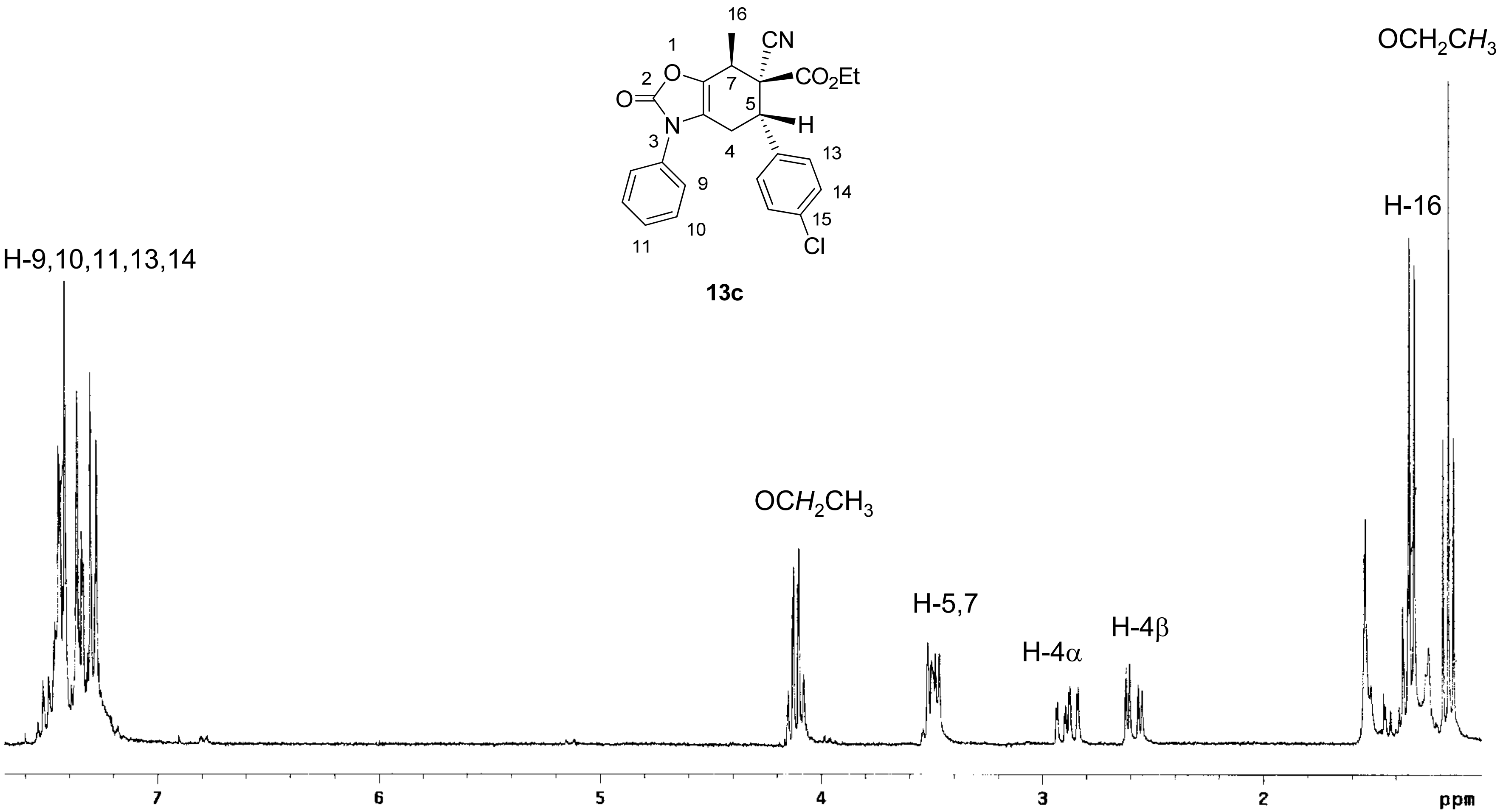

^{1}H NMR ($CDCl_3$) **13d**

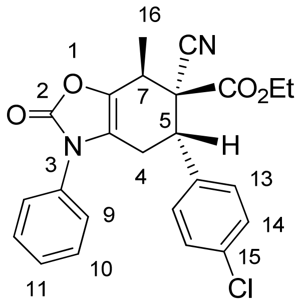

13c

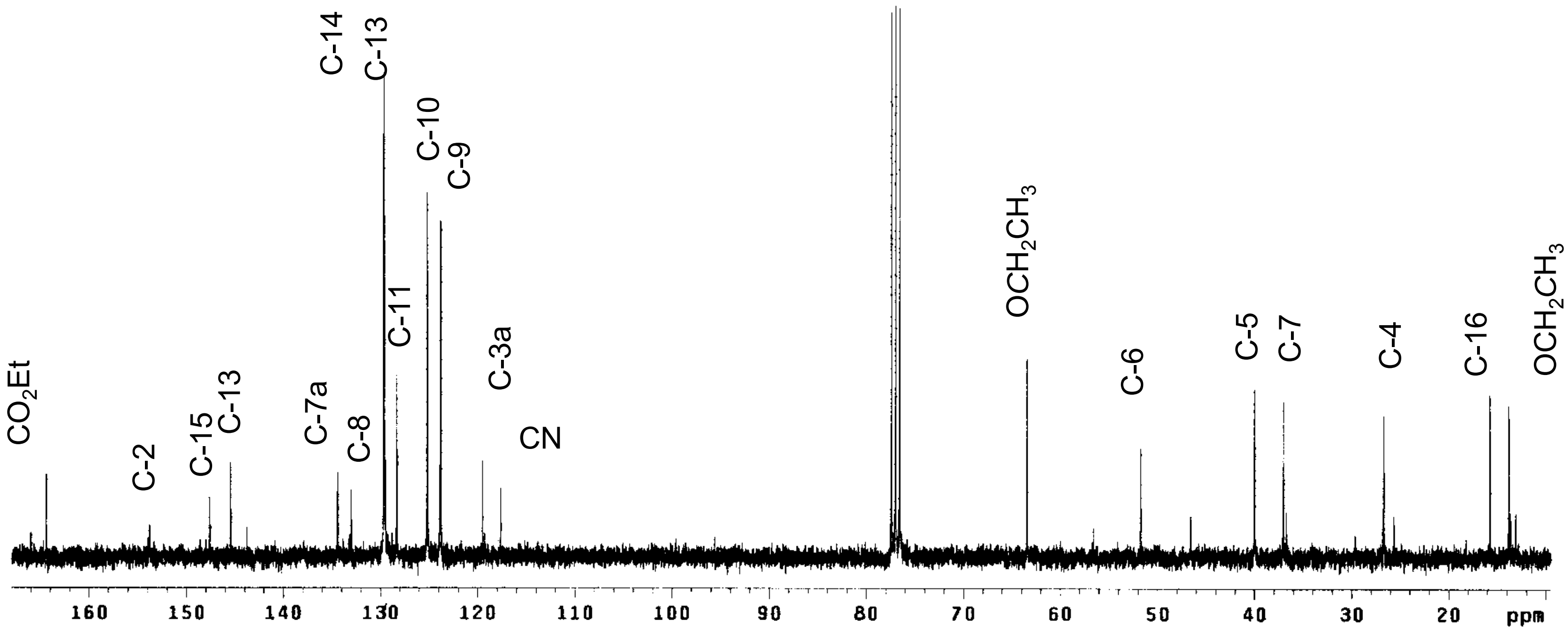

^{13}C NMR (CDCl$_3$) **13c**

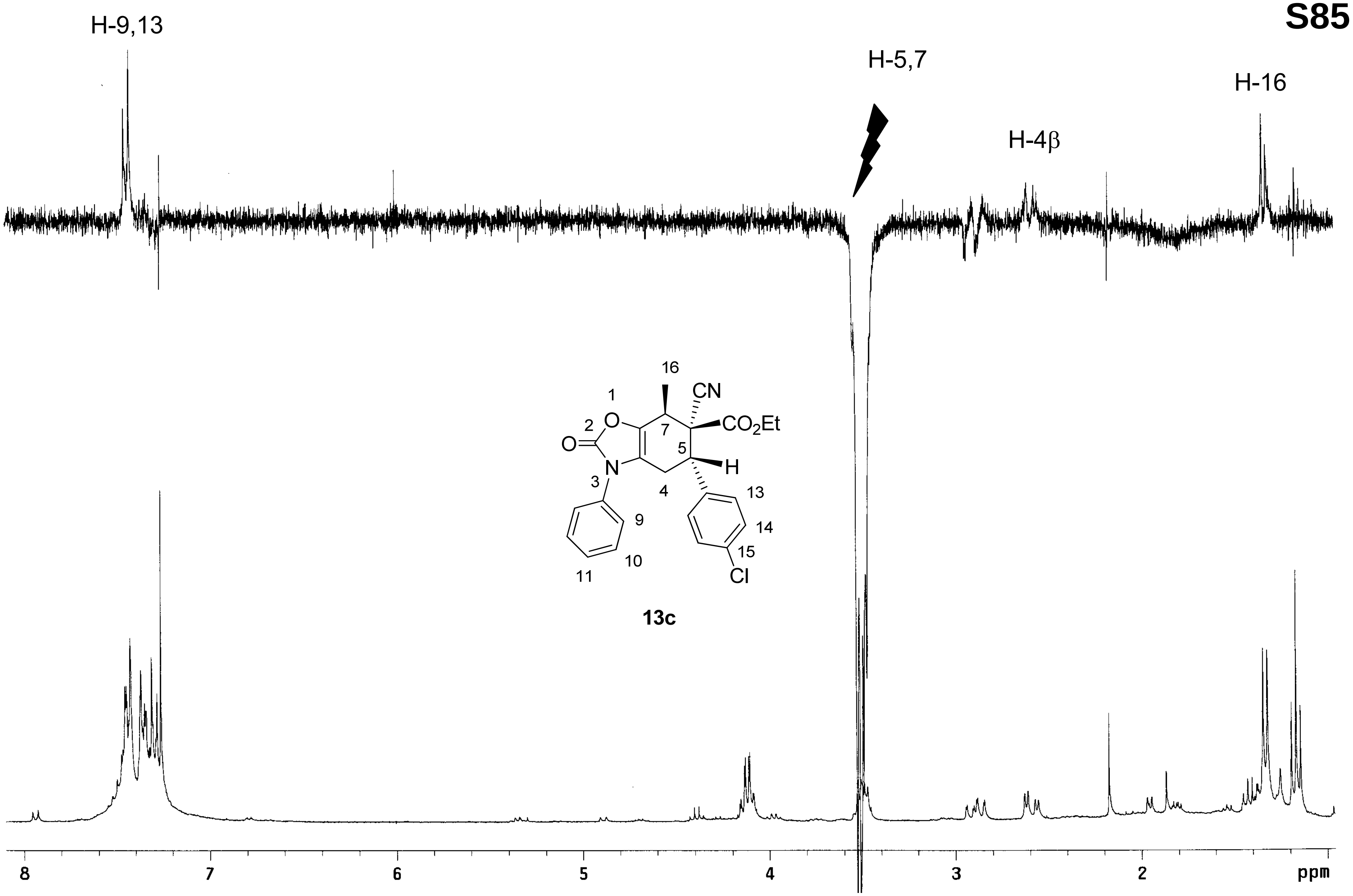

NOE experiment ($CDCl_3$) **13c**

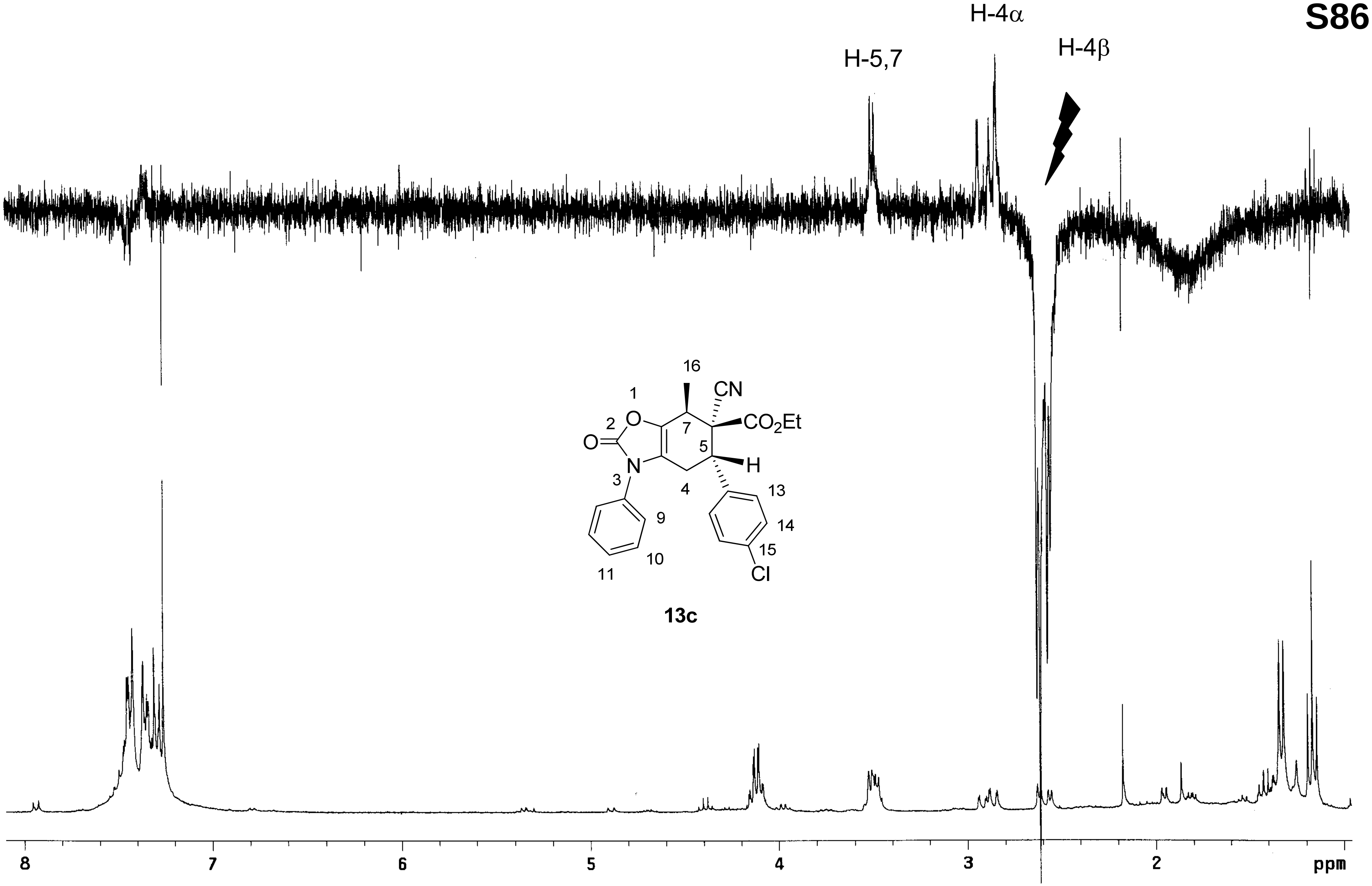

NOE experiment ($CDCl_3$) **13c**

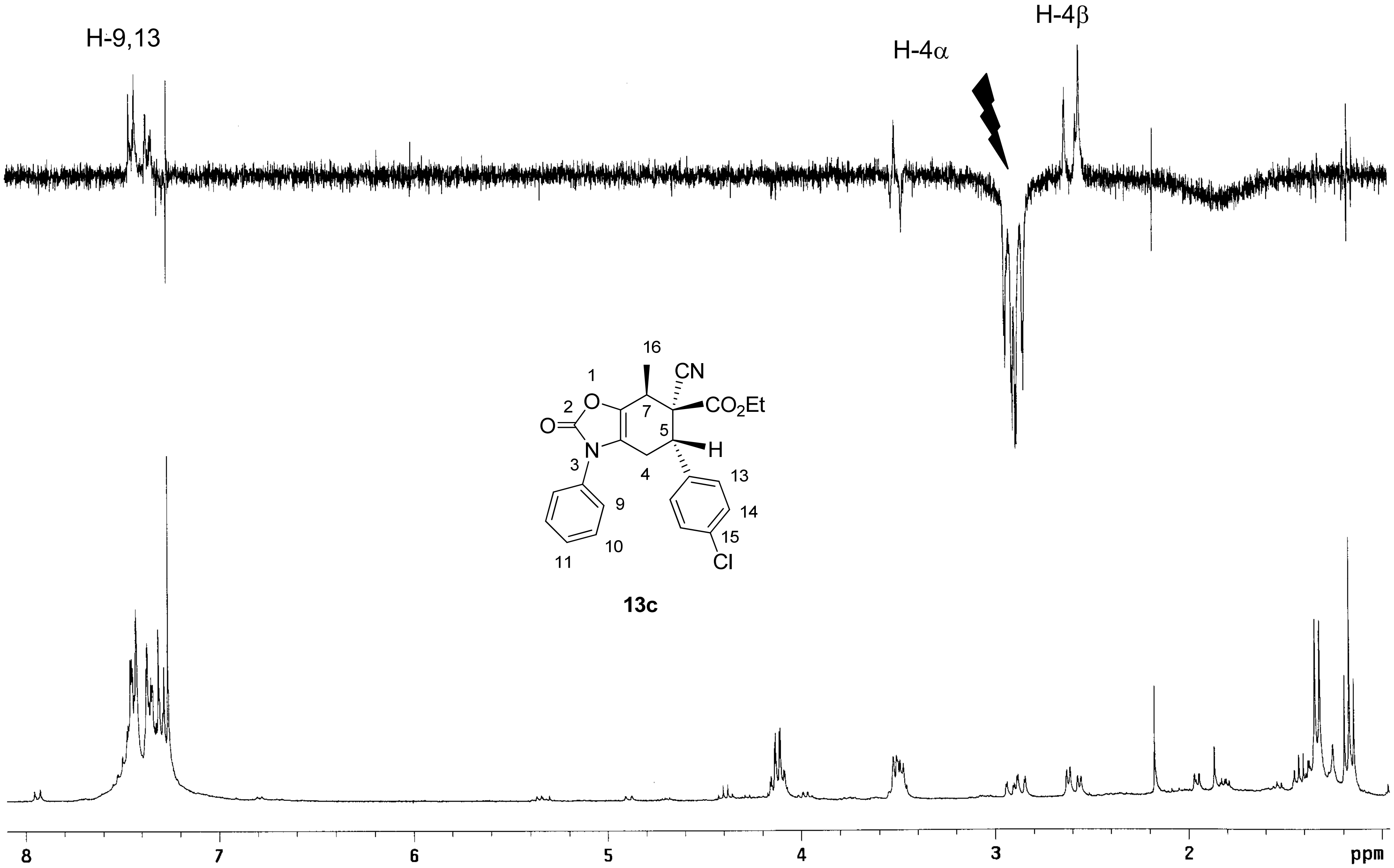

NOE experiment ($CDCl_3$) **13c**

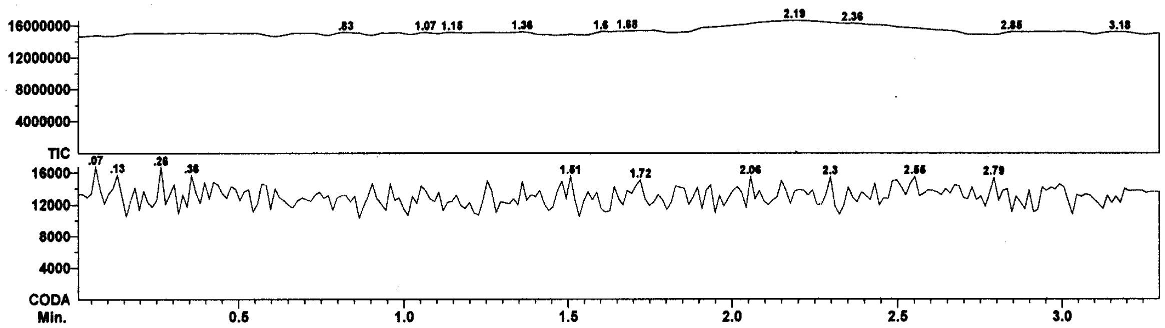

Scan: 145-172 **R.T.: 2.12**

Base: m/z 436; 1.4%FS TIC: 764291 **#Ions: 1755**

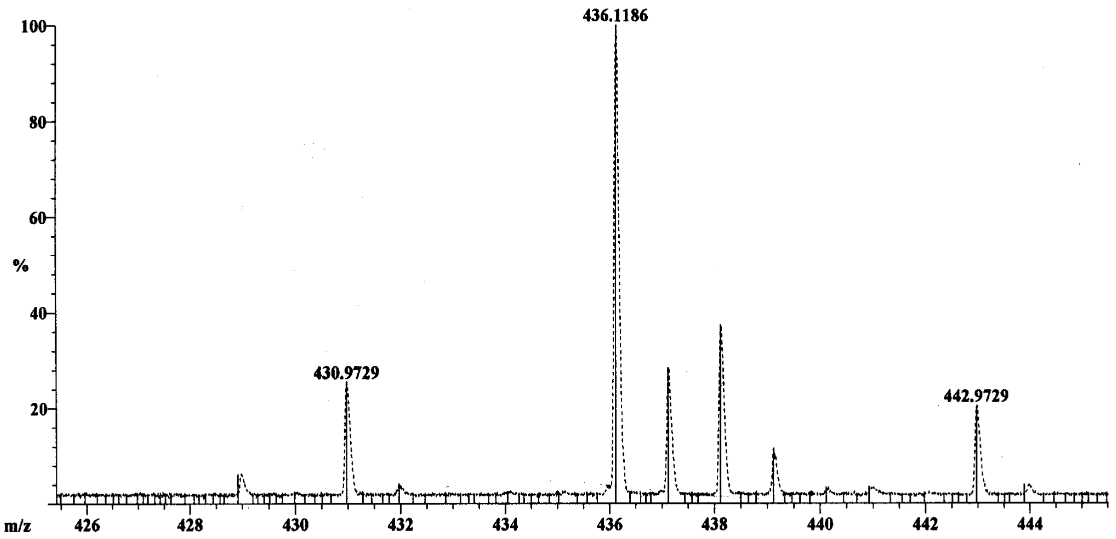

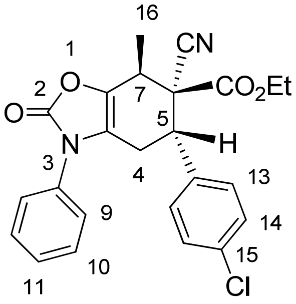

13c

Selected Isotopes : $H_{0-21}C_{0-24}N_{0-2}O_{0-4}Cl_{0-1}$ **Error Limit : 100 mmu**

Measured Mass	***% Base***	***Formula***	***Calculated Mass***	***Error***
436.1186	100.0%	$C_{24}H_{21}N_2O_4Cl$	436.1190	-0.4

HRMS **13c**

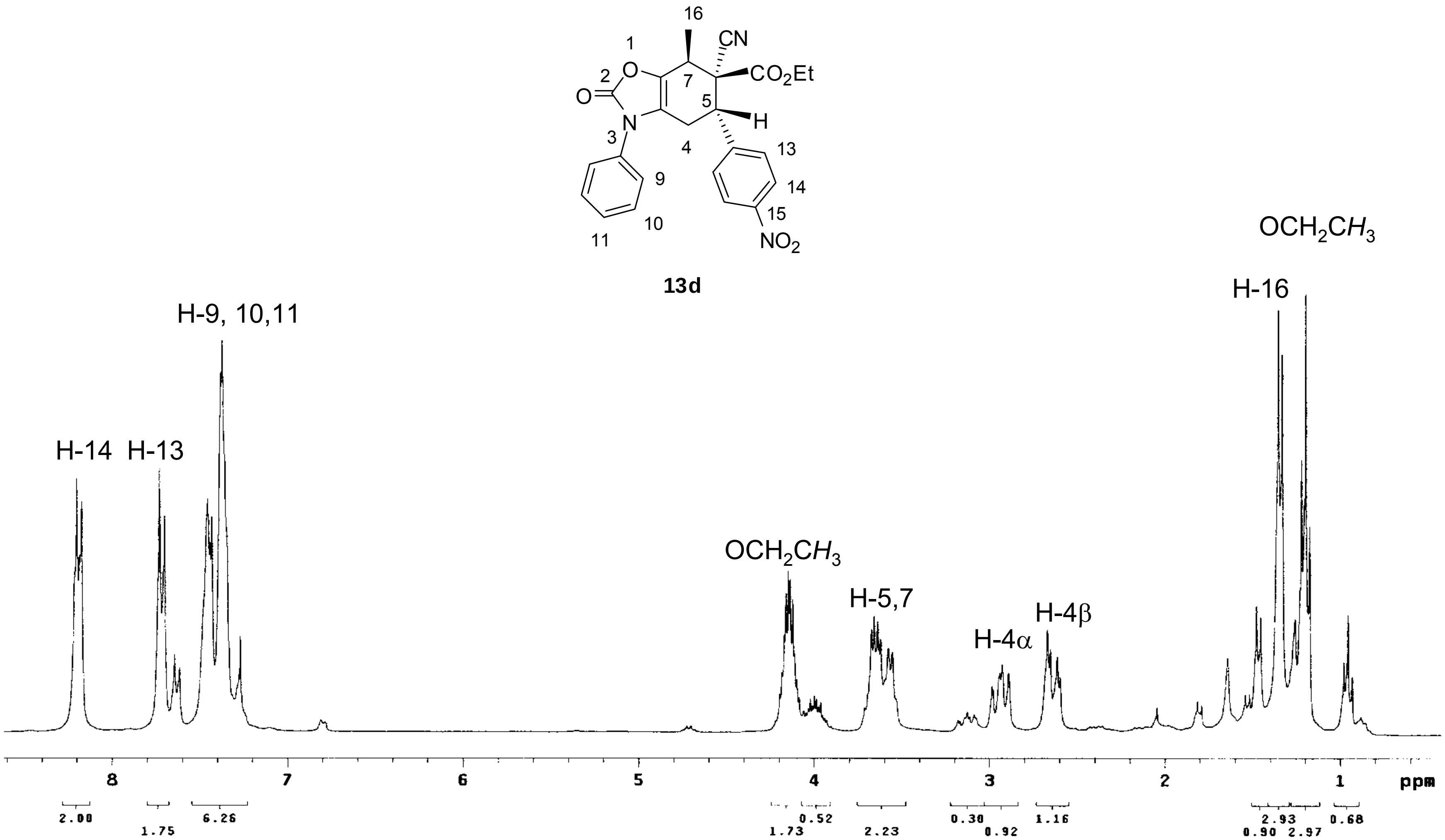

^{1}H NMR ($CDCl_3$) **13d**

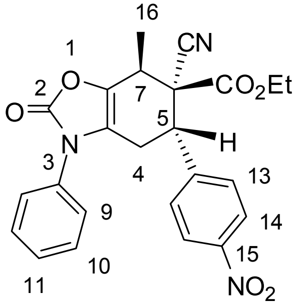

13d

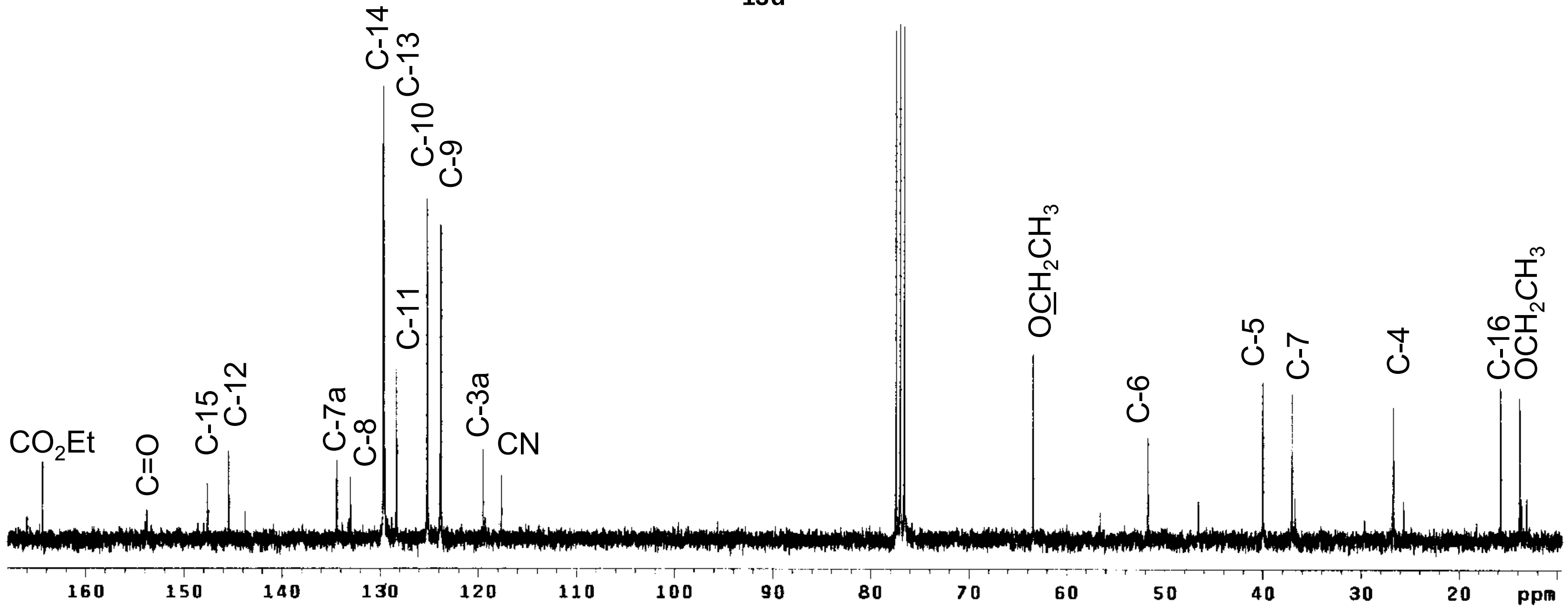

^{13}C NMR ($CDCl_3$) **13d**

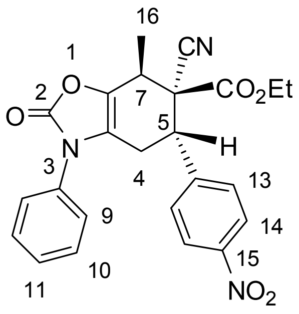

13d

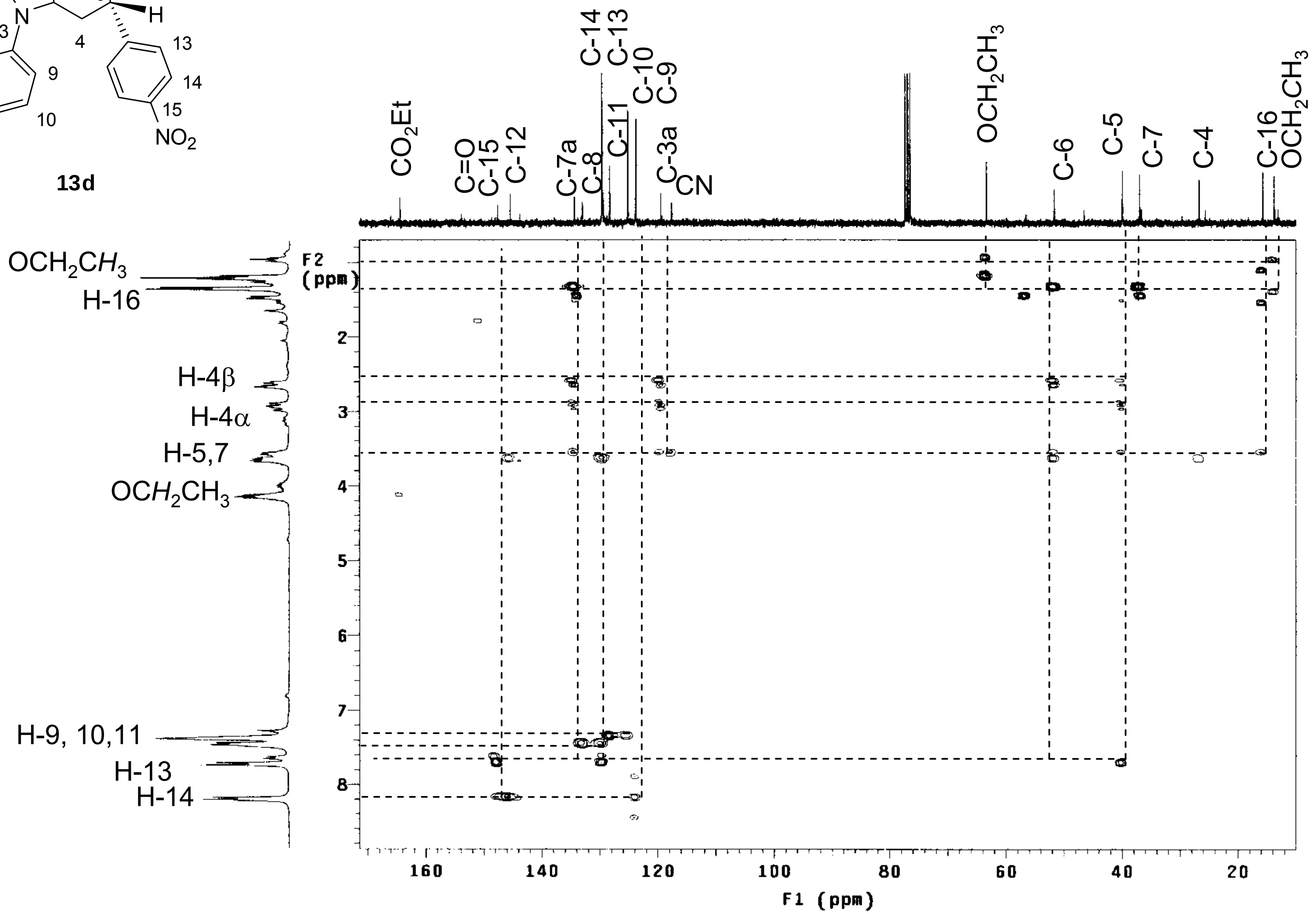

HMBC experiment ($CDCl_3$) **13d**

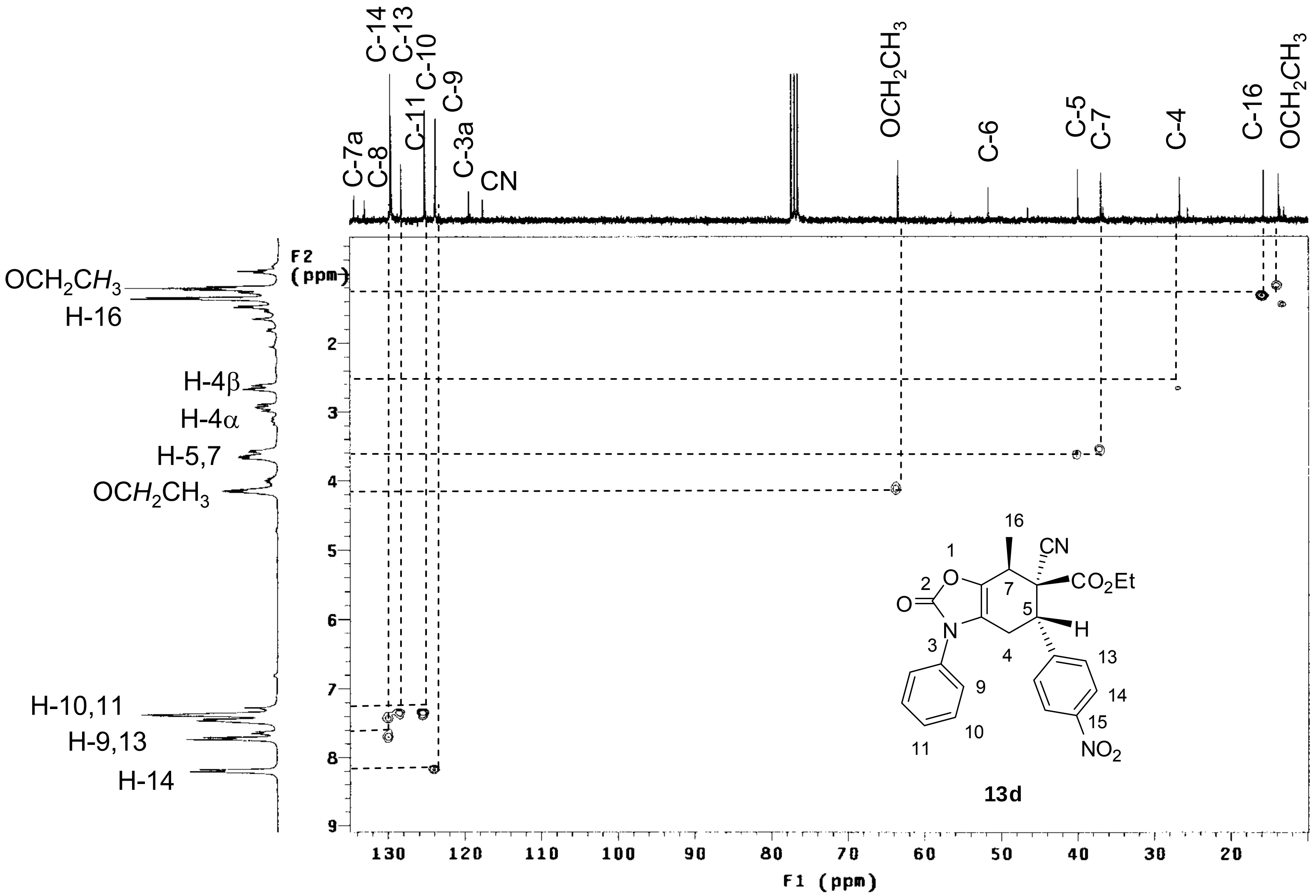

HSQC experiment ($CDCl_3$) **13d**

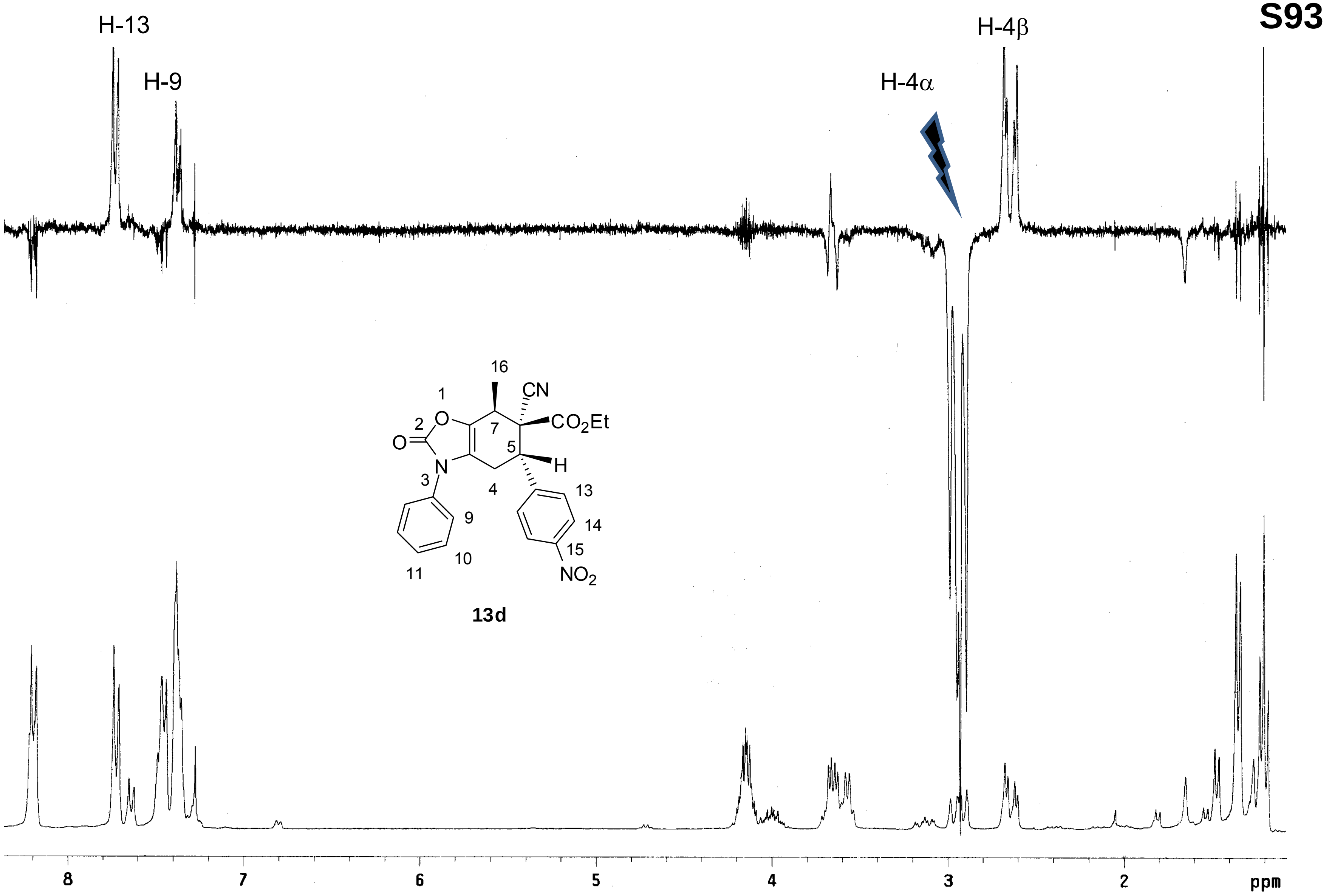

NOE experiment ($CDCl_3$) **13d**

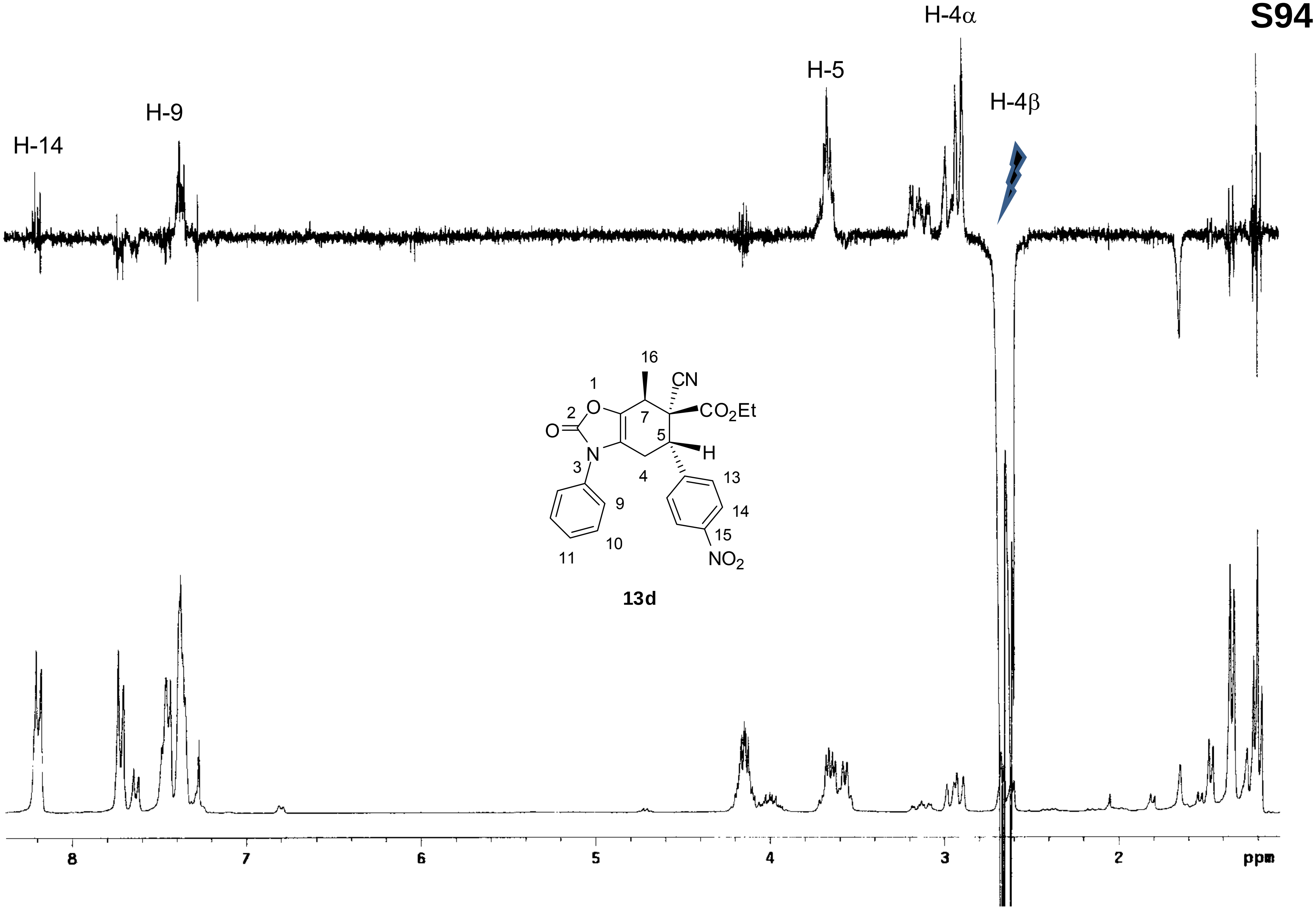

NOE experiment ($CDCl_3$) **13d**

File: FDR 447 IEID sdaDate Run: 06-05-2008 (Time Run: 04:56:27)
Sample: MI 6
Instrument: JEOL GCmate
Inlet: Direct Probe **Ionization mode: EI+**

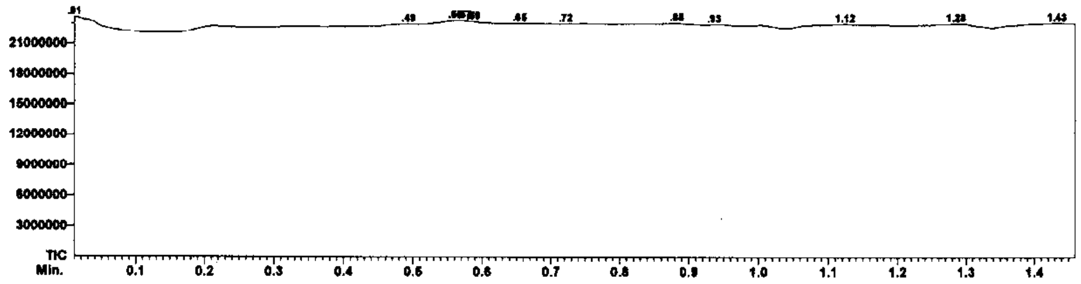

Scan: 36-39 **R.T.: .5**
Base: m/z 281; 14.5%FS TIC: 1569580 **#Ions: 1805**

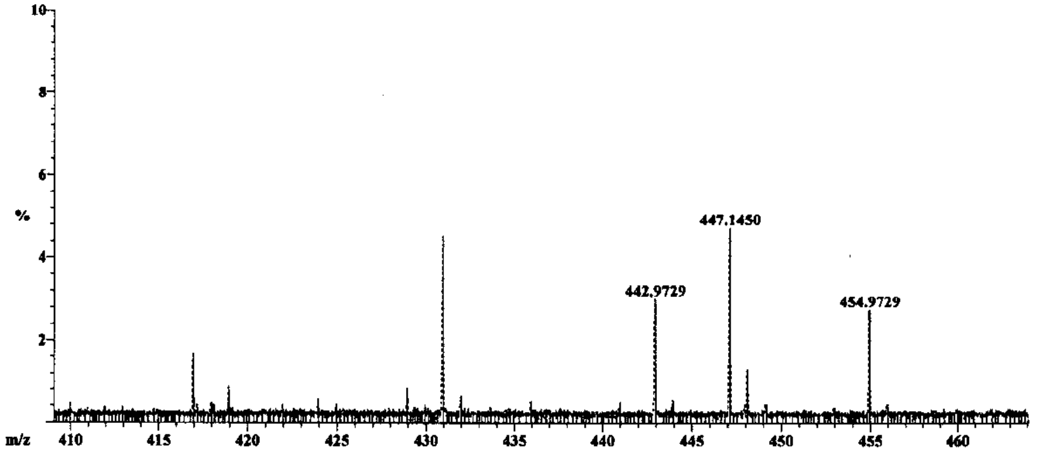

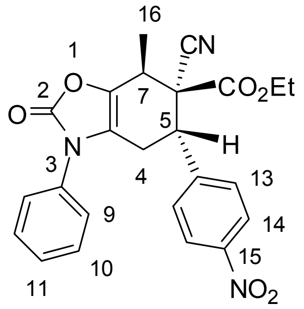

13d

Selected Isotopes : $H_{0\text{-}21}C_{0\text{-}24}N_{0\text{-}3}O_{0\text{-}6}$ **Error Limit : 20 mmu**

Measured Mass	*% Base*	*Formula*	*Calculated Mass*	*Error*
447.1450	4.7%	$C_{24}H_{21}N_3O_6$	447.1430	2.0

HRMS **13d**

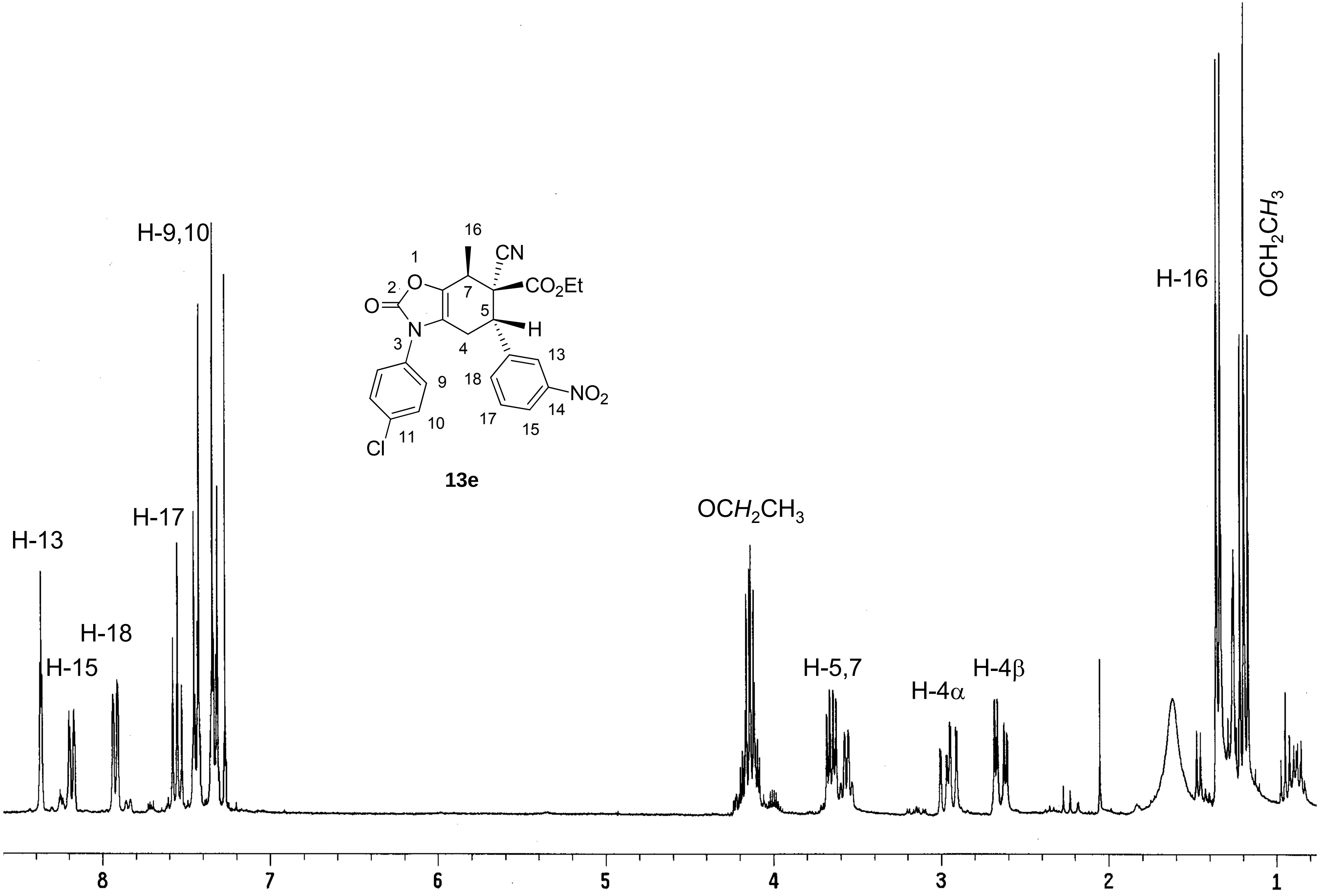

^{1}H NMR ($CDCl_3$) **13e**

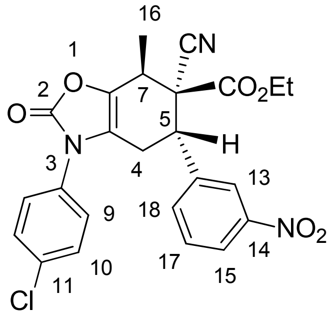

13e

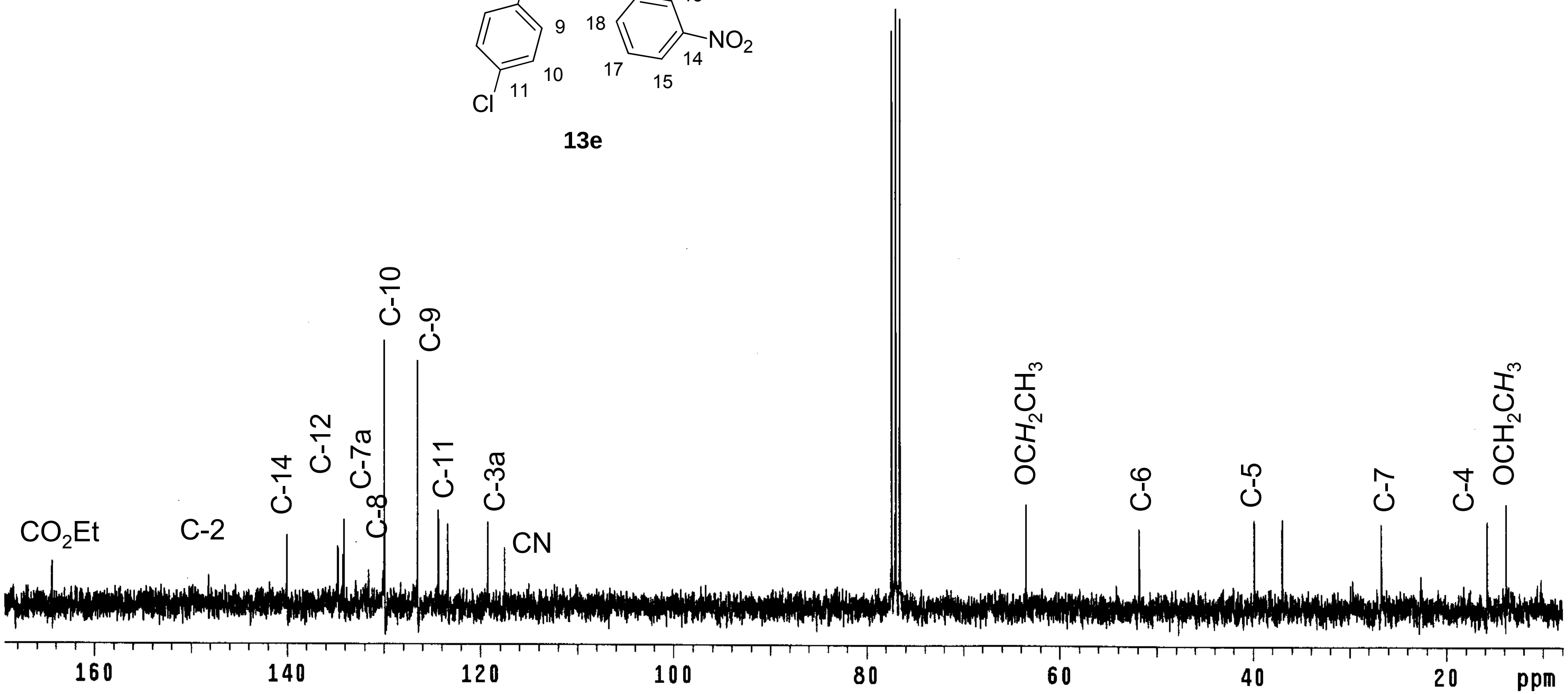

^{13}C NMR ($CDCl_3$) **13e**

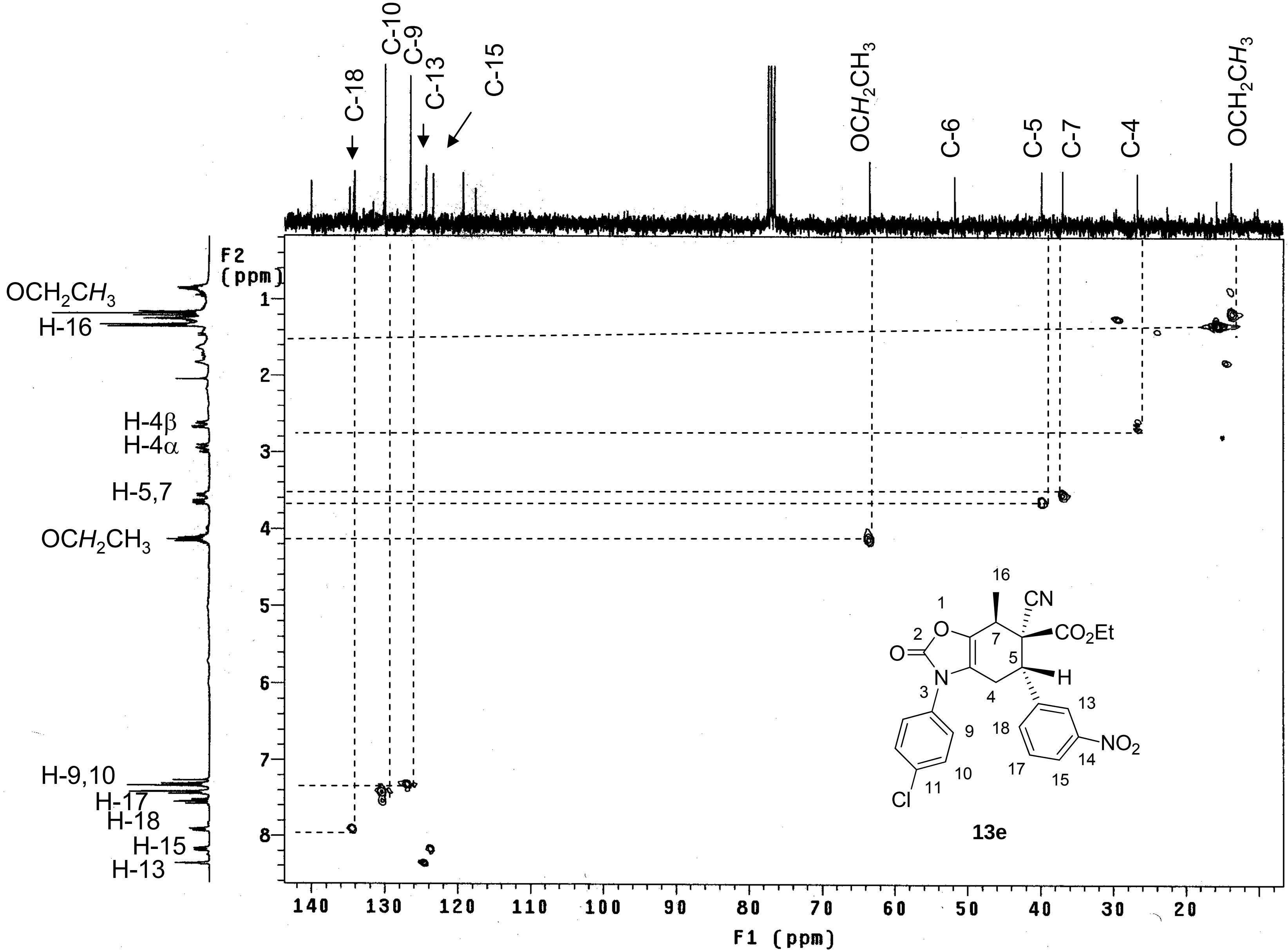

HSQC experiment ($CDCl_3$) **13e**

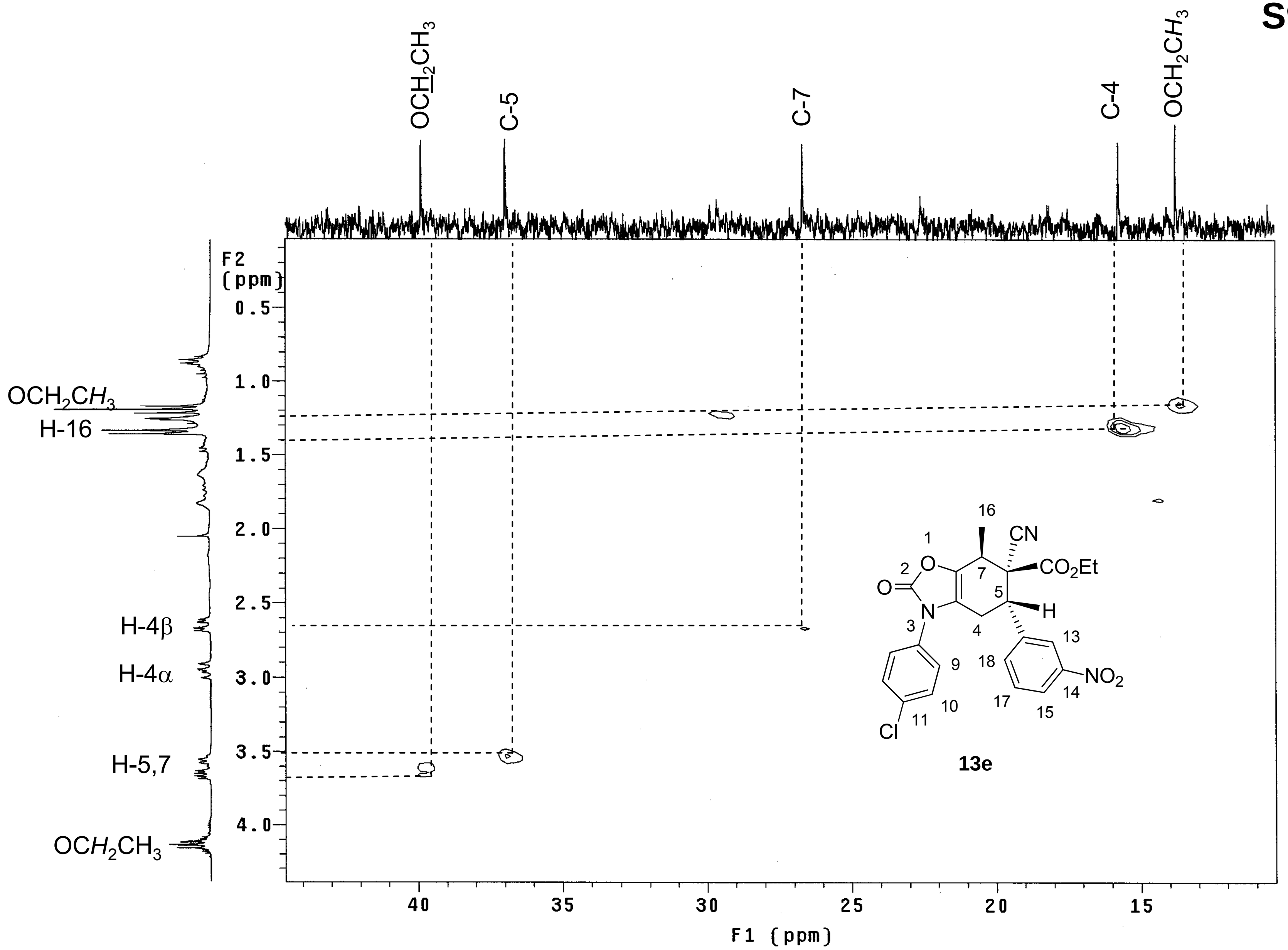

HSQC experiment ($CDCl_3$) **13e**

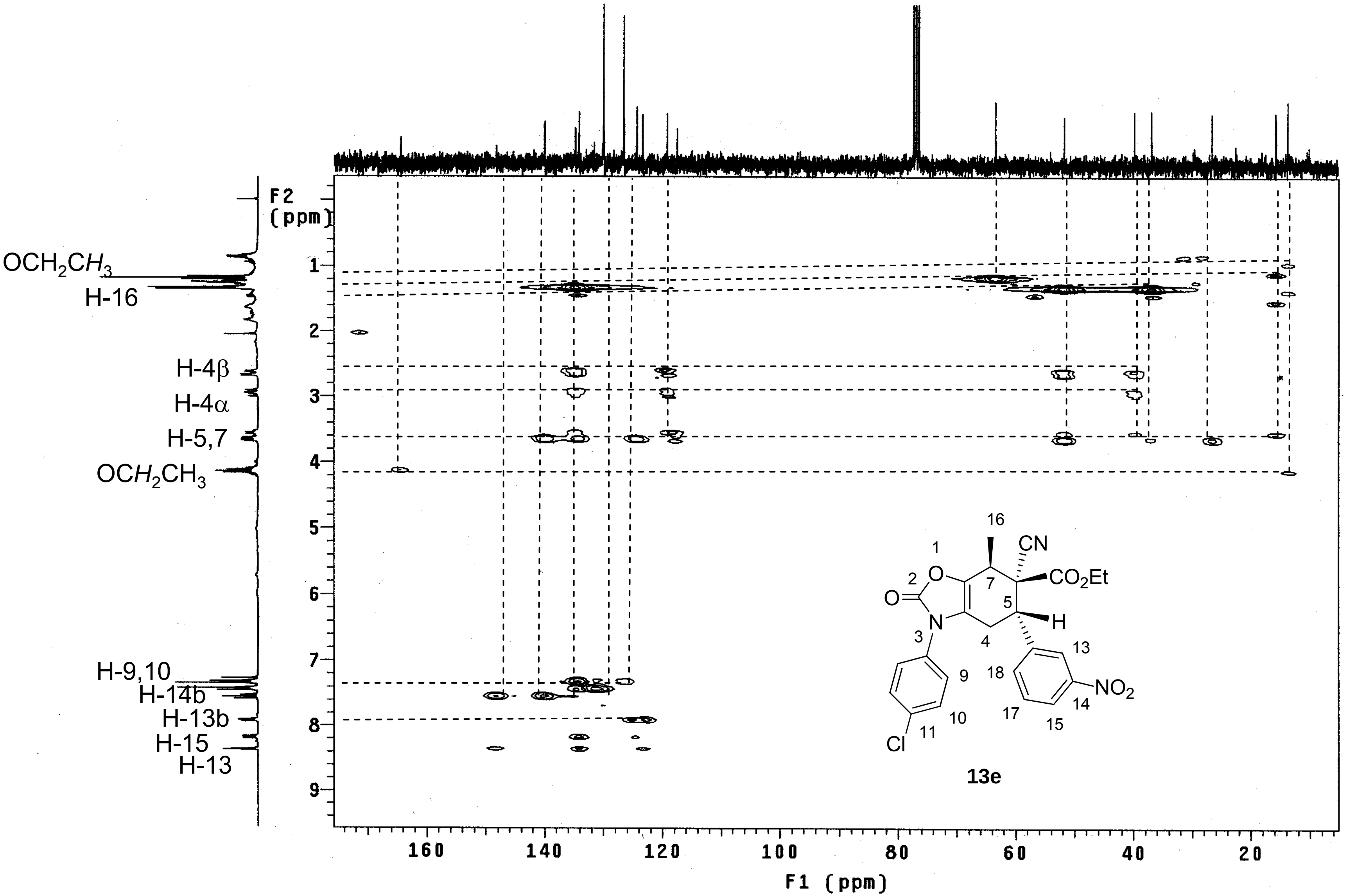

HMBC experiment ($CDCl_3$) **13e**

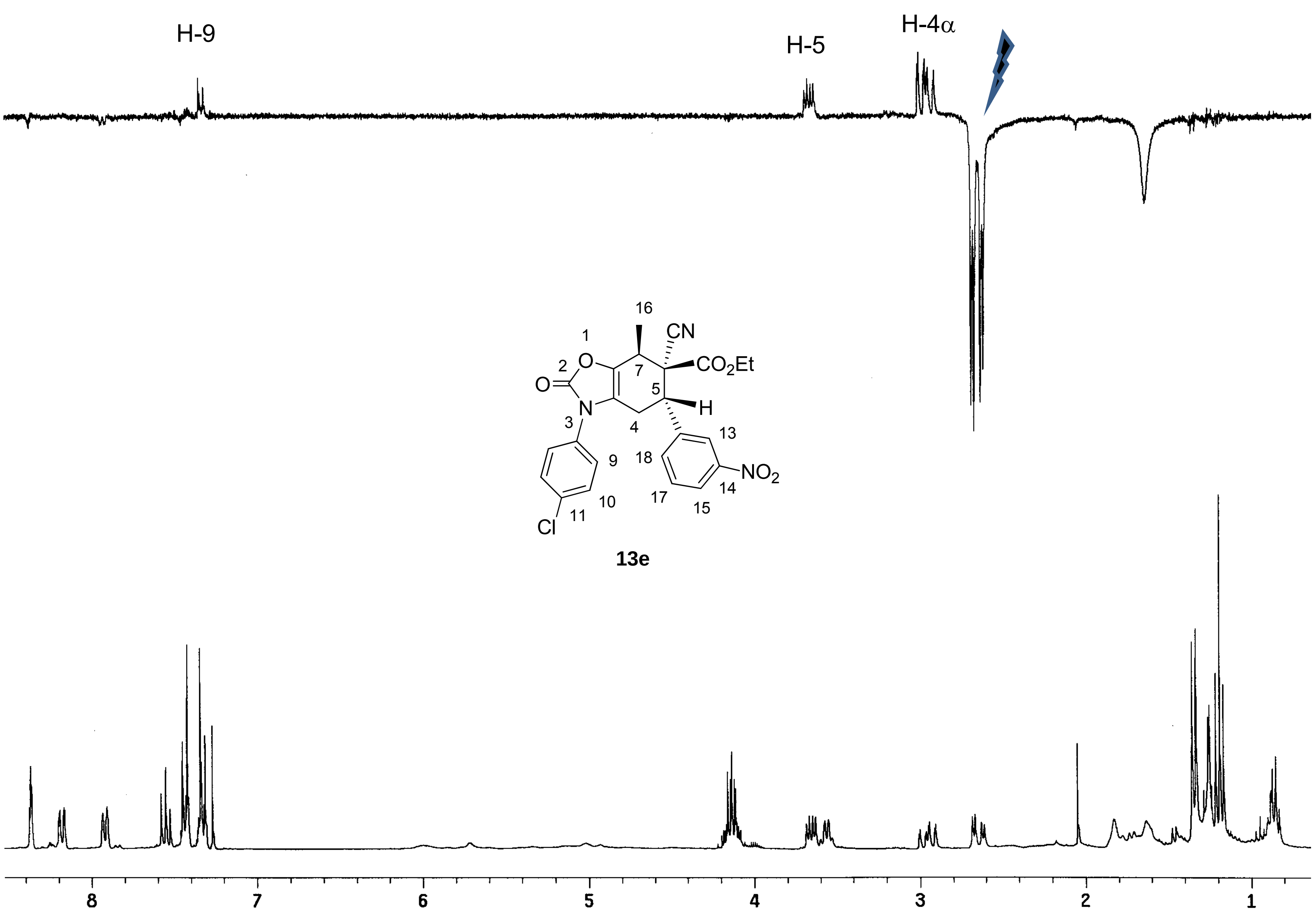

NOE experiment ($CDCl_3$) **13e**

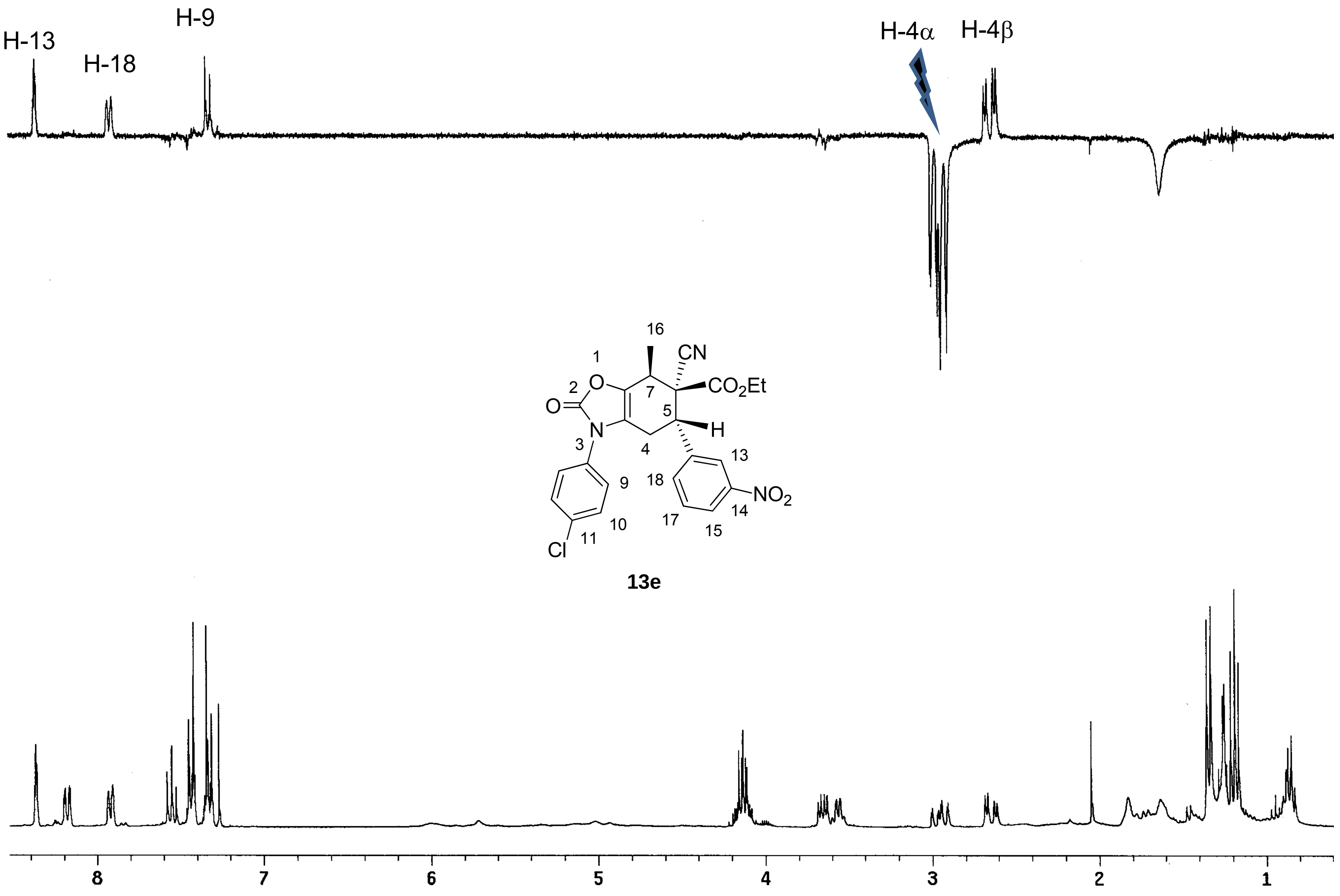

NOE experiment ($CDCl_3$) **13e**

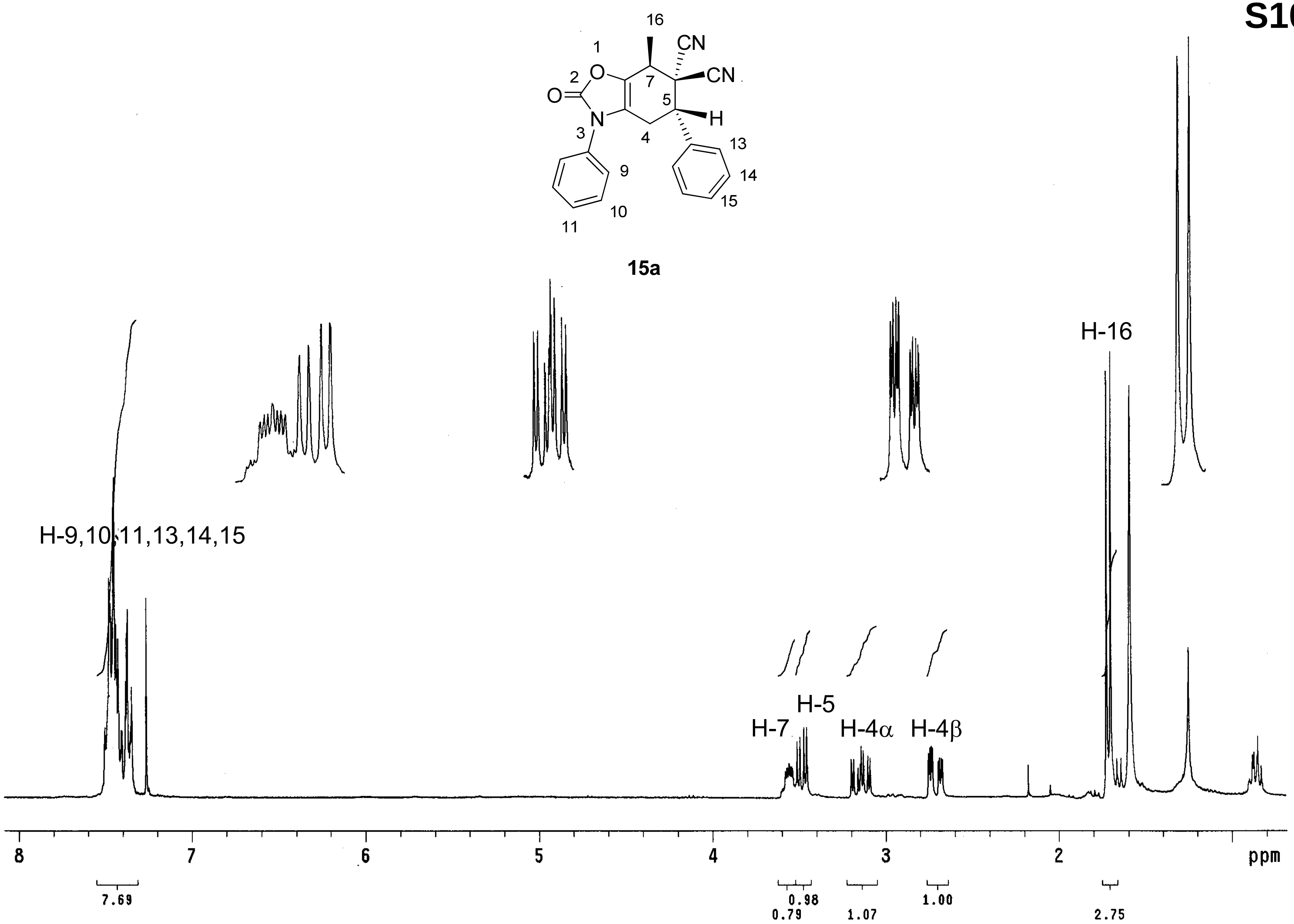

^{1}H NMR ($CDCl_3$) **15a**

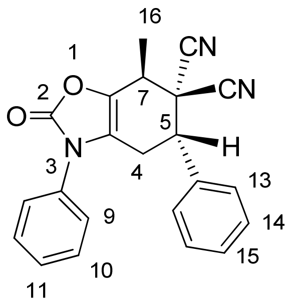

15a

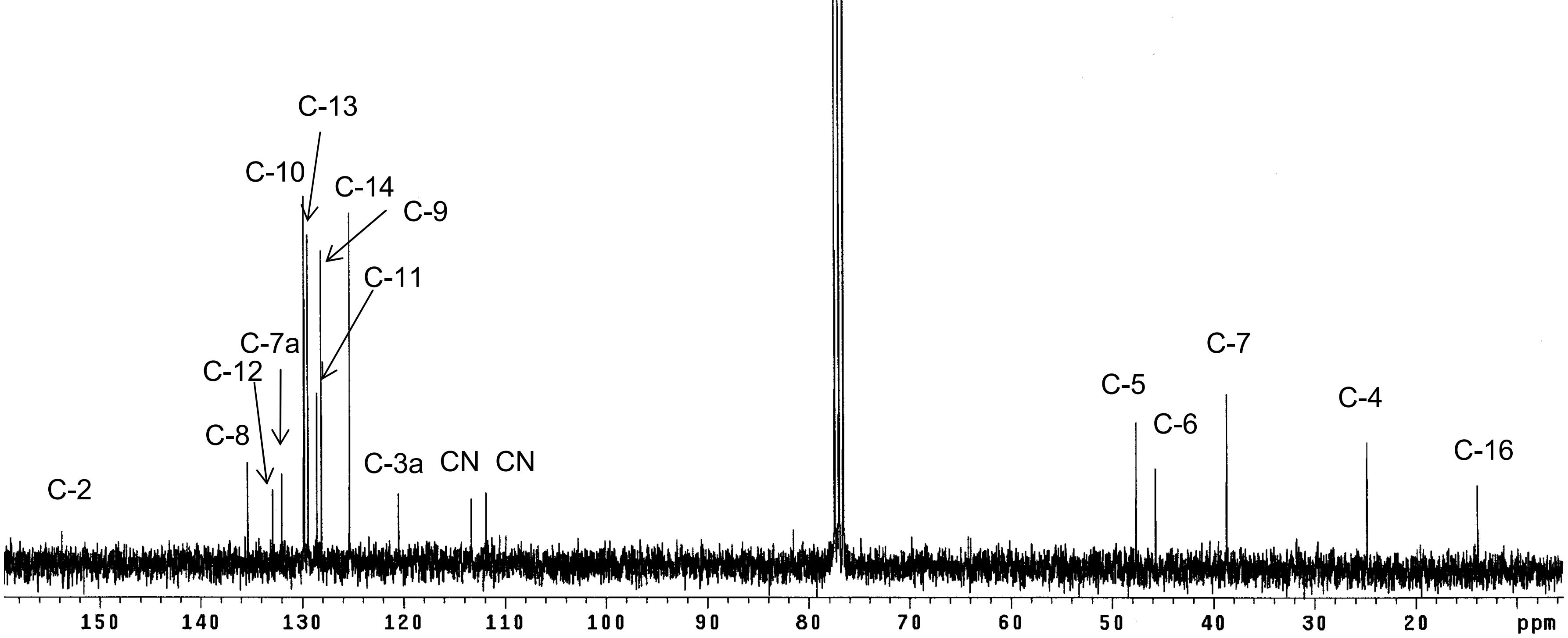

^{13}C NMR ($CDCl_3$) **15a**

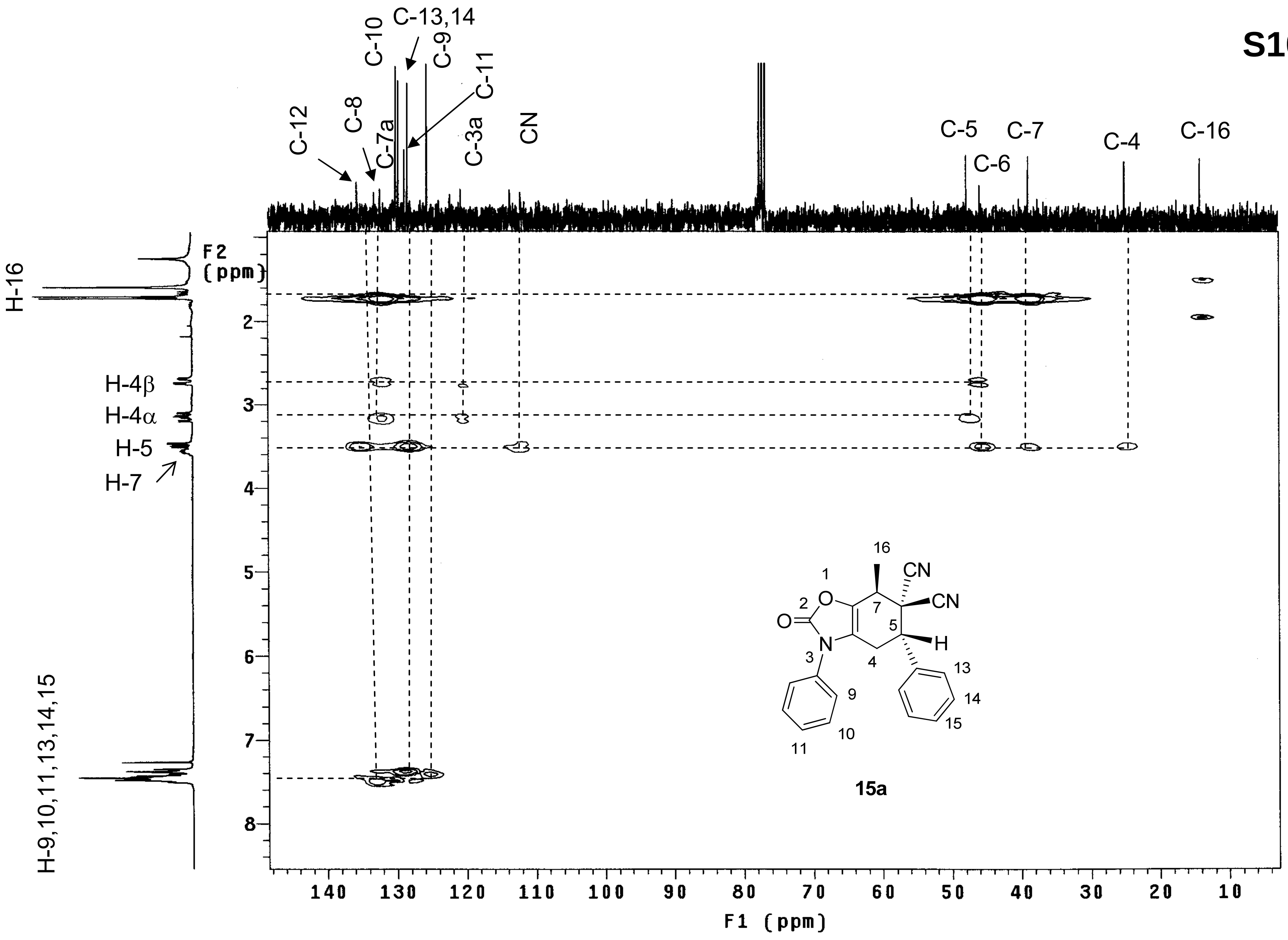

HMBC experiment ($CDCl_3$) **15a**

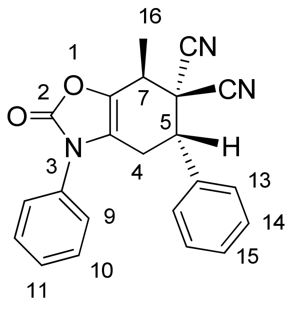

15a

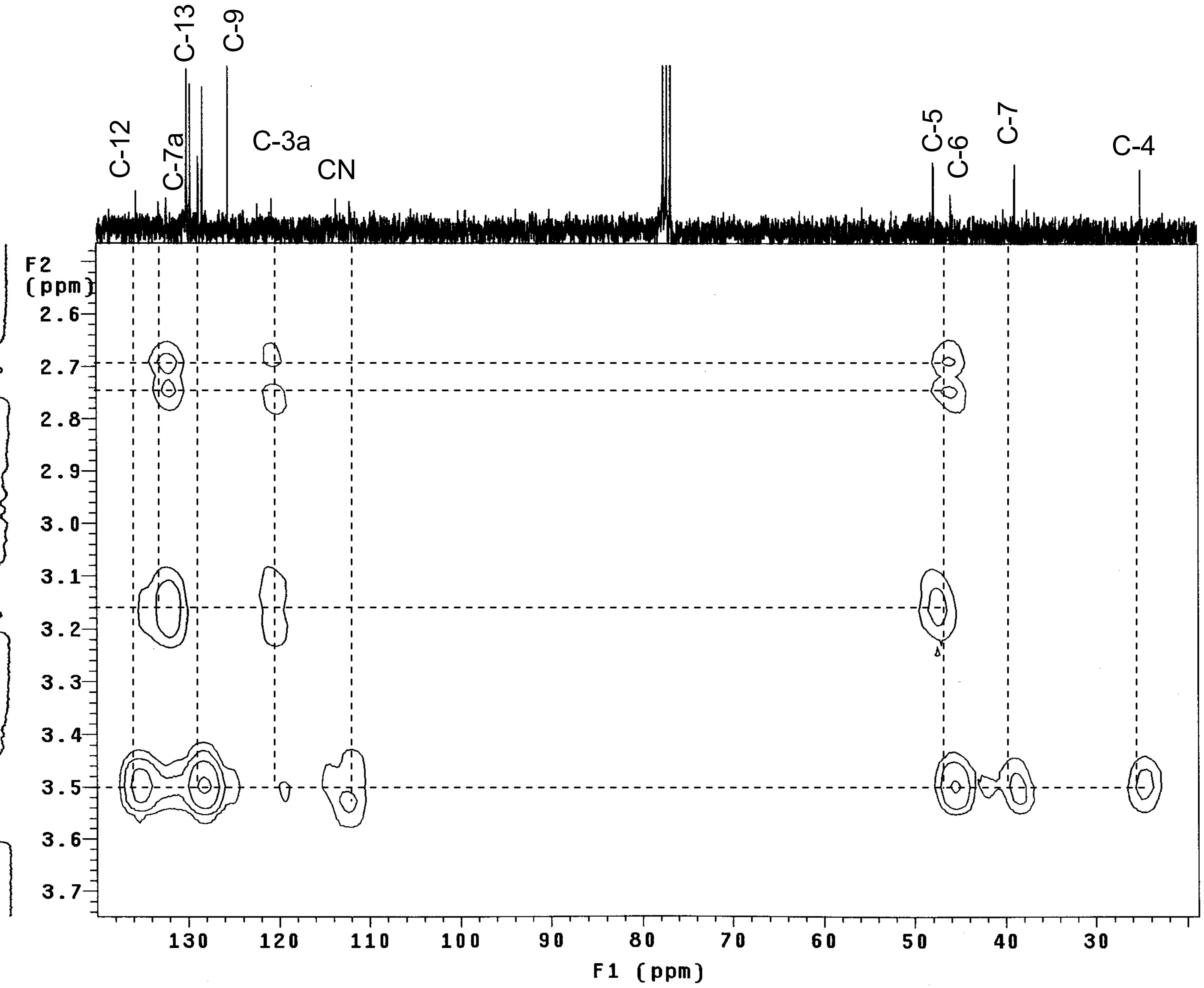

HMBC experiment ($CDCl_3$) **15a**

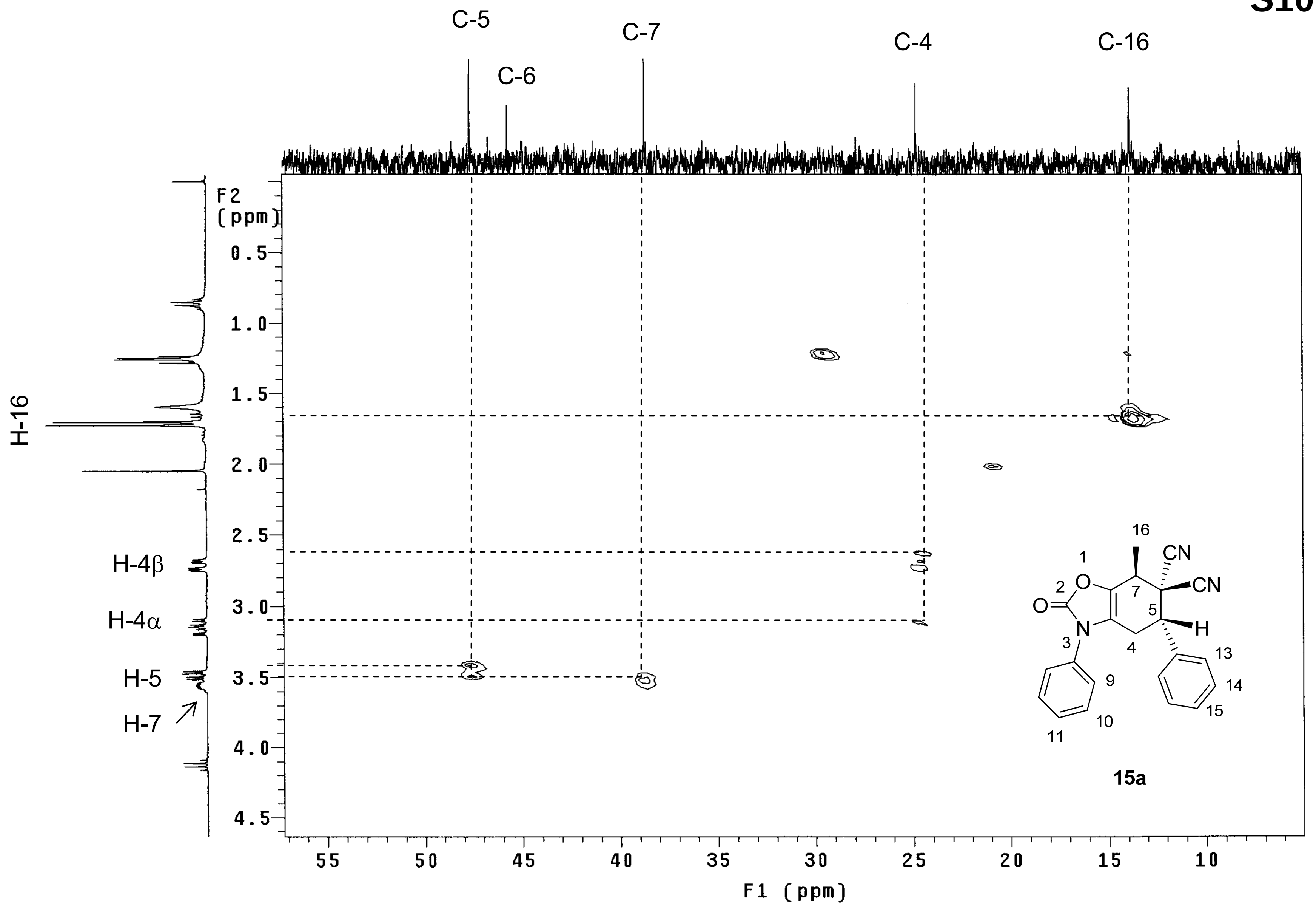

15a

HSQC experiment ($CDCl_3$) **15a**

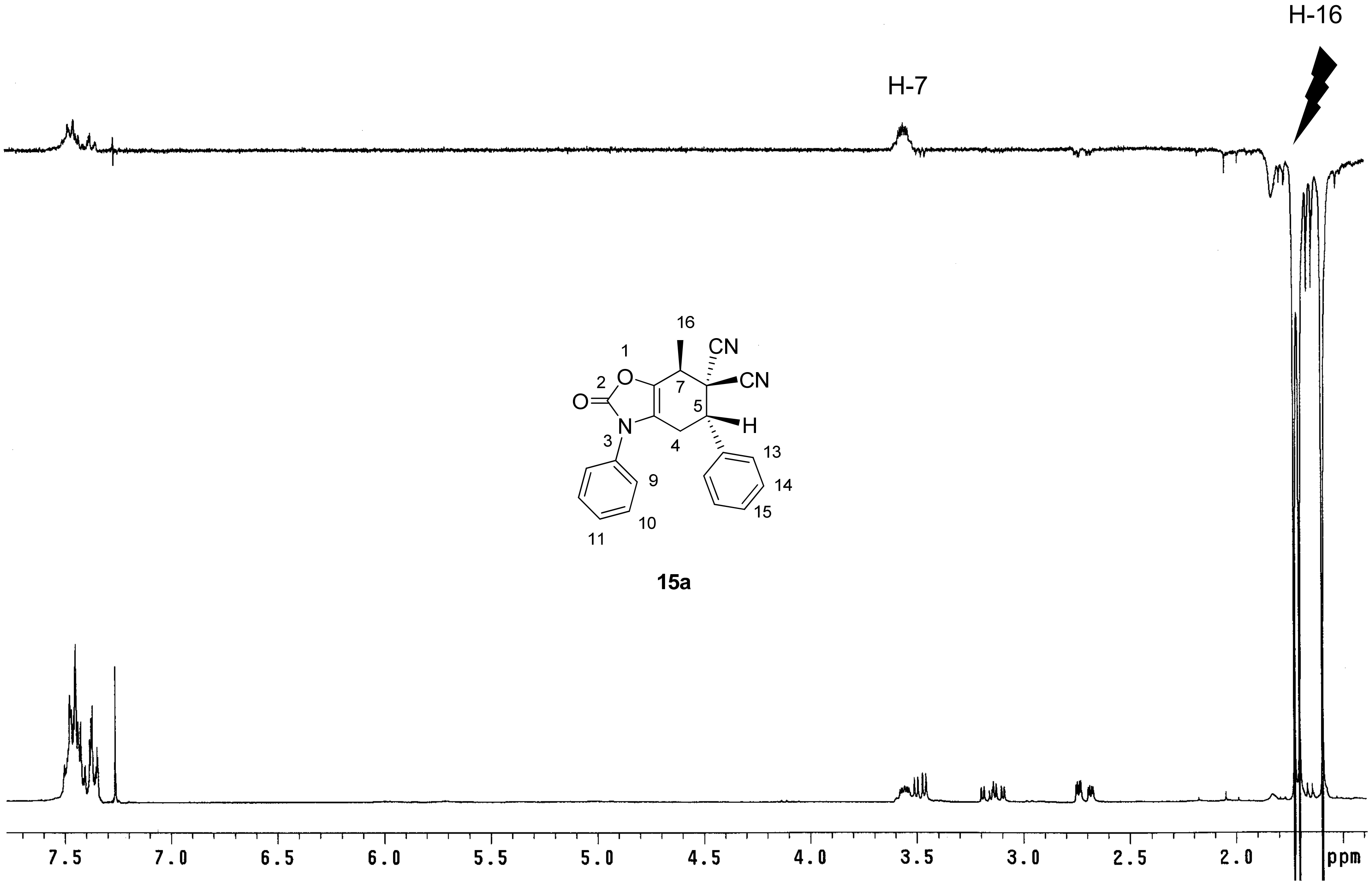

NOE experiment ($CDCl_3$) **15a**

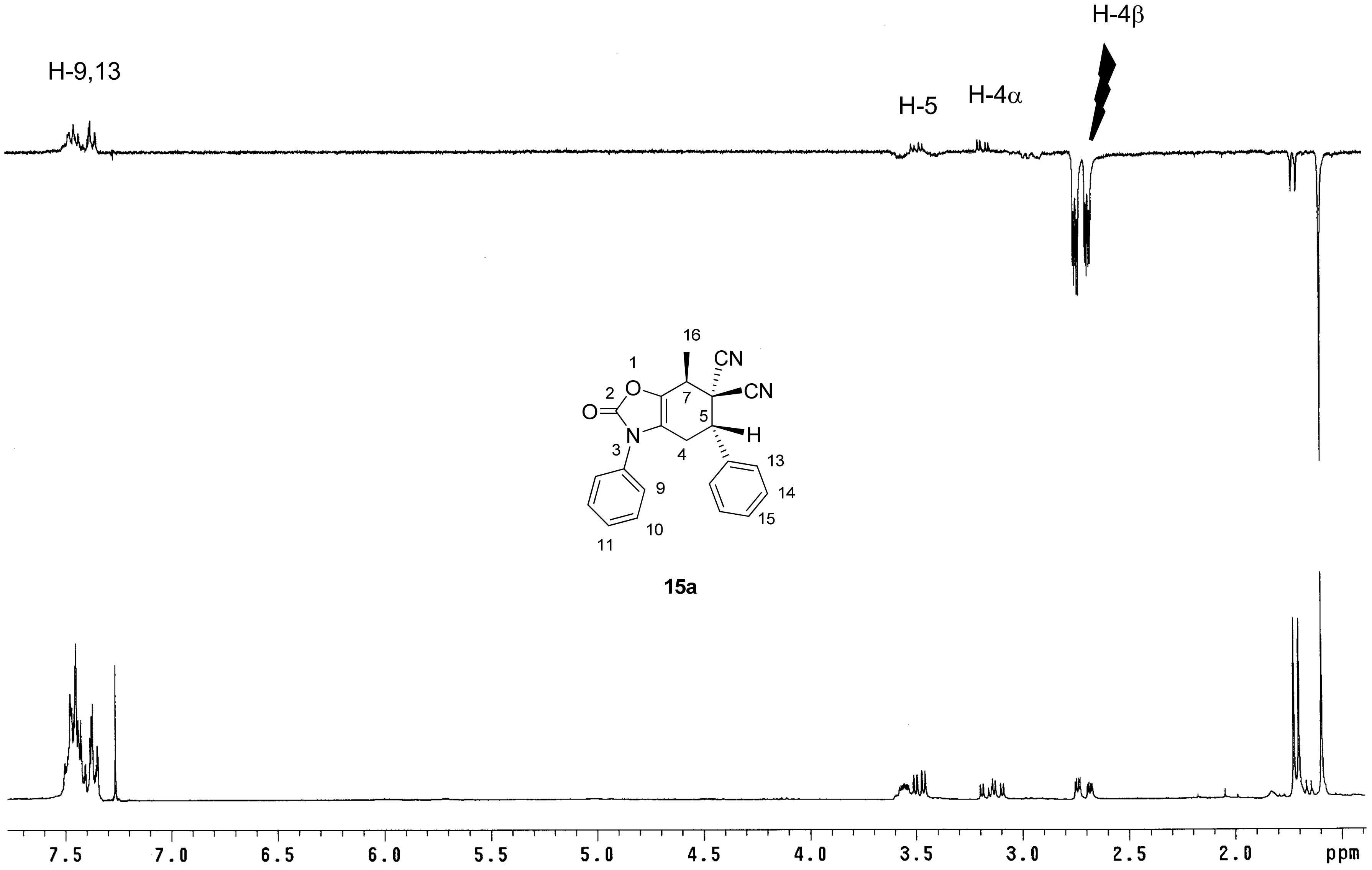

NOE experiment ($CDCl_3$) **15a**

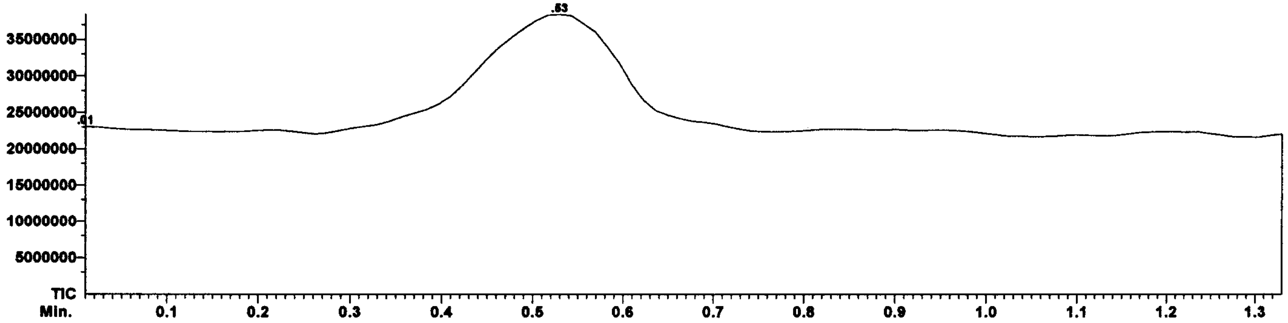

Scan: 40-43 **R.T.: .55**

Base: m/z 201; 73.4%FS TIC: 2356892 **#Ions: 3039**

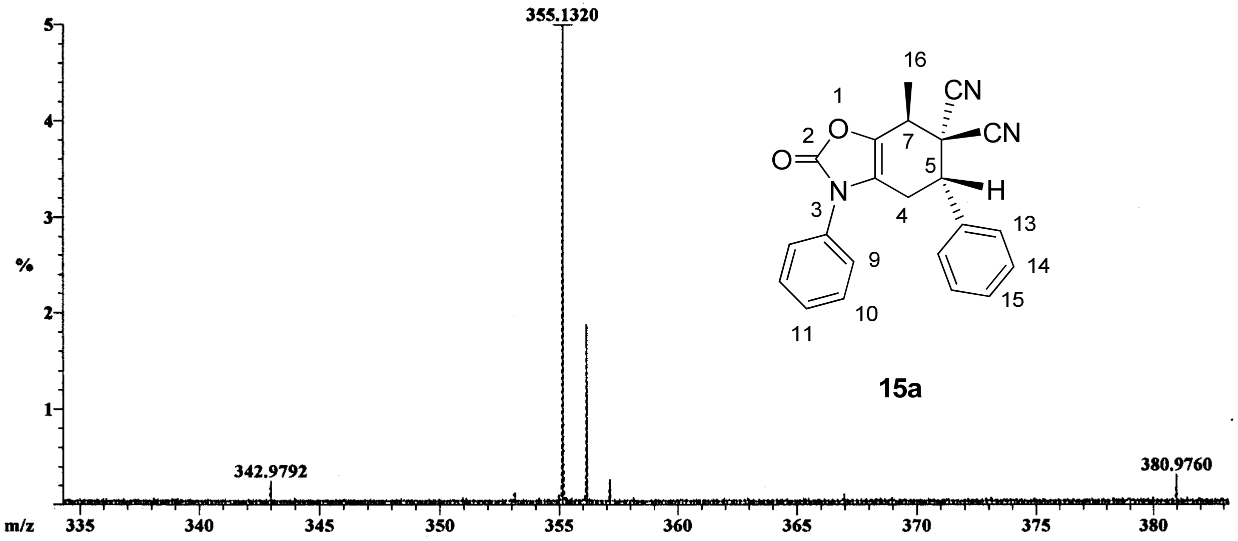

15a

Selected Isotopes : $H_{0-20}C_{0-25}N_{0-4}O_{0-3}$ **Error Limit : 1 mmu**

Measured Mass	***% Base***	***Formula***	***Calculated Mass***	***Error***
355.13199	6.2%	$C_{22}H_{17}N_3O_2$	355.13208	-0.1

HRMS **15a**

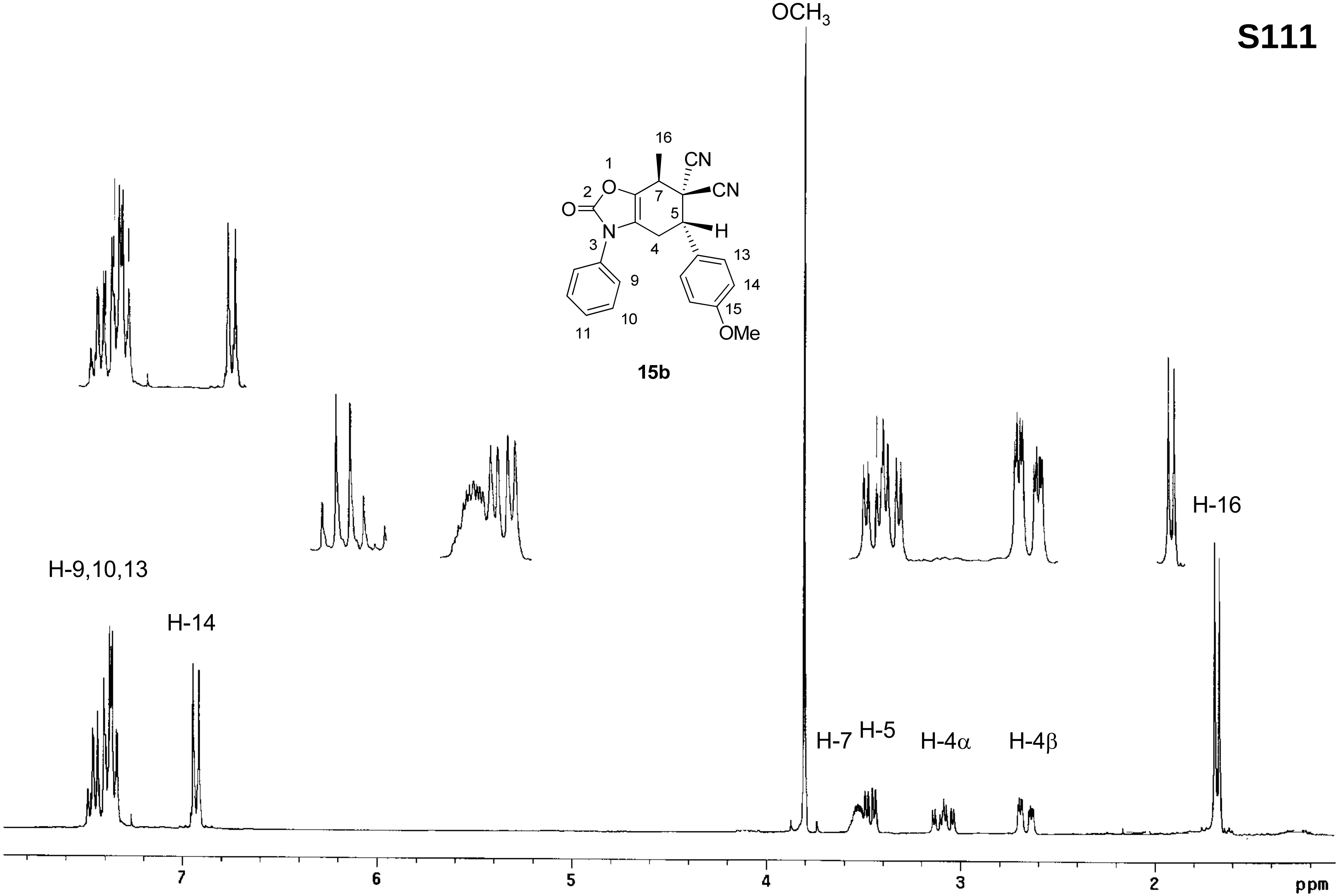

^{1}H NMR ($CDCl_3$) **15b**

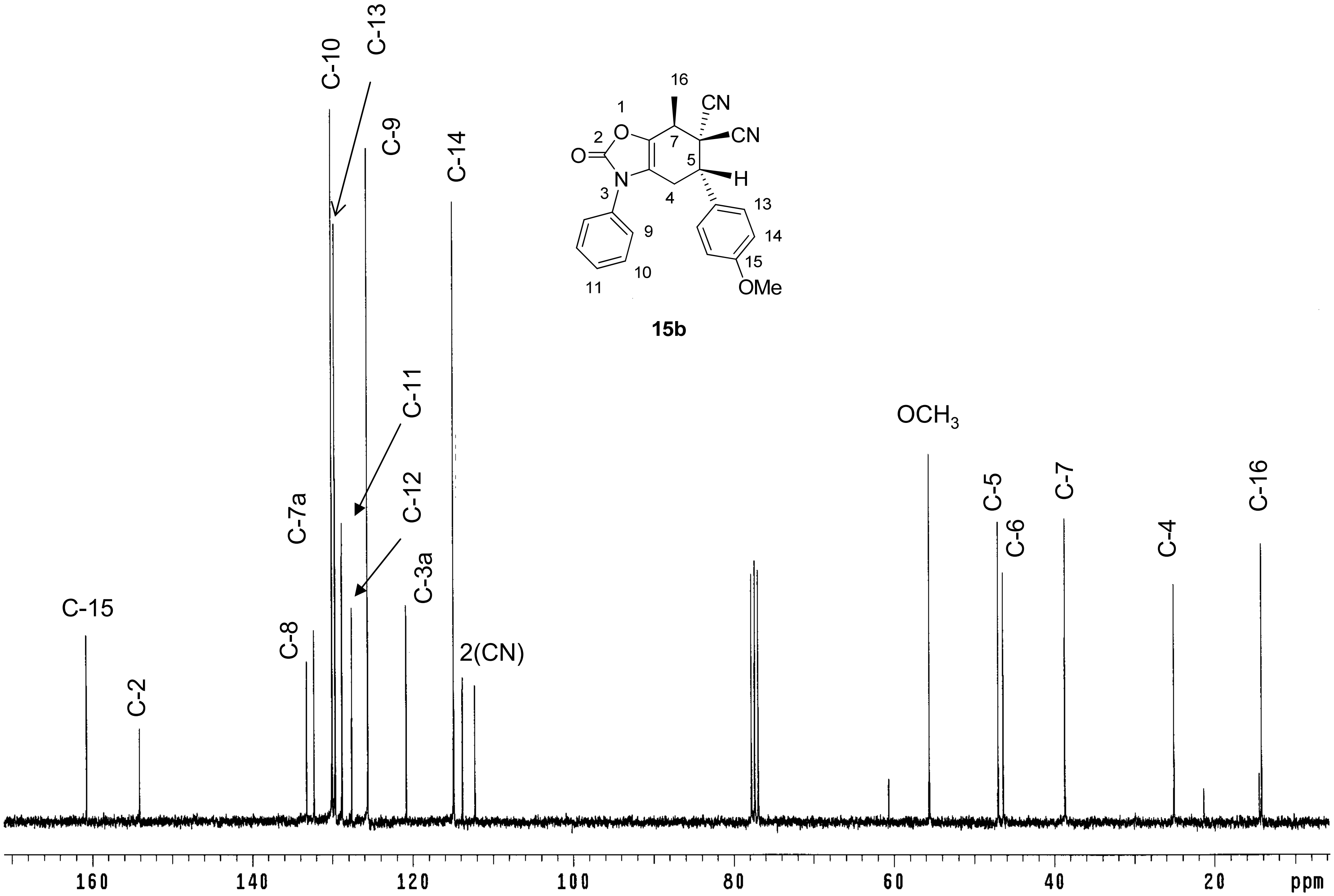

^{13}C NMR ($CDCl_3$) **15b**

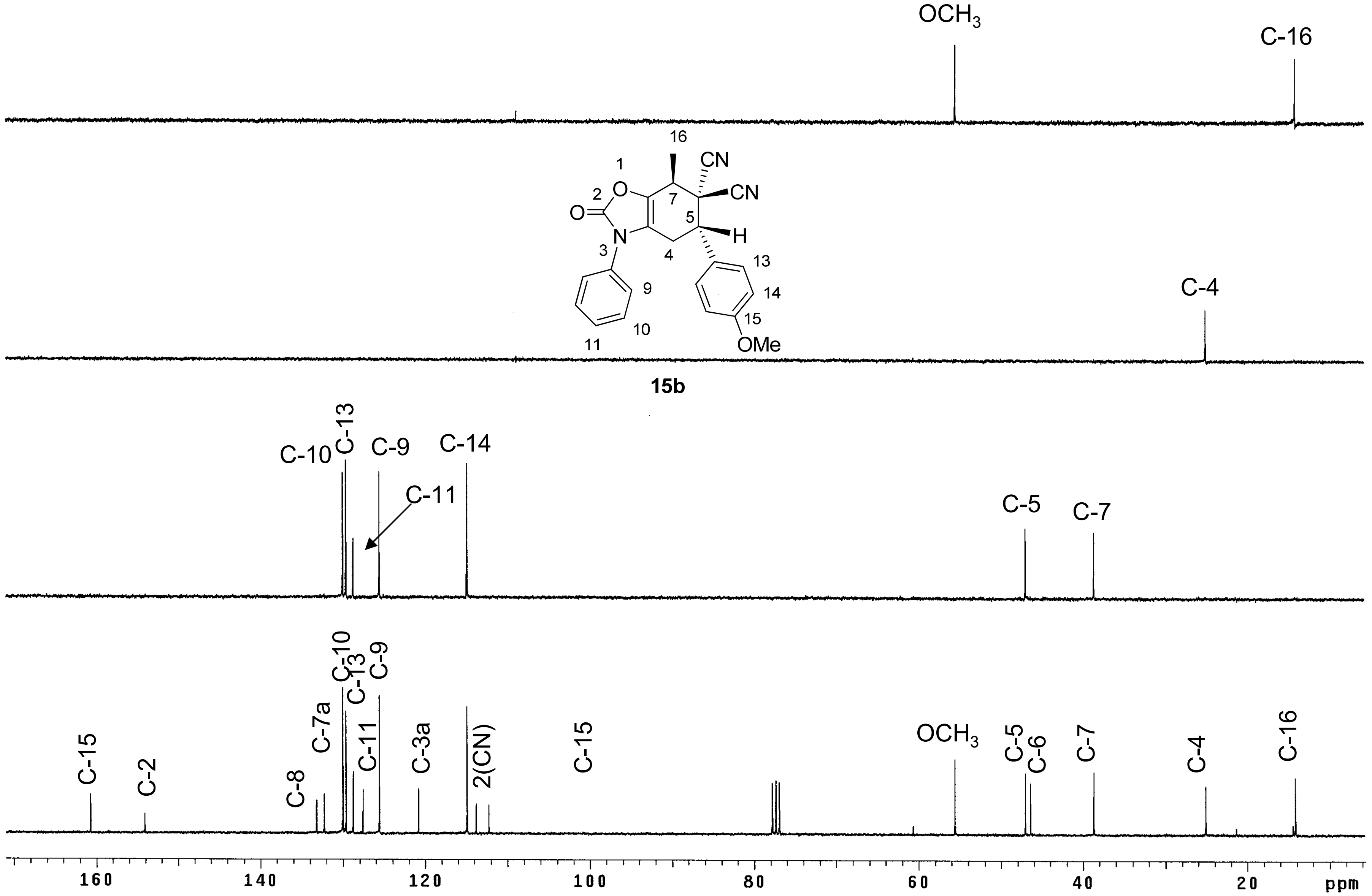

DEPT ($CDCl_3$) **15b**

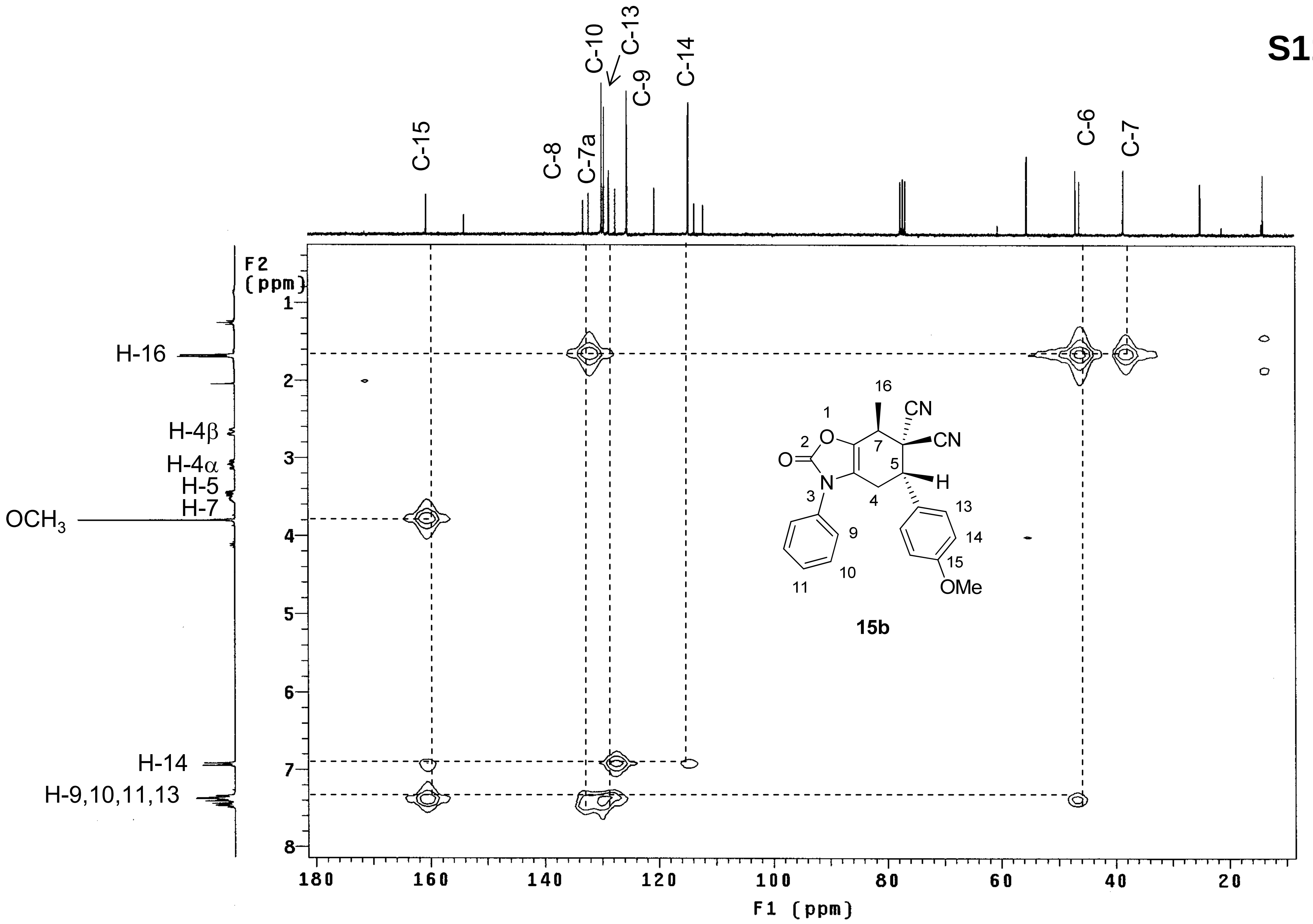

HMBC experiment ($CDCl_3$) **15b**

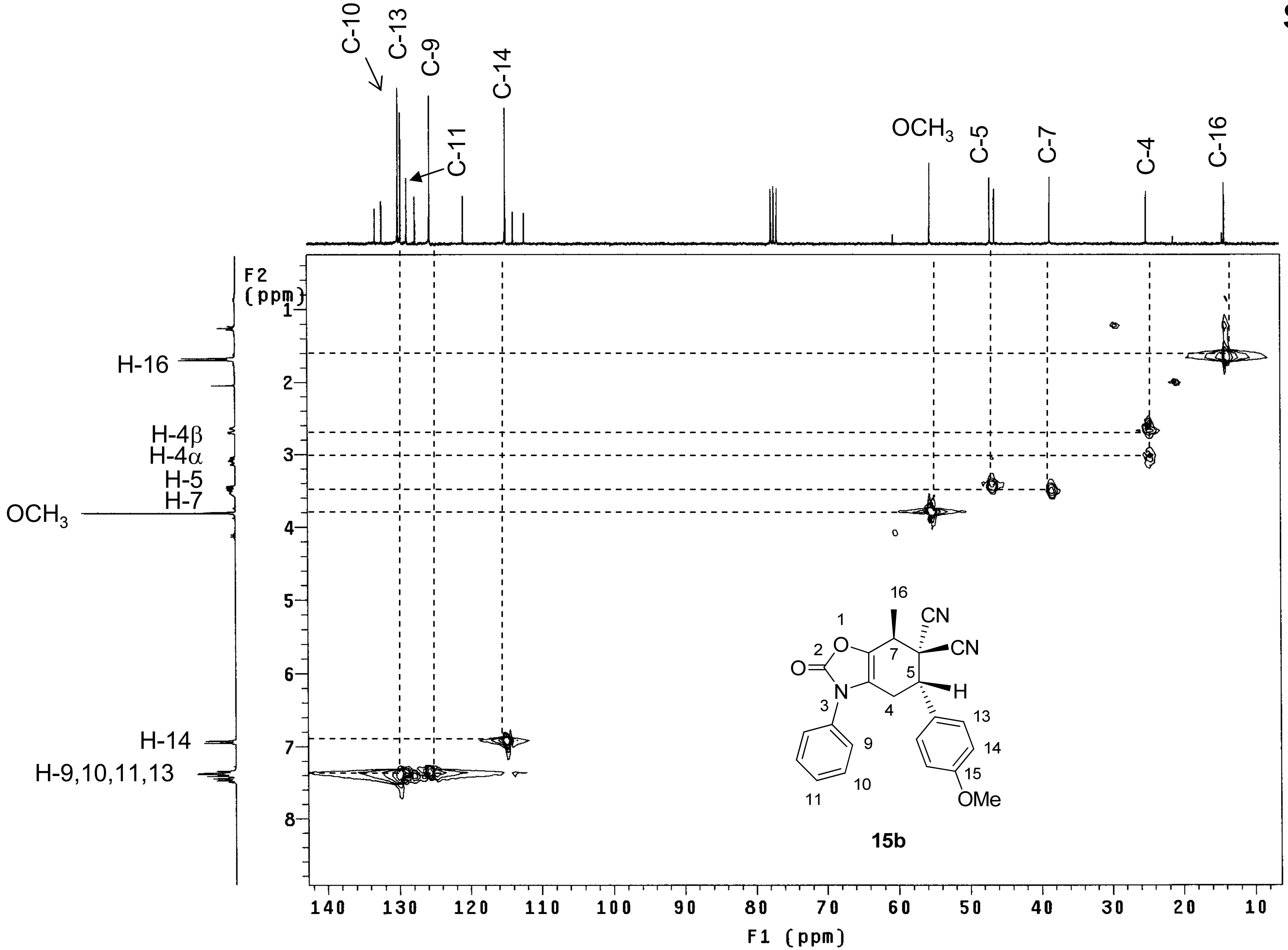

HSQC experiment ($CDCl_3$) **15b**

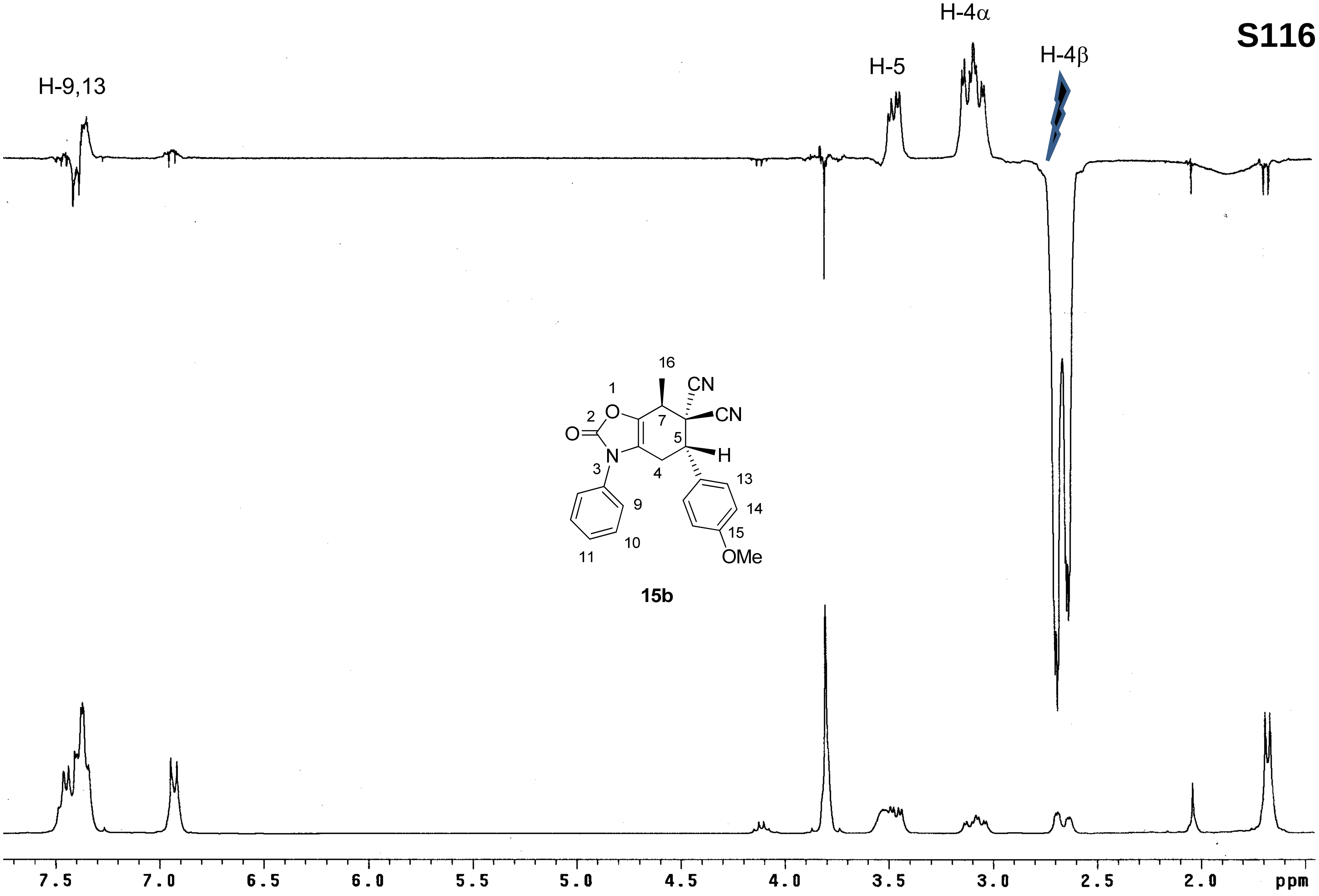

NOE experiment ($CDCl_3$) **15b**

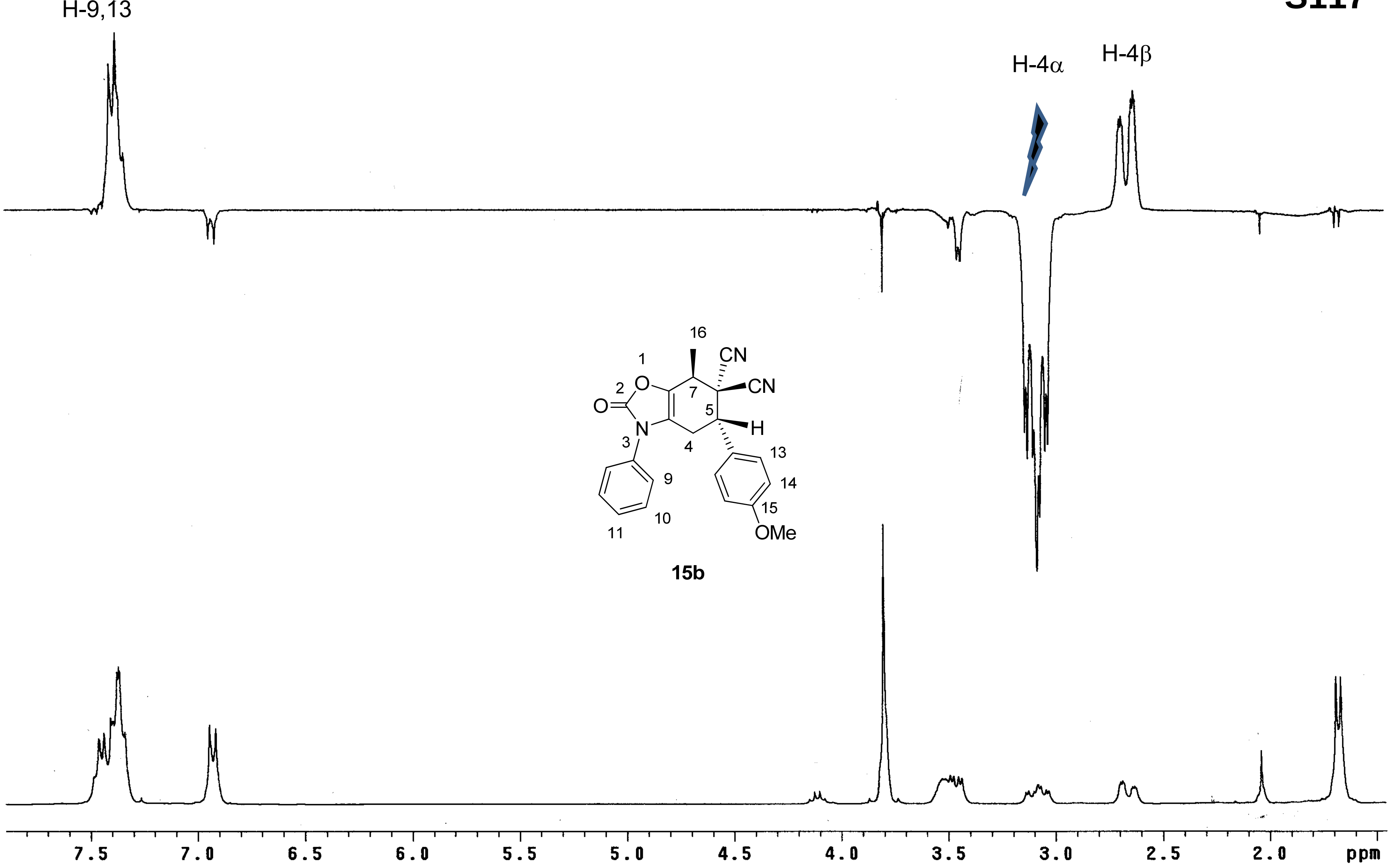

15b

NOE experiment ($CDCl_3$) **15b**

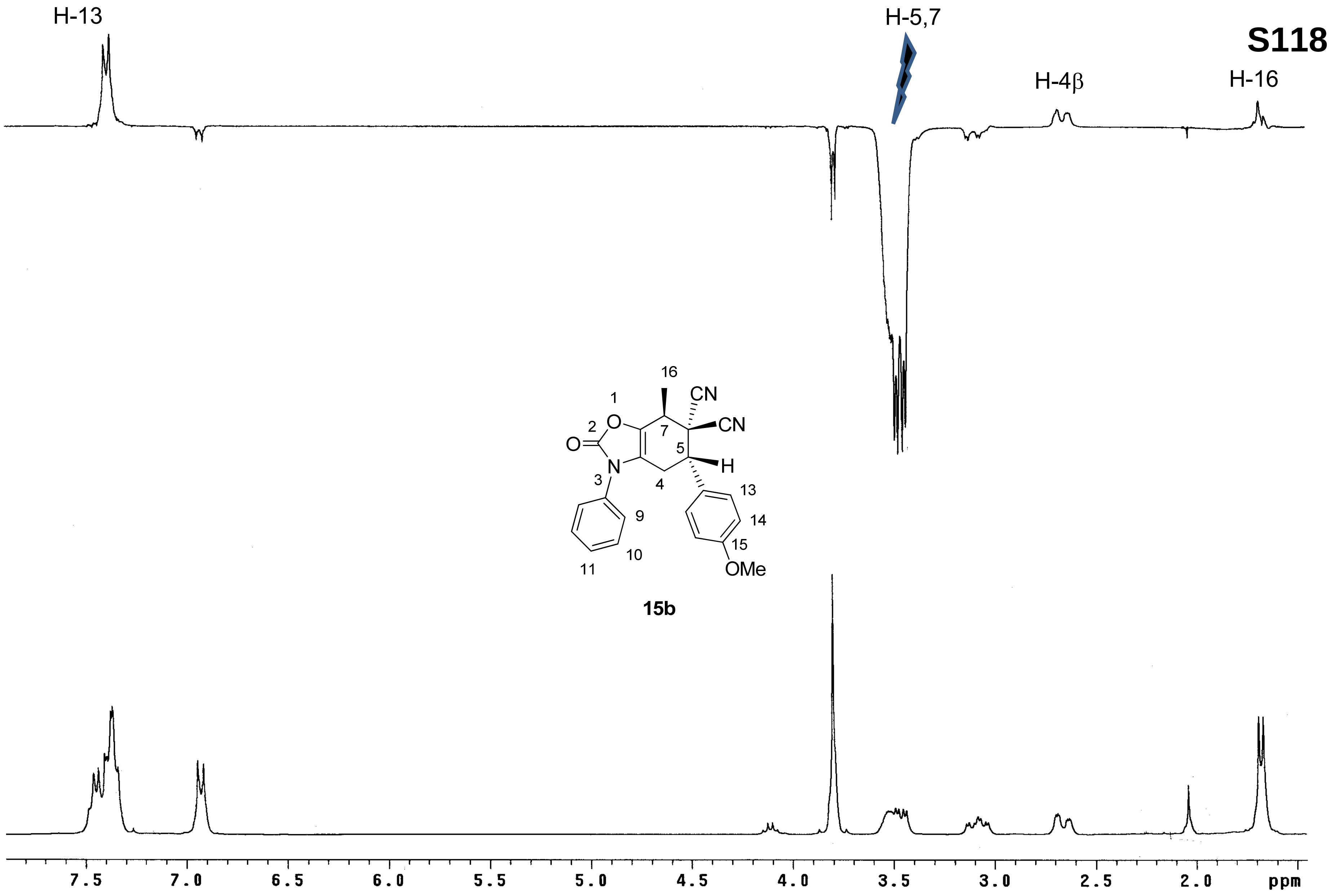

NOE experiment ($CDCl_3$) **15b**

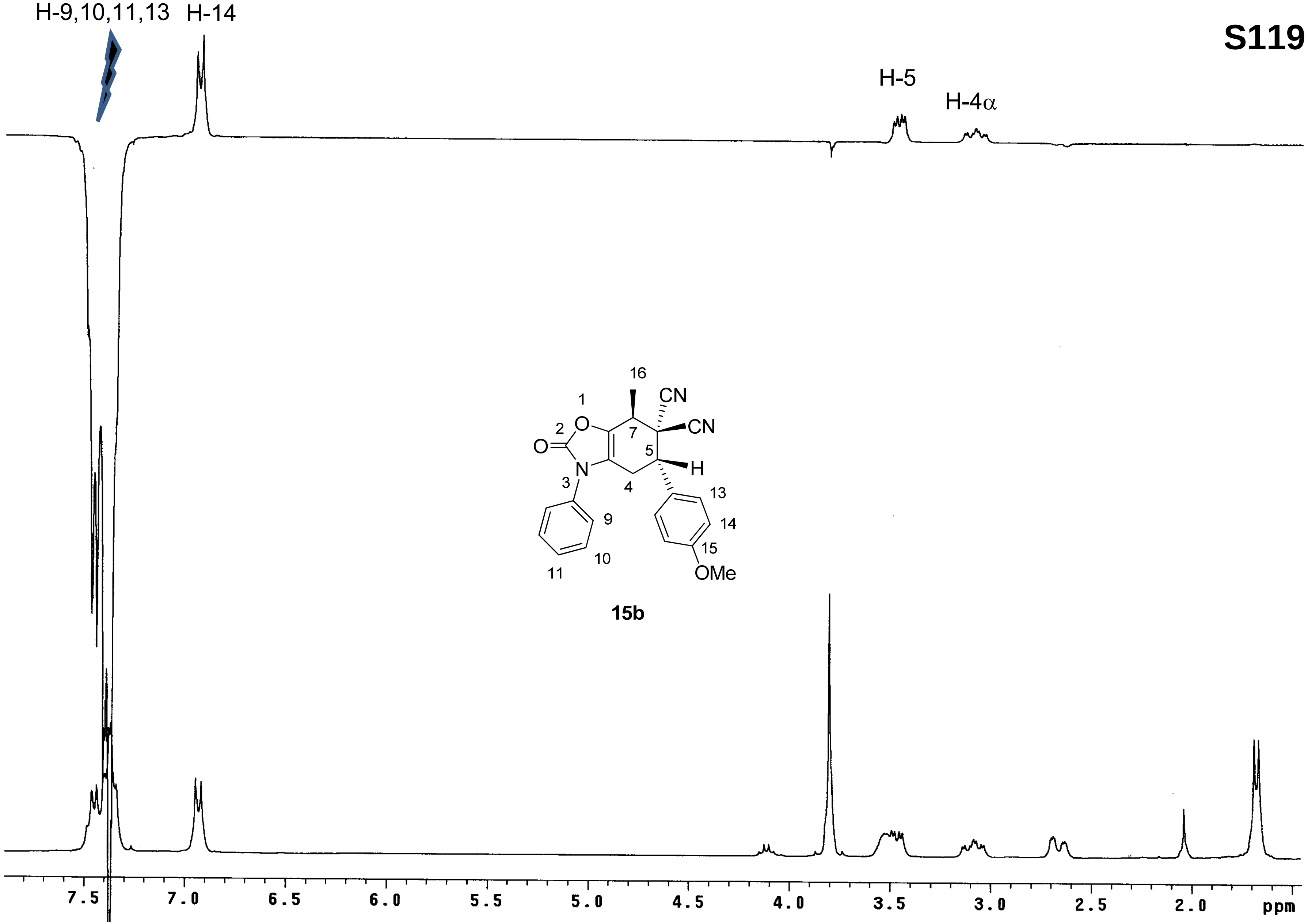

NOE experiment ($CDCl_3$) **15b**

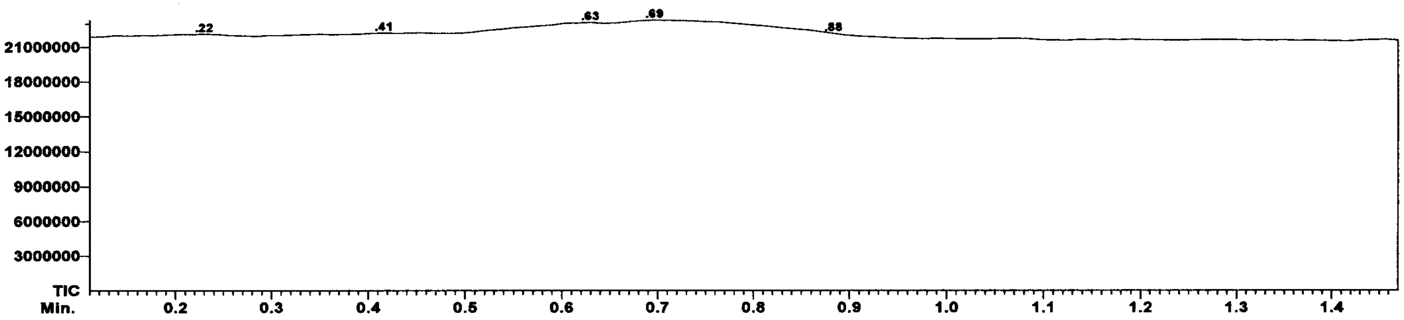

Scan: 60-67 **R.T.: .85**

Base: m/z 385; 2.5%FS TIC: 1193206 **#Ions: 2942**

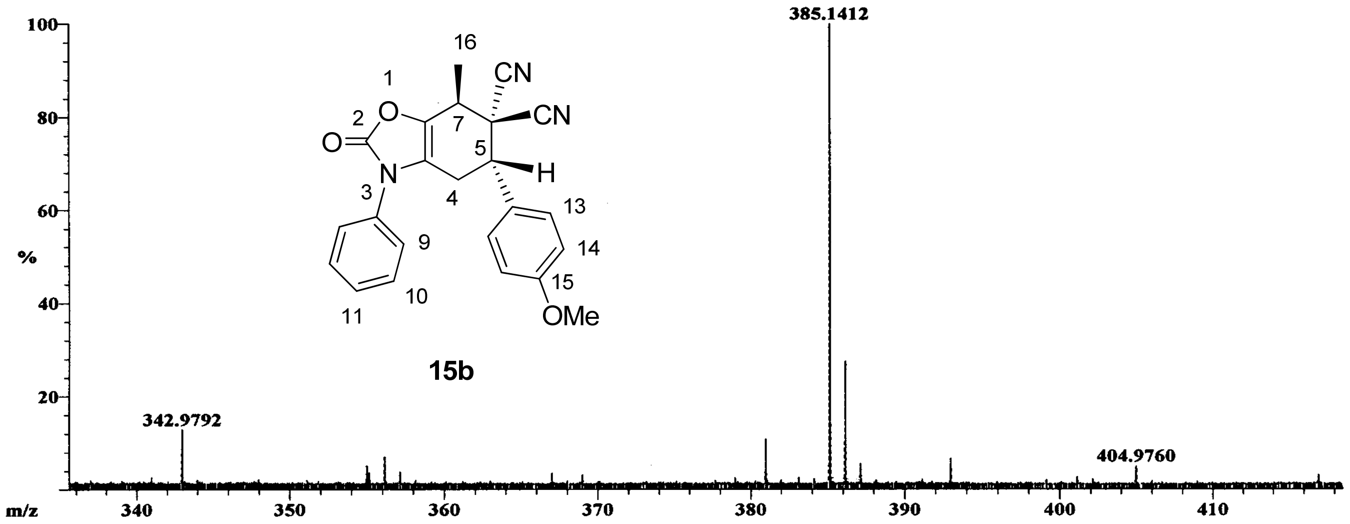

Selected Isotopes : $H_{0-25}C_{0-25}N_{0-5}O_{0-3}$ **Error Limit : 5 mmu**

Measured Mass	***% Base***	***Formula***	***Calculated Mass***	***Error***
**385.14116**	**100.0%**	**$C_{23}H_{19}N_3O_3$**	**385.14265**	**-1.5**

HRMS **15b**

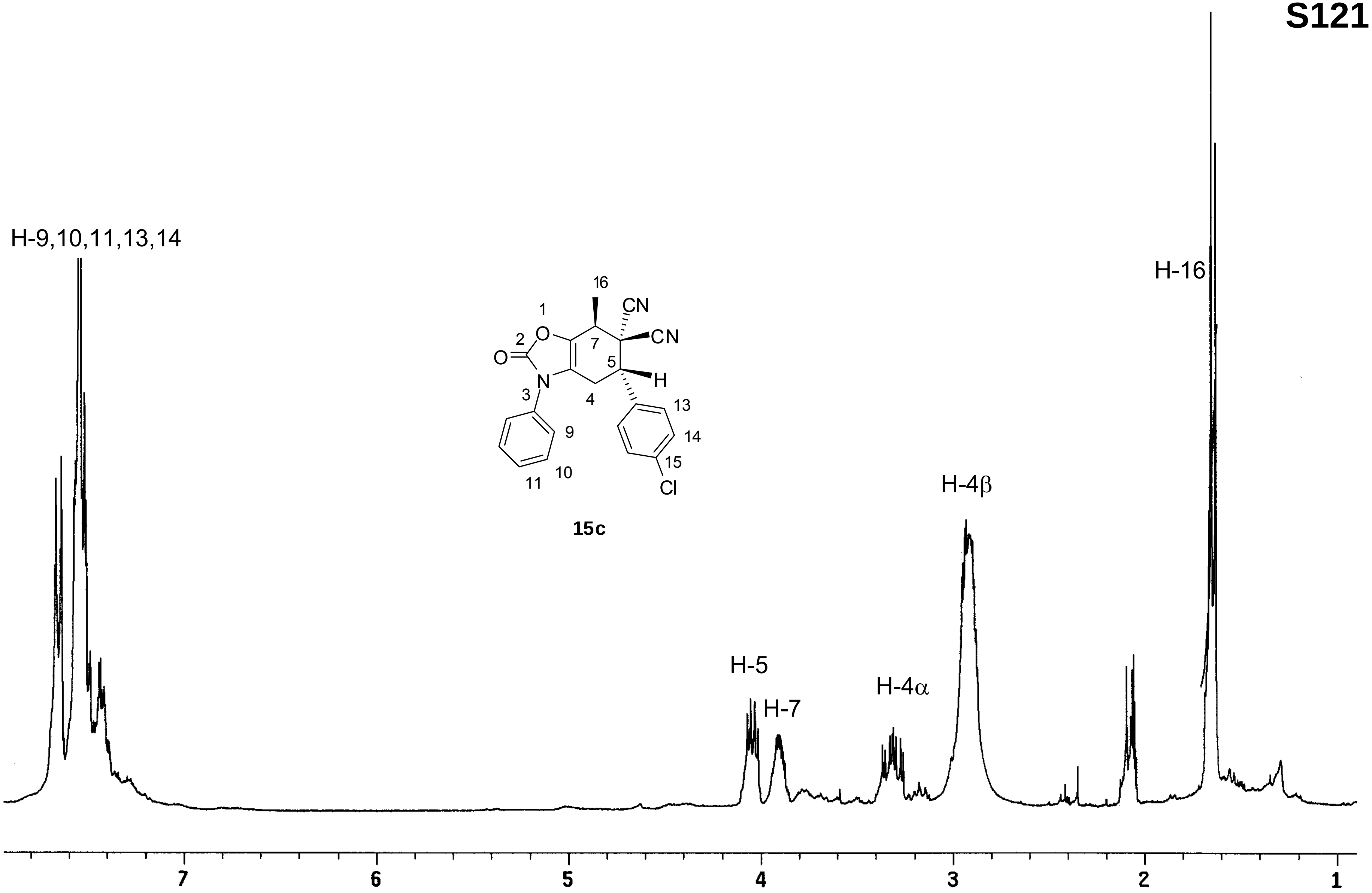

^{1}H NMR ($CDCl_3$) **15c**

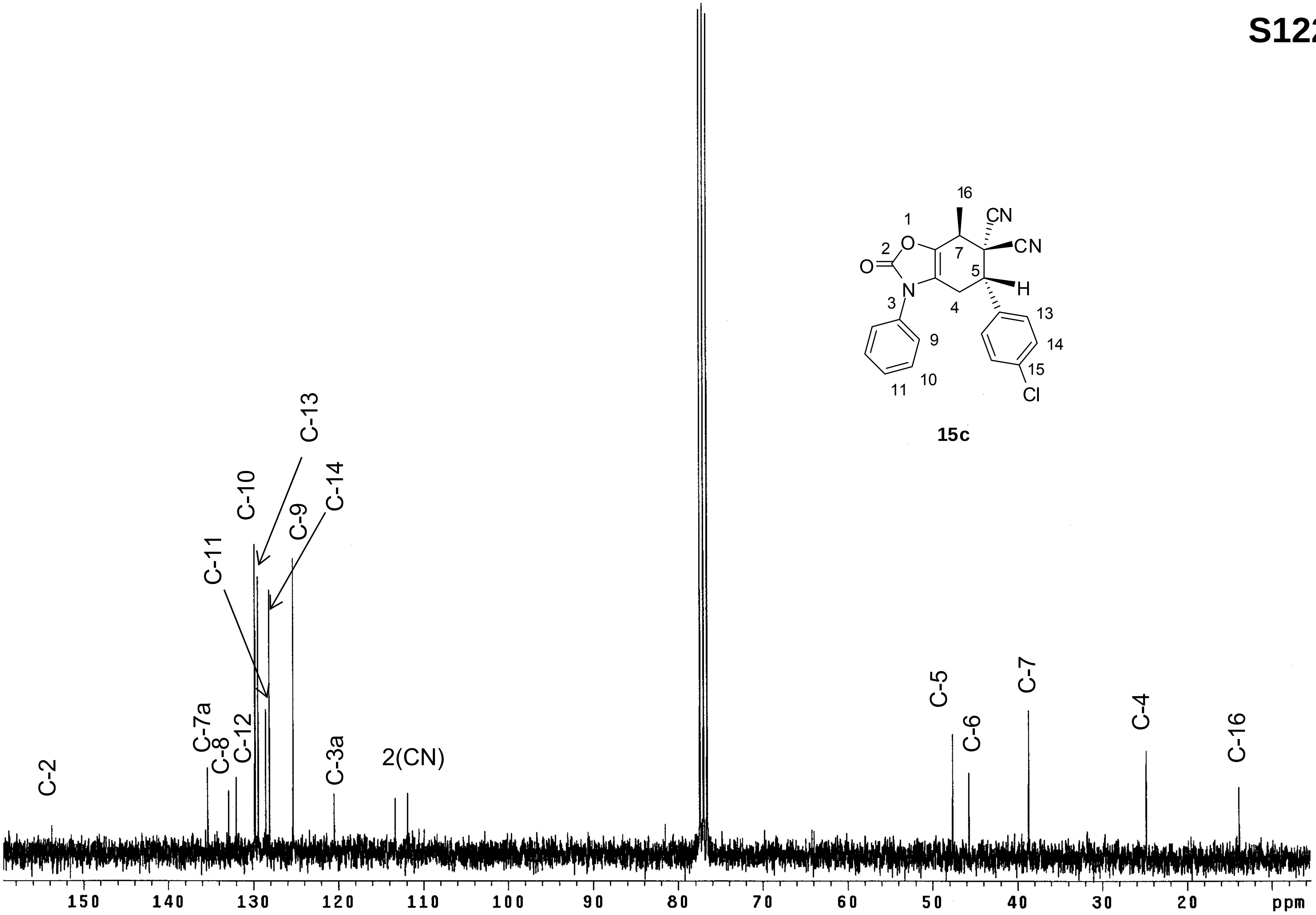

15c

^{13}C NMR (CDCl$_3$) **15c**

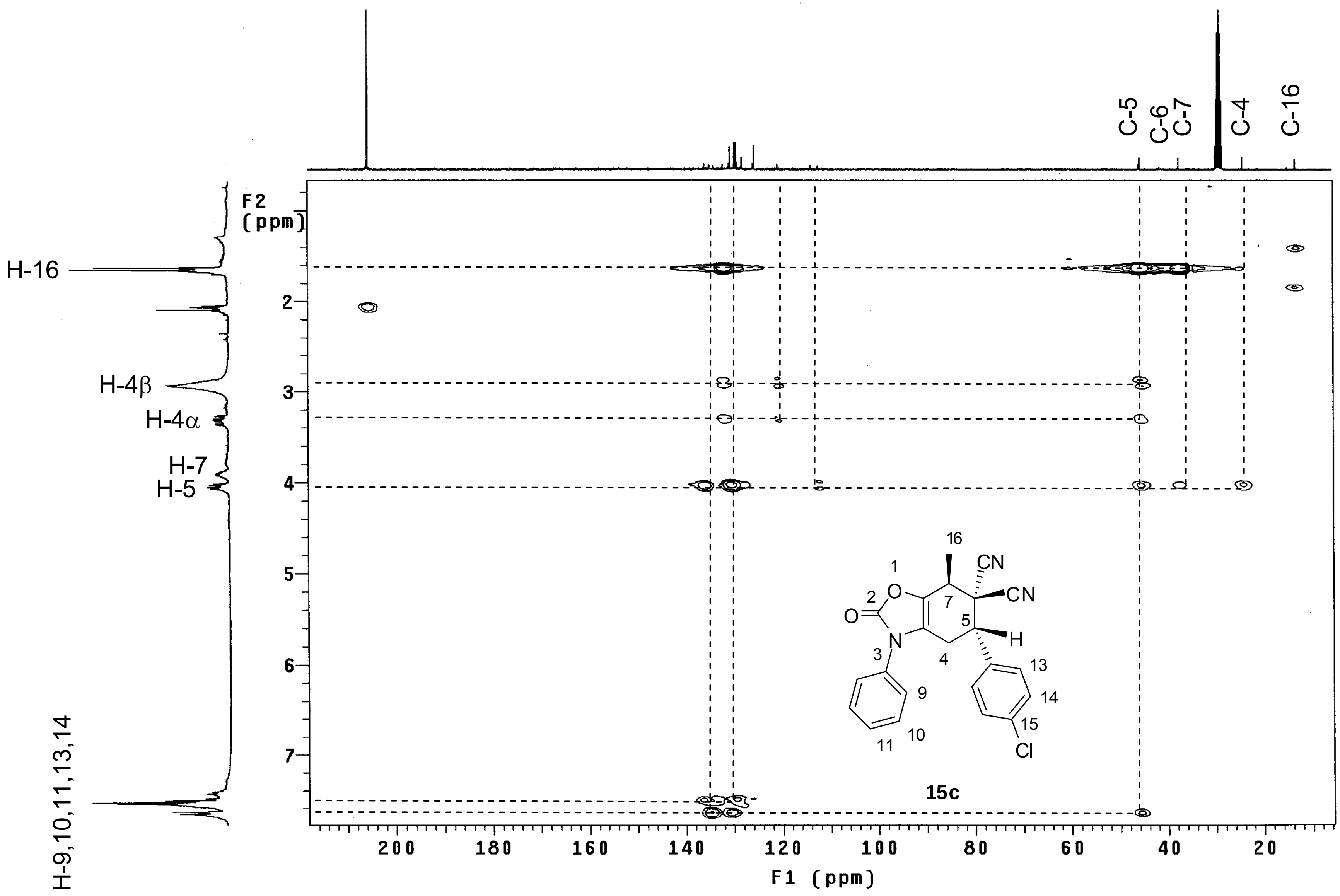

HMBC experiment ($CDCl_3$) **15c**

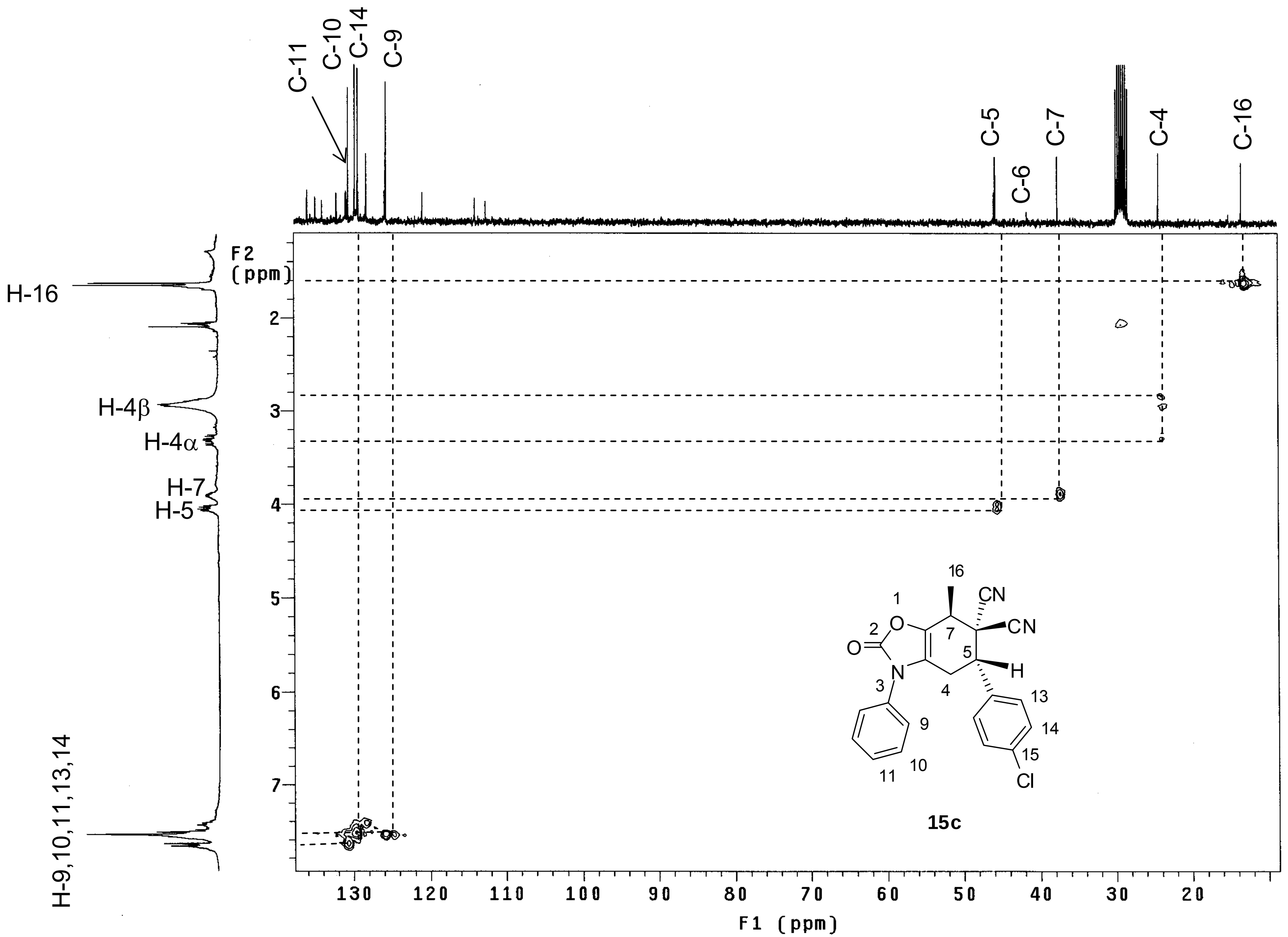

HSQC experiment ($CDCl_3$) **15c**

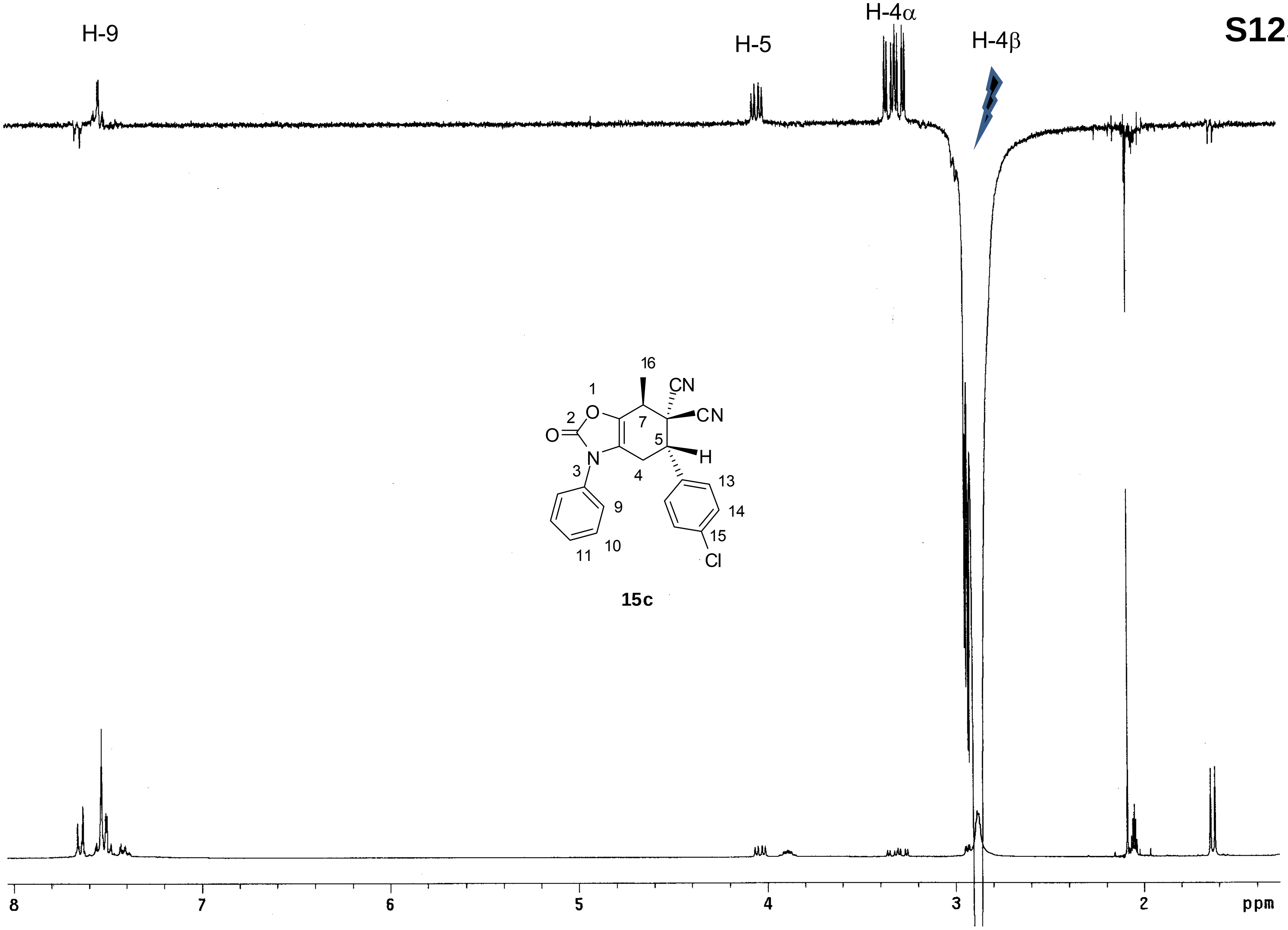

NOE experiment ($CDCl_3$) **15c**

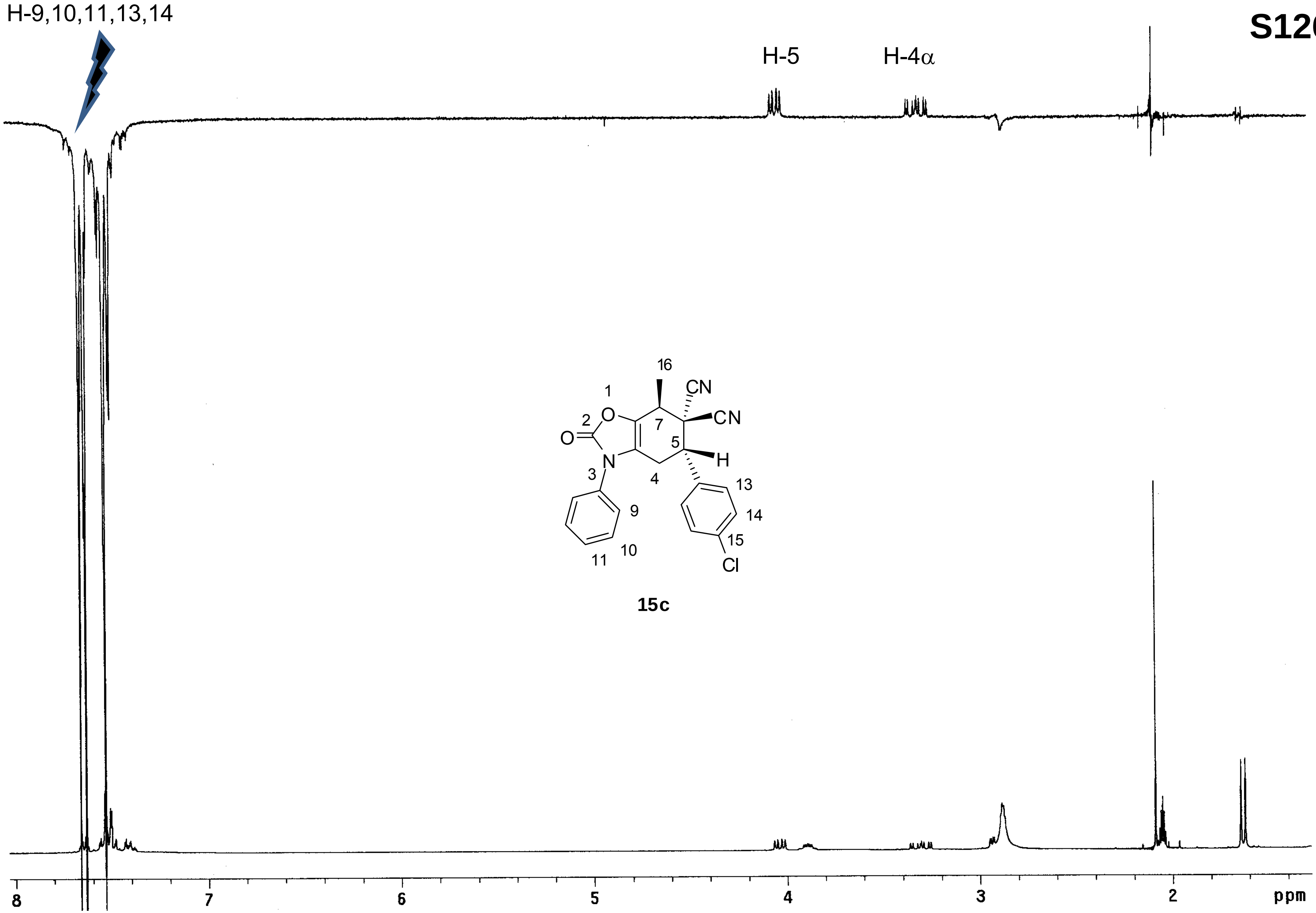

NOE experiment ($CDCl_3$) **15c**

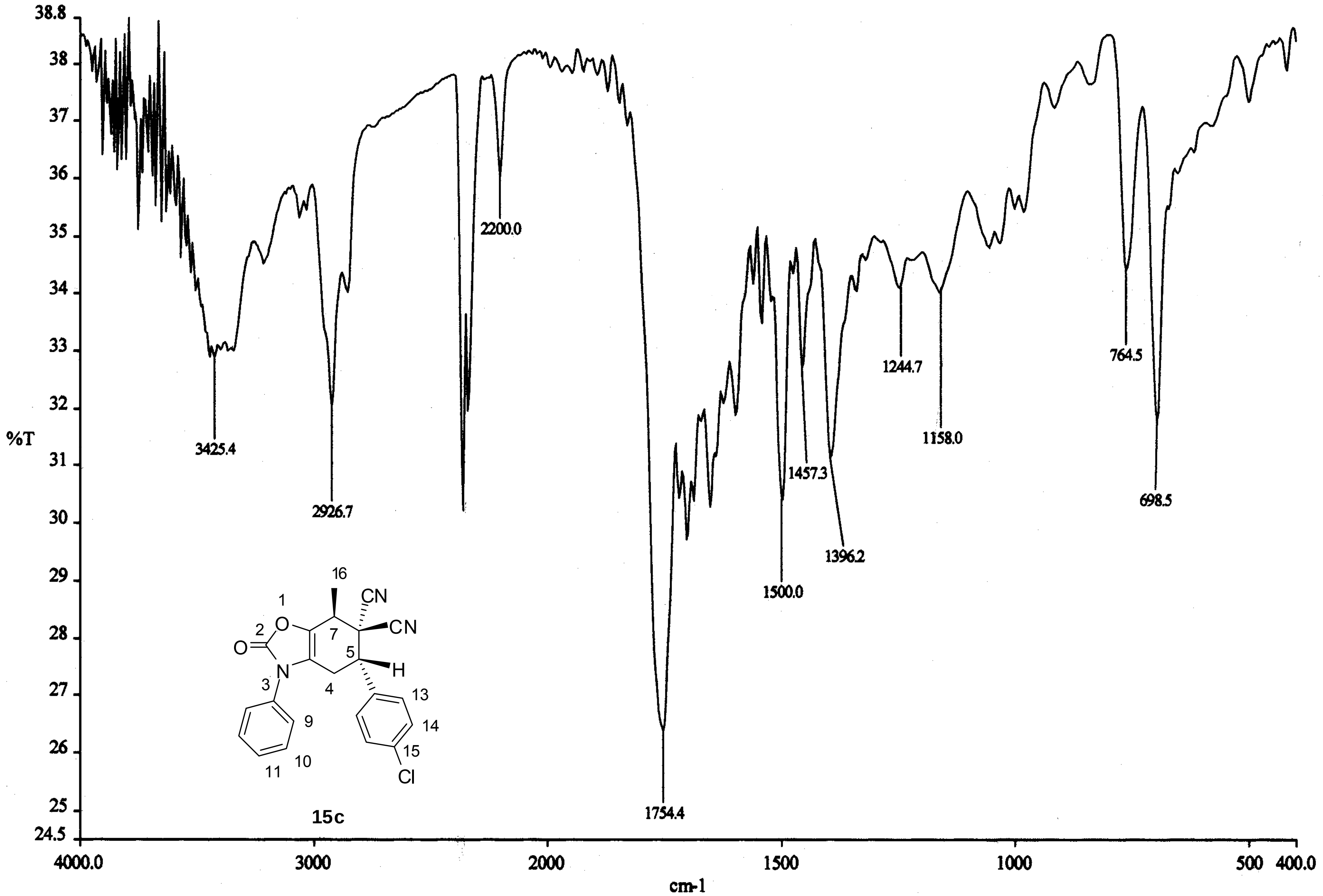
38.8
38
37
36
35
34
33
32
31
30
29
28
27
26
25
24.5
%T
3425.4
2926.7
2200.0
1754.4
1500.0
1457.3
1396.2
1244.7
1158.0
764.5
698.5
4000.0
3000
2000
1500
1000
500
400.0
cm-1
16
1
CN
CN
2
O
O
7
5
H
N
3
4
13
9
14
15
Cl
10
11
15c

FT-IR (KBr) **15c**

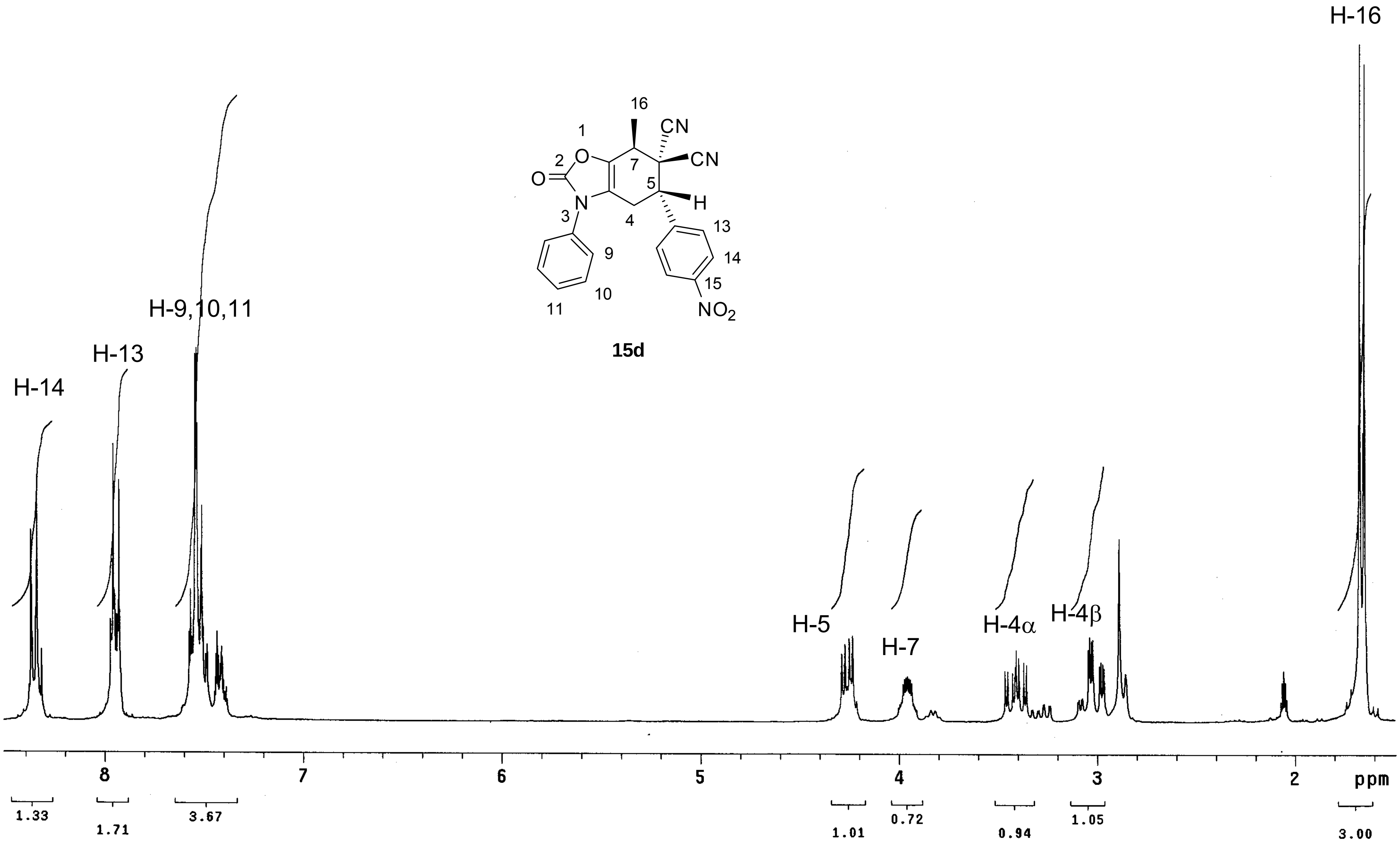

^{1}H NMR ($CDCl_3$) **15d**

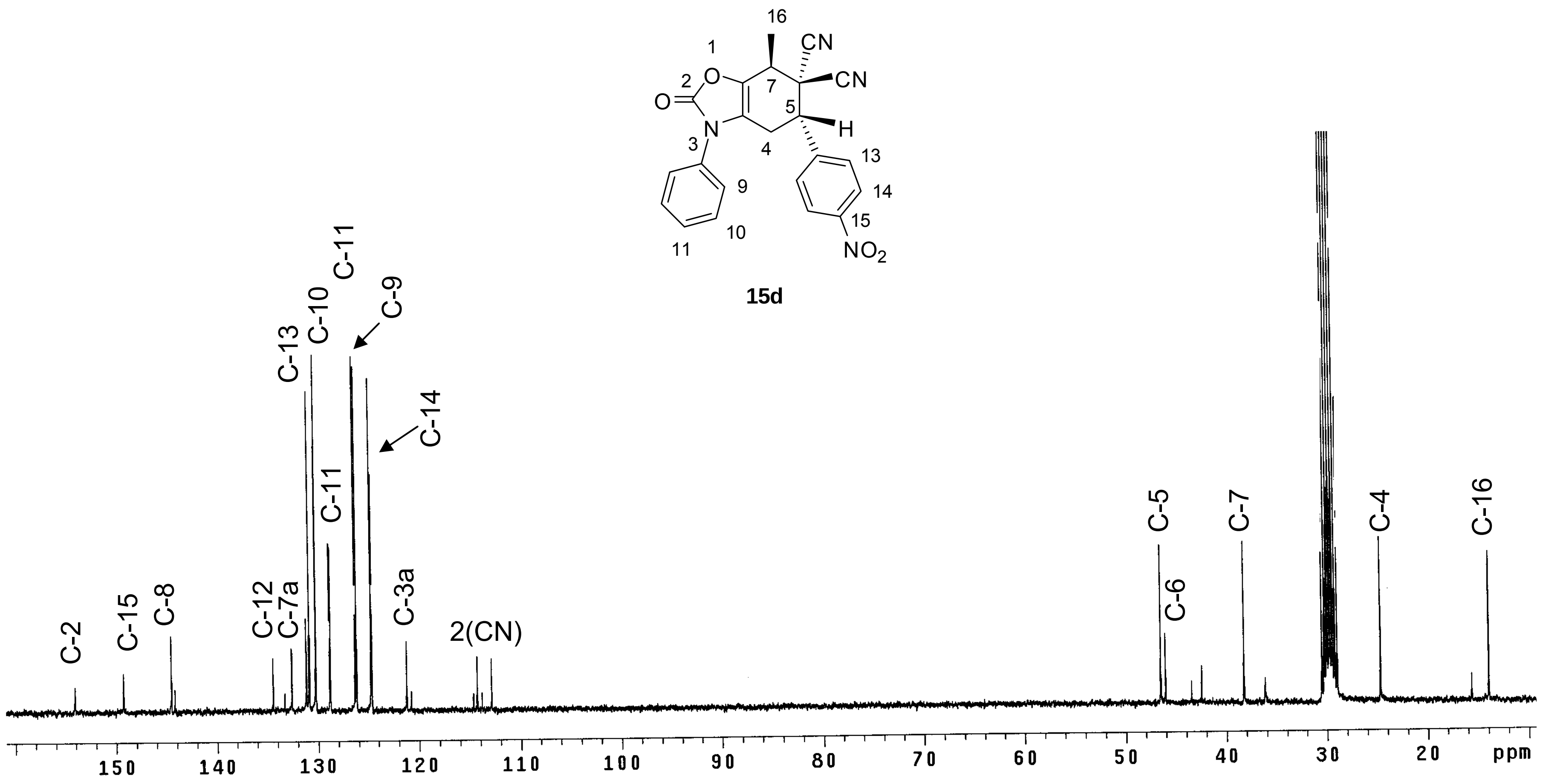

^1H NMR ($CDCl_3$) **15d**

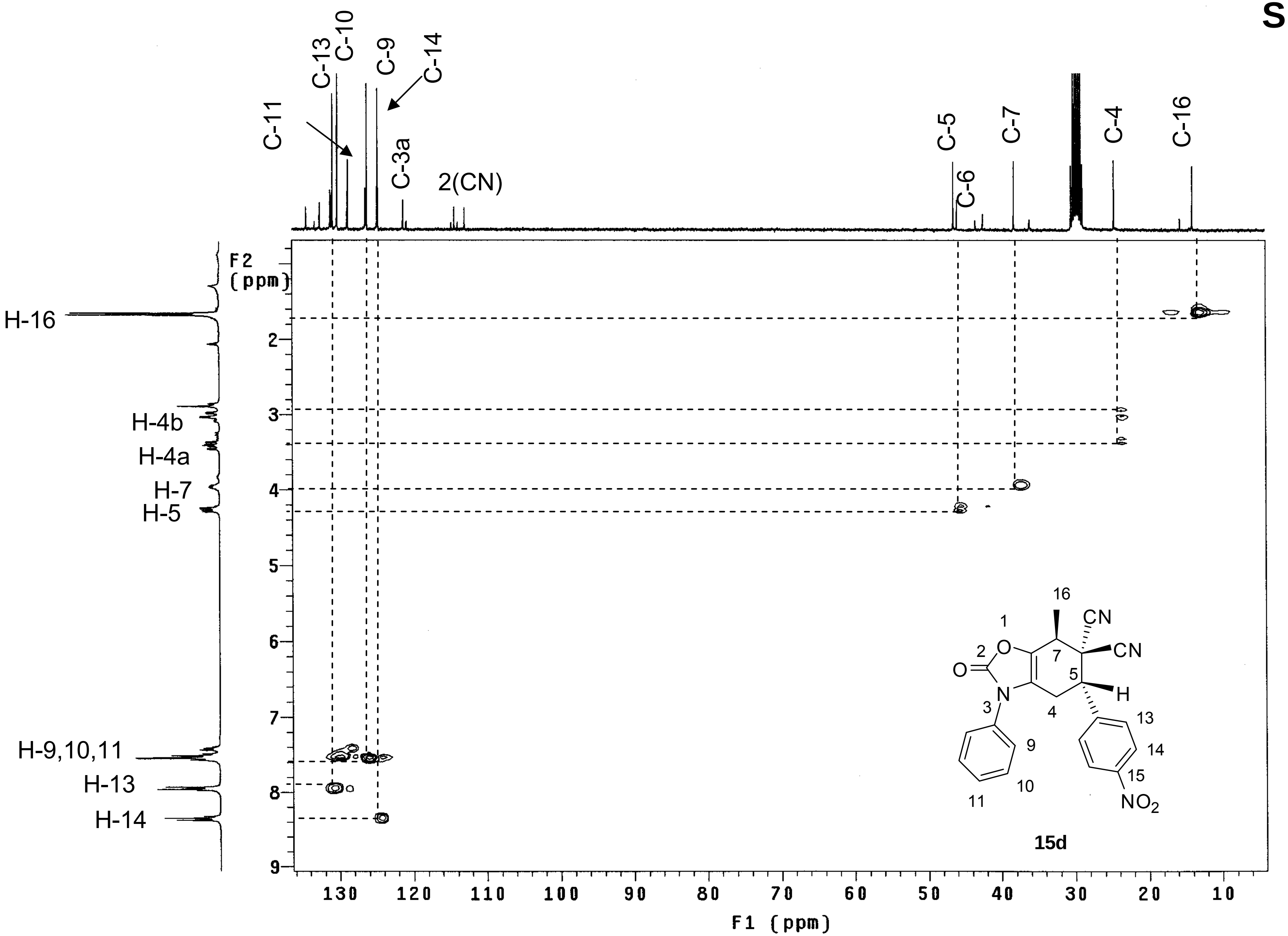

HSQC experiment ($CDCl_3$) **15d**

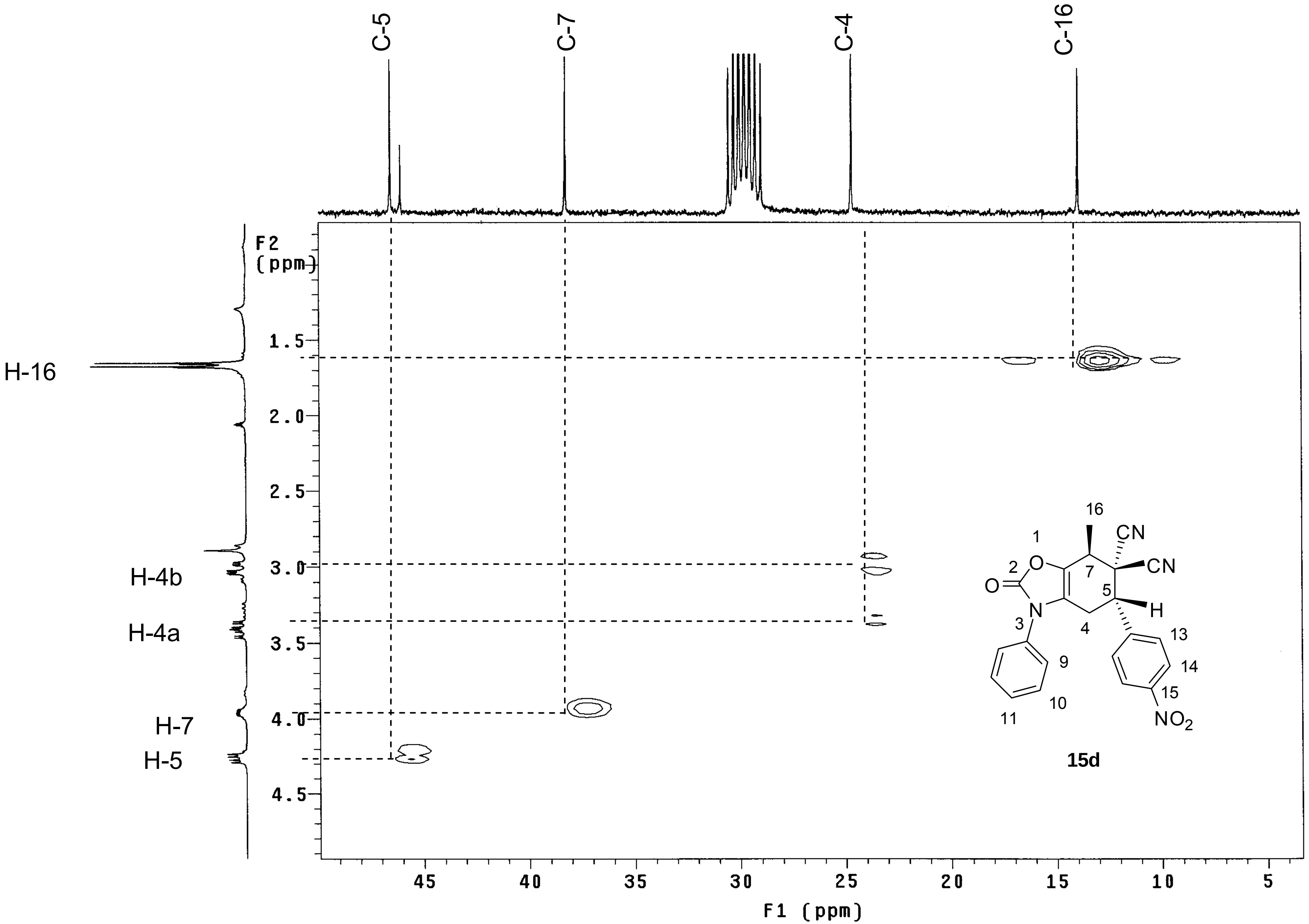

HSQC experiment ($CDCl_3$) **15d**

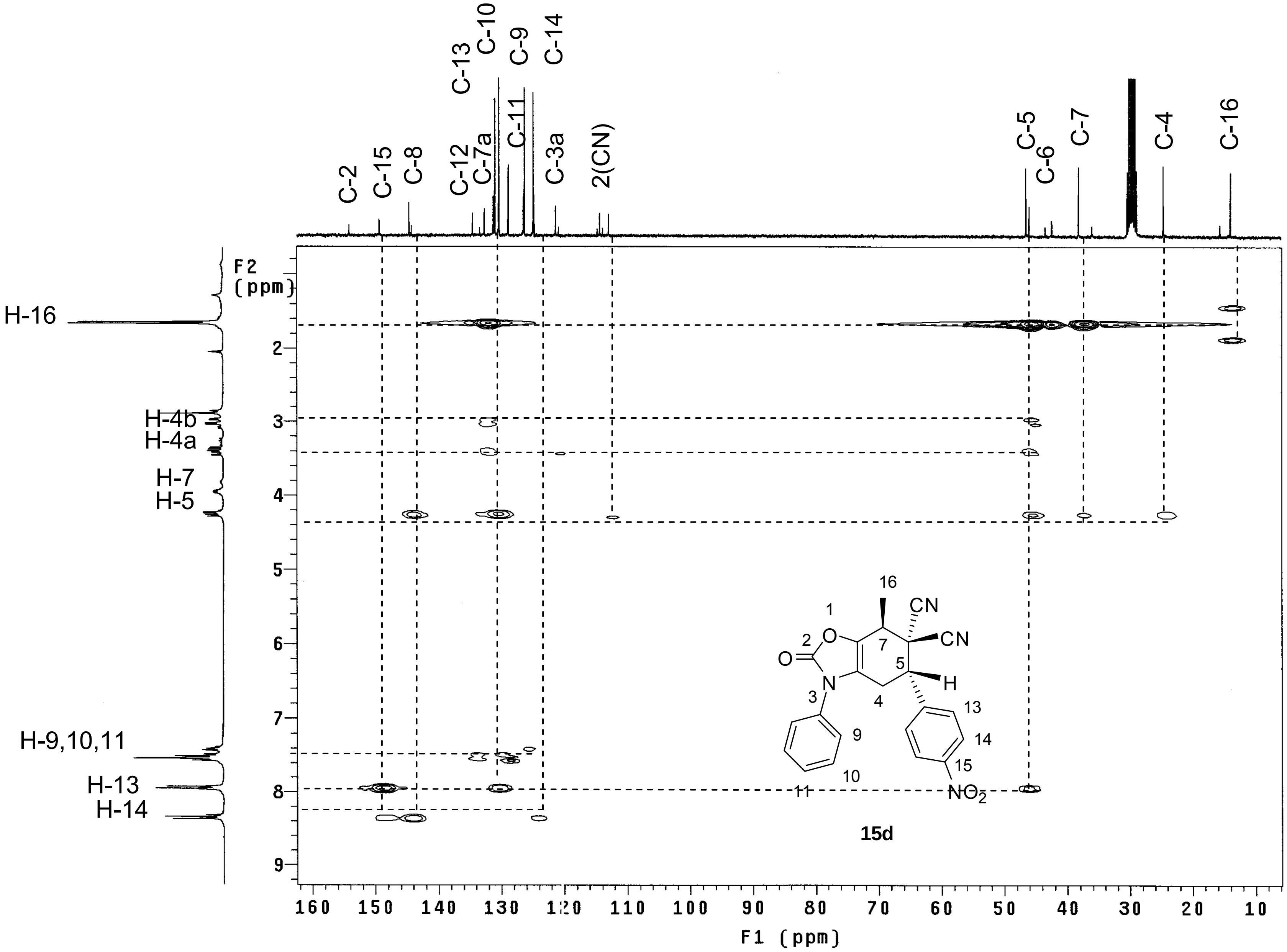

HMBC experiment ($CDCl_3$) **15d**

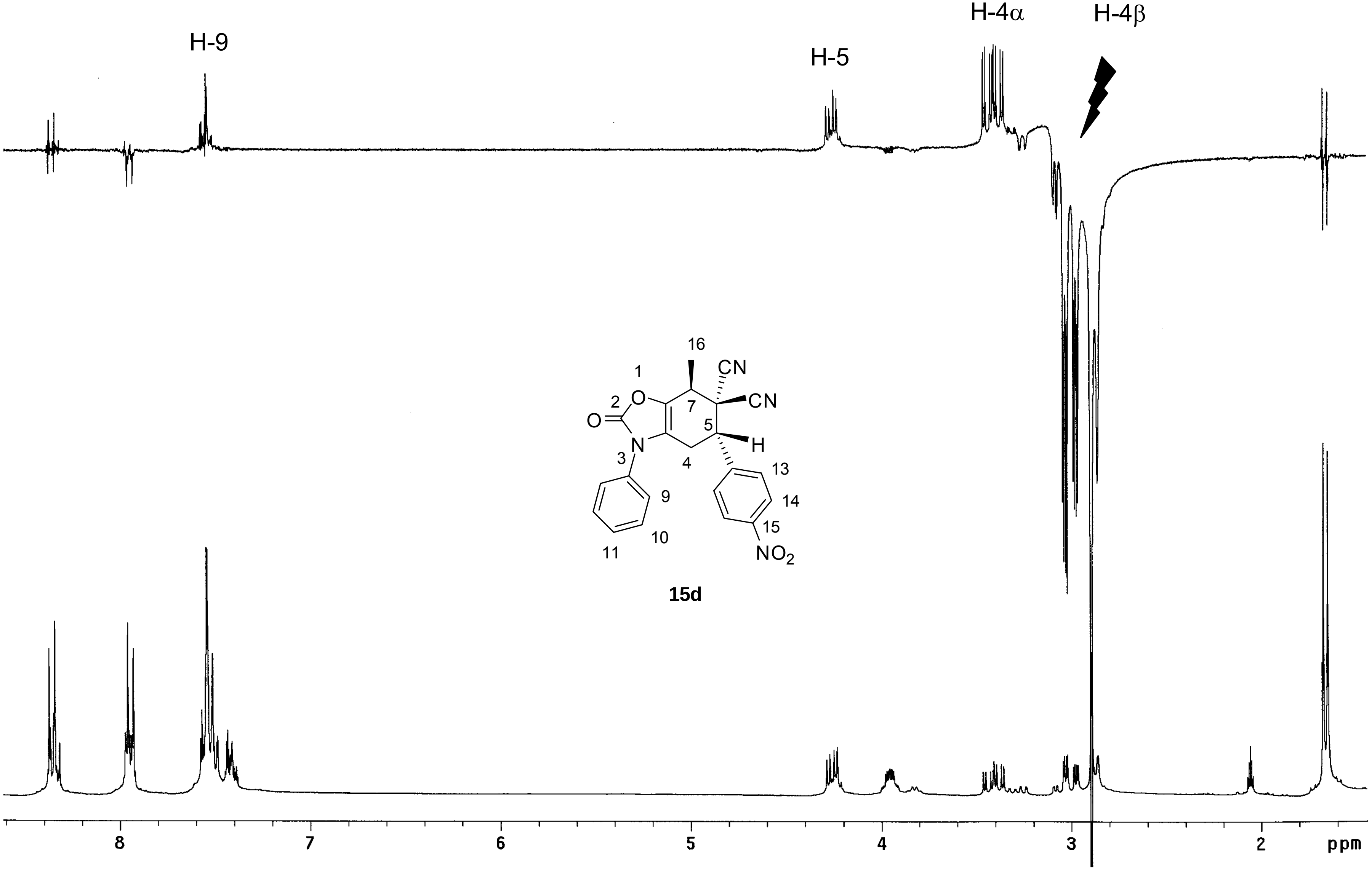

NOE experiment ($CDCl_3$) **15d**

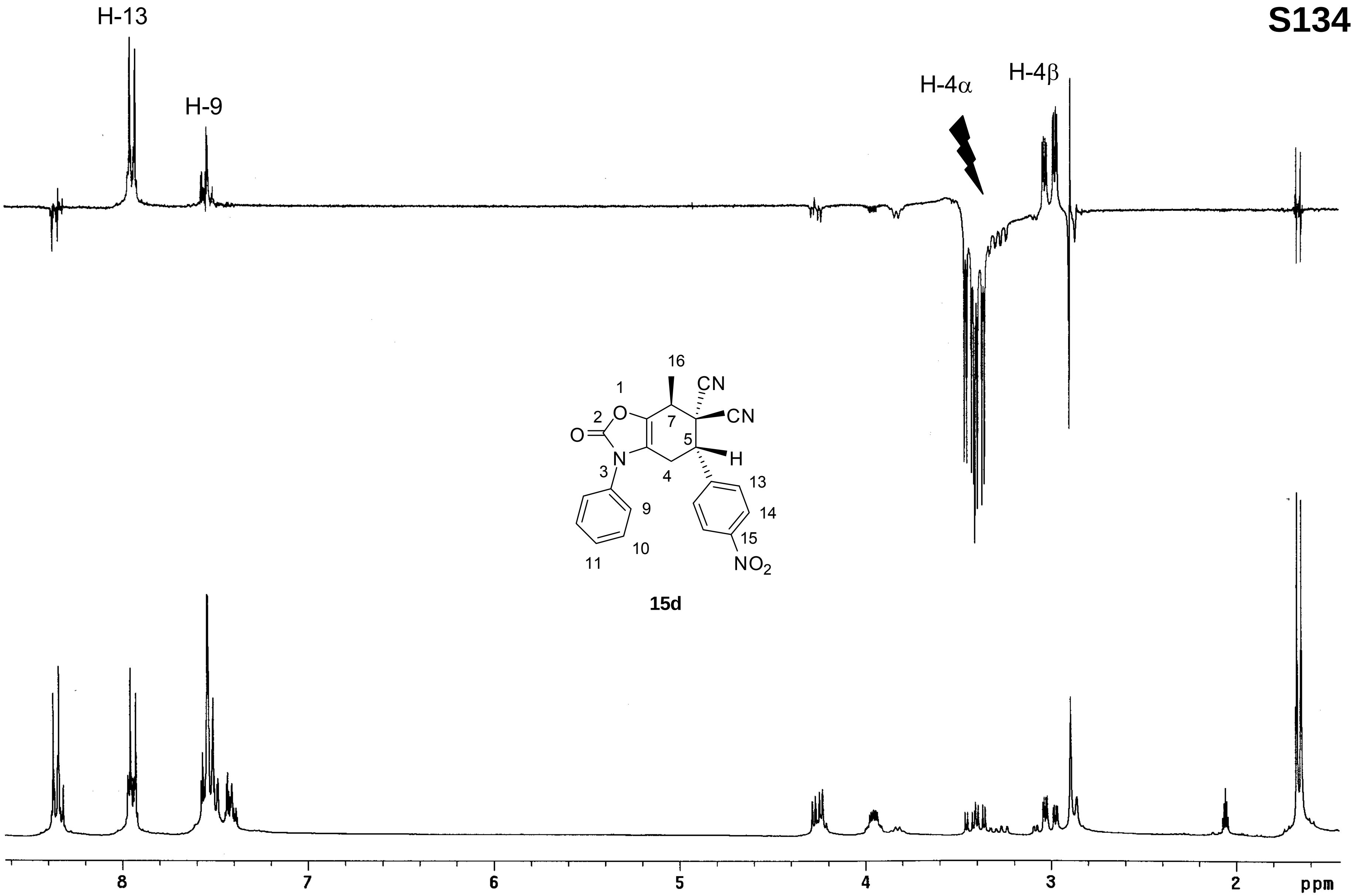

NOE experiment ($CDCl_3$) **15d**

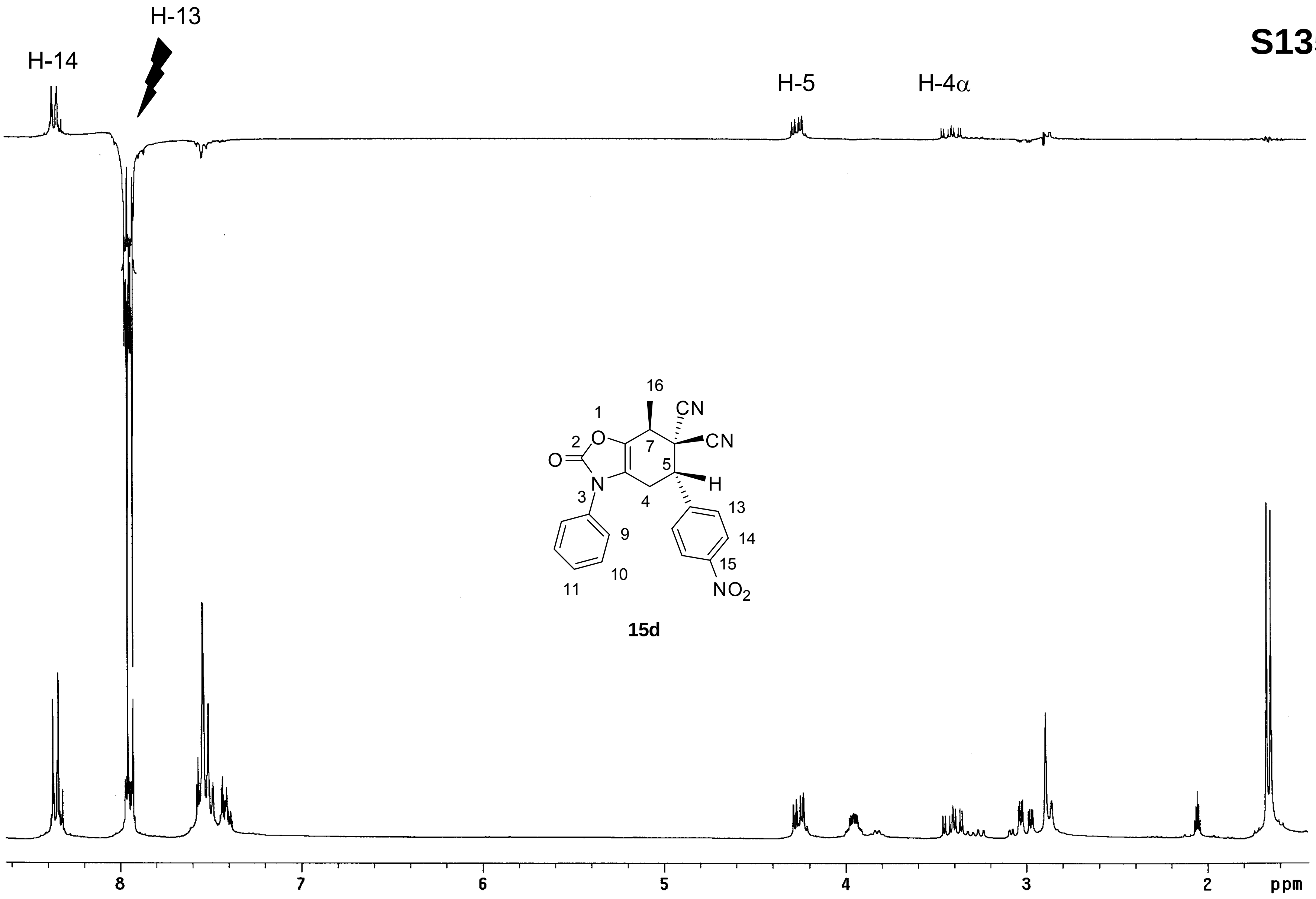

NOE experiment ($CDCl_3$) **15d**

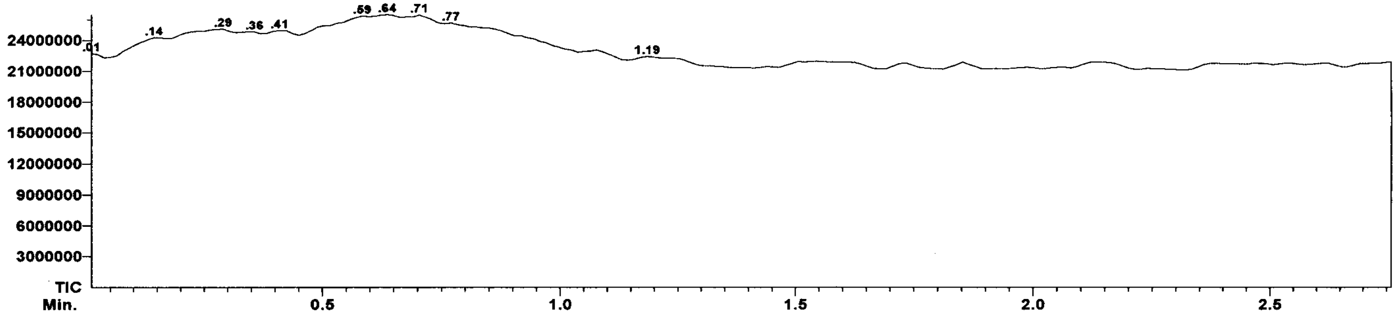

Scan: 39-66 **R.T.: .7**

Base: m/z 400; 15.3%FS TIC: 1248162 **#Ions: 2827**

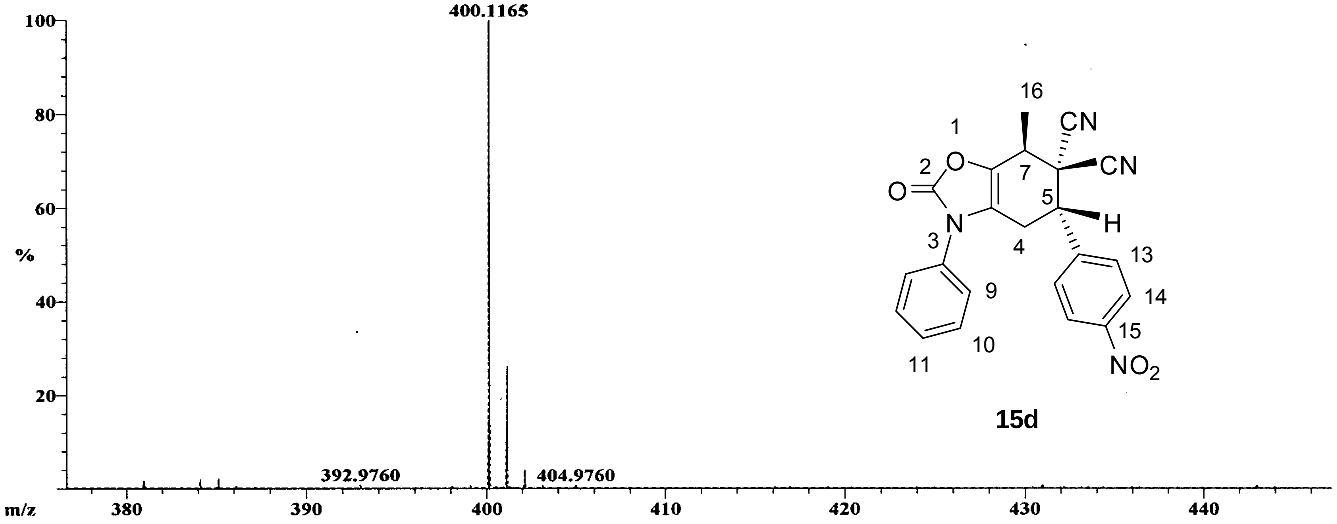

15d

Selected Isotopes : $H_{0-20}C_{0-25}N_{0-5}O_{0-5}$ **Error Limit : 1 mmu**

Measured Mass	*% Base*	*Formula*	*Calculated Mass*	*Error*
400.11651	100.0%	$C_{22}H_{16}N_4O_4$	400.11715	-0.6

HRMS **15d**

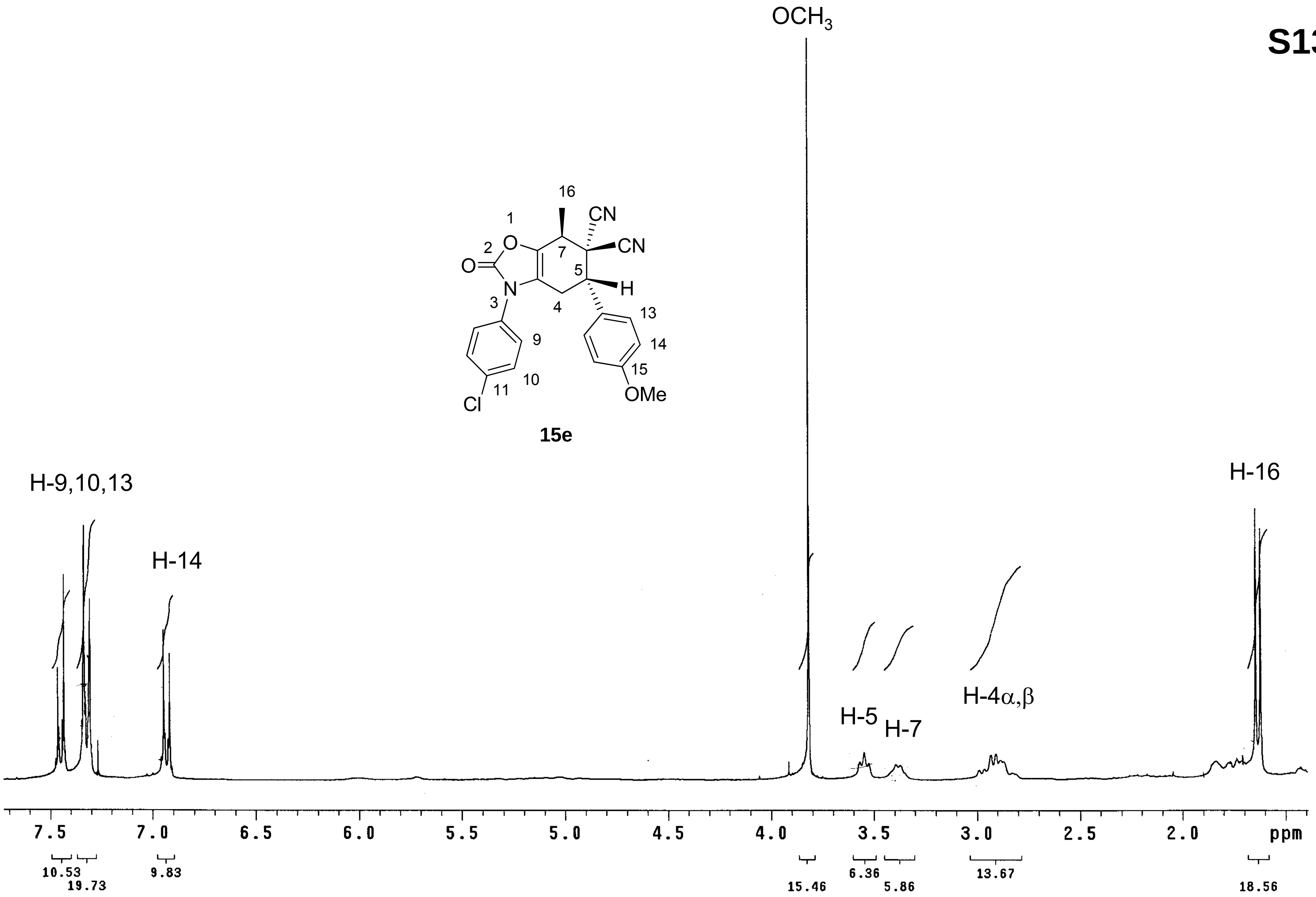

^{1}H NMR ($CDCl_3$) **15e**

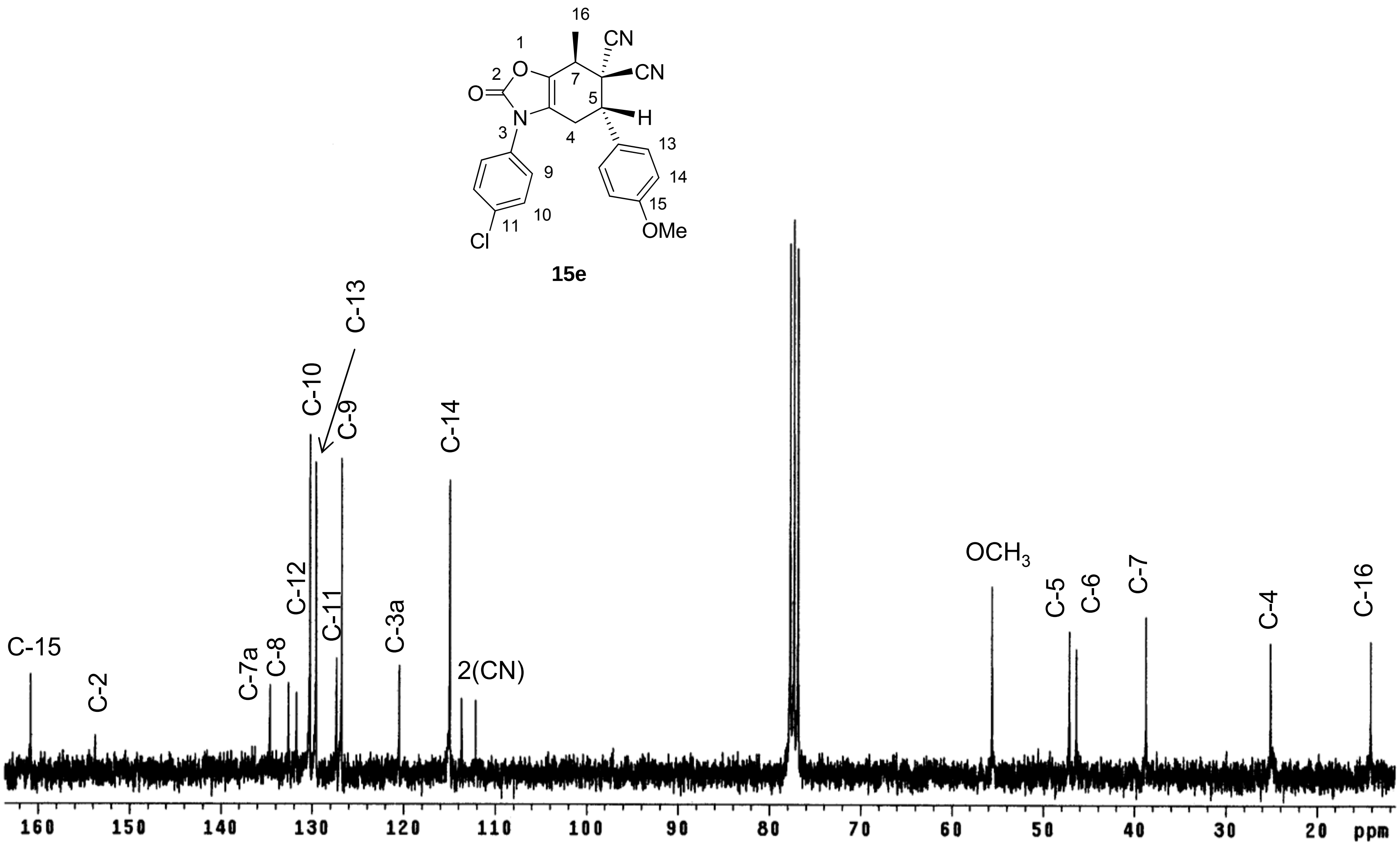

^{13}C NMR ($CDCl_3$) **15e**

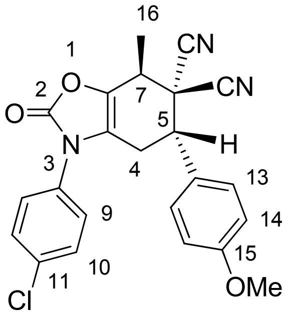

15e

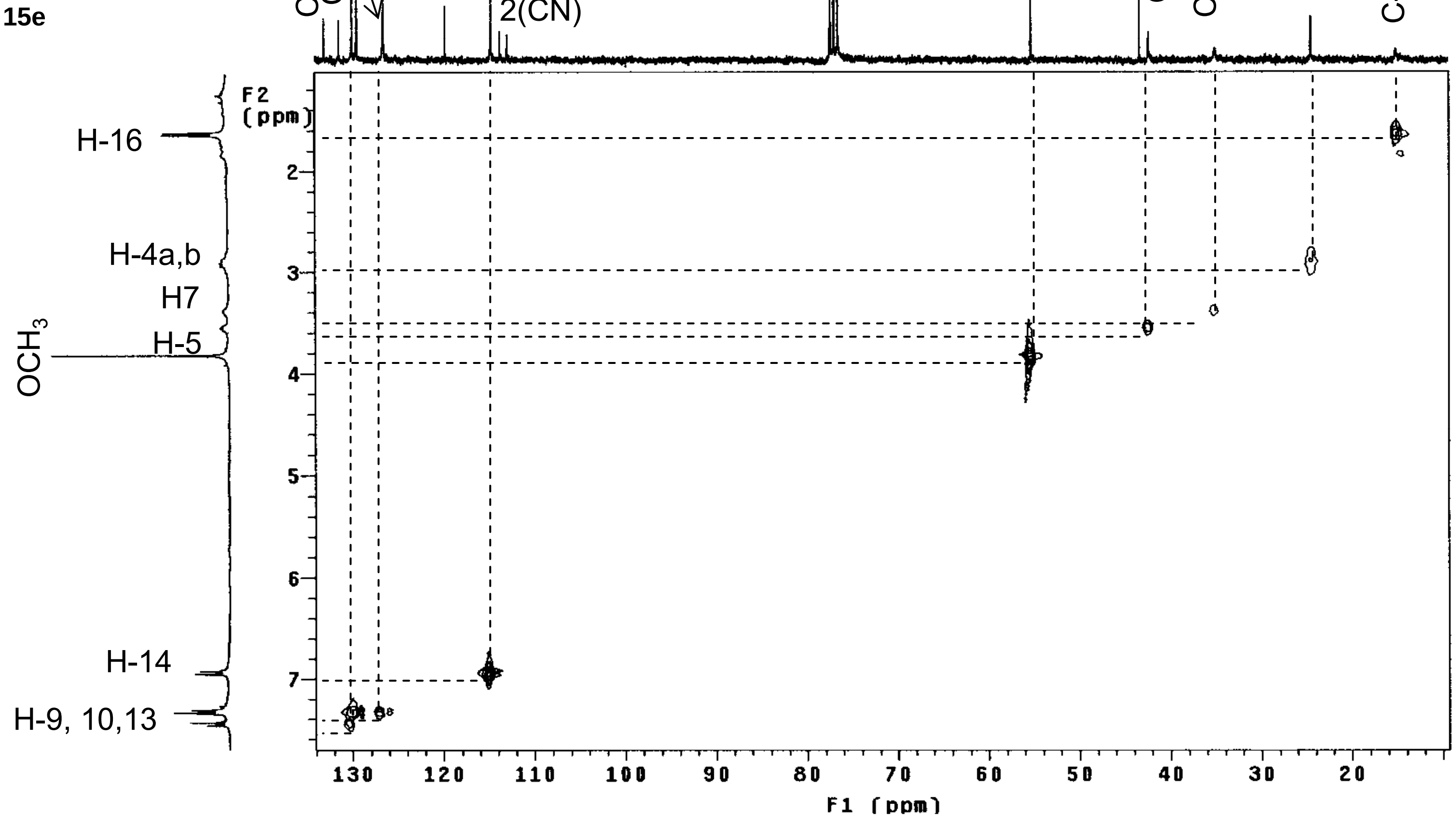

HSQC experiment ($CDCl_3$) **15e**

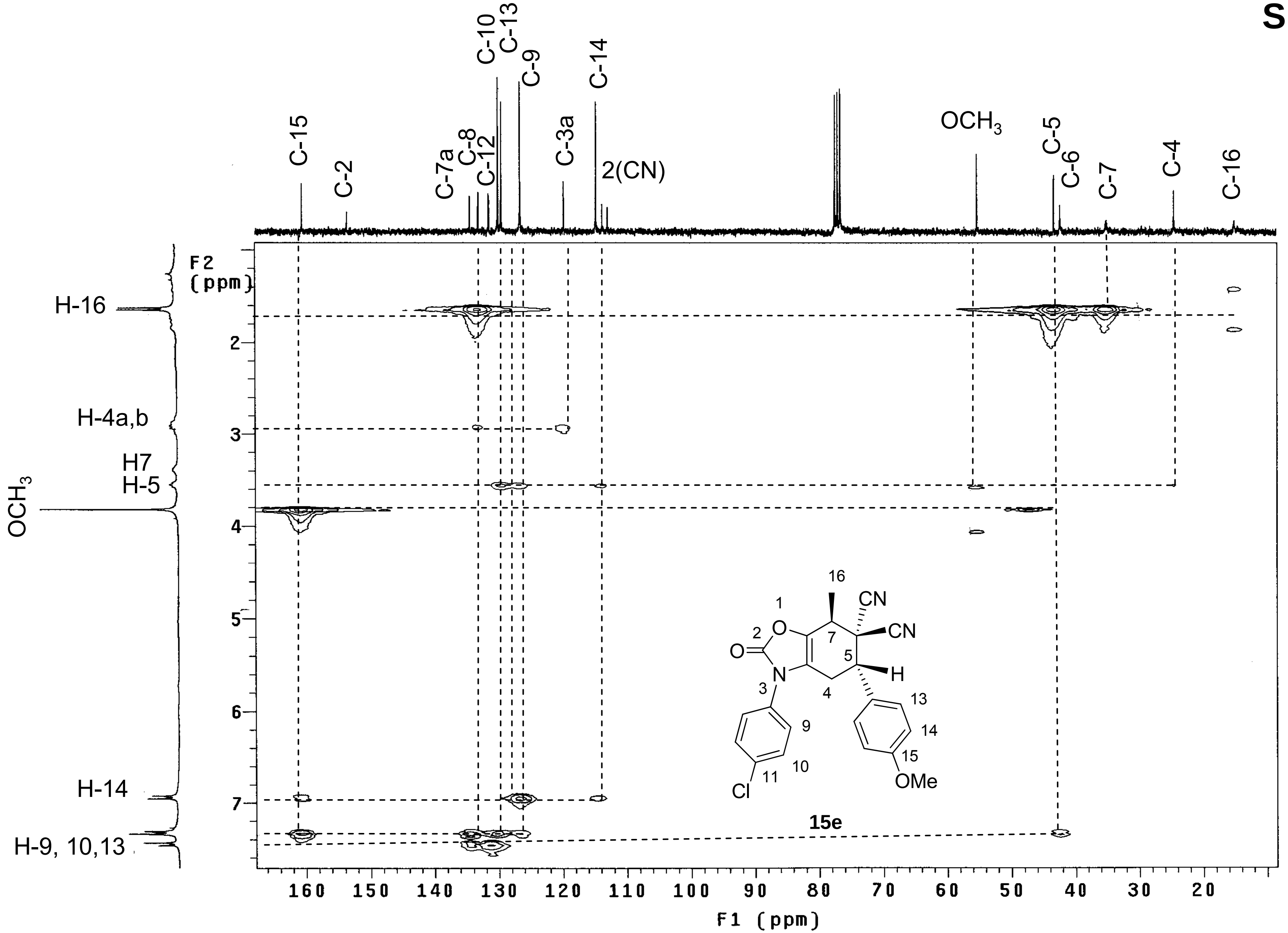

HMBC experiment ($CDCl_3$) **15e**

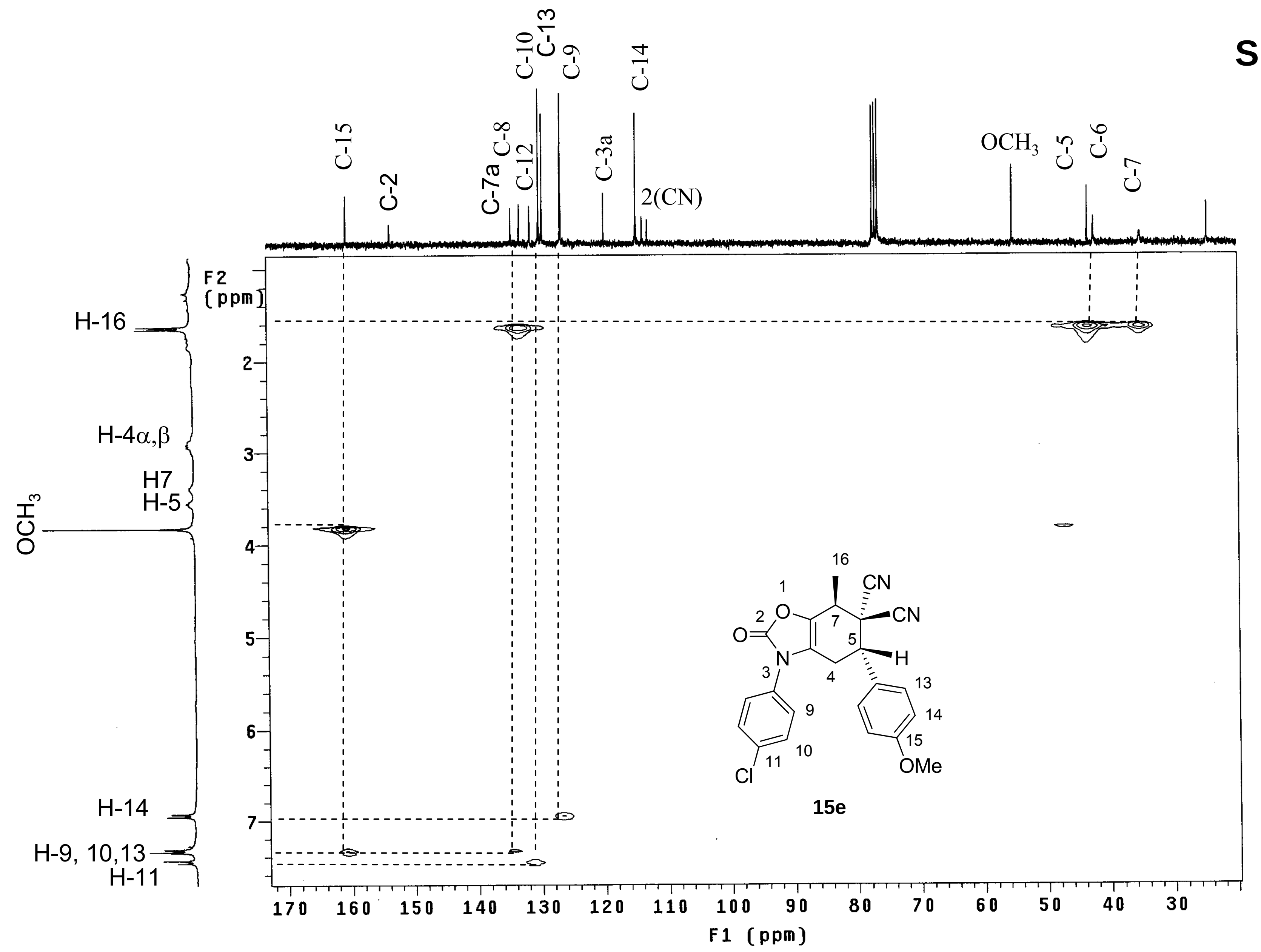

HMBC experiment ($CDCl_3$) **15e**

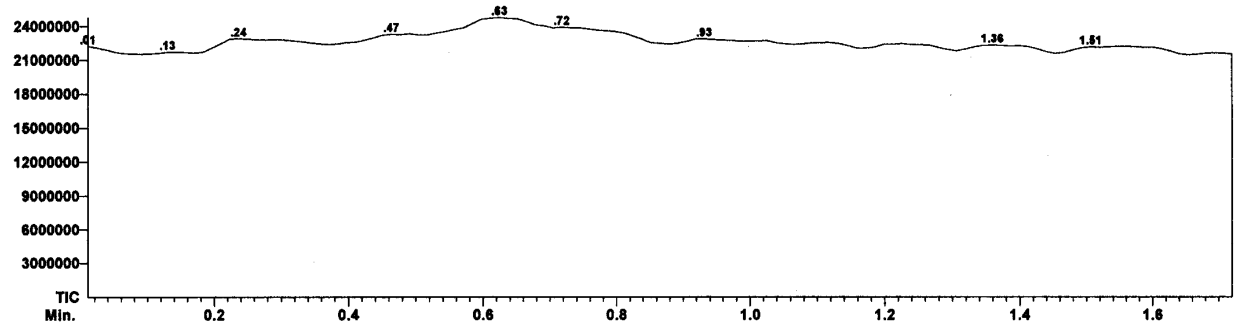

Scan: 41-55 **R.T.: .64**

Base: m/z 419; 5%FS TIC: 1172306 **#Ions: 2913**

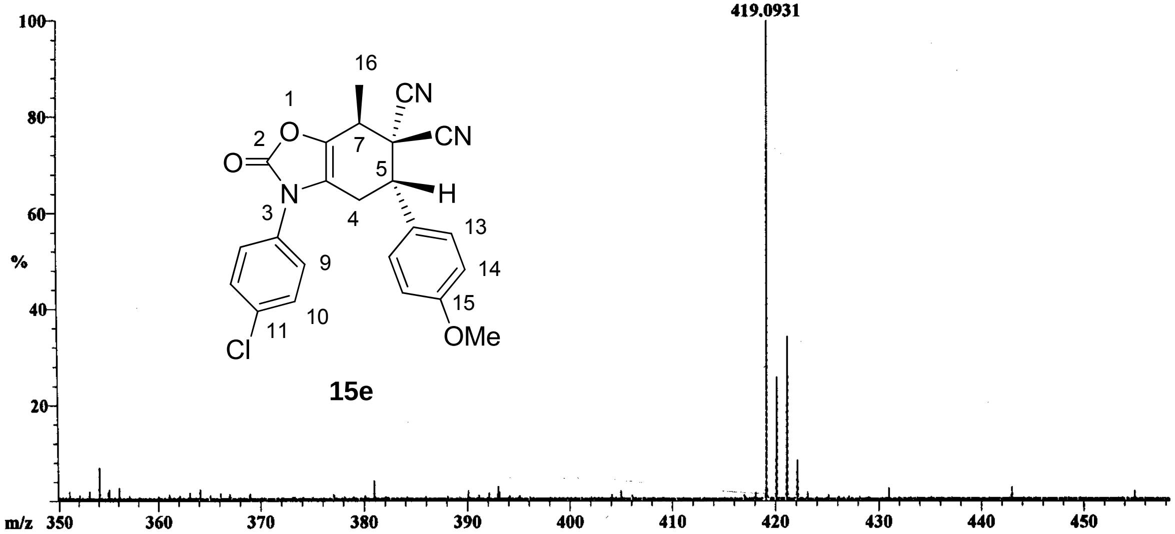

Selected Isotopes : $H_{0-20}C_{0-25}N_{0-4}O_{0-4}Cl_{0-2}$ **Error Limit : 1 mmu**

Measured Mass	***% Base***	***Formula***	***Calculated Mass***	***Error***
**419.09314**	**100.0%**	**$C_{24}H_{18}NO_4Cl$**	**419.09243**	**0.7**

HRMS **15e**

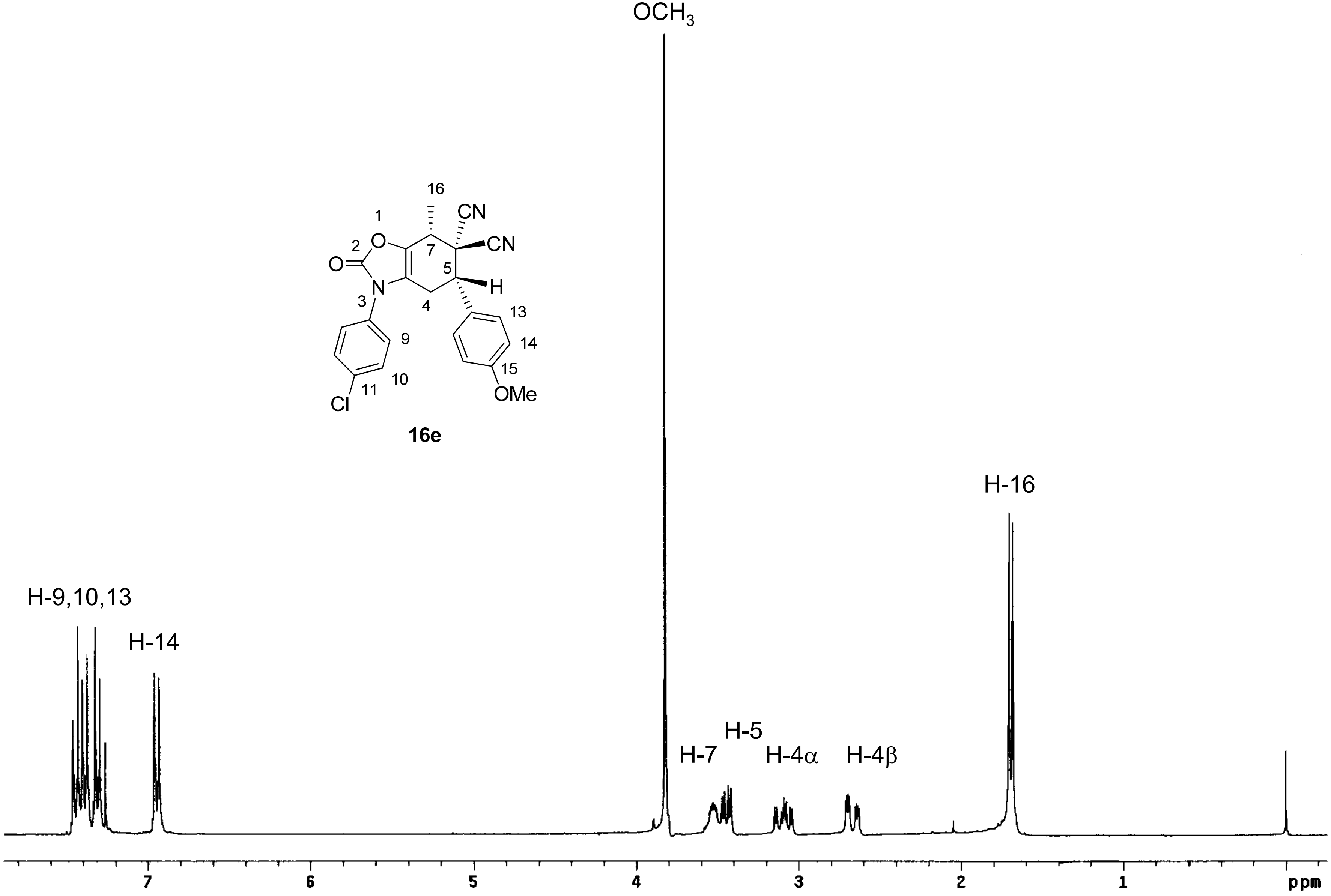

^{1}H NMR ($CDCl_3$) **16e**

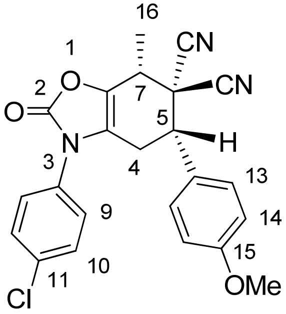

16e

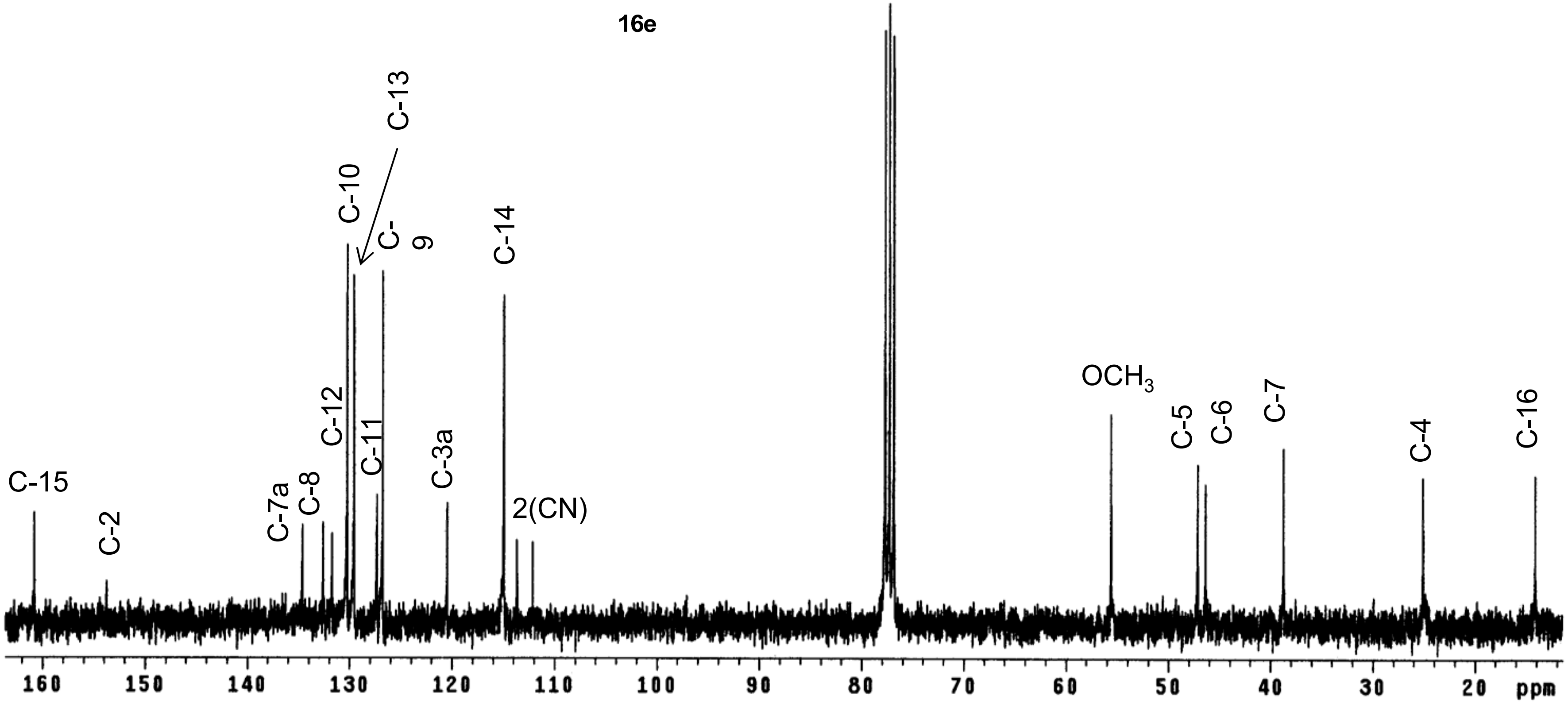

^{13}C NMR ($CDCl_3$) **16e**

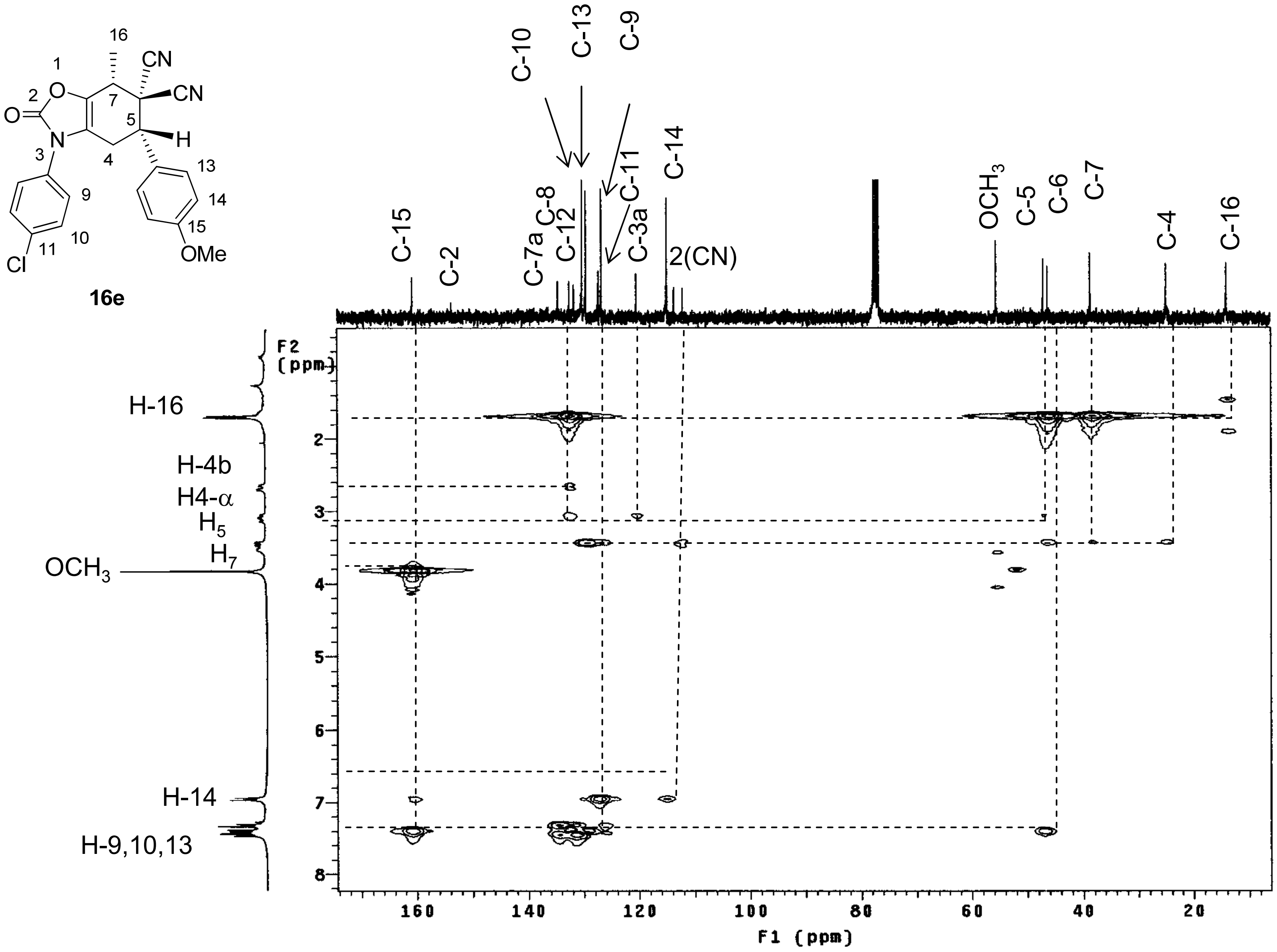

HMBC experiment (CDCl$_3$) **16e**

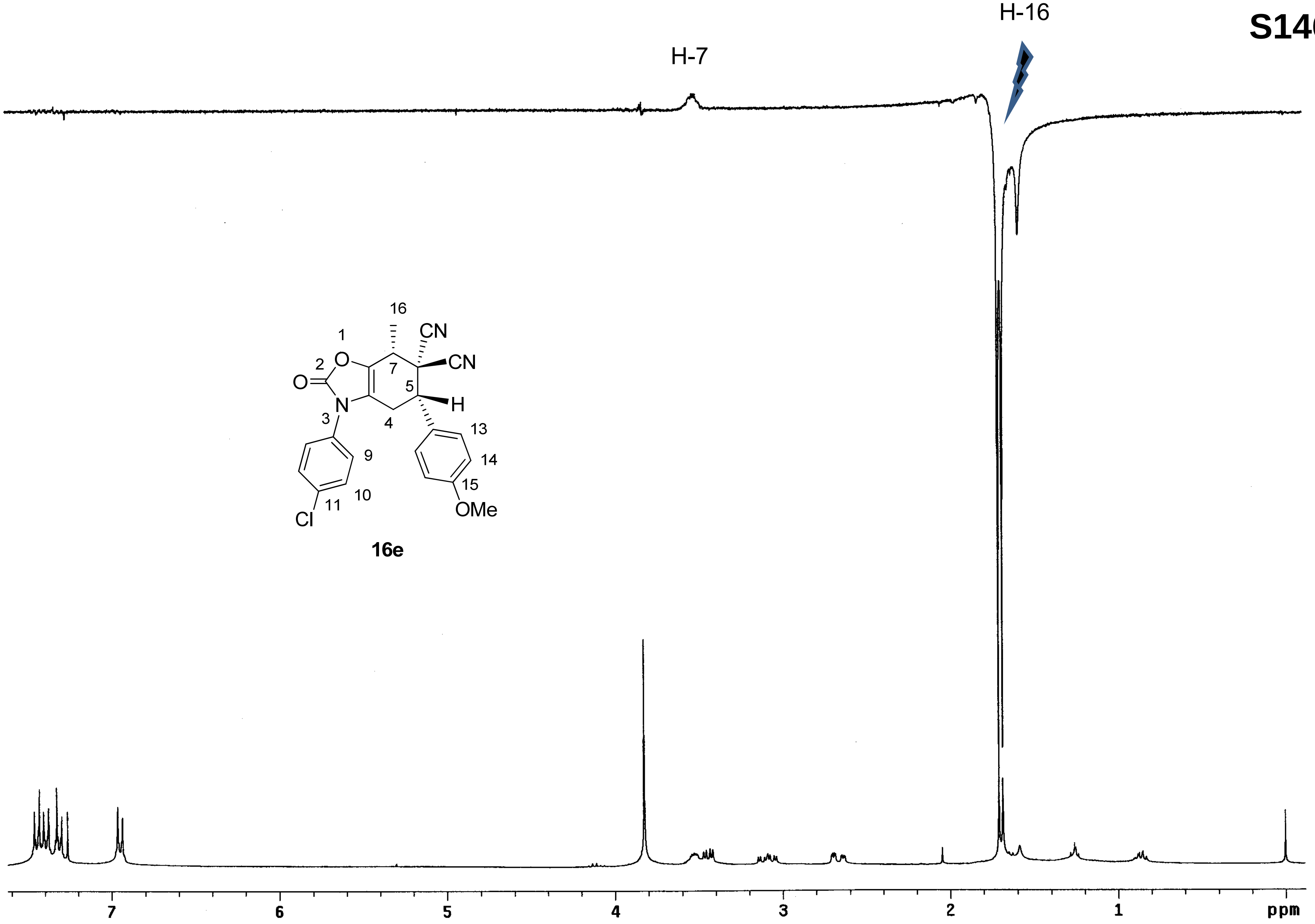

NOE experiment ($CDCl_3$) **16e**

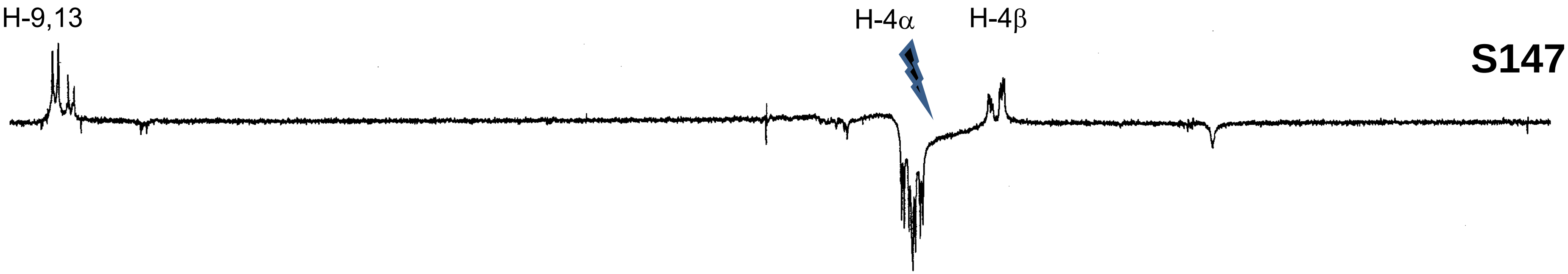

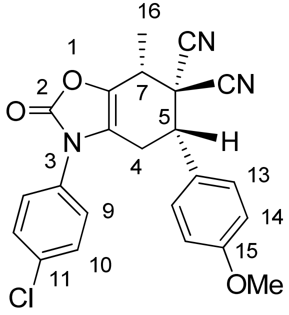

16e

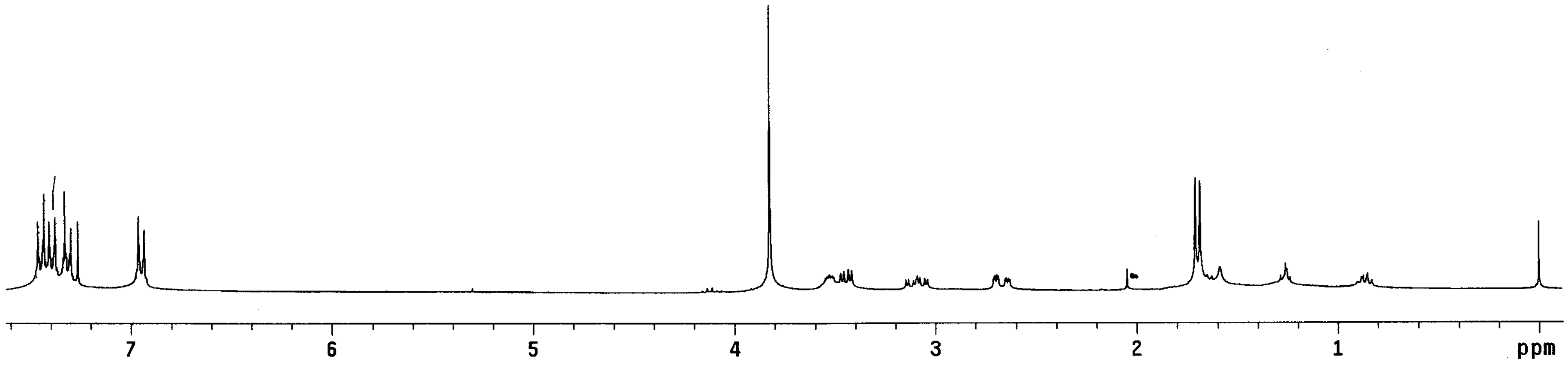

NOE experiment ($CDCl_3$) **16e**

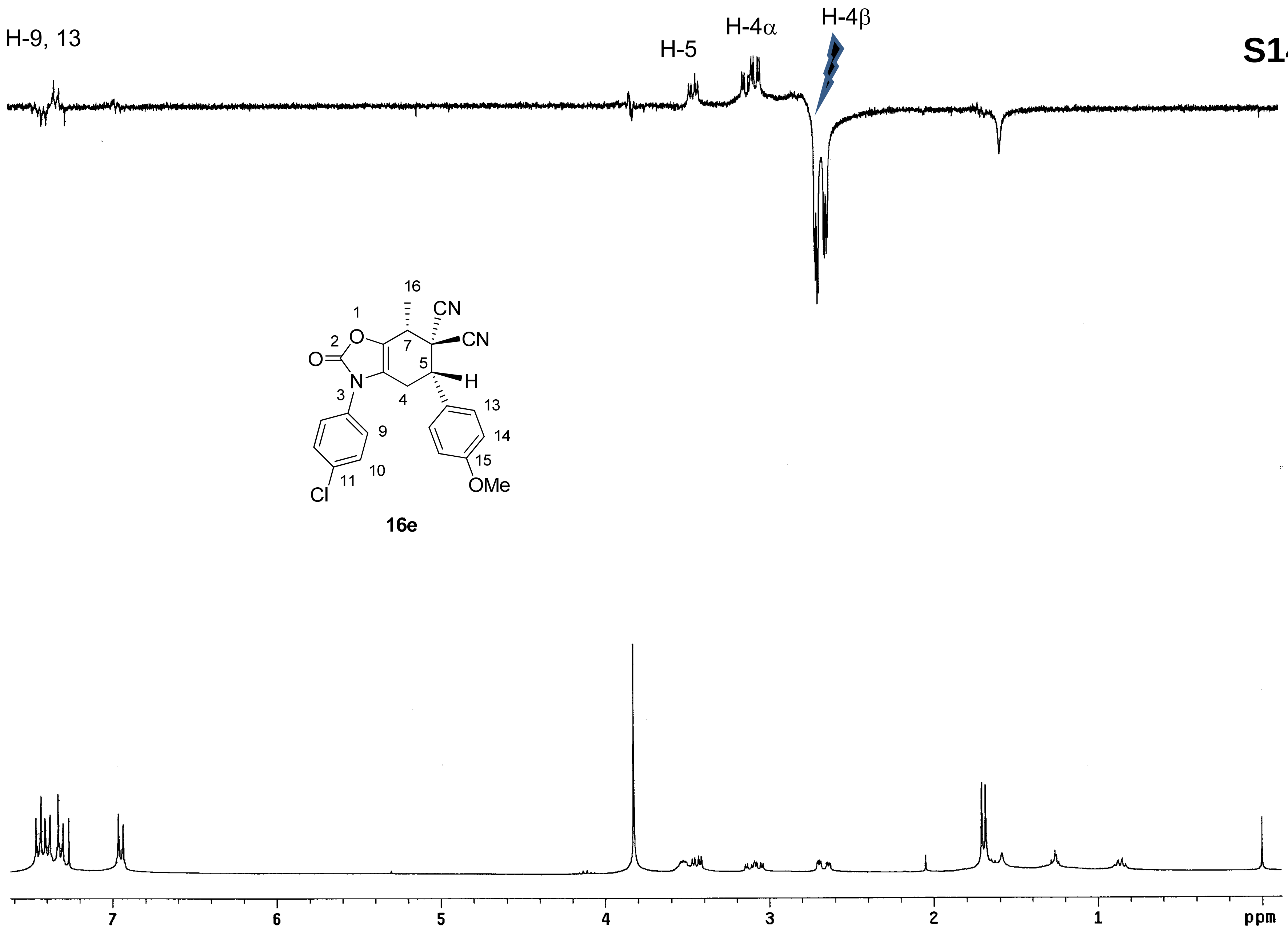

NOE experiment ($CDCl_3$) **16e**

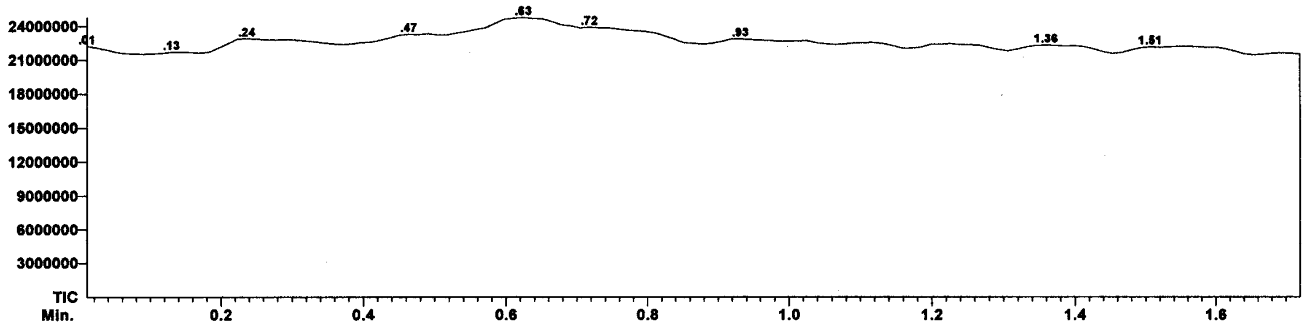

Scan: 41-55 **R.T.: .64**

Base: m/z 419; 5%FS TIC: 1172306 **#Ions: 2913**

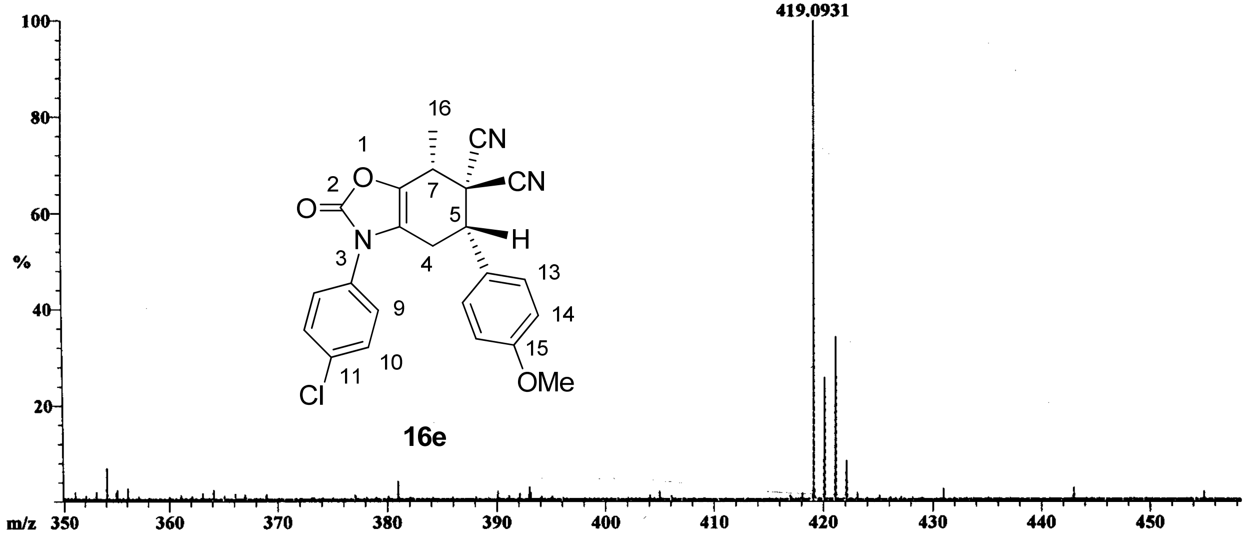

Selected Isotopes : $H_{0-20}C_{0-25}N_{0-4}O_{0-4}Cl_{0-2}$ **Error Limit : 1 mmu**

Measured Mass	***% Base***	***Formula***	***Calculated Mass***	***Error***
**419.09314**	**100.0%**	**$C_{24}H_{18}NO_4Cl$**	**419.09243**	**0.7**

HRMS **16e**

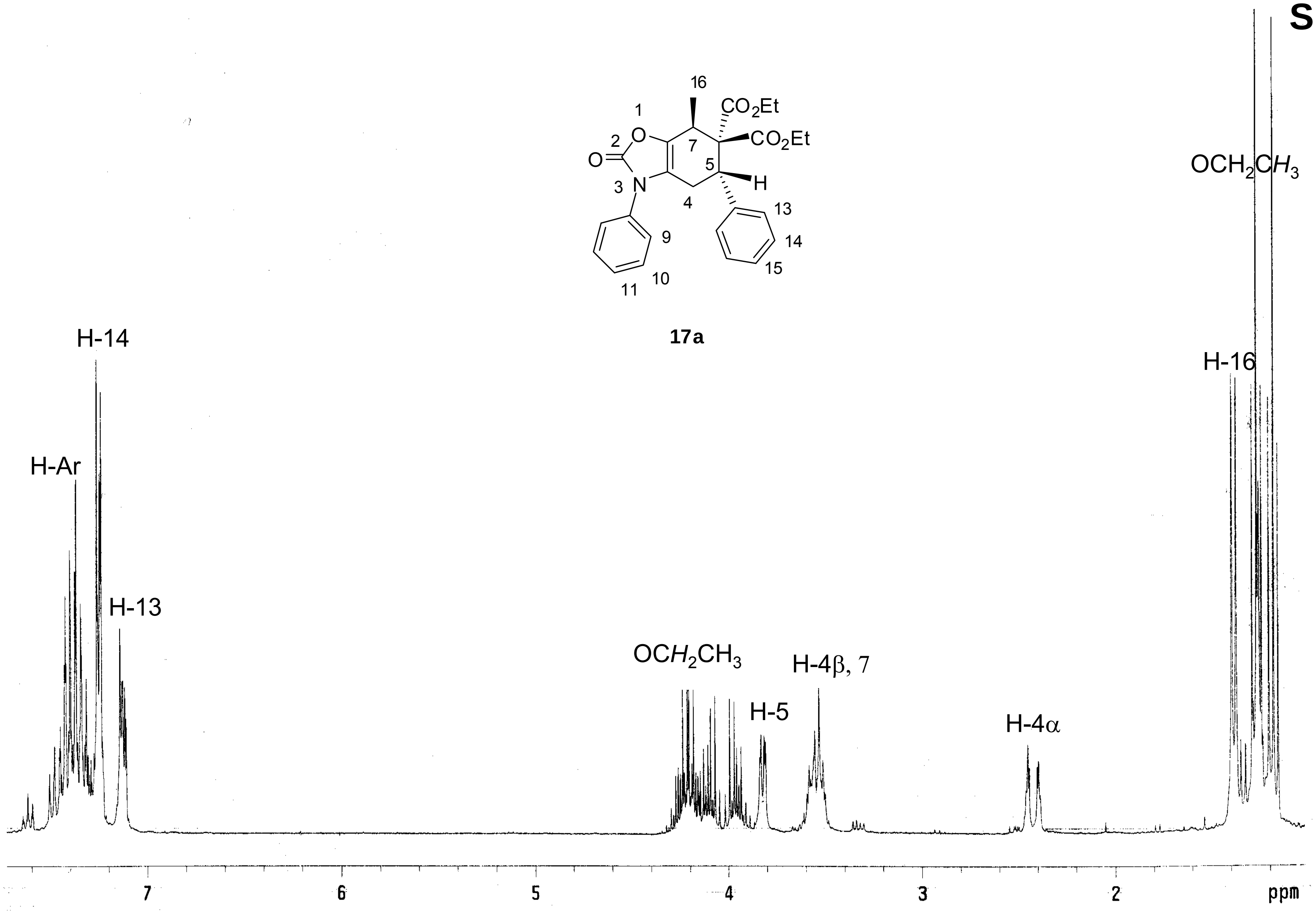

^{1}H NMR ($CDCl_3$) **17a**

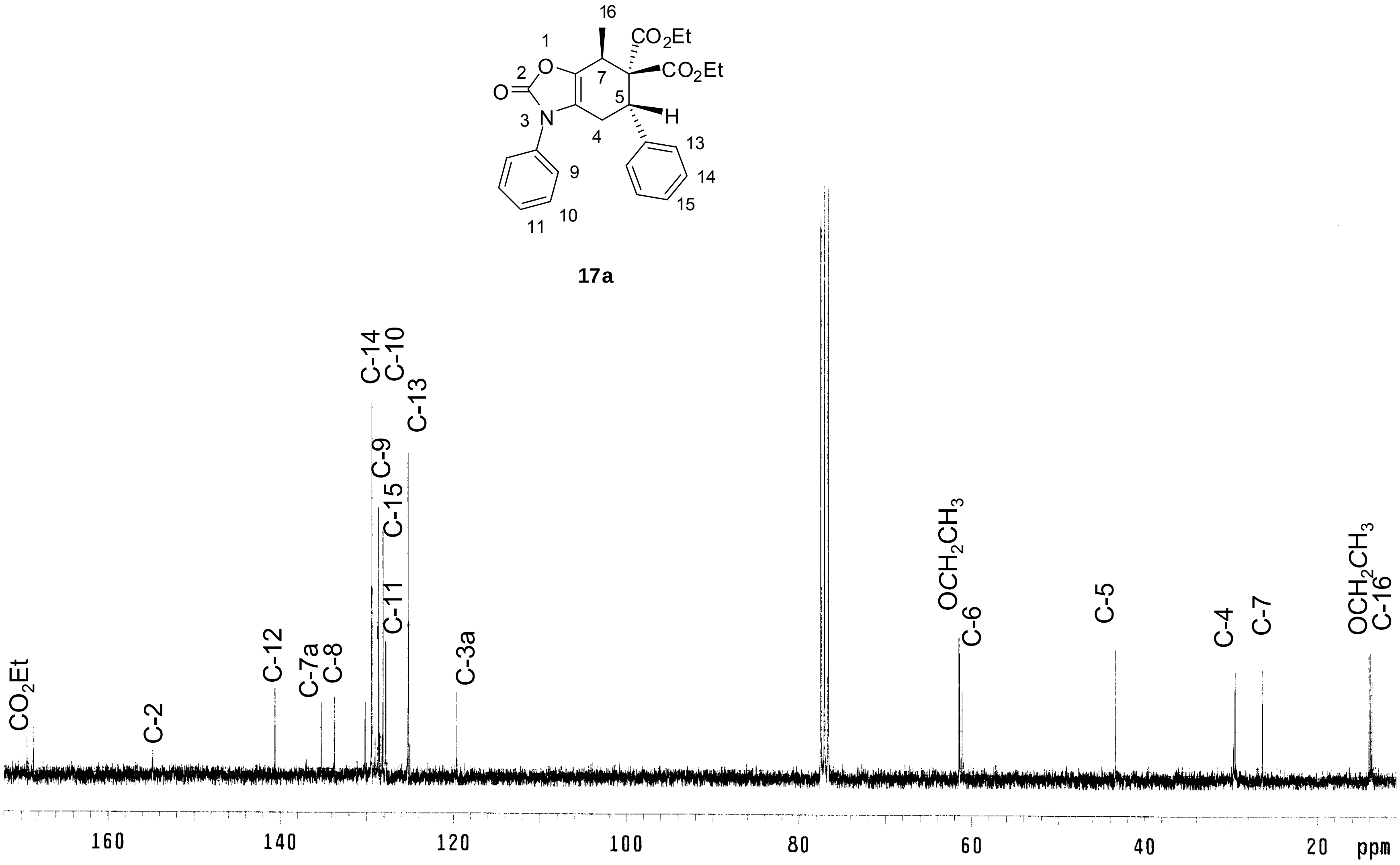

^{13}C NMR ($CDCl_3$) **17a**

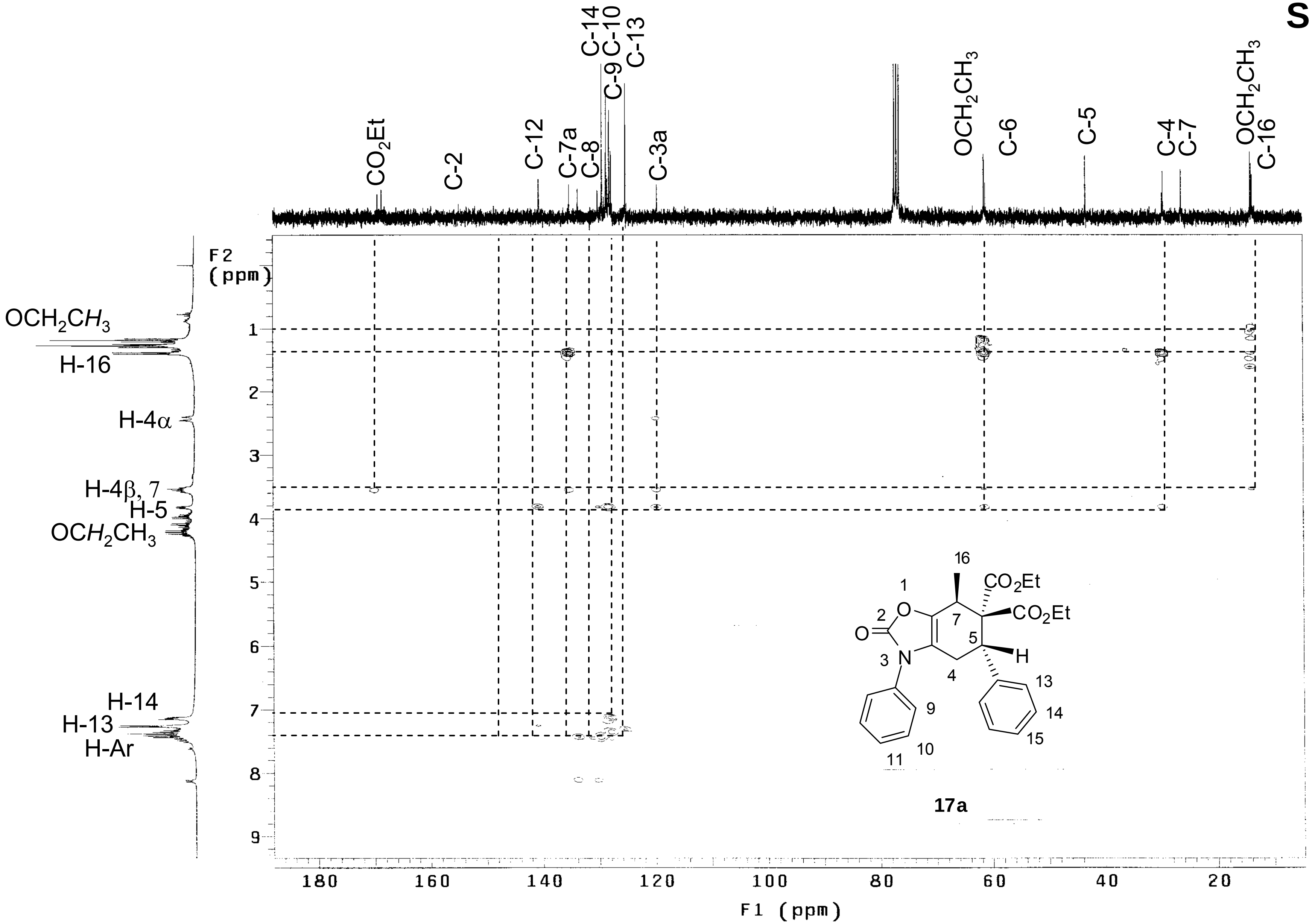

HMBC experiment ($CDCl_3$) **17a**

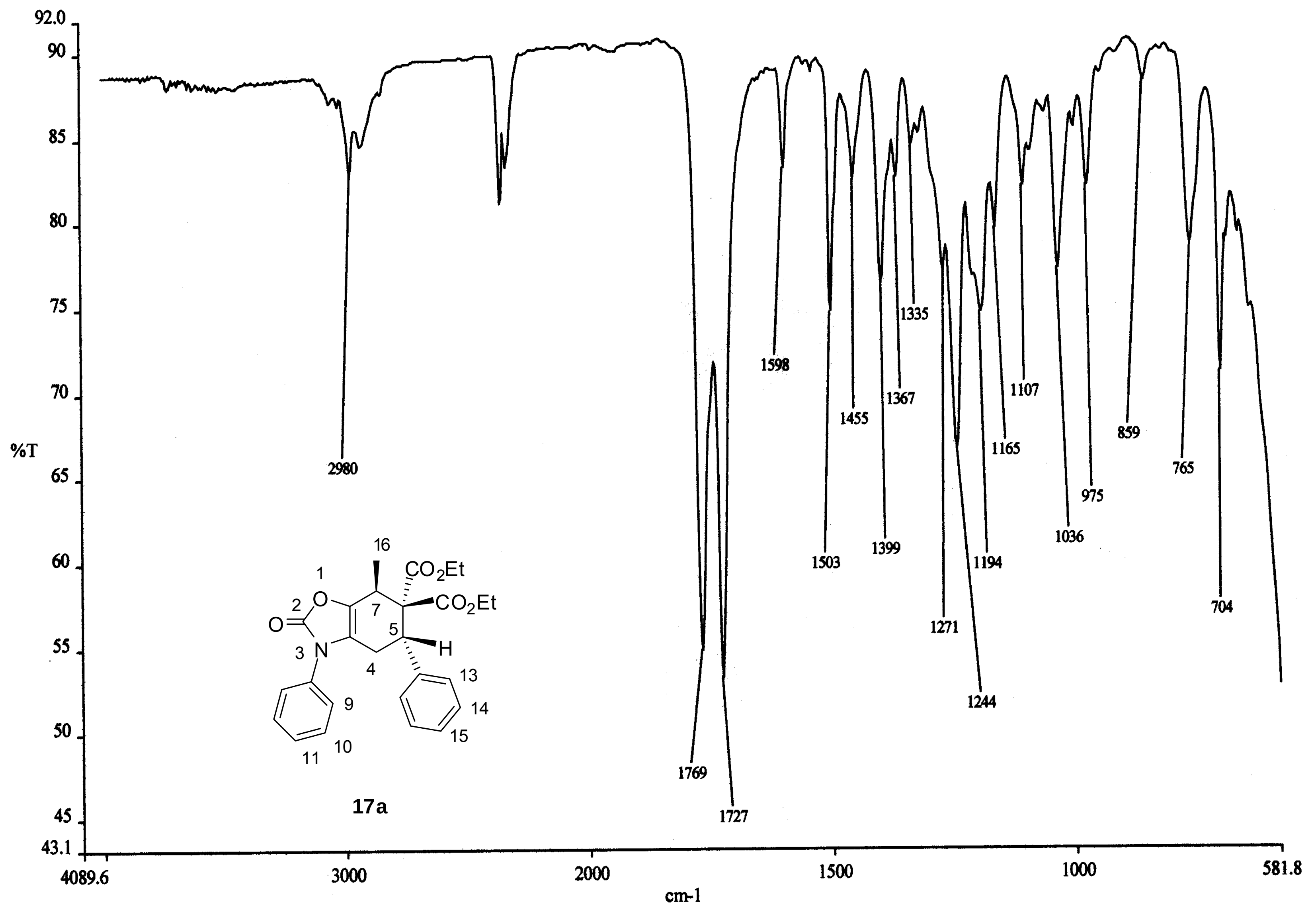
92.0
90
85
80
75
70
65
60
55
50
45
43.1
%T
4089.6
3000
2000
1500
1000
581.8
cm-1
2980
1769
1727
1598
1503
1455
1399
1367
1335
1271
1244
1194
1165
1107
1036
975
859
765
704
CO2Et
CO2Et
H
O
N
1 2 3 4 5 7 9 10 11 13 14 15 16
17a

FT-IR (KBr) **17a**

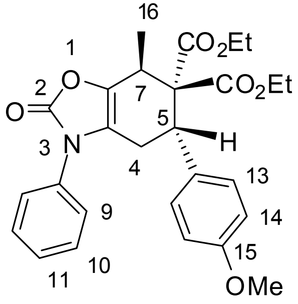

17b

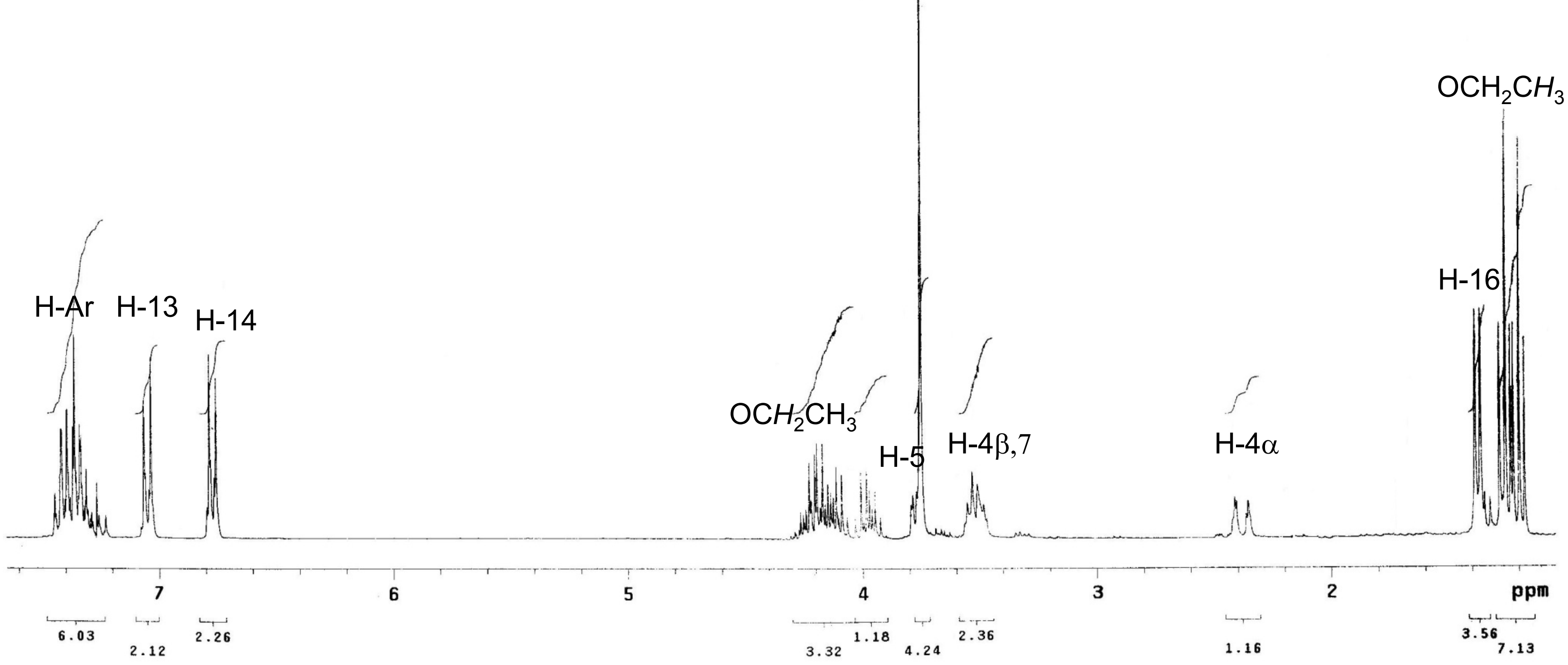

^{1}H NMR ($CDCl_3$) **17b**

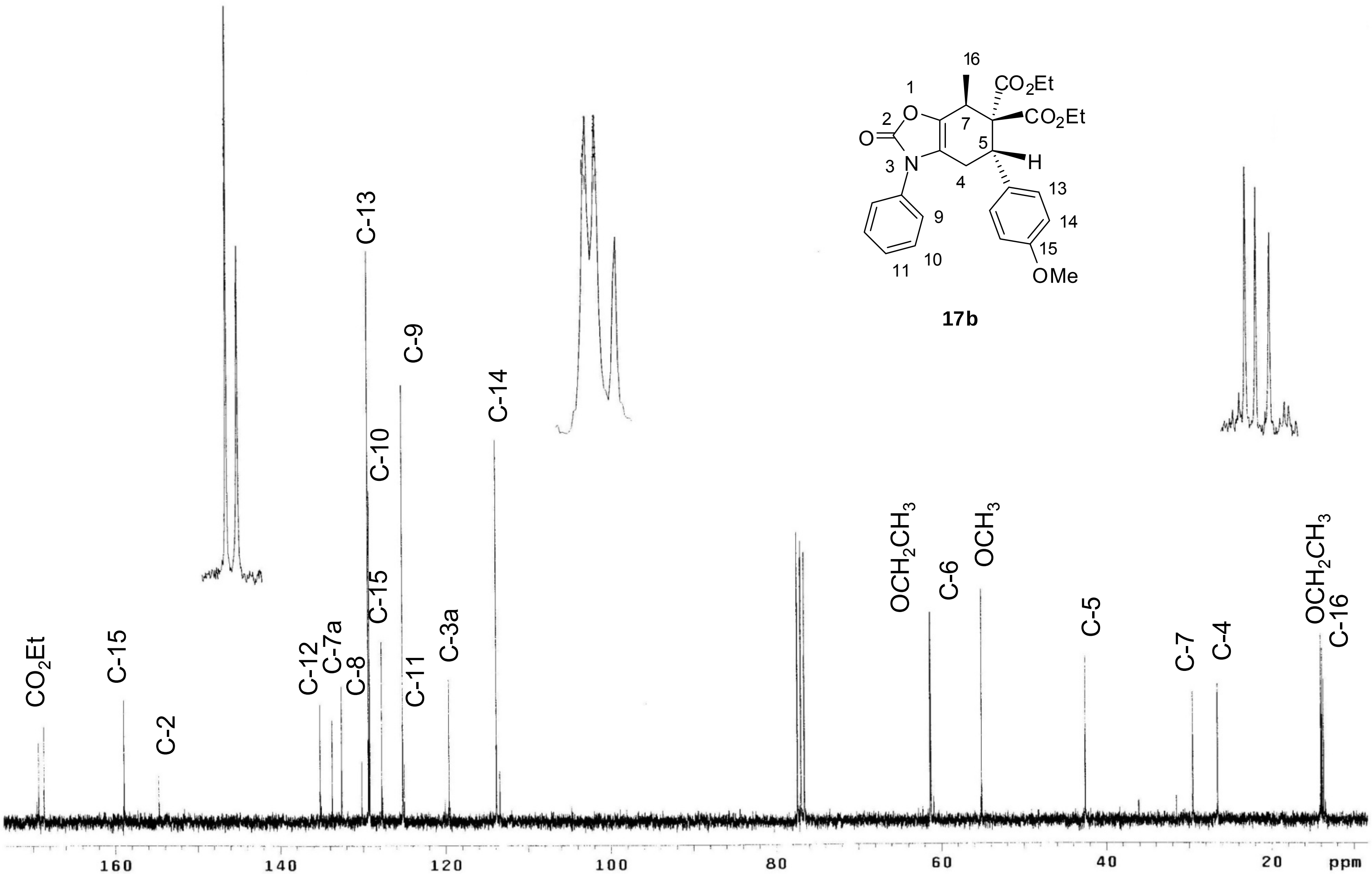

^{13}C NMR ($CDCl_3$) **17b**

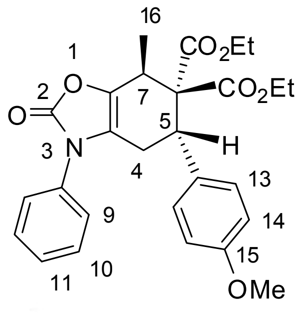

17b

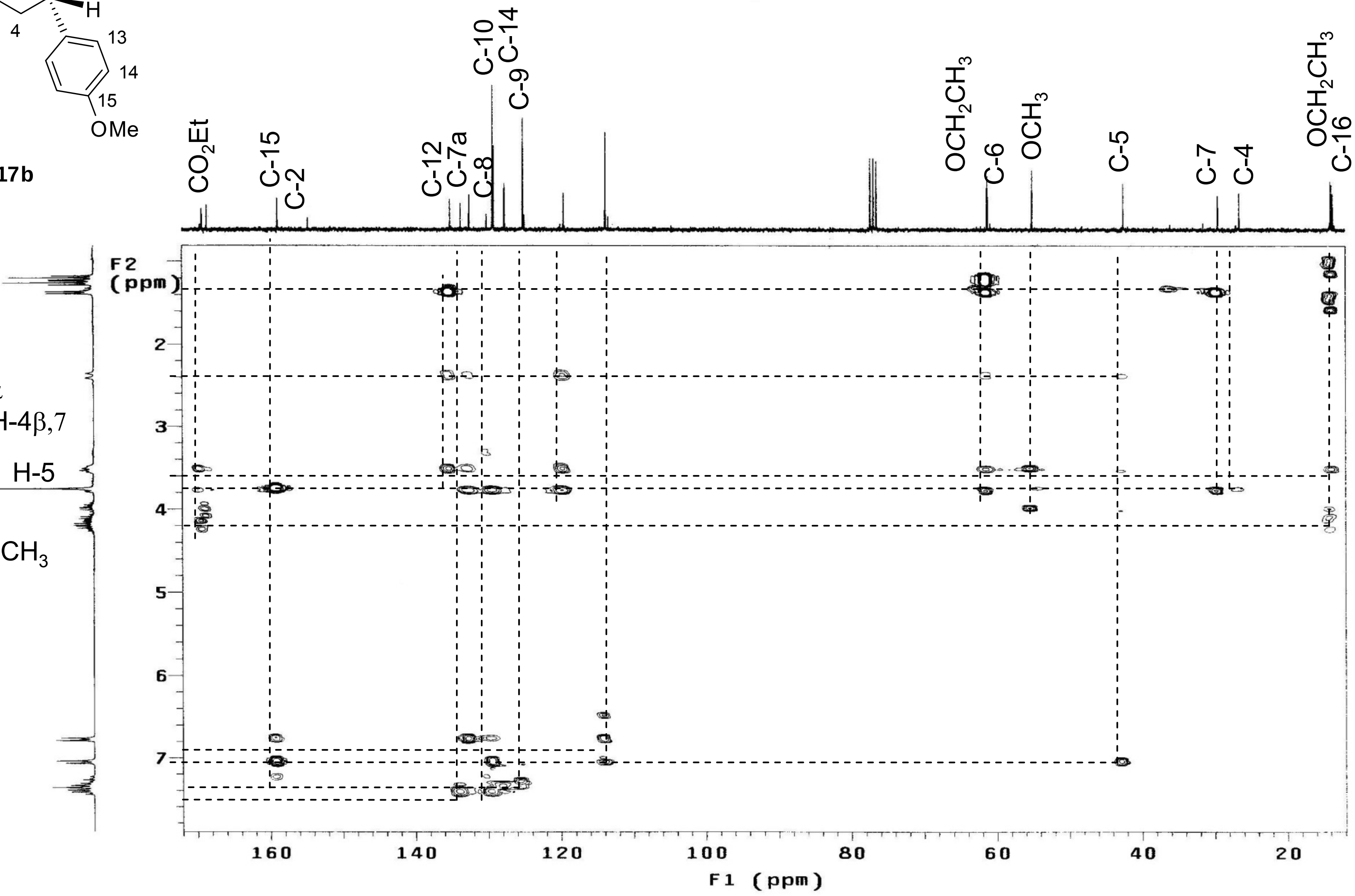

HMBC experiment ($CDCl_3$) **17b**

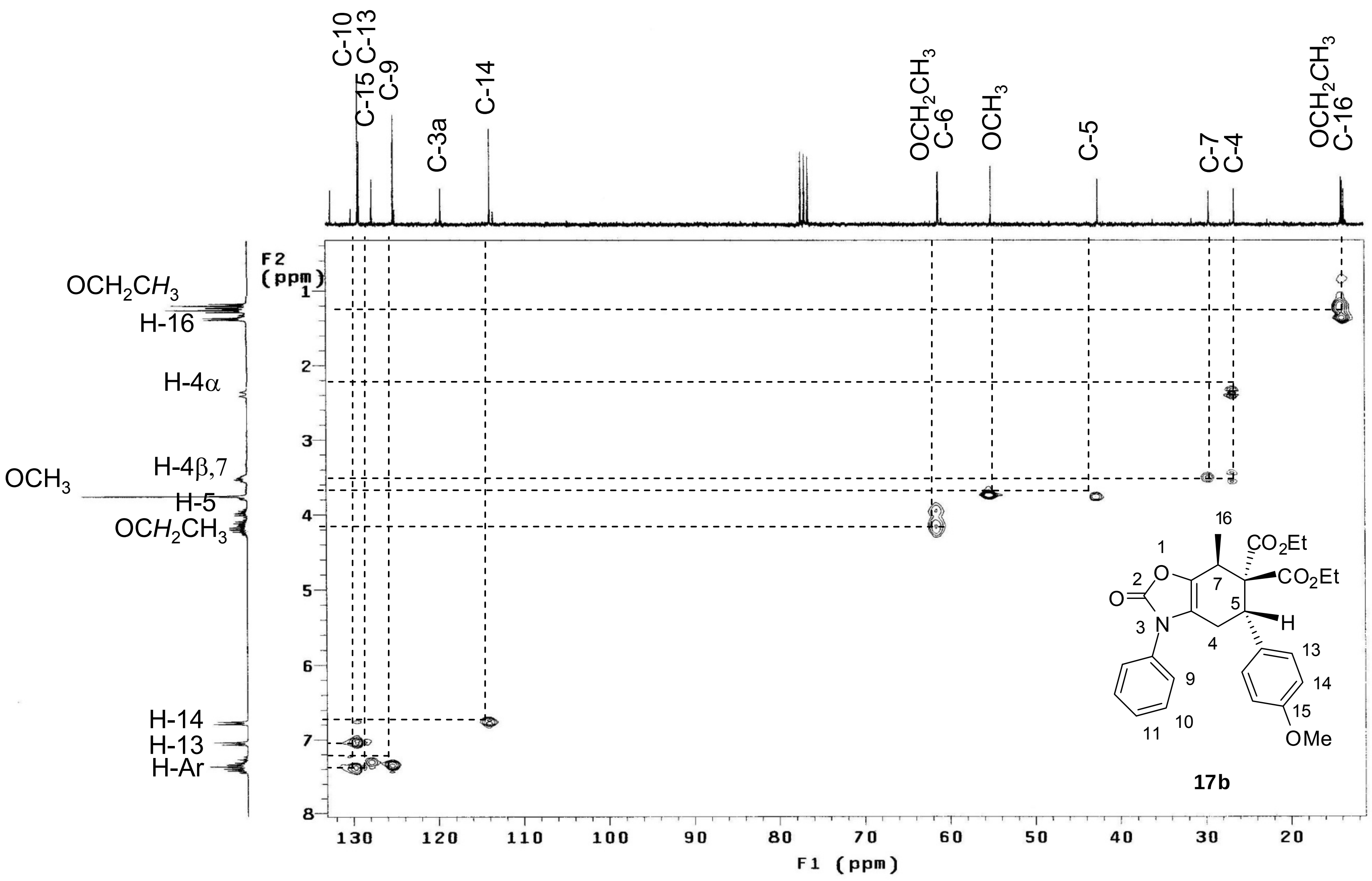

HSQC experiment ($CDCl_3$) **17b**

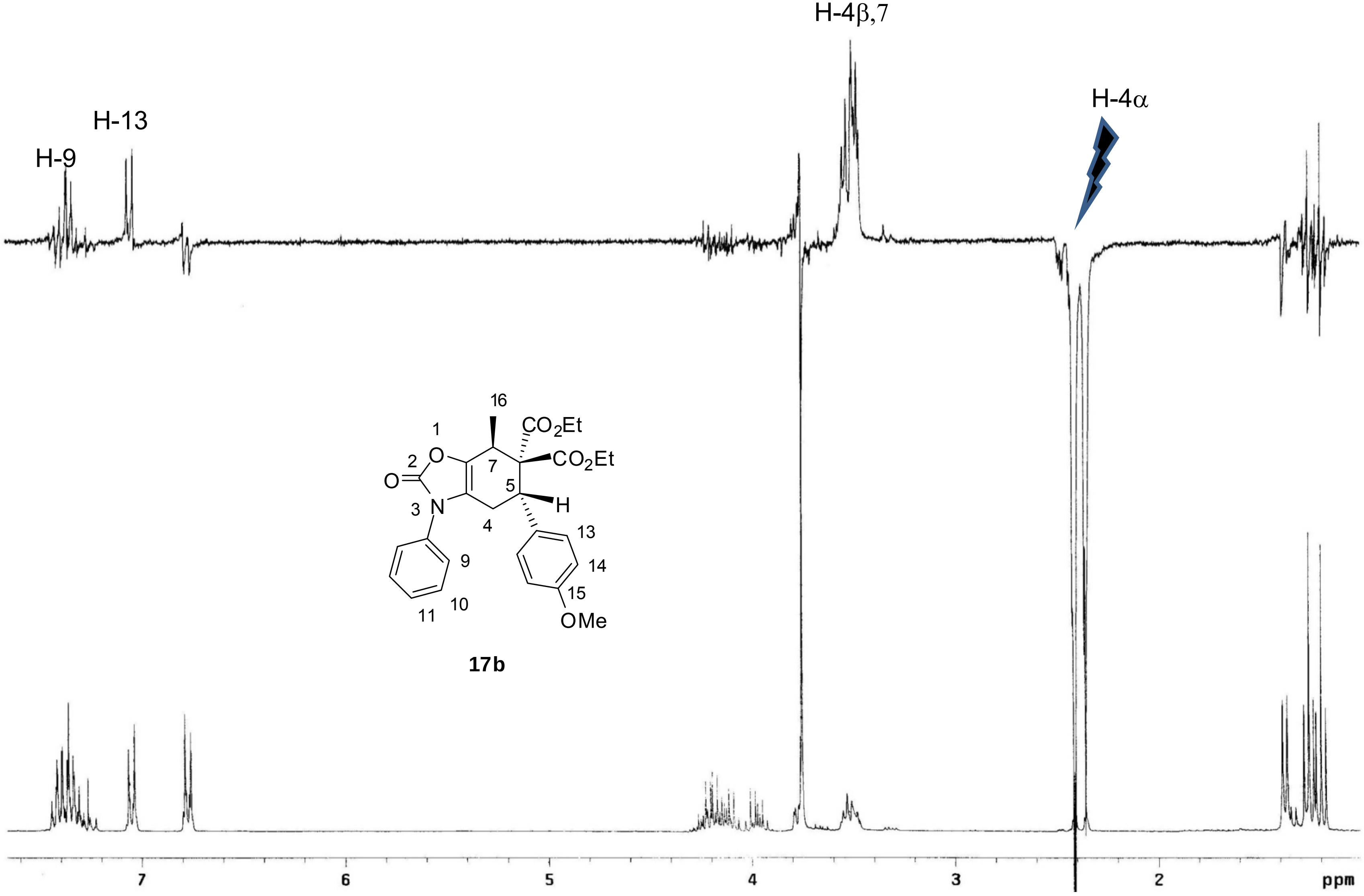

NOE experiment ($CDCl_3$) **17b**

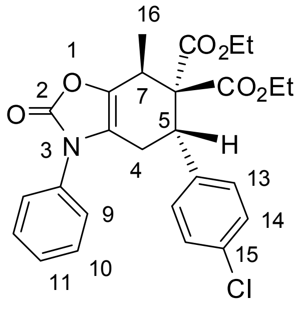

17c

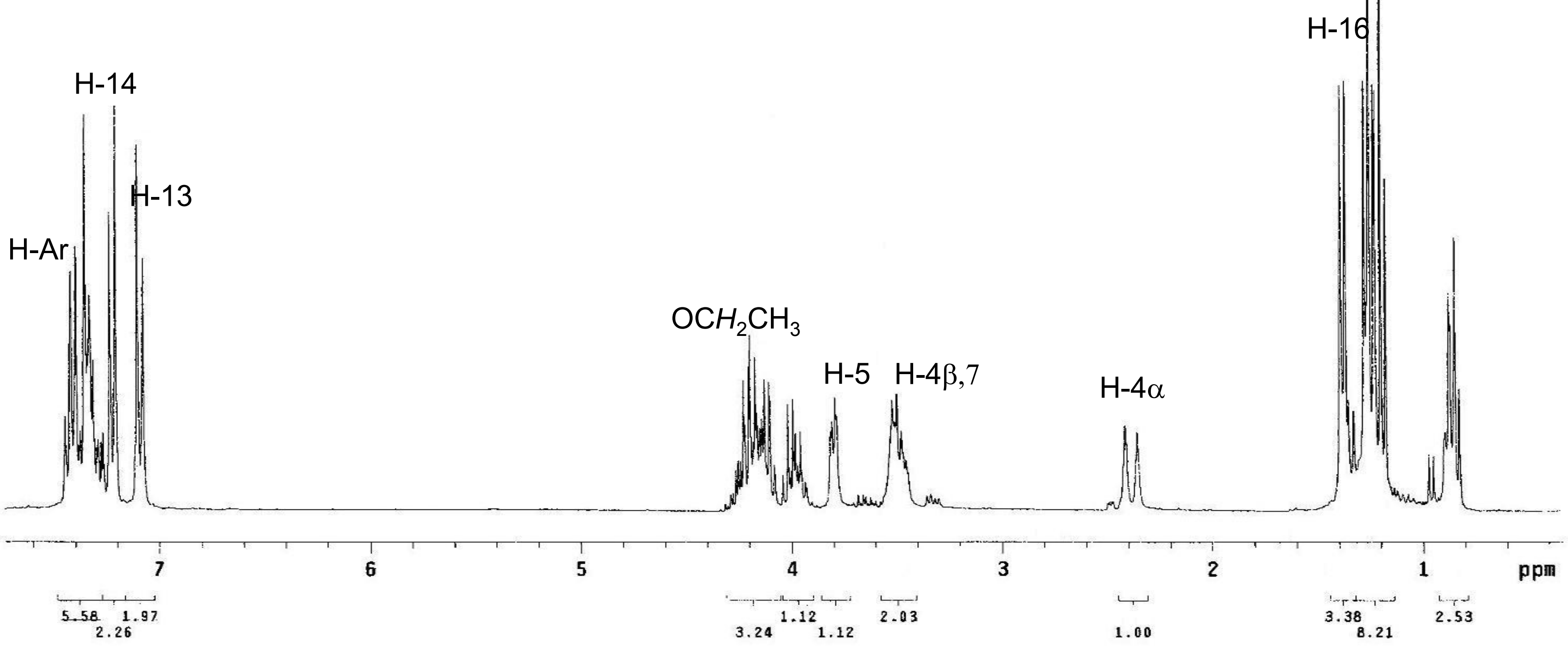

^{1}H NMR ($CDCl_3$) **17c**

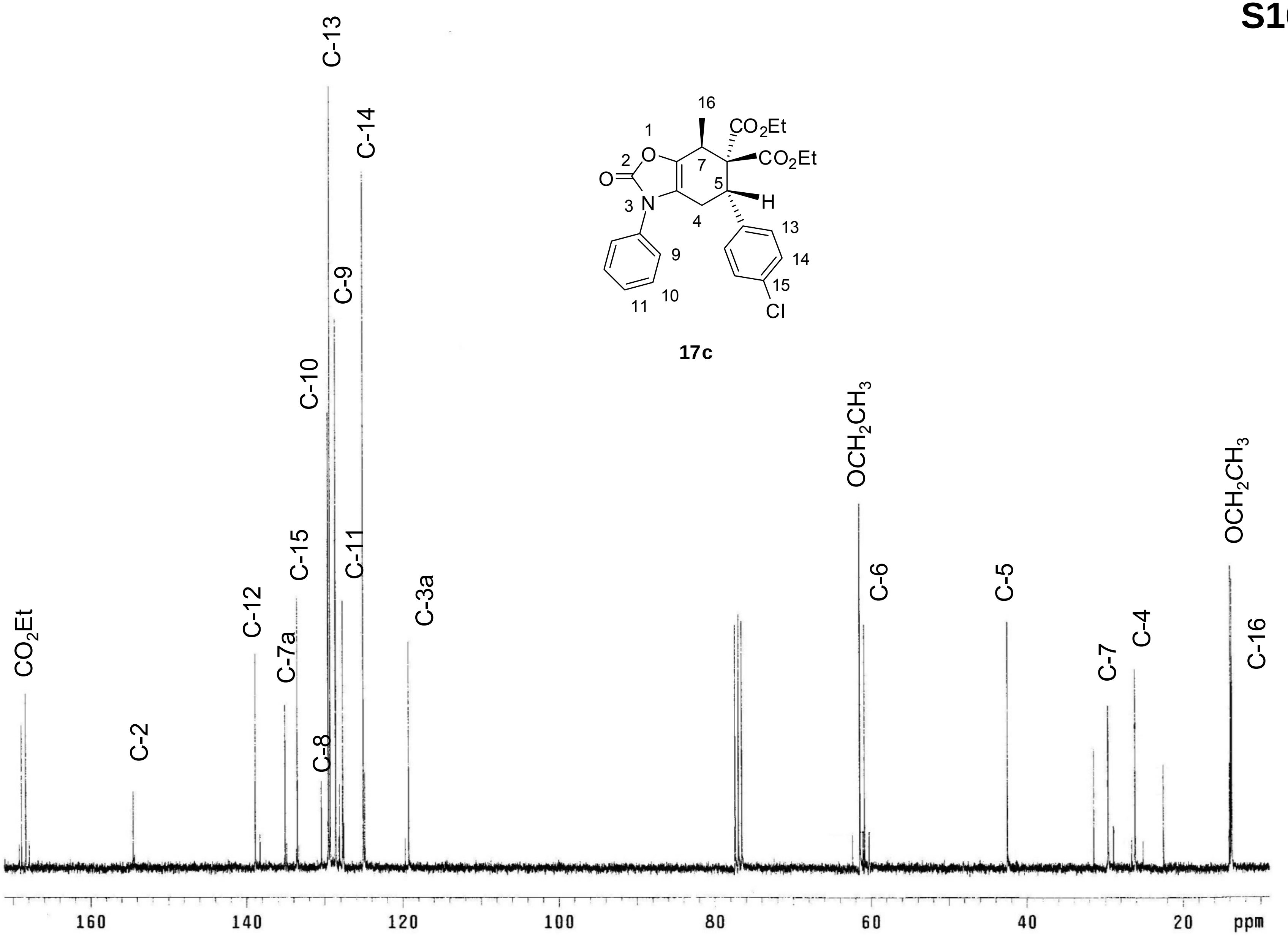

^{13}C NMR ($CDCl_3$) **17c**

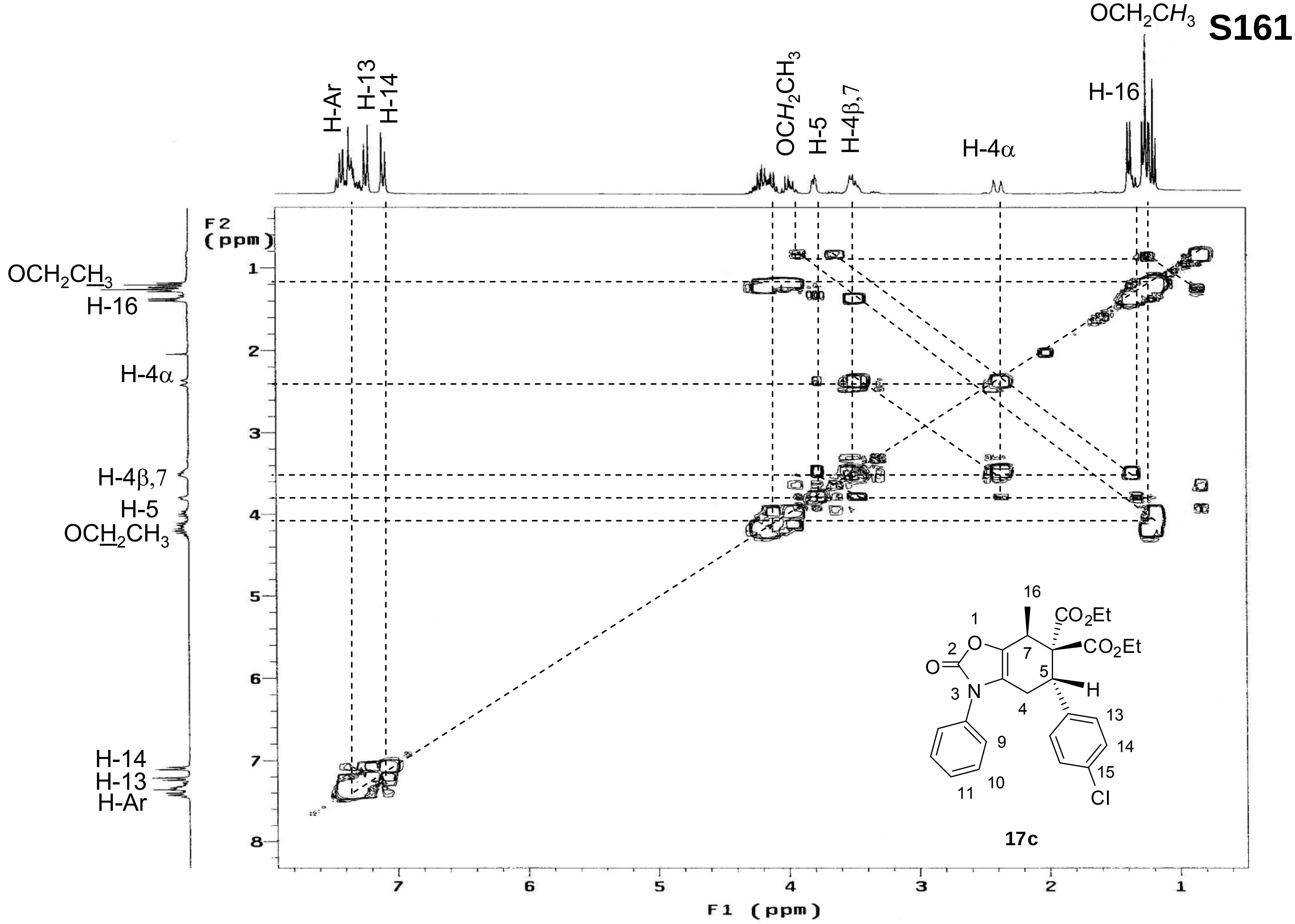

COSY experiment ($CDCl_3$) **17c**

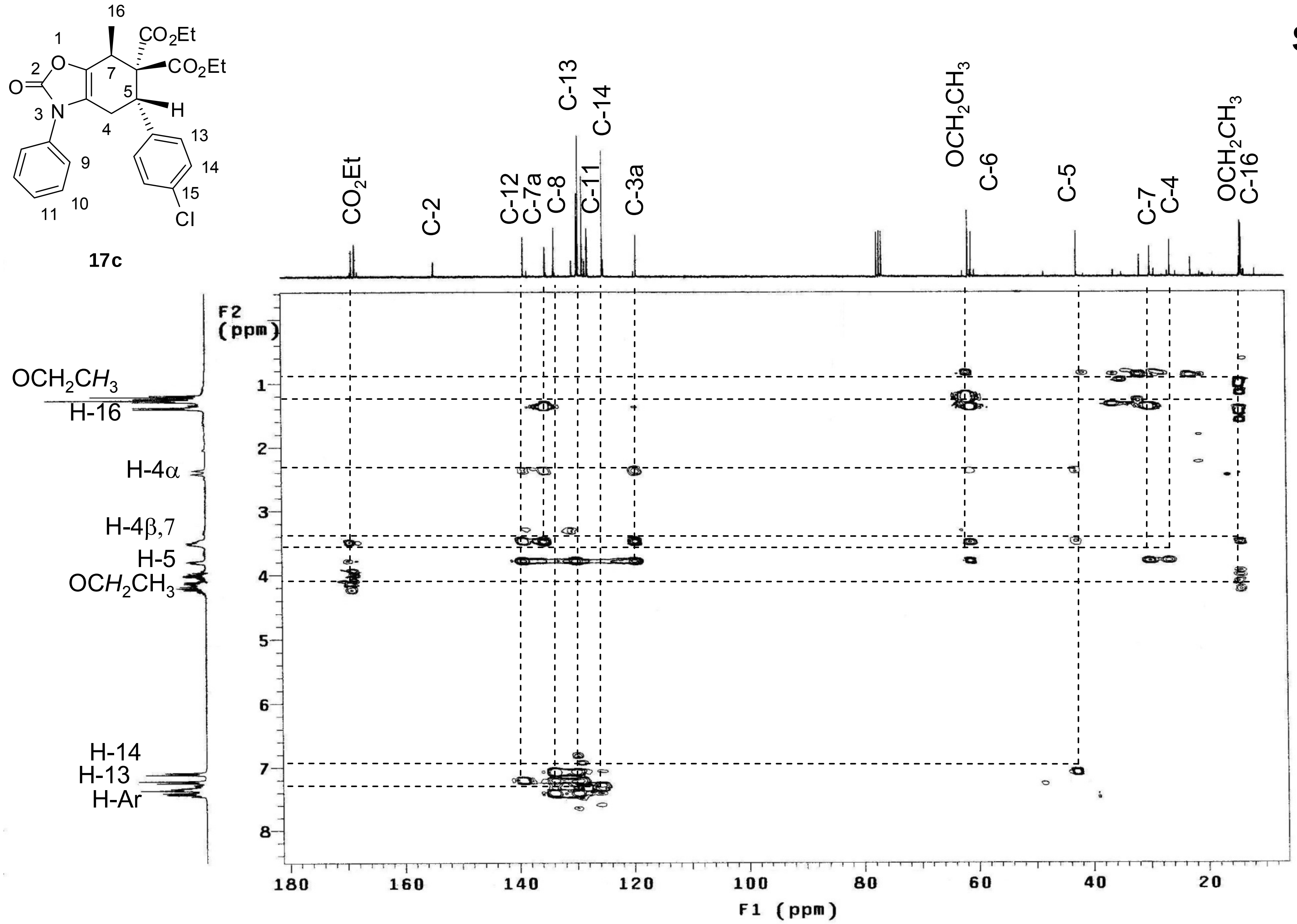

HMBC experiment ($CDCl_3$) **17c**

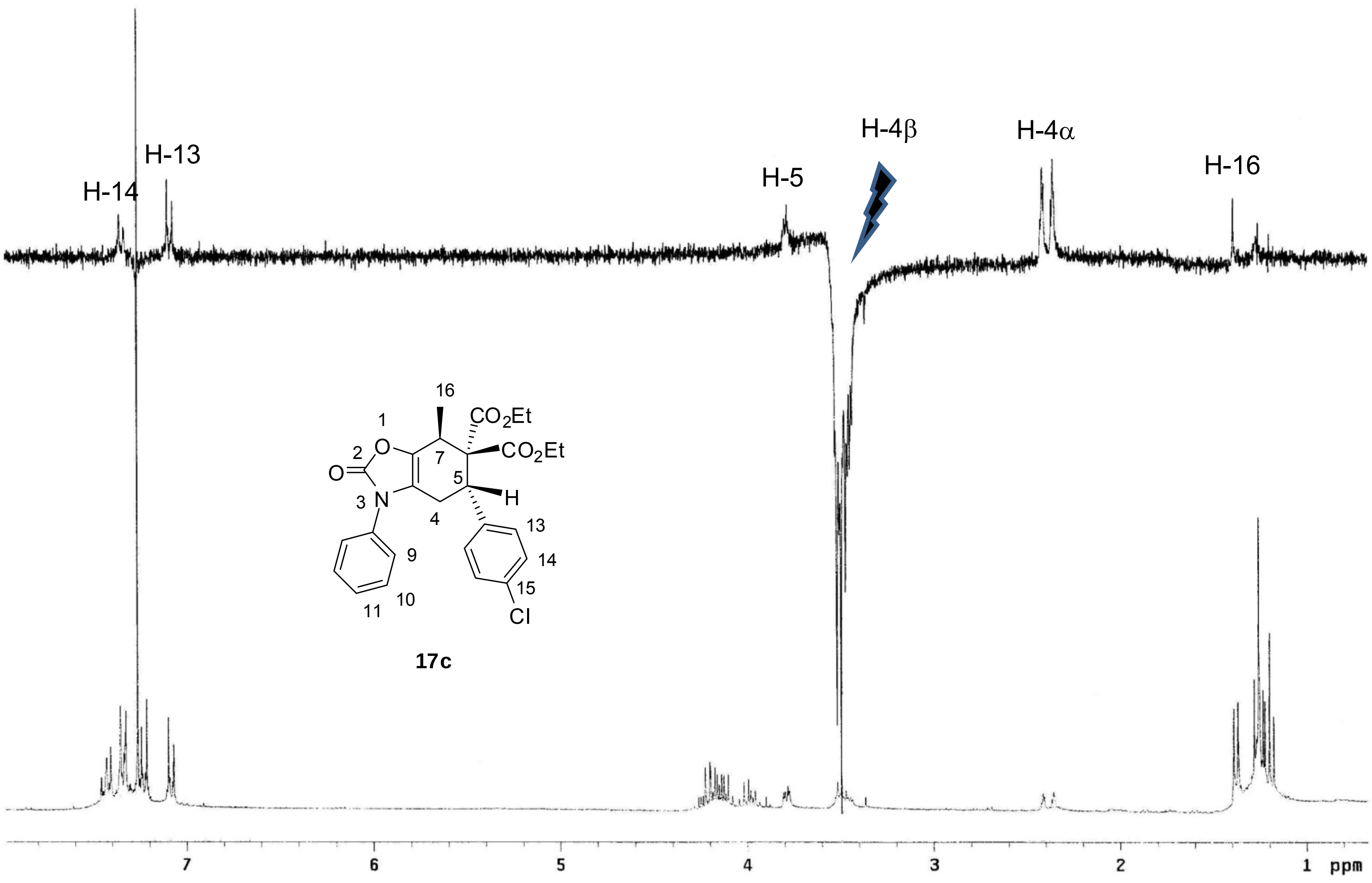

NOE experiment ($CDCl_3$) **17c**

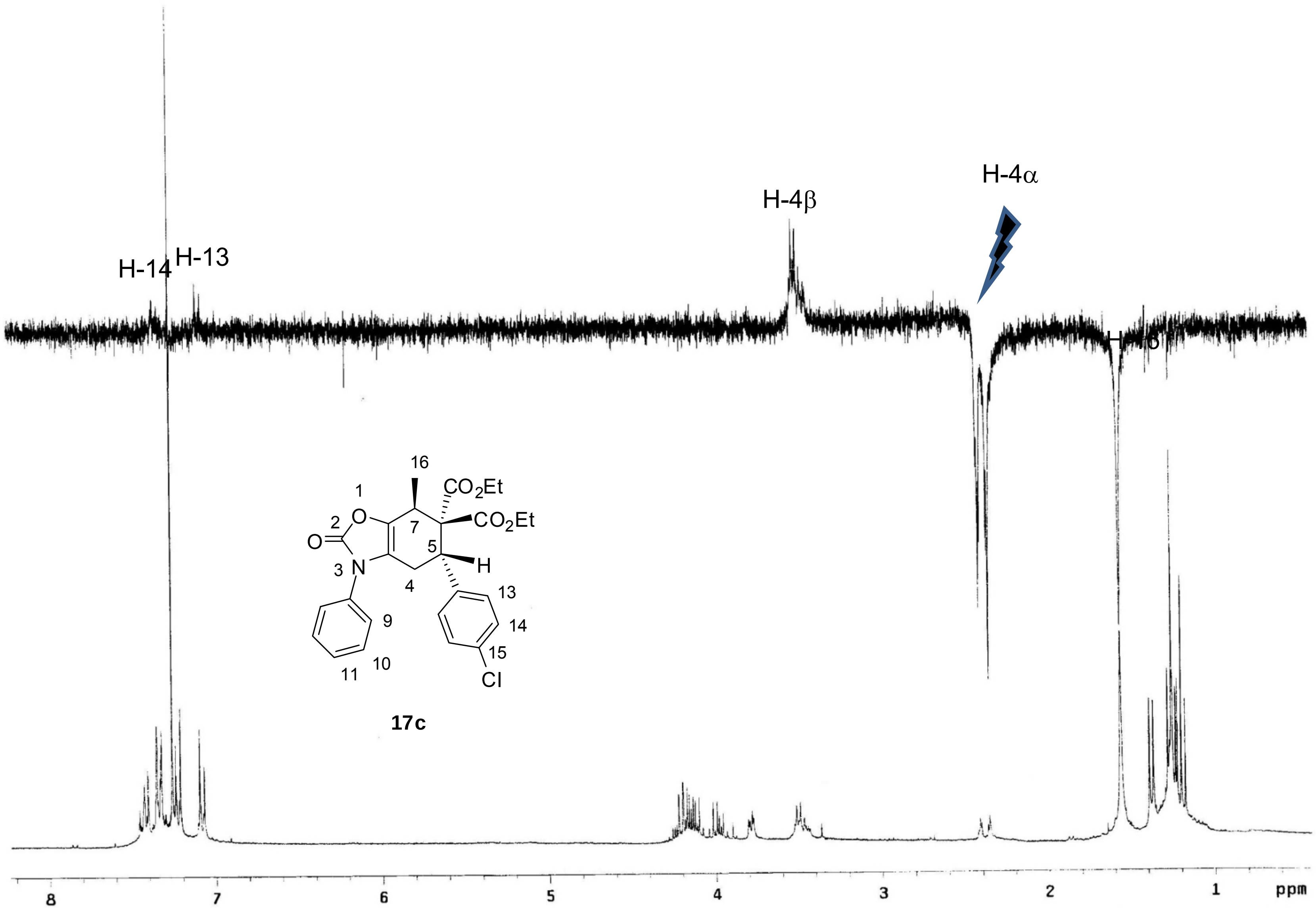

NOE experiment ($CDCl_3$) **17c**

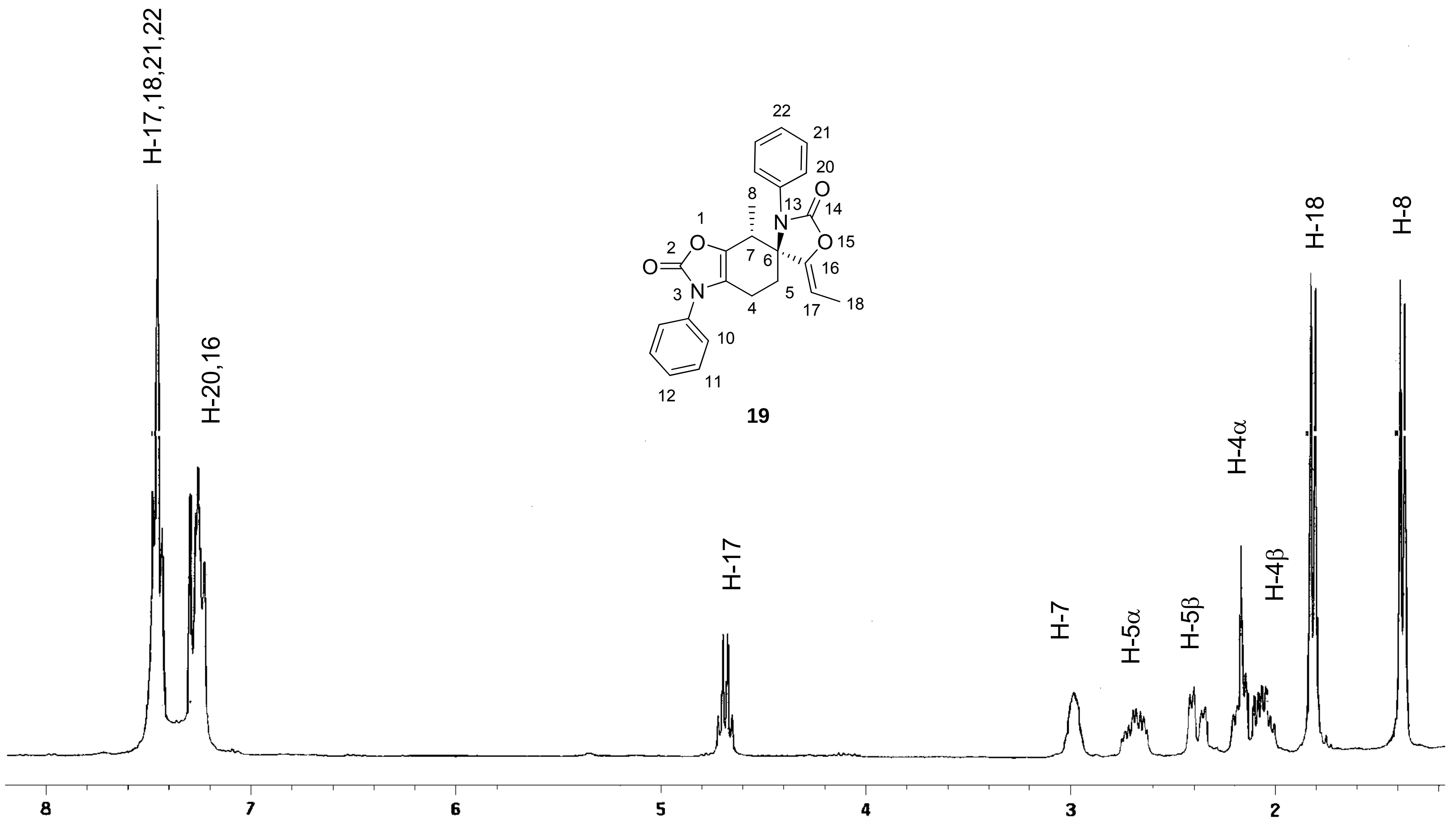
H-17,18,21,22
H-20,16
22
21
20
8
13
14
1
2
7
6
15
16
3
5
4
17
18
10
11
12
19
H-17
H-7
H-5α
H-5β
H-4α
H-4β
H-18
H-8
8
7
6
5
4
3
2

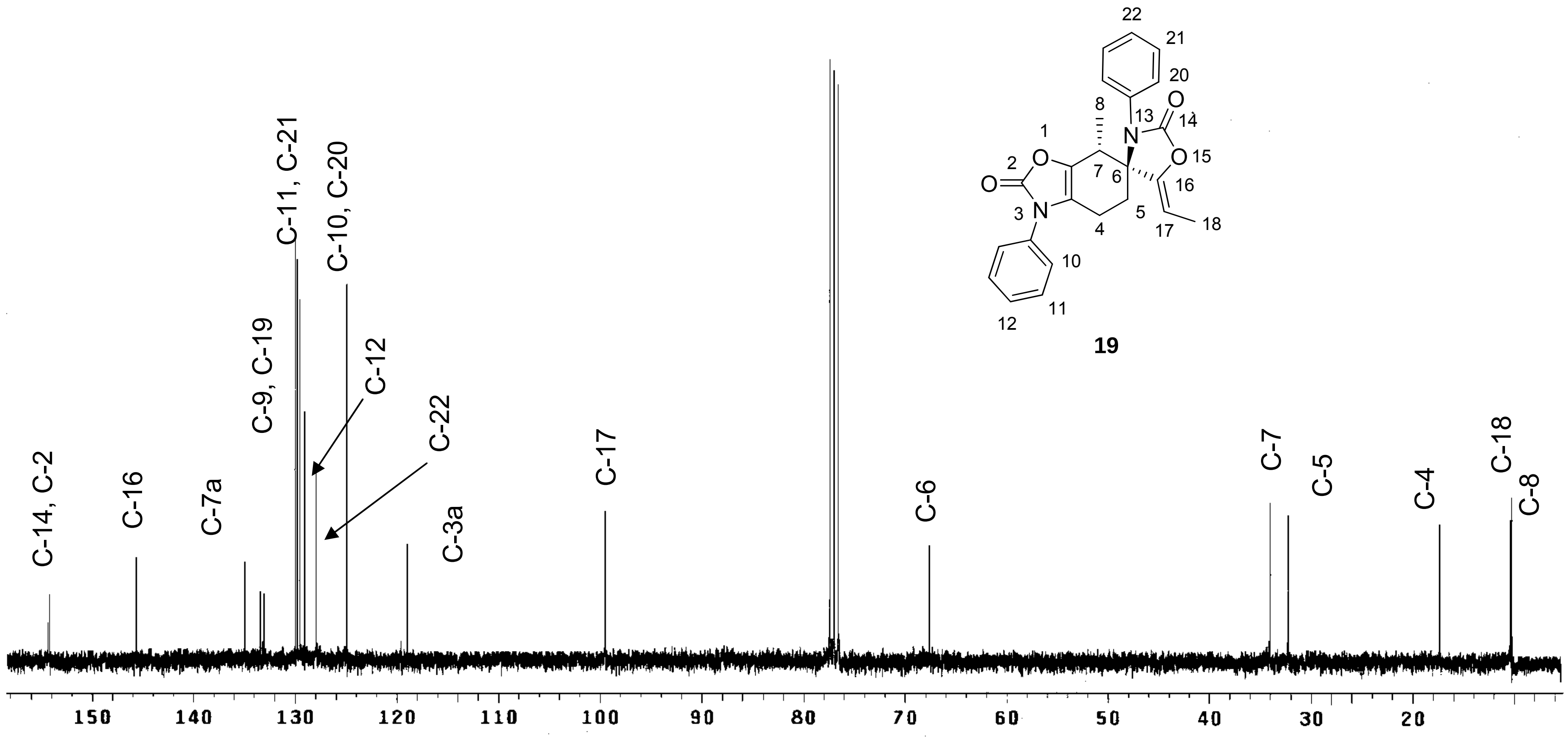

^{13}C NMR ($CDCl_3$) **19**

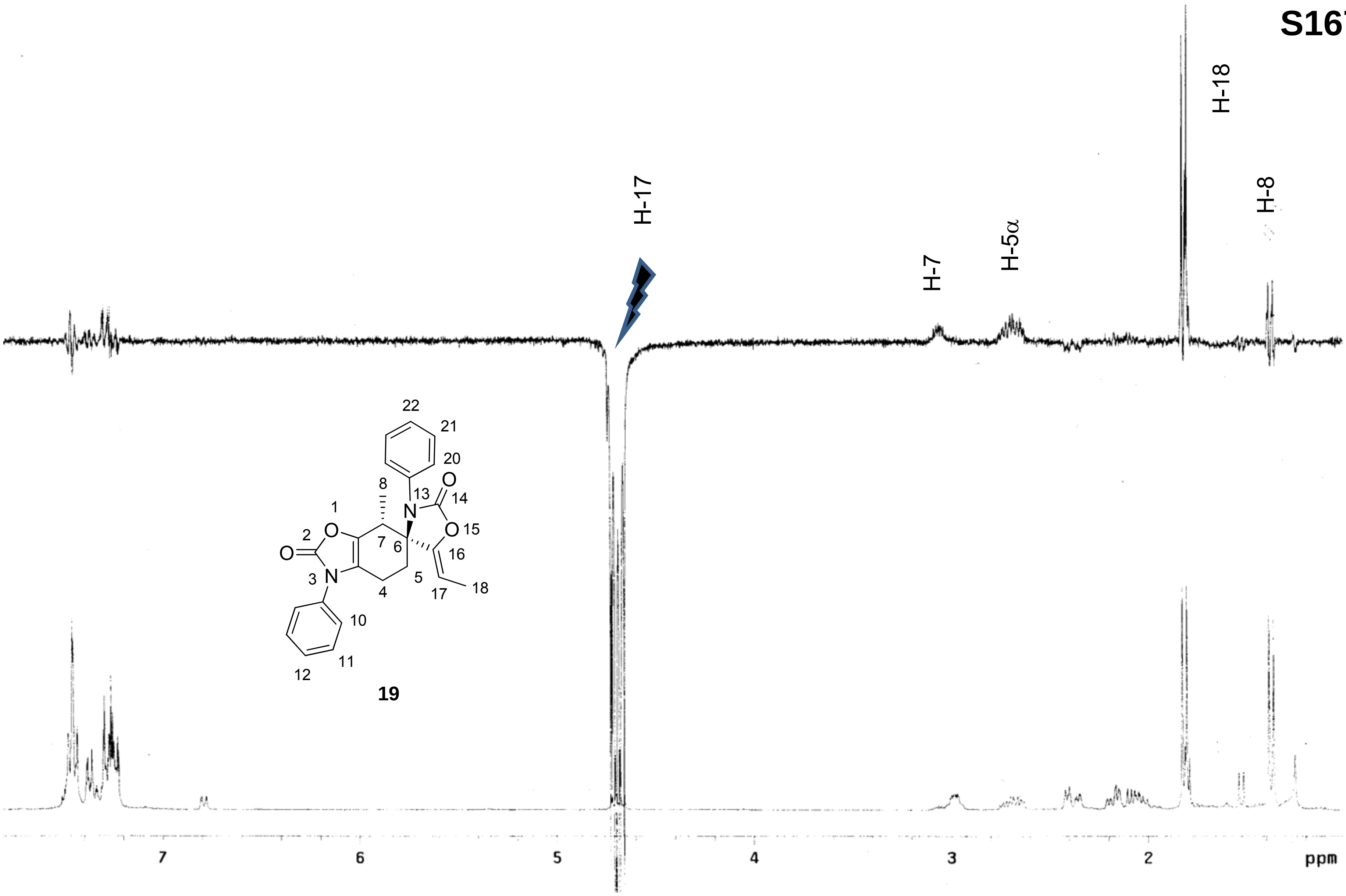

NOE experiment ($CDCl_3$) **19**

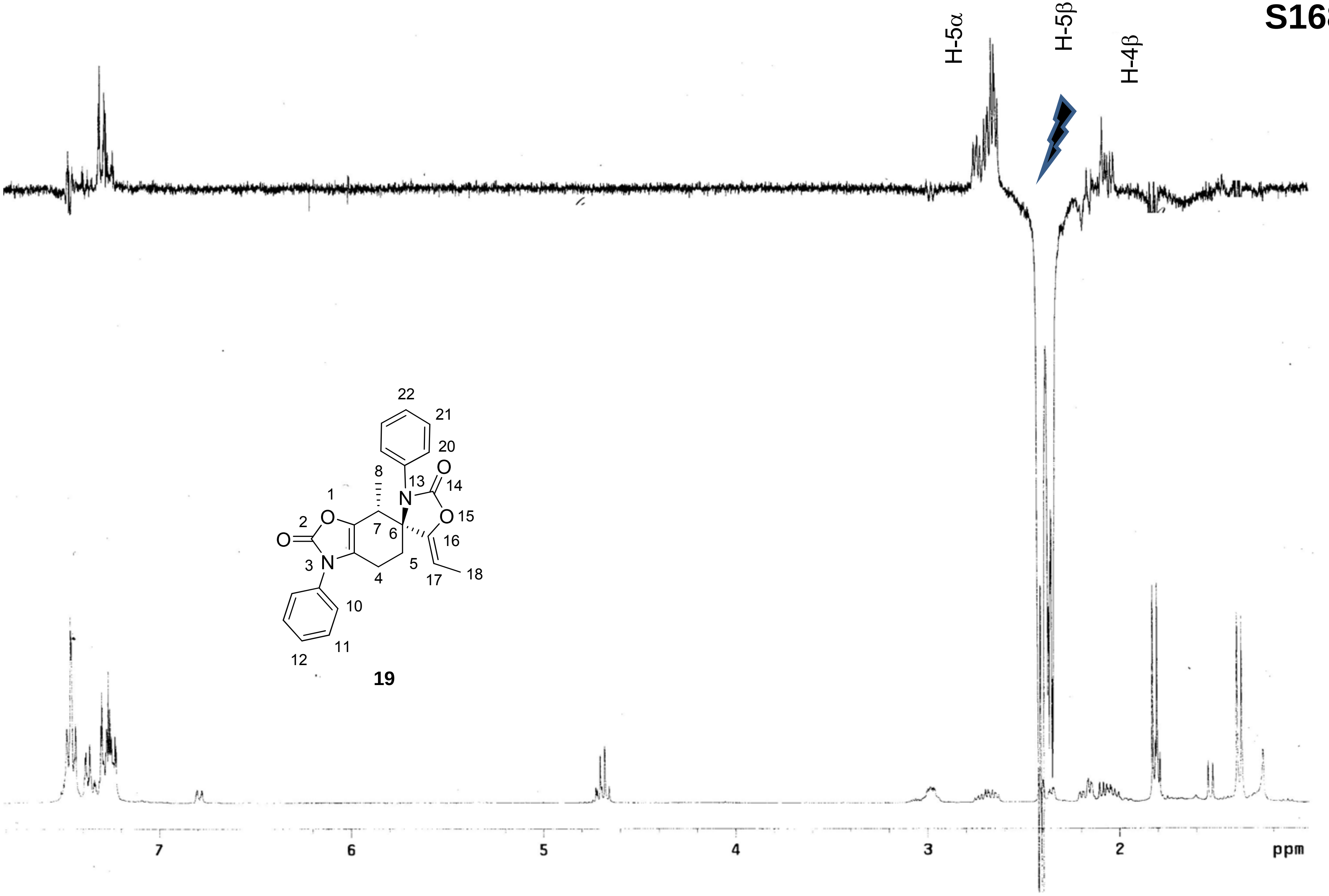

NOE experiment ($CDCl_3$) **19**

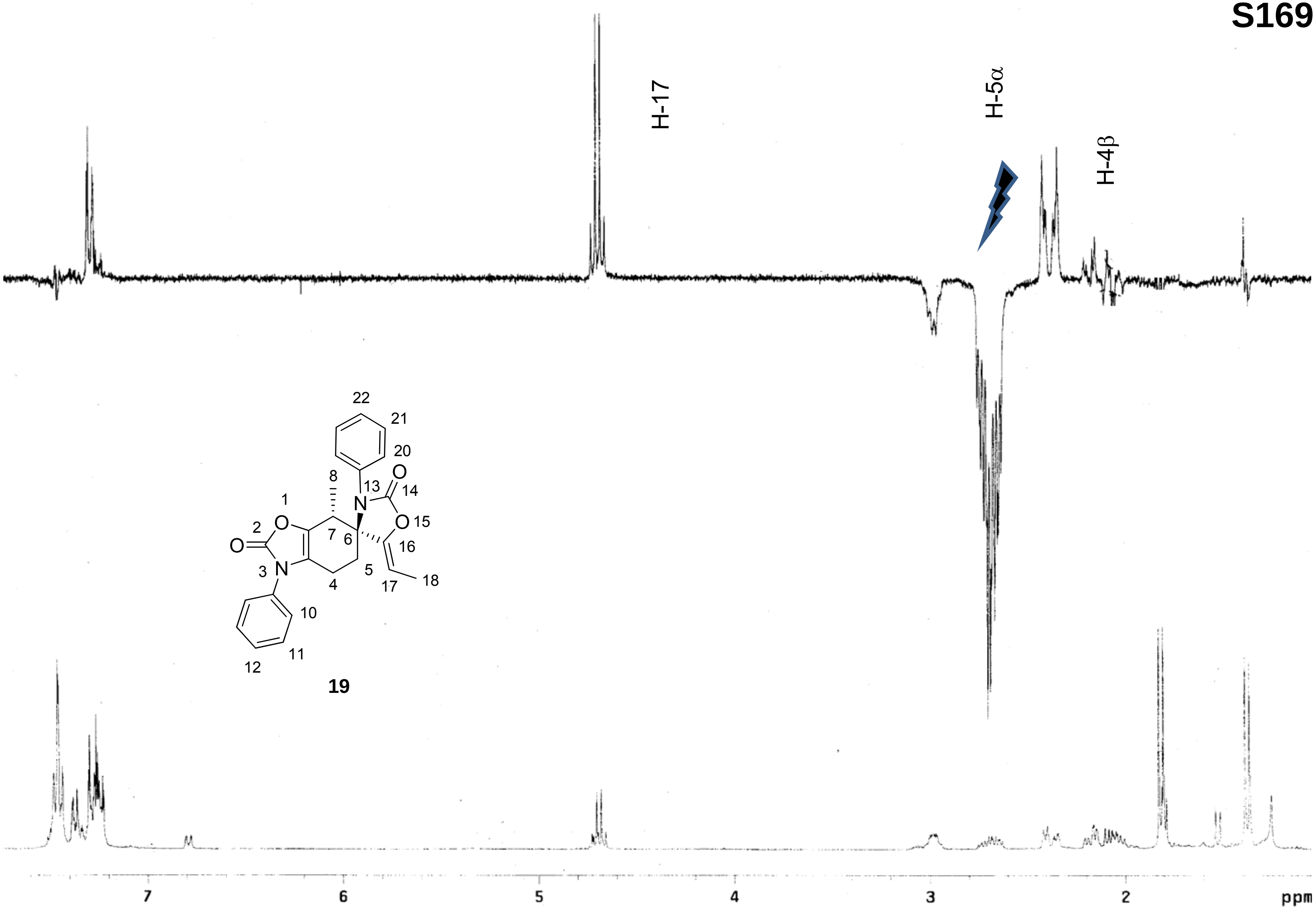

NOE experiment ($CDCl_3$) **19**

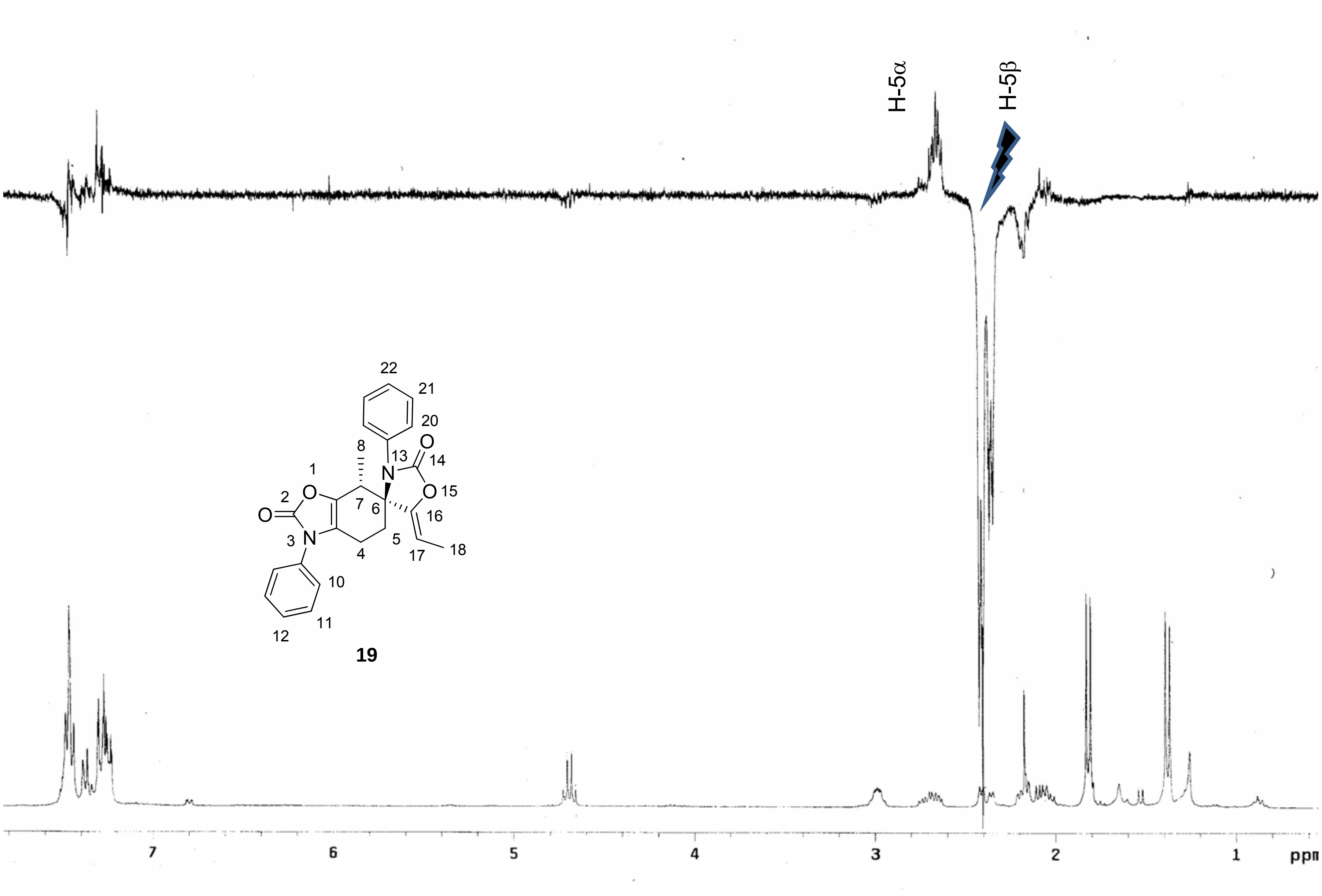

NOE experiment ($CDCl_3$) **19**

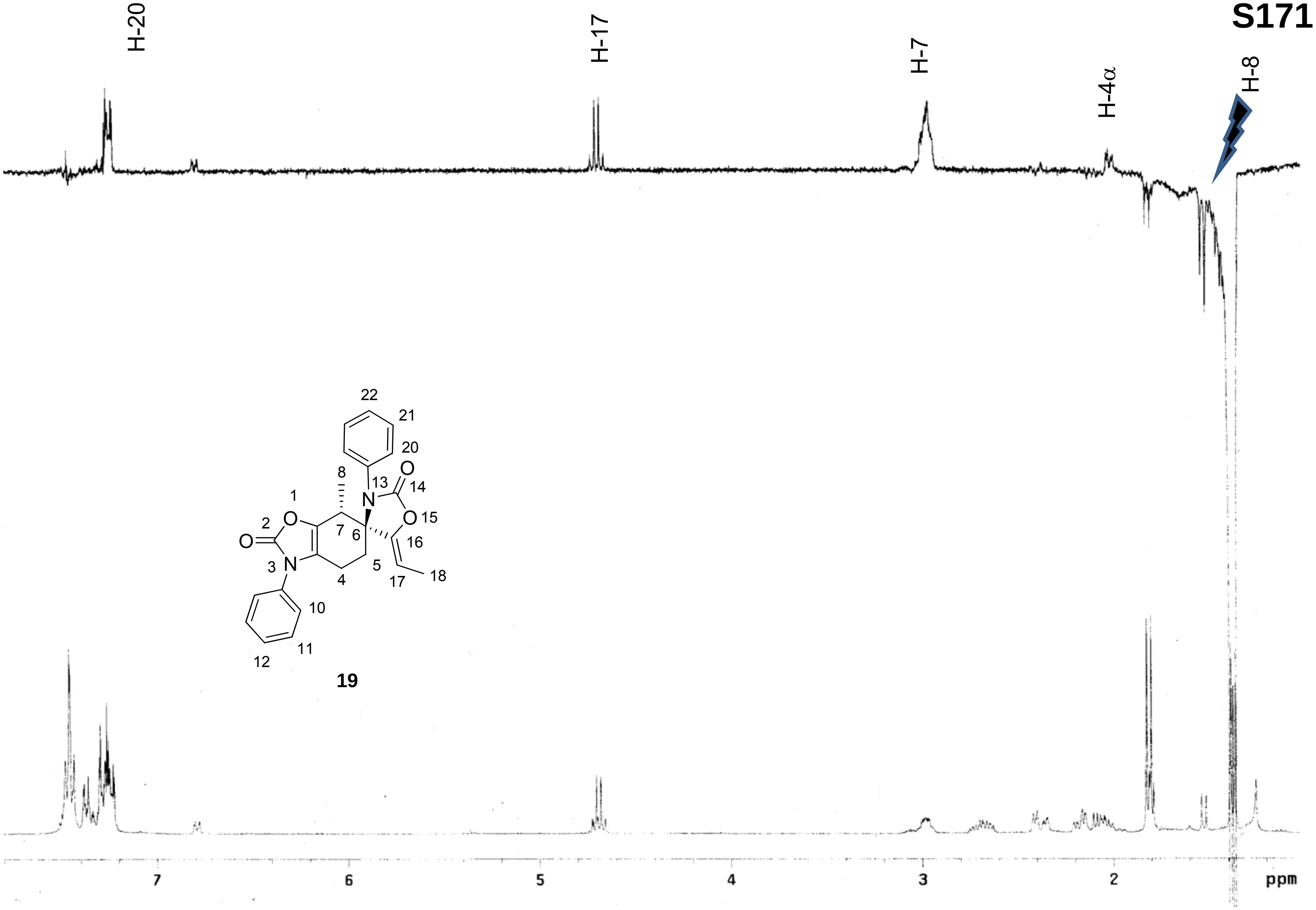

NOE experiment ($CDCl_3$) **19**

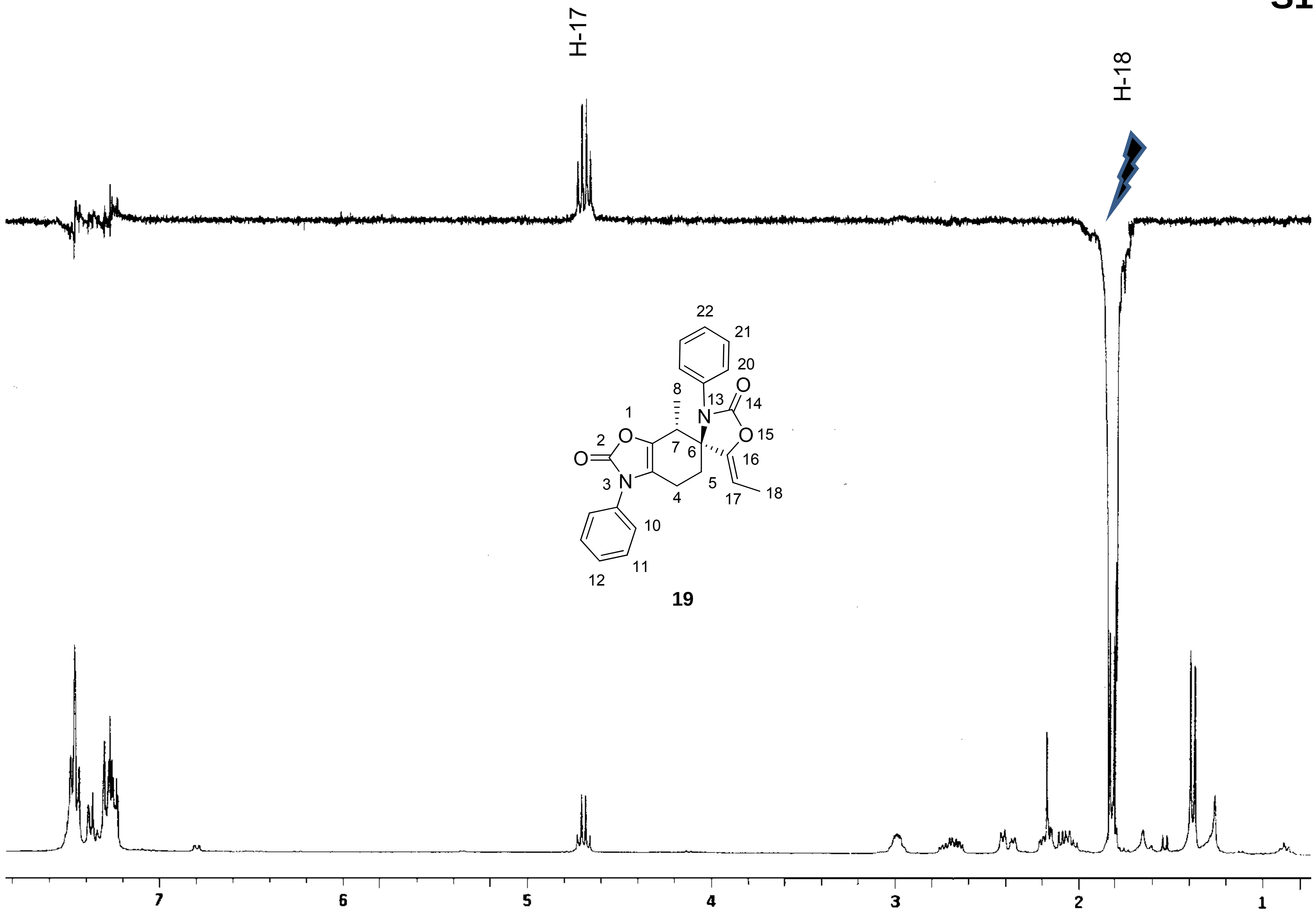

NOE experiment ($CDCl_3$) **19**

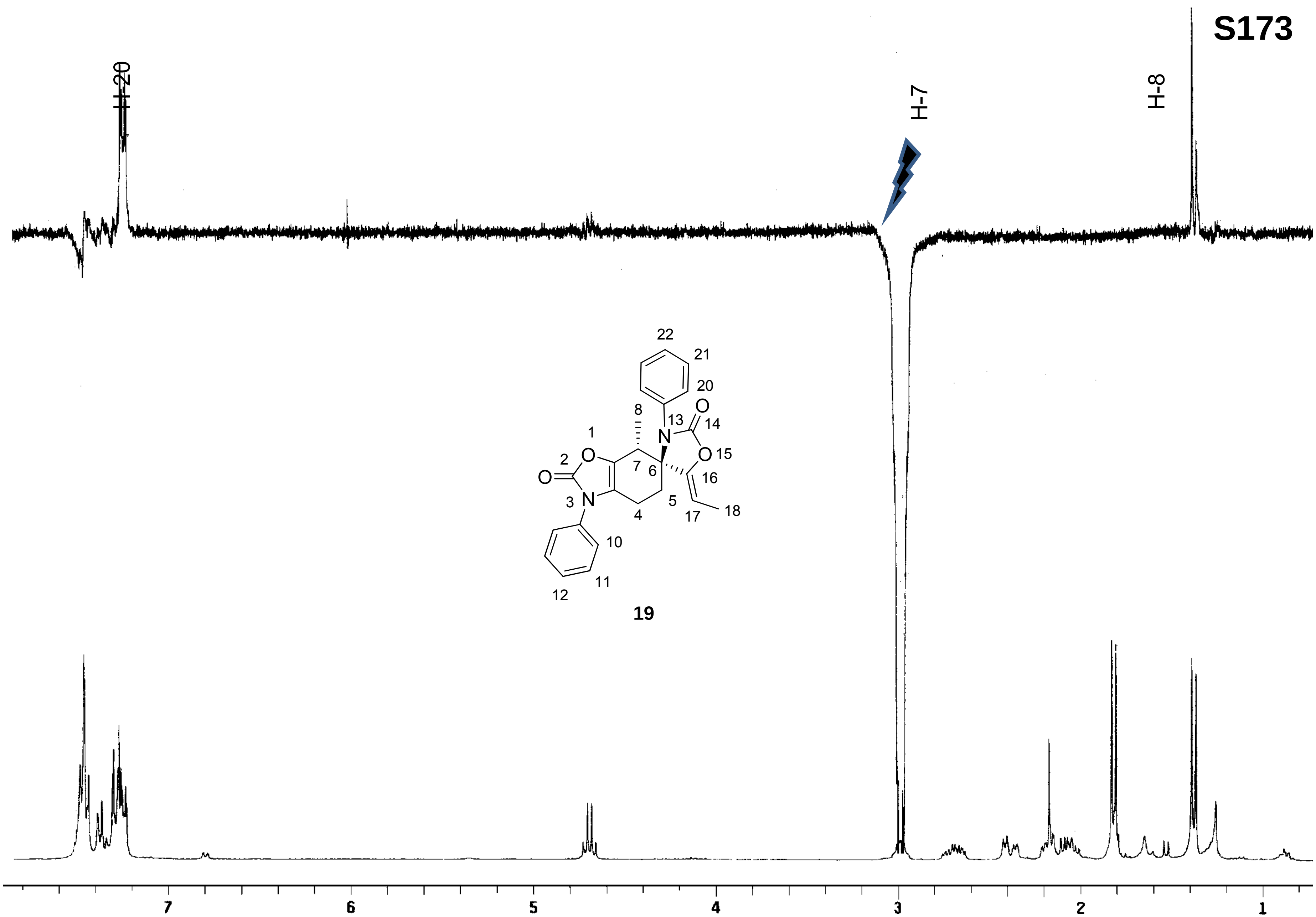

NOE experiment ($CDCl_3$) **19**

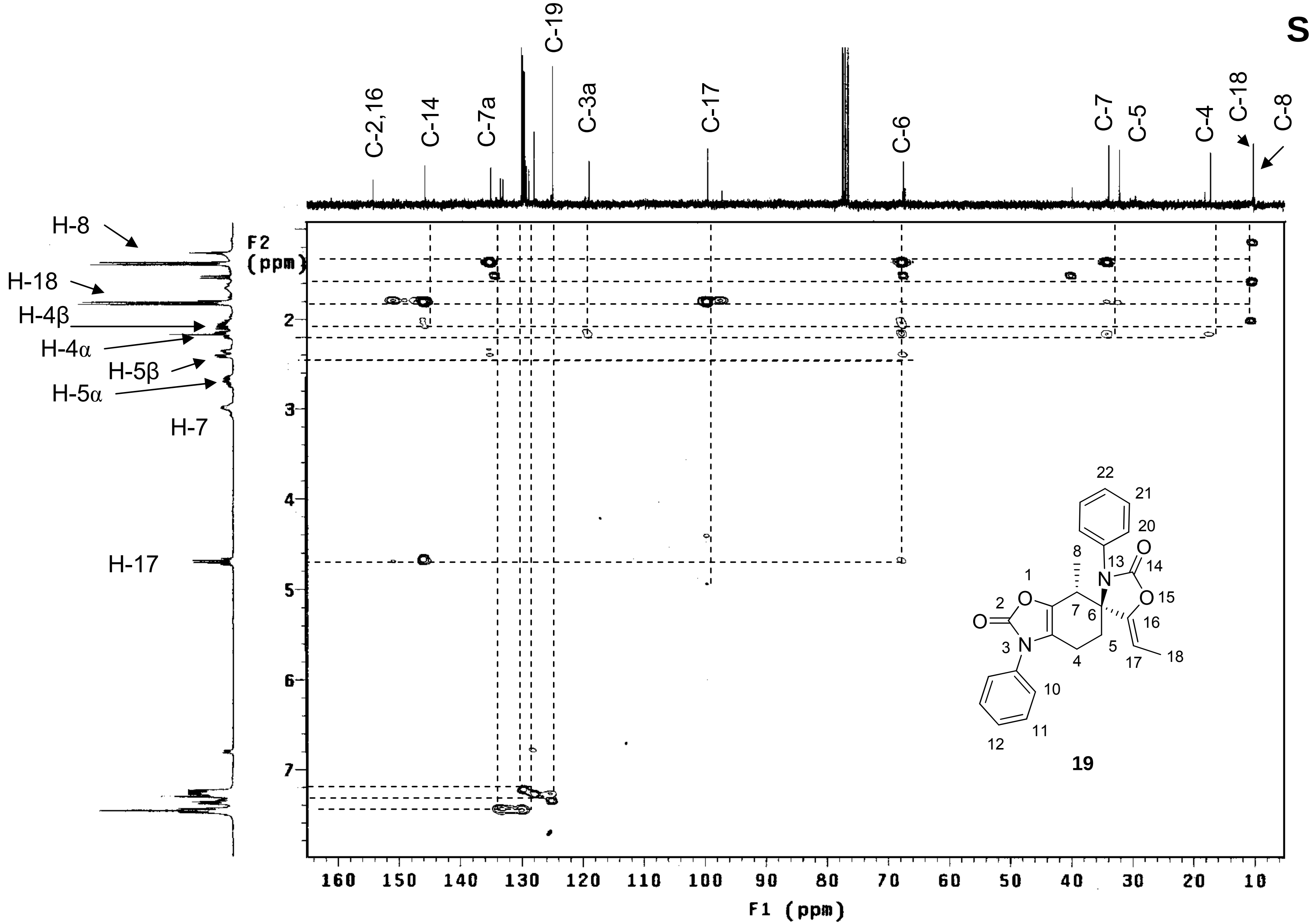

HMBC experiment ($CDCl_3$) **19**

Scan: 99-253 **R.T.: 2.05**

Base: m/z 402; 3.2%FS TIC: 1131560 **#Ions: 368**

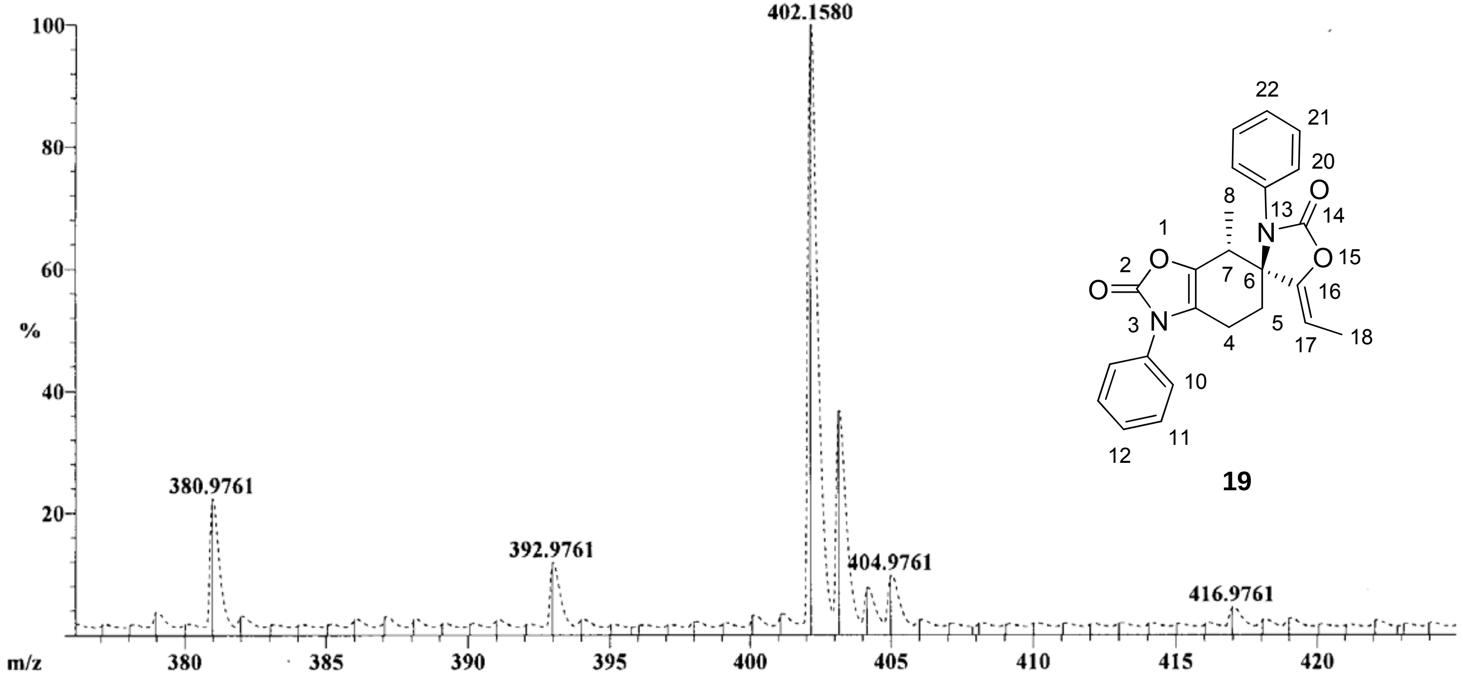

Selected Isotopes : $N_{0-2}O_{0-4}C_{0-24}H_{0-22}$ **Error Limit : 20 ppm**

Measured Mass	*% Base*	*Formula*	*Calculated Mass*	*Error*
402.1580	100.0%	$C_{24}H_{22}N_2O_4$	402.1580	0.1

HRMS **19**

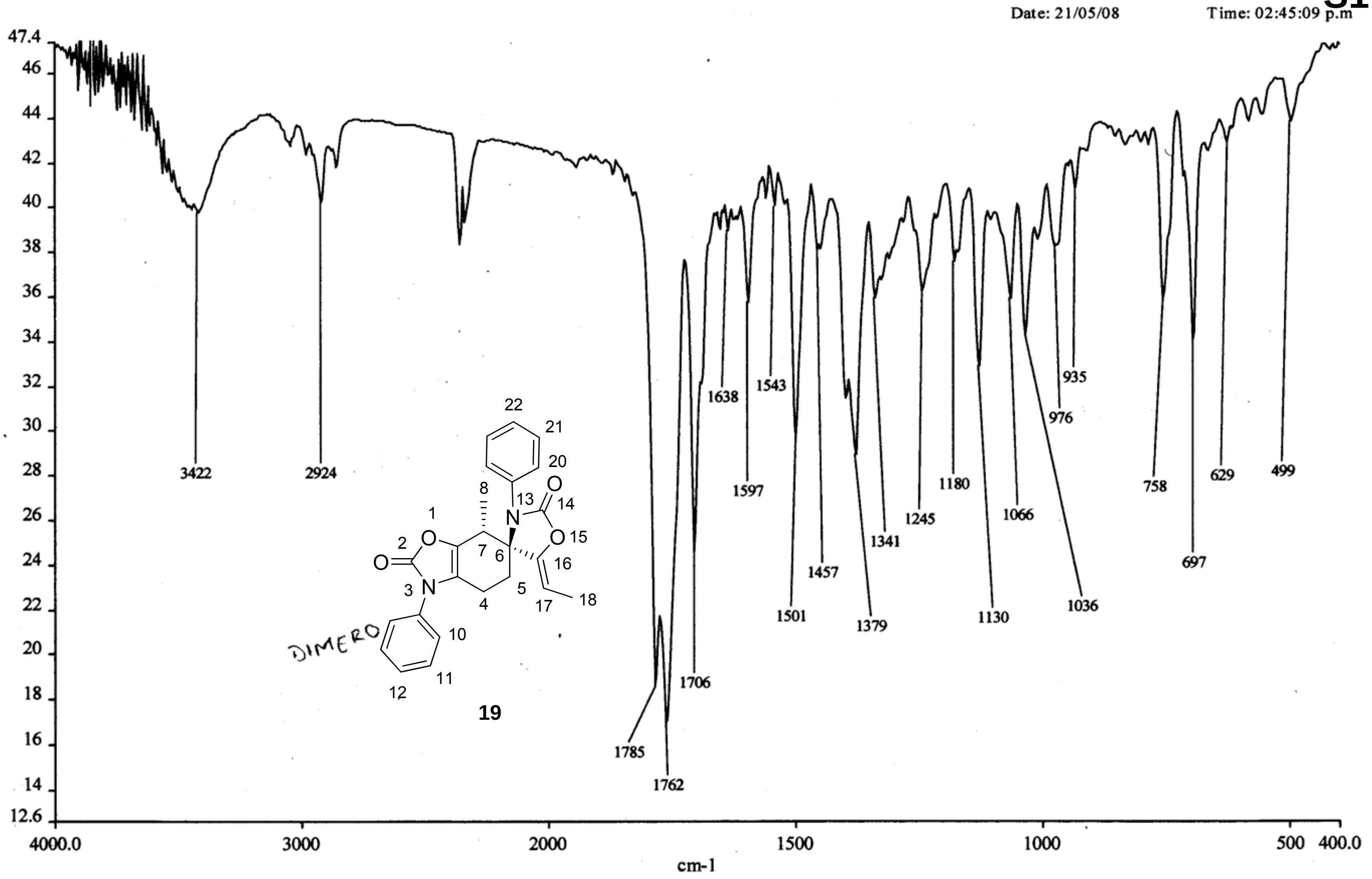
Date: 21/05/08
Time: 02:45:09 p.m
47.4
46
44
42
40
38
36
34
32
30
28
26
24
22
20
18
16
14
12.6
4000.0
3000
2000
1500
1000
500
400.0
cm-1
3422
2924
1785
1762
1706
1638
1597
1543
1501
1457
1379
1341
1245
1180
1130
1066
1036
976
935
758
697
629
499
DIMERO
19

FT-IR (KBr) **19**

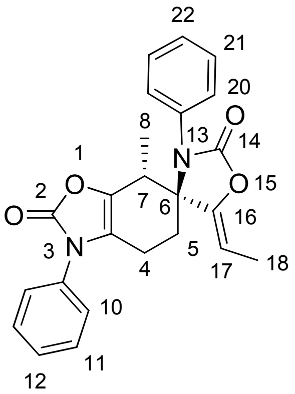

19

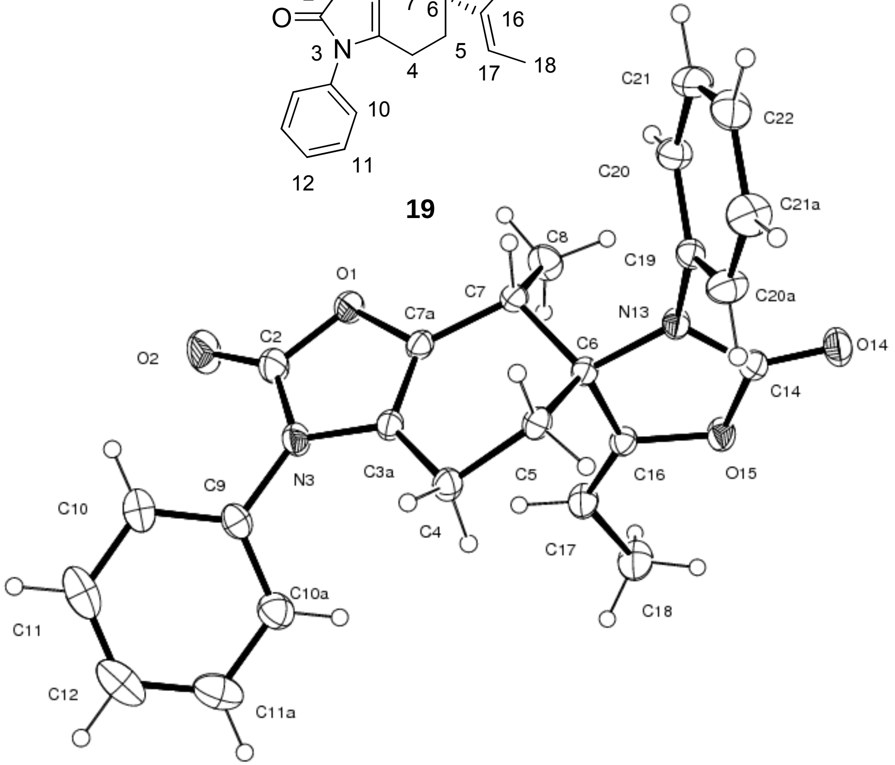

X-Ray structure for **19**

Table 1. Crystal data and structure refinement for adduct **19**.

Identification code		0027-fdr
Empirical formula		C24 H22 N2 O4
Formula weight		402.44
Temperature		292(2) K
Wavelength		0.71073 Å
Unit cell dimensions	a = 8.2082(2) Å	α= 84.819(3)°.
	b = 10.9272(4) Å	β= 89.340(3)°.
	c = 11.5021(5) Å	γ = 77.668(3)°.
Volume		1003.70(6) Å3
Z		2
Density (calculated)		1.332 Mg/m3
Absorption coefficient		0.091 mm-1
F(000)		424
Crystal size		0.65 x 0.48 x 0.39 mm3
Theta range for data collection		2.54 to 32.60°.
Index ranges		-8<=h<=12, -16<=k<=16, -17<=l<=16
Reflections collected		14631
Independent reflections		6514 [R(int) = 0.0155]
Completeness to theta = 27.50°		99.1 %
Max. and min. transmission		0.9652 and 0.9429

Refinement method	Full-matrix least-squares on F^2
Data / restraints / parameters	6514 / 0 / 273
Goodness-of-fit on F^2	1.049
Final R indices [I>2sigma(I)]	R1 = 0.0496, wR2 = 0.1340
R indices (all data)	R1 = 0.0699, wR2 = 0.1429
Largest diff. peak and hole	0.333 and -0.217 e.Å-3

Diene **1**

Zero-point correction =	0.187911 (Hartree/Particle)
Thermal correction to Energy =	0.197784
Thermal correction to Enthalpy =	0.198729
Thermal correction to Gibbs Free Energy =	0.152175
Sum of electronic and zero-point Energies =	-625.779944
Sum of electronic and thermal Energies =	-625.770070
Sum of electronic and thermal Enthalpies =	-625.769126
Sum of electronic and thermal Free Energies =	-625.815680

Coord:	X	Y	Z
C	-1.7282111725	1.2128823597	2.6041125253
C	-1.1704522631	-0.0000000096	2.1941389183
C	-1.7282108986	-1.2128825034	2.6041125448
C	-2.8482257518	-1.2095793408	3.4352532351
C	-3.4082142787	-0.0000002502	3.8503084354
C	-2.8482260386	1.2095789568	3.4352531984
N	-0.0262263313	0.0000001035	1.3349237547
C	-0.0618629833	-0.0000003636	-0.0716730271

C	1.3635953429	0.0000000395	-0.4579173059
O	2.1139841618	0.0000006745	0.7107076873
C	1.2780041484	0.0000007059	1.8083328245
C	-1.1531152169	-0.0000010159	-0.8476816168
O	1.6661675944	0.0000012541	2.9450108674
C	1.9464618321	-0.0000000913	-1.6562412518
H	3.0250393343	0.0000002817	-1.7466909743
H	-4.2789824595	-0.0000003436	4.4990261288
H	1.3479747662	-0.0000005866	-2.5576768999
H	-2.1488100156	-0.0000012287	-0.4230870663
H	-1.0572766818	-0.0000013569	-1.9254764087
H	-1.2784618013	2.1446260908	2.2768717833
H	-3.2802562419	2.1509397701	3.7603408821
H	-3.2802557389	-2.1509402438	3.7603409393
H	-1.2784613062	-2.1446261364	2.2768718264

Lowest vibrational frequencies:

	1	2	3
	A	A	A
Frequencies --	103.8414	111.9167	130.7086
Red. masses --	4.3179	4.2290	5.3862
Frc consts --	0.0274	0.0312	0.0542
IR Inten --	0.6600	0.5131	1.6266

Theoretical Calculations of Diene 2

Diene **2**	
Zero-point correction =	0.217658 (Hartree/Particle)
Thermal correction to Energy =	0.229030
Thermal correction to Enthalpy =	0.229974
Thermal correction to Gibbs Free Energy =	0.180188
Sum of electronic and zero-point Energies =	-664.790511
Sum of electronic and thermal Energies =	-664.779139
Sum of electronic and thermal Enthalpies =	-664.778195
Sum of electronic and thermal Free Energies =	-664.827981

Coord:	X	Y	Z
C	1.2646818000	0.0000015666	1.8248570084
N	-0.0366526204	0.0000003403	1.3457355905
C	-0.0697954679	-0.0000006156	-0.0625493487
C	1.3545813759	-0.0000001220	-0.4421873171
O	2.1048733172	0.0000011184	0.7310981655
C	-1.1828489578	0.0000001647	2.2021165023
C	-1.7418101133	1.2127037626	2.6109915024
C	-2.8634970041	1.2094863408	3.4397991982
C	-3.4243504001	-0.0000002250	3.8540049707

C	-2.8634971576	-1.2094866093	3.4397984292
C	-1.7418102475	-1.2127036297	2.6109907651
C	-1.1648982410	-0.0000017320	-0.8344130475
C	1.9518471551	-0.0000006752	-1.637277573
O	1.6481341922	0.0000024083	2.9639391056
C	3.4426401345	-0.0000000610	-1.831289221
H	-4.2964150958	-0.0000003759	4.5009722281
H	1.3060636015	-0.0000016495	-2.5094410399
H	-2.1584199340	-0.0000020003	-0.4050131329
H	-1.0746368309	-0.0000023897	-1.9126855725
H	-1.2915630993	2.1444100838	2.2843685915
H	-3.2962161806	2.1509374801	3.7638466675
H	-3.2962164582	-2.1509378942	3.7638453051
H	-1.2915633255	-2.1444097993	2.2843672849
H	3.6997100883	0.0000001790	-2.8925672227
H	3.9066743845	0.8788442006	-1.3690562839
H	3.9066750848	-0.8788441058	-1.3690565558

Lowest vibrational frequencies:

	1	2	3
	A	A	A
Frequencies --	82.6455	103.6999	117.2721
Red. masses --	4.3210	4.2396	4.9071
Frc consts --	0.0174	0.0269	0.0398
IR Inten --	1.0520	0.4511	1.0603

Theoretical Calculations of Diene 3

Diene **3**

Zero-point correction =	0.207560 (Hartree/Particle)
Thermal correction to Energy =	0.220123
Thermal correction to Enthalpy =	0.221067
Thermal correction to Gibbs Free Energy =	0.168176
Sum of electronic and zero-point Energies =	-1123.697456
Sum of electronic and thermal Energies =	-1123.684893
Sum of electronic and thermal Enthalpies =	-1123.683949
Sum of electronic and thermal Free Energies =	-1123.736840

Coord:	X	Y	Z
C	-1.6834465688	1.2838429165	2.5512378520
N	-1.1327734599	0.0525855446	2.1422720832
C	-1.8390793676	-1.0215546479	2.6668206952
O	-2.8638670330	-0.5052674429	3.4278946490
C	-2.8230817563	0.8875491943	3.3976049934
C	0.0067076478	-0.1061957866	1.2960192861
C	1.2843983206	-0.1778012371	1.8551093441
C	2.3998978564	-0.3264275538	1.0332774356
C	2.2203880603	-0.3985404513	-0.3480037592
C	0.9499450663	-0.3264272416	-0.9190870177
C	-0.1592260121	-0.1778009147	-0.0887338283

O	-1.6377521253	-2.1967997843	2.5173018414
C	-3.7236796112	1.6129752848	4.0664475667
C	-1.2398108654	2.5042288107	2.2217652532
C	-4.8335993217	1.0228907324	4.8907460259
H	-3.6326639382	2.6922922004	3.9988535159
H	-0.3776131654	2.6391995518	1.5814412091
H	-1.7411707034	3.3878197325	2.5941077300
H	-1.1566233115	-0.1241730404	-0.512101672
H	0.8321394631	-0.3887614023	-1.9947495879
Cl	3.6221998288	-0.5845533672	-1.3890803615
H	3.3956007496	-0.3887619573	1.4569563742
H	1.4014163645	-0.1241736099	2.932304084
H	-5.4441116017	1.8064396509	5.344152152
H	-5.4898999590	0.3891344478	4.2833875338
H	-4.4418555714	0.3891343708	5.6945815818

Lowest vibrational frequencies:

	1	2	3
	A"	A'	A"
Frequencies --	74.0418	83.5076	105.4317
Red. masses --	4.7760	8.1362	4.2466
Frc consts --	0.0154	0.0334	0.0278
IR Inten --	0.2252	0.4292	0.4195

Theoretical Calculations of compound 4a

Compound **4a**

Zero-point correction =	0.217981 (Hartree/Particle)
Thermal correction to Energy =	0.229972
Thermal correction to Enthalpy =	0.230916
Thermal correction to Gibbs Free Energy =	0.179543
Sum of electronic and zero-point Energies =	-664.800316
Sum of electronic and thermal Energies =	-664.788324
Sum of electronic and thermal Enthalpies =	-664.787380
Sum of electronic and thermal Free Energies =	-664.838753

Coord:	X	Y	Z
C	0.3444967272	0.0000000000	-0.4590285901
C	0.1890292679	0.0000000000	0.8928972551
C	1.7489097610	0.0000000000	-0.9842574692
H	1.1385802006	0.0000000000	1.4248824681
C	-0.9918687171	0.0000000000	1.741753977
C	-0.7235649543	0.0000000000	-1.4071337536
N	-1.5978158497	0.0000000000	-2.1765319386
C	-0.7708904464	0.0000000000	3.1359461253
C	-1.8333163567	0.0000000000	4.0329653973

C	-3.1447327774	0.0000000000	3.553951131
C	-3.3829794474	0.0000000000	2.1764417401
C	-2.3240640172	0.0000000000	1.2750068442
H	0.2502393686	0.0000000000	3.5072897057
H	-1.6405260802	0.0000000000	5.101278681
H	-3.9784371821	0.0000000000	4.2498390788
H	-4.4020785627	0.0000000000	1.8021983846
H	-2.5315491168	0.0000000000	0.2126576361
O	1.7723630535	0.0000000000	-2.3266590884
O	2.7396861743	0.0000000000	-0.2806017041
C	3.0853589294	0.0000000000	-2.9405154667
C	2.8831039359	0.0000000000	-4.4423623826
H	3.6311512668	0.8830630447	-2.5944464976
H	3.6311512668	-0.8830630447	-2.5944464976
H	3.8552880599	0.0000000000	-4.9447234348
H	2.3288032378	-0.8864296822	-4.7623542305
H	2.3288032378	0.8864296822	-4.7623542305

Lowest vibrational frequencies:

	1	2	3
	A"	A"	A'
Frequencies --	72.1003	105.8742	112.6419
Red. masses --	5.2515	4.4336	4.4621
Frc consts --	0.0161	0.0293	0.0334
IR Inten --	2.9389	0.0612	1.5674

Theoretical Calculations of compound 4b

Compound **4b**

Zero-point correction =	0.252919 (Hartree/Particle)
Thermal correction to Energy =	0.267034
Thermal correction to Enthalpy =	0.267979
Thermal correction to Gibbs Free Energy =	0.212078
Sum of electronic and zero-point Energies =	-778.650725
Sum of electronic and thermal Energies =	-778.636609
Sum of electronic and thermal Enthalpies =	-778.635665
Sum of electronic and thermal Free Energies =	-778.691565

Coord:	X	Y	Z
C	-1.6978135678	0.0000000000	0.6458950812
C	-0.3857583453	0.0000000000	1.1637518760
C	-0.2389916448	0.0000000000	2.5721632225
C	-1.3323365658	0.0000000000	3.4167956458
C	-2.6306996443	0.0000000000	2.8786388003
C	-2.8050710570	0.0000000000	1.4851551718
C	0.8255279996	0.0000000000	0.3718647013
C	1.0433667009	0.0000000000	-0.9749442489
C	0.0154326749	0.0000000000	-1.9649978163
N	-0.8335411479	0.0000000000	-2.7628847064
C	2.4657778373	0.0000000000	-1.4360279853
O	3.4271668412	0.0000000000	-0.6914623254
O	2.5501137803	0.0000000000	-2.7780817848

C	3.8882573513	0.0000000000	-3.3313834649
C	3.7535978449	0.0000000000	-4.8411547187
H	1.7512651204	0.0000000000	0.9446227487
H	0.7608815072	0.0000000000	2.9971566138
H	-1.2167447529	0.0000000000	4.4950990046
O	-3.6410586371	0.0000000000	3.7813847022
H	-3.7963710335	0.0000000000	1.0489302683
H	-1.8607397929	0.0000000000	-0.4243872835
H	4.4192001070	0.8829012423	-2.9623290768
H	4.4192001070	-0.8829012423	-2.9623290768
H	4.7470640548	0.0000000000	-5.3001474423
H	3.2137590177	-0.8863371653	-5.1853087927
H	3.2137590177	0.8863371653	-5.1853087927
C	-4.9839466810	0.0000000000	3.3074732518
H	-5.6131435615	0.0000000000	4.1978055003
H	-5.1985875238	0.8945534683	2.7111437186
H	-5.1985875238	-0.8945534683	2.7111437186

Lowest vibrational frequencies:

	1	2	3
	A"	A'	A"
Frequencies --	64.1322	99.9633	103.0605
Red. masses --	5.3365	4.5588	5.0958
Frc consts --	0.0129	0.0268	0.0319
IR Inten --	2.1485	1.1931	0.8888

Theoretical Calculations of compound 4c

Compound **4c**

Zero-point correction =	0.207856 (Hartree/Particle)
Thermal correction to Energy =	0.221054
Thermal correction to Enthalpy =	0.221998
Thermal correction to Gibbs Free Energy =	0.167452
Sum of electronic and zero-point Energies =	-1123.707196
Sum of electronic and thermal Energies =	-1123.693998
Sum of electronic and thermal Enthalpies =	-1123.693054
Sum of electronic and thermal Free Energies =	-1123.747600

Coord:	X	Y	Z
C	-1.7299356020	0.0000000000	0.6601508968
C	-0.3973768592	0.0000000000	1.1259712969
C	-0.1823602059	0.0000000000	2.5205627591
C	-1.2400741131	0.0000000000	3.4209750972
C	-2.5446606191	0.0000000000	2.9265185111
C	-2.7949671253	0.0000000000	1.5520978893
C	0.7844450852	0.0000000000	0.2795395869
C	0.9387788269	0.0000000000	-1.0722261900
C	-0.1315043956	0.0000000000	-2.0173570071
N	-1.0112131804	0.0000000000	-2.7805225843
C	2.3435621004	0.0000000000	-1.5985861040

O	3.3333315443	0.0000000000	-0.8939012062
O	2.3650886184	0.0000000000	-2.9401238218
C	3.6770143468	0.0000000000	-3.5575966059
C	3.4706055128	0.0000000000	-5.0588765159
H	1.7337016166	0.0000000000	0.8119148865
H	0.8356244071	0.0000000000	2.8993059625
H	-1.0612323066	0.0000000000	4.4900050913
Cl	-3.8906587248	0.0000000000	4.0473733566
H	-3.8158427018	0.0000000000	1.1876145714
H	-1.9404715528	0.0000000000	-0.401579774
H	4.2231499048	0.8832657899	-3.2128312379
H	4.2231499048	-0.8832657899	-3.2128312379
H	4.4416289415	0.0000000000	-5.5633841444
H	2.9157296797	-0.8865540904	-5.3773722754
H	2.9157296797	0.8865540904	-5.3773722754

Lowest vibrational frequencies:

	1	2	3
	A"	A"	A'
Frequencies --	61.9972	94.0222	97.4843
Red. masses --	6.5273	6.8773	5.5938
Frc consts --	0.0148	0.0358	0.0313
IR Inten --	0.4428	3.9570	0.2736

Theoretical Calculations of compound 4d

Compound **4d**

Zero-point correction =	0.221067 (Hartree/Particle)
Thermal correction to Energy =	0.235151
Thermal correction to Enthalpy =	0.236095
Thermal correction to Gibbs Free Energy =	0.179925
Sum of electronic and zero-point Energies =	-868.257250
Sum of electronic and thermal Energies =	-868.243167
Sum of electronic and thermal Enthalpies =	-868.242222
Sum of electronic and thermal Free Energies =	-868.298392

Coord:	X	Y	Z
C	-2.4368404972	0.0000000000	-1.6188905457
C	-1.1412206808	0.0000000000	-1.1189213455
C	-0.9092176029	0.0000000000	0.2738484332
C	-2.0223453113	0.0000000000	1.1425021285
C	-3.3215886542	0.0000000000	0.6535285972
C	-3.5078504873	0.0000000000	-0.7264984476
C	0.4073767643	0.0000000000	0.8999855018
C	1.6606325934	0.0000000000	0.3761398341
C	2.8073648352	0.0000000000	1.3499811372
O	3.9865151318	0.0000000000	0.7153382983
C	5.1683611442	0.0000000000	1.5590128184
C	6.3764324059	0.0000000000	0.6447182488

N	-4.8823705340	0.0000000000	-1.2597421032
C	1.9687764509	0.0000000000	-1.0184356696
N	2.2137106347	0.0000000000	-2.1566919061
O	2.6678839768	0.0000000000	2.5560771492
H	0.4030049001	0.0000000000	1.9881221015
H	-1.8571351653	0.0000000000	2.2155318677
H	-4.1817866032	0.0000000000	1.3104965052
H	-2.6313389212	0.0000000000	-2.6837944179
H	-0.3122733549	0.0000000000	-1.8141820702
H	5.1323799444	0.8835425979	2.2032089511
H	5.1323799444	-0.8835425979	2.2032089511
H	7.2909183905	0.0000000000	1.2454662774
H	6.3832018756	-0.8867073918	0.0052796159
H	6.3832018756	0.8867073918	0.0052796159
O	-5.0180928665	0.0000000000	-2.4818296285
O	-5.8064586611	0.0000000000	-0.4477612537

Lowest vibrational frequencies:

	1	2	3
	A"	A"	A'
Frequencies --	63.6055	95.5032	100.7345
Red. masses --	5.9222	6.4012	5.1735
Frc consts --	0.0141	0.0344	0.0309
IR Inten --	0.1246	6.3174	0.0364

Theoretical Calculations of compound 5a

Compound **5a**

Zero-point correction =	0.140903 (Hartree/Particle)
Thermal correction to Energy =	0.149419
Thermal correction to Enthalpy =	0.150363
Thermal correction to Gibbs Free Energy =	0.106715
Sum of electronic and zero-point Energies =	-490.910519
Sum of electronic and thermal Energies =	-490.902003
Sum of electronic and thermal Enthalpies =	-490.901058
Sum of electronic and thermal Free Energies =	-490.944706

Coord:	X	Y	Z
C	0.9574415934	0.0000000000	-1.1508158615
C	0.7874789495	0.0000000000	0.2510327478
C	1.9397365316	0.0000000000	1.0672405934
C	3.2135673669	0.0000000000	0.5109427810
C	3.3637085500	0.0000000000	-0.8771933279
C	2.2340168253	0.0000000000	-1.7009577472
C	-0.4942377394	0.0000000000	0.9350439342
C	-1.7758577573	0.0000000000	0.4603155817
C	-2.1488898836	0.0000000000	-0.9222049947
N	-2.4737756050	0.0000000000	-2.0398171691

C	-2.8670805170	0.0000000000	1.392052
H	-0.4228963099	0.0000000000	2.0201258729
H	1.8230028923	0.0000000000	2.147487265
H	4.0862365710	0.0000000000	1.1562968158
H	4.3564852856	0.0000000000	-1.3168797903
H	2.3493705758	0.0000000000	-2.7802278242
H	0.0982471164	0.0000000000	-1.8088624085
N	-3.7476172017	0.0000000000	2.1525780018

Lowest vibrational frequencies:

	1	2	3
	A"	A"	A'
Frequencies --	99.5868	142.3865	168.3196
Red. masses --	4.9416	5.6946	7.4484
Frc consts --	0.0289	0.0680	0.1243
IR Inten --	0.5799	1.3376	2.4481

Theoretical Calculations of compound 5b

Compound **5b**

Zero-point correction =	0.175866 (Hartree/Particle)
Thermal correction to Energy =	0.186494
Thermal correction to Enthalpy =	0.187438
Thermal correction to Gibbs Free Energy =	0.139216
Sum of electronic and zero-point Energies =	-604.761341
Sum of electronic and thermal Energies =	-604.750714
Sum of electronic and thermal Enthalpies =	-604.749769
Sum of electronic and thermal Free Energies =	-604.797992

Coord:	X	Y	Z
C	1.3424898972	0.0000000000	-1.4271708827
C	0.1107391225	0.0000000000	-0.7867456582
C	0.0174801706	0.0000000000	0.6215040465
C	1.2278708670	0.0000000000	1.3580431428
C	2.4583319711	0.0000000000	0.7316854882
C	2.5281321035	0.0000000000	-0.6728384421
C	-1.2143907155	0.0000000000	1.3769582477
C	-2.5266579863	0.0000000000	0.9821045115
C	-3.5577087154	0.0000000000	1.9780295278

N	-4.3897098097	0.0000000000	2.7919743897
C	-2.9774102951	0.0000000000	-0.3756300544
N	-3.3558267166	0.0000000000	-1.4768166494
H	-1.0782620454	0.0000000000	2.4560134642
H	1.1858021112	0.0000000000	2.4437662807
H	3.3832738174	0.0000000000	1.2976480346
O	3.7737035916	0.0000000000	-1.1985750444
H	1.3726897083	0.0000000000	-2.509661376
H	-0.7849738103	0.0000000000	-1.3945190858
C	3.9238387401	0.0000000000	-2.6161498491
H	4.9982292747	0.0000000000	-2.7997105372
H	3.4780384686	-0.8950625085	-3.0648905377
H	3.4780384686	0.8950625085	-3.0648905377

Lowest vibrational frequencies:

	1	2	3
	A"	A"	A'
Frequencies --	94.0386	108.0116	138.4662
Red. masses --	6.2006	4.8056	6.8051
Frc consts --	0.0323	0.0330	0.0769
IR Inten --	0.0196	1.1698	2.8228

Theoretical Calculations of compound 5c

Compound **5c**	
Zero-point correction =	0.130793 (Hartree/Particle)
Thermal correction to Energy =	0.140490
Thermal correction to Enthalpy =	0.141434
Thermal correction to Gibbs Free Energy =	0.094617
Sum of electronic and zero-point Energies =	-949.816570
Sum of electronic and thermal Energies =	-949.806874
Sum of electronic and thermal Enthalpies =	-949.805929
Sum of electronic and thermal Free Energies =	-949.852747

Coord:	X	Y	Z
C	1.3969418696	0.0000000000	-1.4412100431
C	0.1261412796	0.0000000000	-0.8819508506
C	-0.0456056609	0.0000000000	0.5196480897
C	1.1098176249	0.0000000000	1.3306888498
C	2.3845868537	0.0000000000	0.7807267225
C	2.5196738603	0.0000000000	-0.6085142619

C	-1.3254956096	0.0000000000	1.2050947946
C	-2.6067576880	0.0000000000	0.7295223837
C	-3.6991021205	0.0000000000	1.6596665590
N	-4.5806800806	0.0000000000	2.4188978638
C	-2.9754901629	0.0000000000	-0.6539611466
N	-3.2920973058	0.0000000000	-1.7739447325
H	-1.2533244962	0.0000000000	2.2899440135
H	1.0011976915	0.0000000000	2.4114780316
H	3.2638282396	0.0000000000	1.4143421370
Cl	4.1175289827	0.0000000000	-1.3180932498
H	1.5220153172	0.0000000000	-2.5178268119
H	-0.7305292305	0.0000000000	-1.5432906258

Lowest vibrational frequencies:

	1	2	3
	A"	A"	A'
Frequencies --	87.1098	102.4316	137.2402
Red. masses --	10.2689	5.2291	11.5387
Frc consts --	0.0459	0.0323	0.1280
IR Inten --	2.6905	0.2281	3.1422

Theoretical Calculations of compound 5d

Compound **5d**

Zero-point correction =	0.143976 (Hartree/Particle)
Thermal correction to Energy =	0.154564
Thermal correction to Enthalpy =	0.155508
Thermal correction to Gibbs Free Energy =	0.106999
Sum of electronic and zero-point Energies =	-694.365412
Sum of electronic and thermal Energies =	-694.354825
Sum of electronic and thermal Enthalpies =	-694.353880
Sum of electronic and thermal Free Energies =	-694.402390

Coord:	X	Y	Z
C	1.1051061596	0.0000000000	-1.3783073320
C	-0.1551974414	0.0000000000	-0.7953988677
C	-0.2946315251	0.0000000000	0.6099560480
C	0.8727072018	0.0000000000	1.4049942139
C	2.1367511629	0.0000000000	0.8316844125
C	2.2315665828	0.0000000000	-0.5572979364
C	-1.5646636883	0.0000000000	1.3230979597
C	-2.8514942407	0.0000000000	0.8692089005
C	-3.9285706624	0.0000000000	1.8180639550
N	-4.7976125504	0.0000000000	2.5911307238

C	-3.2469943509	0.0000000000	-0.5076089980
N	-3.5879908974	0.0000000000	-1.6200504470
H	-1.4716973051	0.0000000000	2.4060629952
H	0.7801185418	0.0000000000	2.4867763148
H	3.0382669257	0.0000000000	1.4306719518
N	3.5696897774	0.0000000000	-1.1795063638
H	1.2298878601	0.0000000000	-2.4536203159
H	-1.0257933689	0.0000000000	-1.4375288118
O	3.6237203433	0.0000000000	-2.4073429742
O	4.5429218878	0.0000000000	-0.4281237332

Lowest vibrational frequencies:

	1	2	3
	A"	A"	A'
Frequencies --	91.6826	100.2444	140.7107
Red. masses --	8.2829	5.6125	10.6111
Frc consts --	0.0410	0.0332	0.1238
IR Inten --	6.4915	0.0023	4.9602

Theoretical Calculations of compound 6a

Compound **6a**

Zero-point correction =	0.294131 (Hartree/Particle)
Thermal correction to Energy =	0.309696
Thermal correction to Enthalpy =	0.310640
Thermal correction to Gibbs Free Energy =	0.251701
Sum of electronic and zero-point Energies =	-838.662486
Sum of electronic and thermal Energies =	-838.646921
Sum of electronic and thermal Enthalpies =	-838.645977
Sum of electronic and thermal Free Energies =	-838.704916

Coor:	X	Y	Z
C	-1.4396898091	1.7476451155	-1.7904227069
C	-1.1927936613	1.5762668057	-0.4217165978
C	-2.2451100869	1.7476450587	0.4876481686
C	-3.5258938389	2.0607624043	0.0326953418
C	-3.7695308571	2.2160983722	-1.3327314289
C	-2.7218105044	2.0607624571	-2.2415940931
C	0.1858900362	1.3244865658	0.0657220537
C	0.8446078358	0.1687777267	0.2986140681
C	0.2712651563	-1.2068330217	0.0959065501
O	-1.0013354068	-1.1521542679	-0.354025609

C	2.2618569761	0.3585984858	0.7996877016
O	2.7527317152	1.4600255635	0.9732381986
O	2.9224762632	-0.7795595647	1.0332520678
C	4.2796353199	-0.6322316829	1.5130805928
C	4.8383869001	-2.0277545448	1.710629296
O	0.8488014087	-2.2534195077	0.3000964575
H	0.7772290263	2.2170456077	0.2747921649
H	-0.6270453072	1.6286866632	-2.5007778772
H	-2.9002032679	2.1855232462	-3.3057715621
H	-4.7666773464	2.4623276876	-1.6852762060
H	-4.3335215254	2.1855231563	0.7482616865
H	-2.0595399302	1.6286865620	1.5509257712
H	4.8521148151	-0.0556441246	0.7797717030
H	4.2639832442	-0.0556440761	2.4432578108
H	5.8685977435	-1.9655161077	2.0748641618
H	4.2448376768	-2.5850892722	2.4401569019
H	4.8352748495	-2.5850892912	0.7701498959
C	-1.6435693385	-2.4268286372	-0.5810896979
C	-3.0479982553	-2.1423102369	-1.0776301007
H	-1.0544818472	-2.9948094239	-1.3081375257
H	-1.6423681087	-2.9948096707	0.3546564720
H	-3.5731353124	-3.0844503727	-1.2632943444
H	-3.6139247300	-1.5681940515	-0.3391616921
H	-3.0240070579	-1.5681935298	-2.0077011661

Lowest vibrational frequencies:

	1	2	3
	A	A	A
Frequencies --	76.7902	91.1962	102.3364
Red. masses --	4.9386	4.7599	4.0047
Frc consts --	0.0172	0.0233	0.0247
IR Inten --	2.5611	0.0142	0.3599

Theoretical Calculations of compound 6b

Compound **6b**

Zero-point correction =	0.329858 (Hartree/Particle)
Thermal correction to Energy =	0.347448
Thermal correction to Enthalpy =	0.348392
Thermal correction to Gibbs Free Energy =	0.285101
Sum of electronic and zero-point Energies =	-952.521384
Sum of electronic and thermal Energies =	-952.503795
Sum of electronic and thermal Enthalpies =	-952.502850
Sum of electronic and thermal Free Energies =	-952.566141

Coord:	X	Y	Z
C	-2.4181311551	1.5399317624	-2.0379230401
C	-1.3000341064	1.0318371147	-1.3825161909
C	-0.8307428209	1.6062794069	-0.1856817117
C	-1.5222459572	2.7366057401	0.3091447518
C	-2.6405370741	3.2438277712	-0.3276418410
C	-3.1022476658	2.6444850841	-1.5103777317
C	0.3231627699	1.1519338313	0.5797036402
C	1.0269216345	-0.0066608410	0.6037536169
C	2.1707257715	-0.0668802797	1.5601173186
O	2.9280404712	-1.1695556748	1.3633067872
C	4.0801040226	-1.3036625235	2.2249184655

C	4.7760252212	-2.5992662127	1.8555463228
C	0.7471883842	-1.2224164595	-0.2266987336
O	0.7886292665	-1.2807974219	-1.4406516688
O	0.4222145781	-2.2707432626	0.5533716547
C	0.1940447071	-3.5243232970	-0.1322705839
C	-0.1572843712	-4.5587175964	0.9190272031
O	2.4313369883	0.7793597571	2.3947484856
H	0.6762097430	1.8707767816	1.3182611096
H	-0.7673026097	0.1993444737	-1.8237831609
H	-2.7408656712	1.0765737579	-2.9625709081
O	-4.2038880265	3.2120260924	-2.0691059037
H	-3.1731099759	4.1048032467	0.0617390718
H	-1.1716934440	3.2108552741	1.2217711275
H	4.7285757247	-0.4330958648	2.0847741767
H	3.7446323378	-1.2981490341	3.2666442040
H	5.6608548832	-2.7375854680	2.4845278008
H	4.1115747936	-3.4553755850	2.0033672696
H	5.0954176840	-2.5872228495	0.8096470479
H	-0.6105108013	-3.3872567301	-0.8614408069
H	1.0994120949	-3.7899551251	-0.6870498221
H	-0.3368268326	-5.5271047593	0.4417945655

H	0.6569962330	-4.6748488437	1.6397095547
H	-1.0603616393	-4.2706310652	1.4644112519
C	-4.7195750612	2.6541176993	-3.2706434899
H	-5.5940572552	3.2531294146	-3.5271691156
H	-3.9897190963	2.7112147322	-4.0873828608
H	-5.0241381829	1.6096645217	-3.1313533217

Lowest vibrational frequencies:

	1	2	3
	A	A	A
Frequencies --	63.7983	73.3266	94.6687
Red. masses --	4.7262	3.8914	4.2212
Frc consts --	0.0113	0.0123	0.0223
IR Inten --	1.0028	0.2669	0.5932

Theoretical Calculations of compound 6c

Compound **6c**	
Zero-point correction =	0.284811 (Hartree/Particle)
Thermal correction to Energy =	0.301493
Thermal correction to Enthalpy =	0.302437
Thermal correction to Gibbs Free Energy =	0.240456
Sum of electronic and zero-point Energies =	-1297.580218
Sum of electronic and thermal Energies =	-1297.563536
Sum of electronic and thermal Enthalpies =	-1297.562592
Sum of electronic and thermal Free Energies =	-1297.624573

Coord:	X	Y	Z
C	-2.4520858271	3.1118424003	1.2251572178
C	-1.2487259198	2.7510984960	0.6297635536
C	-1.0309532649	1.4515511355	0.1291326514
C	-2.0898485710	0.5249556056	0.2096664750
C	-3.3023000660	0.8786278071	0.7933190648
C	-3.4731415252	2.1654321917	1.3050985936
C	0.2800050498	1.1820575520	-0.4616548372
C	0.9574014850	0.0359683644	-0.6973941287
C	2.3066980151	0.1764323353	-1.3272947660
O	2.8101155811	-1.0219411851	-1.6894285428

C	4.1038954728	-0.9942965583	-2.3365945053
C	4.4902667792	-2.4279382129	-2.6425230831
C	0.4849816569	-1.3513177065	-0.3717531279
O	1.2601648644	-1.9076424567	0.5746048440
C	0.9438170993	-3.2761592511	0.9313028545
C	1.9349131999	-3.7082202727	1.9936863871
O	-0.4680288022	-1.9103269396	-0.8779991878
O	2.8757770912	1.2322294762	-1.5261412937
H	0.8455091266	2.0744780766	-0.7264465712
H	-0.4521529475	3.4864844311	0.5601529500
H	-2.6020282284	4.1117408327	1.6159880043
Cl	-5.0030232856	2.6062263969	2.0447891749
H	-4.1159416696	0.1637742240	0.8435930308
H	-1.9748586187	-0.4647061659	-0.2136321787
H	4.0297769894	-0.3841846631	-3.2420966234
H	4.8200500018	-0.5063054534	-1.6684919115

H	5.4650728381	-2.4503147353	-3.1392277853
H	4.5583584099	-3.0187825929	-1.7247187017
H	3.7564991793	-2.8985271975	-3.3028359200
H	-0.0895183756	-3.3161756768	1.2890914487
H	1.0060600465	-3.8942337836	0.0304341935
H	1.7298796508	-4.7406327303	2.2930690916
H	2.9598757631	-3.6555896528	1.6161968963
H	1.8610423078	-3.0716281362	2.8797474493

Lowest vibrational frequencies:

	1	2	3
	A	A	A
Frequencies --	60.6230	72.0145	91.2721
Red. masses --	6.1833	4.2511	4.7923
Frc consts --	0.0134	0.0130	0.0235
IR Inten --	0.2266	0.1489	0.4212

Theoretical Calculations of compound 6d

Compound **6d**

Zero-point correction =	0.297297 (Hartree/Particle)
Thermal correction to Energy =	0.314903
Thermal correction to Enthalpy =	0.315847
Thermal correction to Gibbs Free Energy =	0.252276
Sum of electronic and zero-point Energies =	-1042.124171
Sum of electronic and thermal Energies =	-1042.106565
Sum of electronic and thermal Enthalpies =	-1042.105621
Sum of electronic and thermal Free Energies =	-1042.169191

Coord:	X	Y	Z
C	-0.5300652914	1.2112112502	2.0862635567
C	-0.0102455966	-0.0001110307	1.6052803240
C	-0.5308762797	-1.2113108081	2.0856951669
C	-1.5682880269	-1.2176135373	3.0110645333
C	-2.0733988331	0.0001445864	3.4608757576
C	-1.5674777304	1.2177755378	3.0116294148
C	1.1586207639	-0.0002812730	0.6928613536
C	1.1917178030	-0.0001720916	-0.6560649553
C	-0.0990937937	0.0001965973	-1.4310326939
O	-1.1880444270	0.0007866534	-0.8888863943

C	2.5895915272	-0.0004376588	-1.2391546657
O	3.5854230895	-0.0009413061	-0.5382495305
O	2.6400092333	0.0003093449	-2.5761310789
C	3.9711712520	0.0002914762	-3.1502056463
C	3.8135237617	0.0011424054	-4.6576183623
O	0.0537769416	-0.0004813572	-2.7632810324
C	-1.1749416848	-0.0003145325	-3.5311880928
C	-0.7909567115	-0.0011917064	-4.9975745322
H	2.1366237640	-0.0005574020	1.1743587209
H	-0.1255740019	2.1511999983	1.7258747886
H	-1.9876867643	2.1413883048	3.3887908182
N	-3.1667320352	0.0002783355	4.4425749929
H	-1.9891084009	-2.1411209555	3.3878026090
H	-0.1270132396	-2.1514007748	1.7248659206
H	4.5084999339	0.8820940584	-2.7884948249
H	4.5080614361	-0.8821672655	-2.7894451693
H	4.8000104327	0.0011412465	-5.1312245842
H	3.2695447149	-0.8853766601	-4.9950242856
H	3.2700060288	0.8883058137	-4.9940743467
H	-1.7616780840	-0.8818675903	-3.2555924281
H	-1.7609024535	0.8820194522	-3.2564430406
H	-1.6940223973	-0.0010881189	-5.6155737843

H	-0.2017801132	0.8851992724	-5.2483193215
H	-0.2025707604	-0.8883492412	-5.2474646670
O	-3.5922017360	1.0903313200	4.8257895911
O	-3.5928347019	-1.0896726270	4.8253774068

Lowest vibrational frequencies:

	1	2	3
	A	A	A
Frequencies --	68.5457	73.1474	102.2378
Red. masses --	7.3793	4.9678	4.0324
Frc consts --	0.0204	0.0157	0.0248
IR Inten --	0.5598	0.2415	0.6242
